# 3,4-Dihydropyrimidin-2(1*H*)-One Hybrids as Phenotypic Anti-Breast Cancer Agents: Synthesis, Colony-Formation Profiling and Computational Target Deconvolution

**DOI:** 10.3390/ijms27219618

**Published:** 2026-10-27

**Authors:** Hadeel D. Sultan, Rocco Buccheri, Anas J. Rasras, Mohammad S. Alzubi, Eyad A. Younes, Placido Mineo, Maria S. Molonia, Francesco Cimino, Antonio Rescifina, Nicola Micale

**Affiliations:** 1 Department of Chemistry, Faculty of Science, Al-Balqa Applied University, P.O. Box 206, Al-Salt 19117, Jordan; 52207039007@std.bau.edu.jo (H.D.S.); rasras.chem@bau.edu.jo (A.J.R.); 2 Department of Drug and Health Sciences, University of Catania, Viale A. Doria 6, 95125 Catania, Italy; rocco.buccheri@unict.it; 3 Department of Chemistry, Faculty of Science, The Hashemite University, P.O. Box 330127, Zarqa 13133, Jordan; e.younes@hu.edu.jo; 4 Department of Chemical Sciences, University of Catania, Viale A. Doria 6, 95125 Catania, Italy; placido.mineo@unict.it; 5 Department of Chemical, Biological, Pharmaceutical and Environmental Sciences, University of Messina, Viale F. Stagno D’Alcontres 31, 98166 Messina, Italy; mariasofia.molonia@unime.it (M.S.M.); francesco.cimino@unime.it (F.C.); nicola.micale@unime.it (N.M.)

**Keywords:** 3,4-Dihydropyrimidin-2(1*H*)-ones, MCF-7 breast cancer cells, phenotypic screening, colony-formation assay, TOP2A target deconvolution

## Abstract

3,4-Dihydropyrimidin-2(1*H*)-ones (DHPMs) are privileged heterocyclic scaffolds with broad biological activities. Sixteen DHPM hybrids (**4a**–**p**) carrying *para*- or *ortho*-aryloxy linkers and diverse terminal amines were prepared through a modular propargylation, Mannich-type, and Biginelli sequence. The compounds were ranked by NIH/3T3 cytotoxicity, MCF-7 antiproliferative activity (SRB assay), and colony formation under continuous exposure at the MCF-7 IC_25_. Compounds **4a** and **4m** were the most potent in MCF-7 cells (IC_50_ = 10.7 and 11.6 µM; preliminary selectivity indices of 9.6 and 9.1), but **4m** inhibited colony formation by 68.3% compared with 25.3% for **4a**, supporting its prioritization as the most balanced lead. Ligand-based target prediction followed by GNINA screening of seven candidate targets identified TOP2A as the best match to the experimental pIC_50_ ranking (Pearson r = 0.883, Spearman ρ = 0.893, *n* = 7), with Eg5/KIF11 as a secondary comparator. Docking, 200 ns molecular dynamics, and MM/PBSA estimates were compatible with this hypothesis but did not constitute experimental target validation. These data identify **4m** as a starting point for DHPM-based antiproliferative agents and TOP2A as a hypothesis to be tested experimentally.

## 1. Introduction

3,4-Dihydropyrimidin-2(1*H*)-ones (DHPMs) are a privileged class of heterocyclic compounds that have attracted sustained interest in medicinal chemistry because of their synthetic accessibility, broad functionalization patterns, and documented biological relevance [1,2,3,4]. In particular, the Biginelli reaction provides rapid access to structurally diverse DHPM derivatives, making this scaffold especially attractive for preparing focused small-molecule libraries suitable for preliminary structure–activity relationship studies [5,6,7]. Recent work has continued to exploit this scaffold in breast cancer models: C5-substituted fatty-acid DHPMs designed from the Eg5 inhibitor monastrol inhibited MCF-7 cell growth with an IC_50_ as low as 2.3 µM [8].

Breast cancer accounted for 11.6% of all new cancer cases worldwide in 2022 and was the second most frequently diagnosed cancer overall after lung cancer [9]. Cytotoxic regimens in advanced cancer carry a substantial burden of cutaneous and cardiac adverse effects [10,11,12], which sustains the need for new chemotypes with a more favorable balance between antiproliferative activity and tolerability. Recent studies have uncovered new vulnerabilities and delivery strategies in breast cancer, including MAEL-dependent metabolic reprogramming that supports breast cancer progression [13] and nanoplatforms that enhance natural killer cell responses against triple-negative breast cancer [14]. Compounds that modulate ferroptosis and PI3K/AKT/mTOR signaling in cervical cancer [15] and strategies that address the immune microenvironment of ovarian cancer [16] illustrate the breadth of approaches under investigation in oncology. Small molecules and peptides are also being pursued in infectious diseases, as shown by recent work on antiviral agents against chikungunya virus [17] and antimicrobial peptides that activate macrophages [18]. Several of the targets examined in this study are already clinically exploited. Topoisomerase II is the target of established DNA-damaging agents [19]; Eg5/KIF11 inhibitors, such as ispinesib, filanesib, and litronesib, have reached clinical trials, with phase II as the most advanced stage [20]; the PI3Kα-selective inhibitor alpelisib extended median progression-free survival from 5.7 to 11.0 months in PIK3CA-mutated, hormone receptor-positive advanced breast cancer [21]; HDAC inhibitors, including entinostat and tucidinostat, have been evaluated in breast cancer trials [22].

In anticancer drug discovery, phenotypic screening remains a valuable strategy for identifying lead compounds when the primary molecular target is unknown a priori [23,24]. In this context, compact heterocyclic scaffolds endowed with tunable polarity, conformational adaptability, and peripheral diversification are particularly useful, as they allow the exploration of biologically relevant chemical spaces while maintaining synthetic tractability [1,2,3,4]. DHPMs fit well within this framework because they can be combined with different substituent patterns and appended motifs to probe the steric, electronic, and physicochemical determinants of their activity.

In this study, we designed a modular DHPM-based library featuring three conserved elements: a 3,4-dihydropyrimidin-2(1*H*)-one core, an aryloxy fragment, and a propargyl-linked terminal amine. This design was intended to allow systematic variations in the molecular shape, basicity, polarity, aromaticity, and hydrogen-bonding potential. Additionally, *para*- and *ortho*-substituted aryloxy derivatives were synthesized to assess the impact of regioisomeric orientation on their biological activity. The terminal amino group was diversified to include arylpiperazine, alkylpiperazine, morpholine, piperidine, bulky dialkylamino, and amide-containing motifs in the molecular structure. Together, these modifications produced a focused set of analogs suitable for phenotypic medicinal chemistry studies.

Importantly, this study was not designed using a strict single-target design strategy. Instead, the compounds were synthesized as a structurally coherent exploratory library and were subsequently evaluated using a phenotypic workflow. A preliminary cytotoxicity screen against NIH/3T3 fibroblasts was used to identify compounds with a more favorable safety profile, followed by antiproliferative assessment in MCF-7 breast cancer cells and a colony formation assay under continuous exposure, which provided an orthogonal phenotypic measure of colony-forming ability. This approach revealed non-uniform biological behavior across the series and enabled the prioritization of compound **4m** as the most balanced lead when antiproliferative potency, selectivity, and colony formation inhibition were considered together.

Accordingly, this study represents a phenotypically driven medicinal chemistry investigation complemented by computational target deconvolution. It addresses three questions: how the regiochemistry of the aryloxy linker and the identity of the terminal amine influence the antiproliferative profile of the DHPM–propargylamine scaffold; whether the ranking obtained in a bulk-cell assay (SRB) is preserved in a colony-formation assay under continuous exposure; and which candidate targets can rationalize the observed ranking. In silico analyses were intended to generate hypotheses about potential protein targets that could explain the observed biological ranking within the active subset rather than provide definitive target validation.

## 2. Results and Discussion

### 2.1. Chemistry and Library Design

**Scheme 1:**
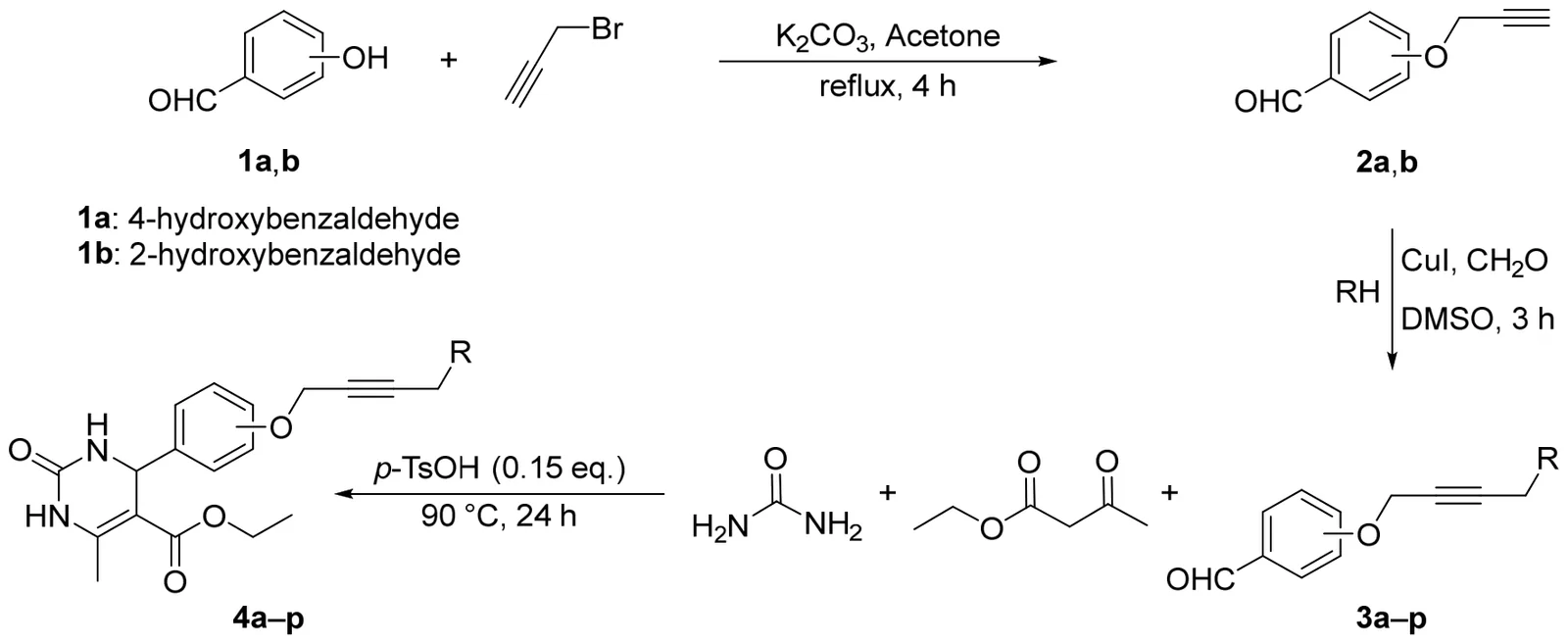
General synthetic route to 3,4-dihydropyrimidin-2(1*H*)-one hybrids **4a**–**p**.

Target DHPM hybrids **4a**–**p** were synthesized through a concise modular sequence starting from hydroxybenzaldehyde derivatives. As outlined in Scheme 1, *O*-propargylation of the parent hydroxybenzaldehydes afforded intermediates **2a**,**b** in quantitative yield. These intermediates were subsequently subjected to Mannich-type functionalization with secondary amines and formaldehyde in the presence of CuI to provide the corresponding amino-substituted aldehydes **3a**–**p** [25]. The target compounds were finally assembled through a Biginelli-type condensation of intermediates **3a**–**p** with ethyl acetoacetate and urea under *p*-toluenesulfonic acid catalysis, thus furnishing the desired 3,4-dihydropyrimidin-2(1*H*)-one hybrids **4a**–**p** in moderate to good overall yield (Table 1) [26].

Because condensation was performed under achiral conditions, compounds **4a**–**p** were obtained as racemic mixtures, and no enantiomeric resolution was performed on them.

From a design perspective, this synthetic route is advantageous because it allows for late-stage modification of the terminal amino group while maintaining a consistent DHPM backbone across the series. This strategy enabled the creation of a focused library to systematically examine the effects of regioisomerism and terminal amine identity on biological activity. Specifically, *para*- and *ortho*-substituted aryloxy subseries were designed to investigate how the orientation of the propargylamino side chain influences biological activity, while different terminal amino groups were chosen to explore the impact of aromaticity, steric bulk, basicity, and polarity on the activity and selectivity.

### 2.2. Structural Characterization

The structures of compounds **4a**–**p** were confirmed by ^1^H NMR, ^13^C NMR, DEPT-135, and HRMS analyses. In the ^1^H NMR spectra, the DHPM-containing products consistently displayed diagnostic resonances of the ethyl ester moiety, including a quartet for the methylene group and a triplet for the terminal methyl group, along with a singlet attributable to the C6 methyl substituent. The methine proton of the DHPM ring was typically observed as a singlet or, in some cases, as a weakly split signal owing to coupling with a neighboring NH proton. In the spectra recorded in DMSO-d_6_, the two DHPM NH resonances were generally observed in the downfield region, with N1–H typically occurring at approximately δ = 9.1–9.2 and N3–H at approximately δ = 7.7–8.1. These assignments were based on chemical shifts, integration, and comparison with related DHPM systems [27,28]. Where resolved, the coupling between H-4 and N3–H provided additional support for this assignment. Because no dedicated D_2_O-exchange experiments were performed, these resonances were reported as spectroscopic assignments rather than being independently confirmed by exchange measurements.

Further support for the assigned structures was provided by the ^13^C NMR spectra, which showed the expected signals for the ester carbonyl, ureide carbonyl, aromatic carbons, and alkyne carbons of the propargyl linker. The methylene groups adjacent to the oxygen and nitrogen were readily identified in the aliphatic region. Overall, the spectroscopic data were consistent with the proposed structures and confirmed the successful construction of the designed DHPM library.

### 2.3. Initial Cytotoxic Screening and Hit Prioritization

To preliminarily assess whether the synthesized DHPM hybrids were compatible with further anticancer evaluation, the compounds were screened for basal cytotoxicity against NIH/3T3 mouse fibroblasts. This step was intended as a pragmatic prioritization filter, rather than a definitive safety assessment. Applying a 100 µM cutoff to the CC_50_ point estimates (Table 2 and Figure S1, reported with 90% confidence limits, CL), seven derivatives, **4a**, **4e**, **4f**, **4h**, **4i**, **4m**, and **4o**, were considered suitable for further biological profiling, whereas the remaining compounds displayed lower CC_50_ values and were not evaluated further. Although the lower 90% confidence bounds of **4a** and **4m** were marginally below 100 µM, their CC_50_ point estimates exceeded the predefined cutoff, and both compounds were retained for testing in MCF-7 cells. The corresponding SIs (9.6 and 9.1, respectively; Table 3) further support this prioritization. These indices should be regarded as preliminary. CC_50_ values were determined in murine NIH/3T3 fibroblasts after 24 h of exposure, whereas IC_50_ values were determined in human MCF-7 cells after six days of exposure. A non-tumorigenic human breast epithelial line, such as MCF-10A, was not included.

This initial selection indicates that the biological behavior of this series is highly sensitive to changes in the peripheral structure. Even small modifications to the terminal amino group or aryl substitution pattern can shift a compound from being relatively well tolerated to being more cytotoxic. Therefore, the NIH/3T3 assay narrowed the focus to compounds with a more favorable preliminary safety profile.

### 2.4. Antiproliferative Activity Against MCF-7 Cells

Seven compounds prioritized through NIH/3T3 screening were evaluated for their antiproliferative activity against MCF-7 human breast cancer cells. As shown in Table 3 and Figure S2, all selected derivatives were active, but their potency differed markedly within this subset. Compounds **4a** and **4m** were the most potent derivatives, with IC_50_ values of 10.7 and 11.6 µM, respectively, whereas compound **4i** showed intermediate activity (IC_50_ = 43.6 µM). Compound **4h** was less active but retained a measurable antiproliferative effect (IC_50_ = 75.5 µM), whereas **4e** and **4f** were substantially weaker, and **4o** exhibited only weak antiproliferative activity in MCF-7 cells.

In addition to the NIH/3T3 data, these results identified **4a** and **4m** as the most promising compounds in the biologically characterized subset. Notably, **4a** and **4m** exhibited the lowest MCF-7 IC_50_ values and the highest SIs within the subset, despite their comparatively modest NIH/3T3 CC_50_ values. Consequently, they were selected as the main candidates for further investigations. However, the IC_50_ values obtained from the bulk-cell SRB assay alone were insufficient to define the best lead compound, as they did not account for possible differences in colony-forming ability with continuous exposure.

### 2.5. Colony-Formation Profiling Under Continuous Exposure Supports the Prioritization of ***4m*** as the Most Balanced Lead

To complement the bulk-cell SRB assay with an orthogonal measure of colony-forming ability, the selected compounds were evaluated at their corresponding MCF-7 IC_25_ concentrations in a six-day colony formation assay under continuous exposure (Table 4). Compound **4o** was not subjected to this assay because it exhibited the weakest antiproliferative activity within the biologically prioritized subset and was therefore considered a low-priority candidate for further phenotypic characterization. The colony formation assay revealed a compound ranking that differed from that obtained using the SRB antiproliferative endpoint, providing an orthogonal criterion for lead prioritization.

Compound **4m** produced the highest mean inhibition of colony formation, reducing the number of colonies by 68.3%. When considered together with its MCF-7 IC_50_ value and SI, this result supports the prioritization of **4m** as the most balanced derivative in the biologically characterized subset. In contrast, compound **4a**, despite displaying antiproliferative potency comparable to that of **4m** in the SRB assay, reduced colony formation by 25.3%. This difference indicates that the relative performances of **4a** and **4m** depend on the experimental format and cellular growth conditions.

Compounds **4h** and **4i** showed intermediate but appreciable colony formation inhibition, with values slightly above 50%. Compound **4e** also substantially inhibited colony formation despite its weaker IC_50_ in the SRB assay. In contrast, **4f** exhibited weak activity in both phenotypic assays, while **4o** was not evaluated due to its limited MCF-7 antiproliferative activity.

**Figure 1:**
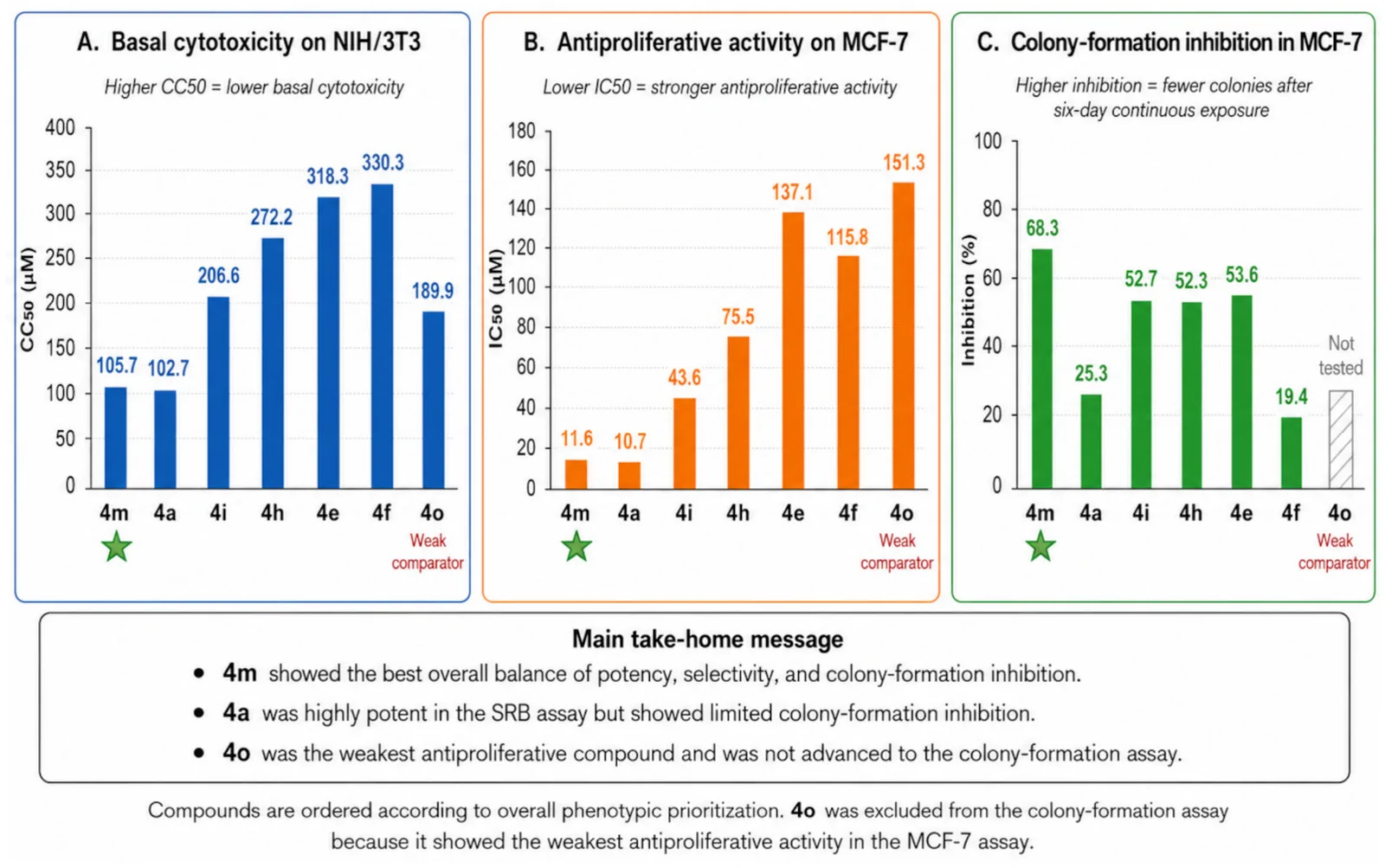
Comparative phenotypic profiles of the selected compounds based on NIH/3T3 cytotoxicity, MCF-7 antiproliferative activity, and colony formation inhibition under continuous exposure. The green star denotes the prioritized lead compound **4m**.

Overall, these findings show that the series does not behave as a simple potency continuum across the two phenotypic assays. Some compounds were favored in the bulk-cell SRB assay, whereas others effectively inhibited colony formation during continuous exposure to the drug. This assay-dependent behavior supports the prioritization of **4m** over **4a**, without implying post-treatment persistence or an irreversible loss of reproductive capacity. The assay-dependent differences and the resulting lead prioritization are summarized in Figure 1.

### 2.6. Phenotypic Structure–Activity Relationships

Although the present compound series was not developed through a classical target-based optimization campaign, the biological results allowed the identification of several preliminary phenotypic structure–activity relationships. In particular, the nature of the terminal amino group markedly influenced both the MCF-7 antiproliferative activity and colony formation inhibition. Arylpiperazine-containing derivatives were particularly informative, as this subgroup included the two most potent compounds in the SRB assay, **4a** and **4m**. This finding indicates that the introduction of an aromatic substituent on the piperazine ring is compatible with strong antiproliferative activity. However, the different behaviors of **4a** and **4m** in the colony-formation assay show that subtle variations in aromatic identity, substitution pattern, and overall side-chain geometry can substantially modify the phenotypic profile across different experimental formats.

A comparison of the *para*- and *ortho*-substituted aryloxy derivatives further indicated that the regiochemistry is not a neutral structural feature. The position of the propargylamino-containing substituent is expected to affect the overall molecular shape, conformational preferences, and spatial projection of the terminal amino group. Within this context, *ortho*-pyridylpiperazine derivative **4m** displayed the most balanced profile, combining a low MCF-7 IC_50_ value, favorable SI, and the highest mean inhibition of colony formation under continuous exposure. In contrast, the *para*-methoxyphenylpiperazine analog **4a** showed comparable potency in the SRB assay but substantially lower colony formation inhibition. This assay-dependent difference suggests that the structural features of **4m** are more favorable for maintaining activity in the colony formation assay without implying post-treatment persistence or irreversible clonogenic damage.

Morpholine derivatives provide particularly informative regioisomeric comparisons. The *para*-substituted compound **4h** exhibited greater antiproliferative activity than its *ortho*-substituted counterpart **4o**, supporting the hypothesis that the optimal orientation of the terminal cyclic amine depends on both its chemical identity and regiochemistry of the aryloxy linker. The piperidine derivative **4i** also displayed an intermediate profile in both assays, indicating that a non-aromatic cyclic amine could retain measurable activity but was insufficient to reproduce the performance of the most active arylpiperazine-containing compound.

Additional differences emerged among the polar piperazine derivatives. Compound **4e** showed weak antiproliferative potency in the SRB assay but appreciable inhibition of colony formation, demonstrating that the ranking of compounds depends on the biological endpoint. In contrast, the amide-containing derivative **4f** exhibited comparatively weak activity in both phenotypic assays, indicating that increased polarity alone does not improve the cellular activity. Overall, the results suggest that the most favorable phenotypic profile arises from the combined contribution of the conserved DHPM core, regiochemical orientation of the aryloxy linker, and terminal arylpiperazine group, providing an appropriate balance of aromaticity, polarity, basicity, and conformational adaptability.

**Figure 2:**
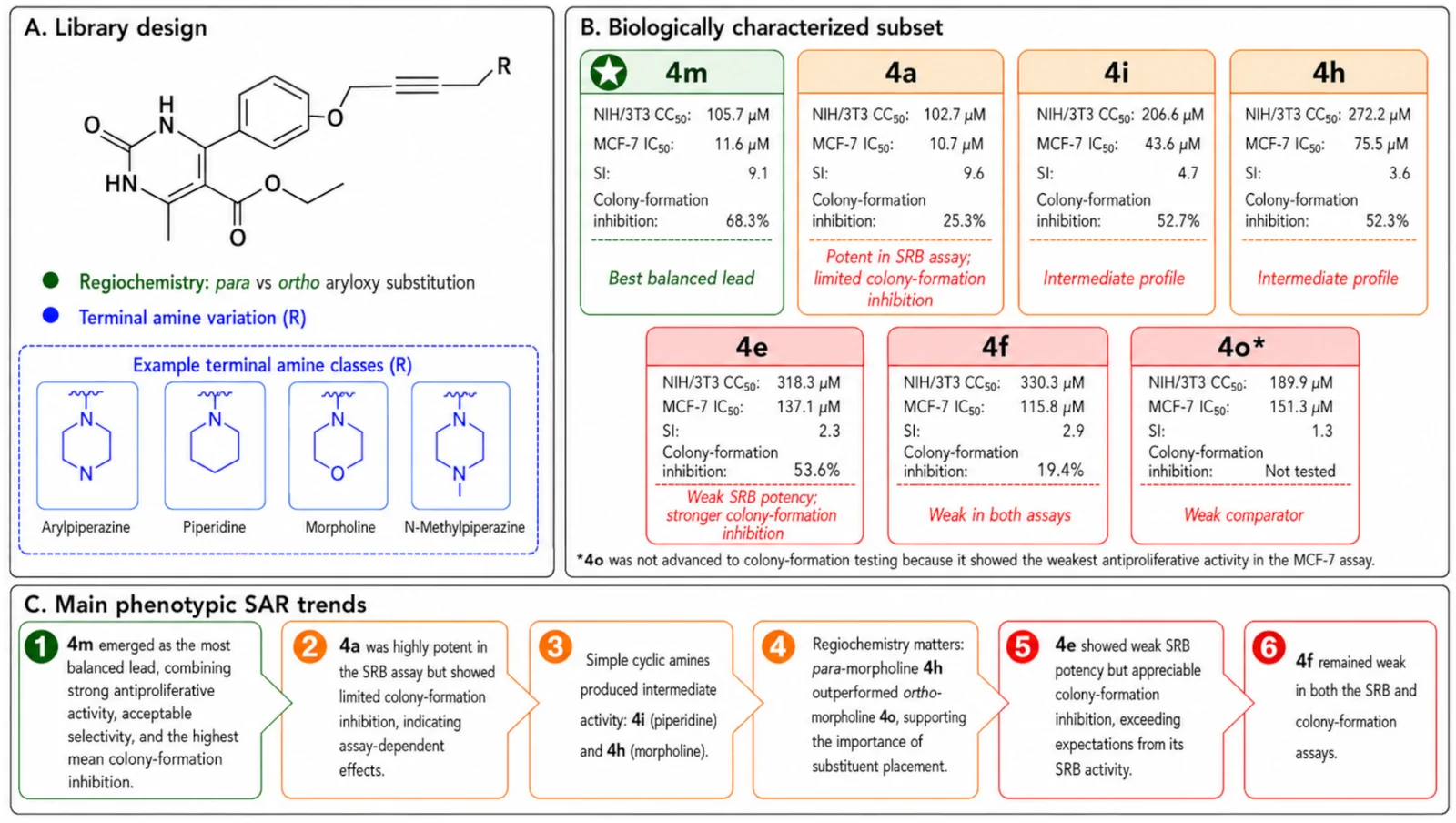
Phenotypic SAR summary of the DHPM series. The green star identifies the prioritized lead compound **4m**. Colors distinguish the phenotypic prioritization groups: green, prioritized lead; orange, favorable/intermediate profiles; red, lower-priority profiles.

The principal matched-pair comparisons and resulting phenotypic SAR trends are illustrated in Figure 2.

### 2.7. Computational Target Deconvolution of the Active Subset

To generate mechanistic hypotheses capable of rationalizing the phenotypic ranking of the biologically characterized subset (**4a**, **4e**, **4f**, **4h**, **4i**, **4m**, and **4o**), a computational target deconvolution workflow combining ligand-based prediction and preliminary structure-based screening was applied. This analysis was explicitly conceived as a hypothesis-generating strategy rather than a definitive target identification.

Ligand-based target prediction using SuperPred 3.0 [29] identified DNA topoisomerase I as the most recurrent cancer-related prediction across the subset, while DNA topoisomerase IIα (TOP2A), Aurora kinase B, PI3Kα, HDAC8, and HDAC10 emerged as additional hypotheses (Table S1). Eg5/KIF11, which was not among the SuperPred predictions, was included as an orthogonal mitotic comparator because of its established relevance to the DHPM scaffold class [30,31]. These seven targets were subsequently evaluated by GNINA using CNN affinity values on ligand-bound crystal or cryo-EM structures validated by redocking (Section 3.5.2 and Table S2), and their agreement with the experimental MCF-7 pIC_50_ ranking was assessed using Pearson and Spearman correlation analyses.

**Figure 3:**
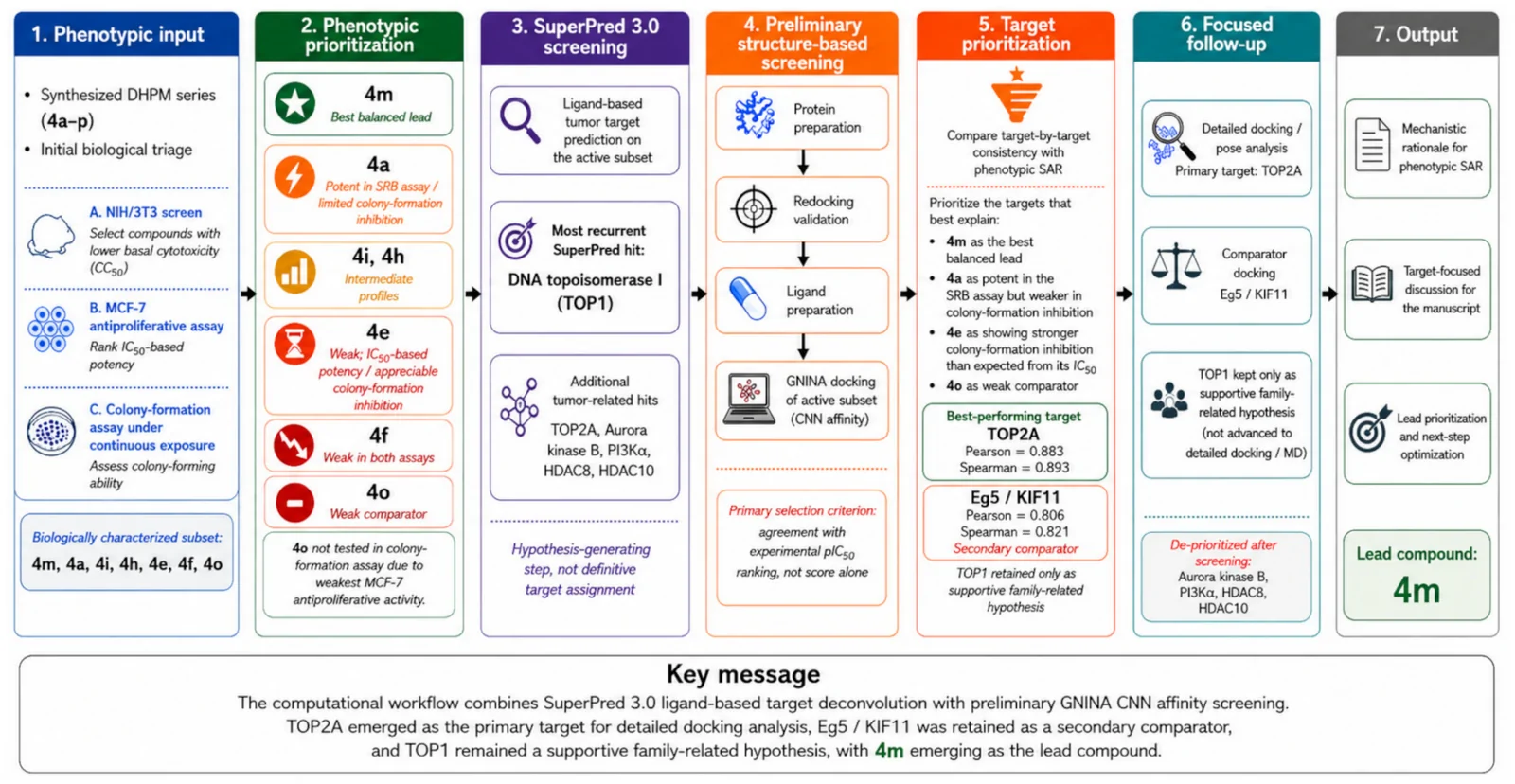
Computational target deconvolution workflow. Phenotypic prioritization-guided target hypothesis generation and preliminary target screening.

Among the screened targets, TOP2A showed the strongest agreement with the experimental ranking (Pearson r = 0.883; Spearman ρ = 0.893), followed by Eg5/KIF11 (r = 0.806; ρ = 0.821) (Table 5 and Figure S3). HDAC10 showed intermediate agreement (r = 0.679; ρ = 0.541), whereas DNA topoisomerase I, despite being the most recurrent SuperPred prediction, showed only moderate structure-based agreement (r = ρ = 0.500). Aurora kinase B, PI3Kα, and HDAC8 poorly correlated with the phenotypic data (SuperPred output is provided in Table S1). For Aurora kinase B, the discrepancy between the Pearson and Spearman coefficients (0.503 vs. 0.107) indicates that the apparent linear trend is not preserved on a rank basis. Therefore, TOP2A was selected as the primary mechanistic hypothesis for focused structural analysis, whereas Eg5/KIF11 was retained as a secondary mechanistic comparison. Two-sided *p*-values were 0.008 (Pearson) and 0.007 (Spearman) for TOP2A and 0.029 and 0.023 for Eg5/KIF11; no other target reached *p* < 0.05. Because seven targets were tested, the *p*-values for TOP2A corresponded to approximately 0.059 (Pearson) and 0.048 (Spearman) after Bonferroni correction. Given the limited sample size (*n* = 7) and the exploratory nature of the analysis, these correlations were used solely to prioritize mechanistic hypotheses and should not be interpreted as evidence for experimental validation. The overall target deconvolution workflow is illustrated in Figure 3.

### 2.8. Docking Analysis of the Prioritized Target Hypothesis

Detailed docking analysis was performed on TOP2A, the primary target hypothesis identified in Section 2.7. Eg5/KIF11 was analyzed in parallel as a secondary comparator.

**Figure 4:**
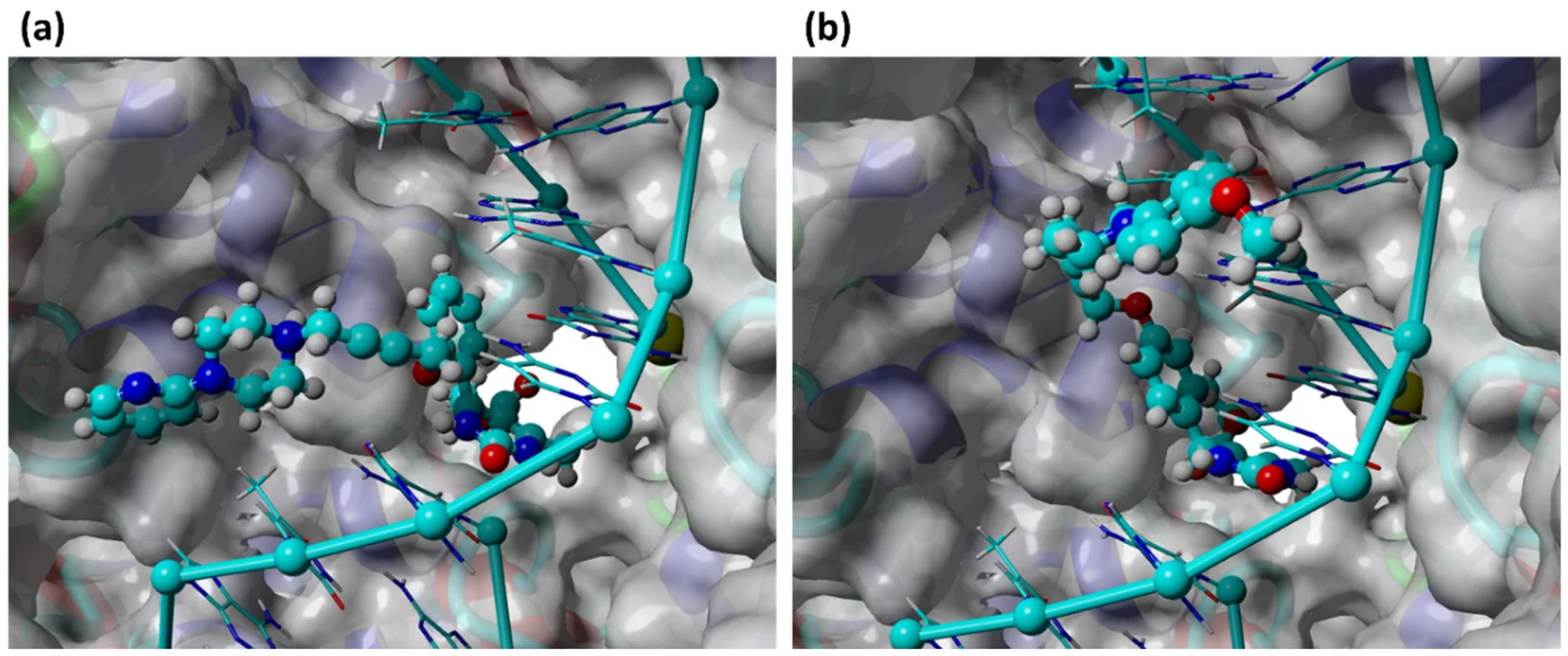
Representative predicted docking poses of lead compound **4m** (**a**) and potent SRB-active analog **4a** (**b**) within the DNA cleavage site of TOP2A, highlighting the intercalation-type binding mode predicted for the DHPM core.

All seven compounds adopted predicted intercalation-type poses within the TOP2A DNA cleavage site, which is the site occupied by etoposide in the 6ZY5 structure used for redocking (RMSD = 2.12 Å; Section 3.5.2). The docking of dihydropyrimidin-2(1*H*)-thione derivatives to a DNA-bound structure of human TOP2A (PDB 4FM9) has also been reported [32]. The DHPM core was positioned between adjacent nucleobases, whereas the aryloxy–propargyl–amine substituent modulated the orientation and local complementarity of the ligands within the binding region. Compounds **4a** and **4m** displayed the highest TOP2A CNN affinity values of the series, 6.57 and 6.29, respectively, consistent with their strong antiproliferative activity in the MCF-7 SRB assay. Representative predicted binding modes of these compounds are shown in Figure 4.

Docking to Eg5/KIF11 revealed additional compound-specific interaction patterns. Compound **4m** formed hydrogen-bond contacts with Glu118 and Leu214, a charge-assisted interaction with Glu116, and additional aromatic and hydrophobic contacts. In contrast, **4a** interacts mainly with Arg119 and Arg221 and shows a charge interaction with Glu118. The remaining compounds retained subsets of these interactions with target proteins. The complete residue interaction profiles are listed in Table S3. Eleven of the 14 Eg5 residues contacted by the docked compounds (Table S3) lie within 4.5 Å of monastrol in its crystal structure with Eg5 [33], that is, in the allosteric pocket that accommodates monastrol and other DHPM-type inhibitors [30,31]; the exceptions were Phe113, Pro121, and Arg221. The co-crystallized inhibitor of 6G6Y [34] contacts ten of these 14 residues. Overall, Eg5 docking provided complementary information but did not supersede TOP2A as the primary mechanistic hypothesis.

Taken together, the docking results support TOP2A as the primary structure-based mechanistic hypothesis, while Eg5/KIF11 remains a secondary comparator, providing complementary information on compound-specific binding differences between the two targets.

### 2.9. Molecular Dynamics and Comparative Binding-Energy Analysis

To examine the dynamic behaviors of the prioritized complexes, 200 ns MD simulations were performed for compounds **4a**, **4i**, **4m**, and **4o** in complex with TOP2A and Eg5/KIF11; these four compounds spanned the MCF-7 potency range of the profiled subset (pIC_50_ = 4.97 for **4a**, 4.94 for **4m**, 4.36 for **4i**, and 3.82 for **4o**). The potential energy profiles showed no systematic drift over the simulated interval, indicating the absence of significant energetic instability.

The Eg5 trajectories were comparatively heterogeneous in nature. Compounds **4i** and **4a** approached more stable RMSD regimes after approximately 110 and 140 ns, respectively, whereas **4m** and **4o** did not reach a fully stable RMSD plateau within 200 ns. The corresponding R_g_ profiles exhibited greater structural variability, particularly for **4m** and **4o**. These results were less consistent with the overall phenotypic ranking and supported the use of Eg5 primarily as a secondary comparison. The complete Eg5 potential energy profiles, RMSD, and Rg values are shown in Figure S4.

**Figure 5:**
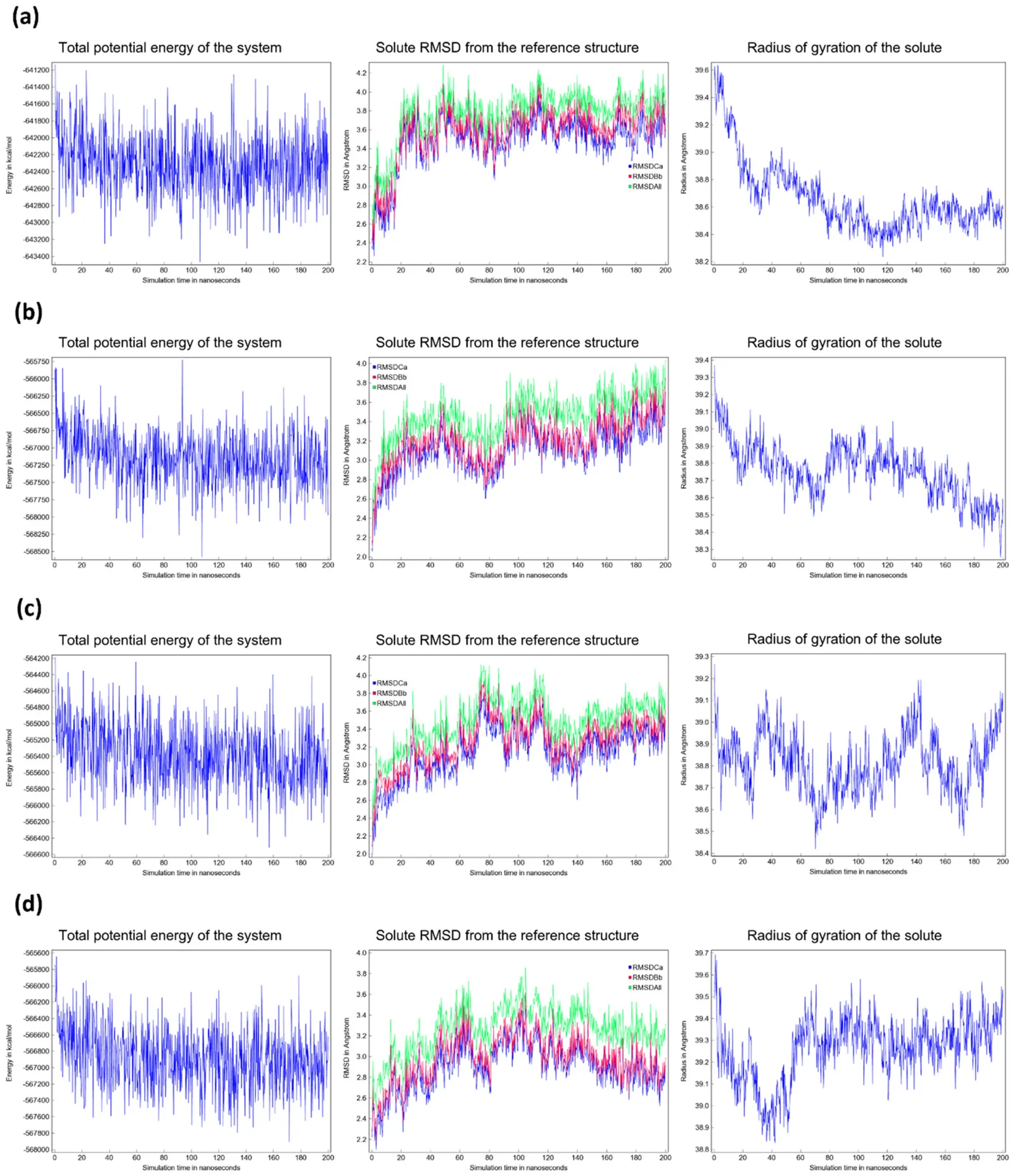
MD results for the TOP2A protein. **Left**: total potential energy of the system as a function of time (200 ns) for the ligand–protein complexes. **Center**: solute RMSD from the reference structure as a function of time (200 ns). The plot shows the alpha carbons (RMSDCa, blue), backbone (RMSDBb, red), and all-heavy atom (RMSDAll, green) RMSDs. **Right**: R_g_ as a function of time (200 ns). Panels: (**a**) **4a**, (**b**) **4i**, (**c**) **4m**, (**d**) **4o**.

In contrast, the TOP2A complexes showed more convergent dynamic behavior (Figure 5). All four ligands approached an overall RMSD steady-state regime, although with different equilibration patterns. Compound 4a showed the fastest convergence and a pronounced decrease in R_g_, consistent with the formation of a compact intercalative complex. Compound **4i** also showed progressive stabilization, whereas 4o exhibited a less compact final profile. Compound 4m remained more conformationally dynamic than **4a**, but its fluctuations occurred within an overall convergent TOP2A framework, consistent with an adaptive intercalative binding mode.

The comparative MM/PBSA energy of the binding estimates was obtained from the MD trajectories (Table 6). According to the YASARA energy convention, TOP2A–**4m** showed the most favorable average value (91.4 kcal/mol), followed by **4i** (46.7 kcal/mol), **4o** (45.1 kcal/mol), and **4a** (25.7 kcal/mol). This ordering is compatible with the higher colony formation inhibition observed for **4m** relative to **4a**, although it should not be interpreted as evidence of a causal relationship, experimental binding affinity, or ligand residence time. The comparison remains exploratory because it is based on one trajectory per complex, and compound **4e**, an important phenotypic outlier, was not included in the MD analysis.

For Eg5, the corresponding average values were 297.7 kcal/mol for **4a**, 287.3 kcal/mol for **4m**, 245.9 kcal/mol for **4o**, and −29.8 kcal/mol for **4i**. Frame-by-frame inspection of **4i** indicated that this negative average was not driven by a small number of outlier snapshots: negative BindEnergy values were recorded for the large majority of the 800 analyzed frames (median = −31.8 kcal/mol), with only a limited subset of markedly favorable snapshots, concentrated mainly between 150 and 180 ns, coinciding with abrupt frame-to-frame shifts in the complex solvation terms. This pattern suggests transient fluctuations in the implicit solvation contribution rather than a sustained favorable binding mode, consistent with the comparatively unstable and non-convergent character already noted for the Eg5 trajectories. Absolute energies were not compared between TOP2A and Eg5 because the two receptor systems differ substantially in composition, size, charge, and, in the case of TOP2A, the presence of a DNA duplex. Moreover, the Eg5 energy ranking did not reproduce the experimental phenotypic ordering, further supporting its role as a secondary comparator.

PCA provided an additional description of the conformational space sampled during the simulations (Paragraph S1). For TOP2A, **4m** sampled a comparatively confined and recurrent conformational region, whereas **4a** explored a broader and more directionally shifted conformational space; **4i** and **4o** displayed intermediate patterns. In contrast, the Eg5 complexes showed broader and less convergent conformational landscapes without a clear relationship to the experimental ranking. Complete PCA analyses are provided in Figures S5 and S6. Collectively, the MD, MM/PBSA, and PCA results support the prioritization of TOP2A as the principal computational mechanistic hypothesis, while Eg5/KIF11 remains a complementary secondary comparator.

### 2.10. In Silico Physicochemical and Liability Assessment

The physicochemical and predicted developability profiles of the three most relevant compounds (**4a**, **4i**, and **4m**) were evaluated using DataWarrior and SwissADME (Table 7). The calculated LogP values ranged from 2.80 to 3.38, and the TPSA values ranged from 79.9 to 96.03 Å^2^. Compounds **4i** and **4m** showed no Lipinski or Veber violations, whereas **4a** displayed one Lipinski violation due to its molecular weight (518.61 g/mol). All three compounds were predicted to have high gastrointestinal absorption and no blood–brain barrier permeation.

No mutagenic, tumorigenic, reproductive, or irritant liabilities were predicted for the analyzed compounds. Compounds **4m** and **4i** generated no PAINS alerts, whereas **4a** matched the anil_di_alk_C(246) structural filter, which is associated with its substituted arylamine motif. This alert should be regarded as a potential assay interference liability requiring experimental verification and does not, by itself, demonstrate nonspecific biological activity. Overall, the predicted physicochemical and liability profiles further support the prioritization of **4m** as the most balanced compound in the investigated subset.

## 3. Materials and Methods

### 3.1. General Information

All chemicals required for the synthesis were purchased from Merck KGaA (Darmstadt, Germany) and Acros Organics BV, Thermo Fisher Scientific (Geel, Belgium). All reactions were monitored by thin-layer chromatography (TLC) using silica gel 60 F_254_ pre-coated on aluminum plates (Merck, Germany). Column chromatography (CC) using silica gel 70–230 mesh (Merck KGaA, Darmstadt, Germany) was used for purification. The melting points were measured using a Stuart SMP2 instrument and were not corrected. ^1^H, ^13^C, DEPT135, and, where applicable, ^19^F NMR spectra were recorded on a Bruker 400 MHz-Avance III spectrometer (Bruker BioSpin GmbH, Rheinstetten, Germany) using DMSO-d_6_ or CDCl_3_ as solvents. ^13^C and ^19^F NMR spectra were acquired at 100 and 377 MHz, respectively. For the fluorinated derivatives, resolved ^13^C–^19^F coupling constants were included in the ^13^C NMR characterization. ^19^F NMR spectra were additionally recorded for compounds **4b** and **4c** during the revision. High-resolution mass spectra (HRMS) were recorded using a HRMS Bruker Daltonics 7.0T US+ (Bruker Daltonics GmbH & Co. KG, Bremen, Germany).

### 3.2. General Procedure for Synthesis of Compounds ***3a***–***p***

A solution of 2- or 4-hydroxybenzaldehyde **1** (300 mg, 2.45 mmol, 1.0 equiv.) and K_2_CO_3_ (1 g, 7.35 mmol, 3.0 equiv.) was prepared in acetone (10 mL) at room temperature. Propargyl bromide (557 μL, 7.35 mmol, 3.0 equiv.) was added dropwise, and the mixture was then refluxed for 4 h. Upon cooling to ambient temperature, the suspension was filtered and concentrated under reduced pressure, yielding products **2a**,**b** as yellow solids (390 mg, quantitative yield). The solid was then rinsed with acetone (2 × 20 mL) and not further purified. Compounds **2a** and **2b** (300 mg, 1.872 mmol, 1.0 equiv.) were combined with the corresponding secondary amine (2.246 mmol, 1.2 equiv.), aqueous formaldehyde (35%, 1 mL), and CuI (20 mg) in DMSO (2 mL) and stirred at ambient temperature for 3 h, following the copper-catalyzed Mannich-type (A3-type) coupling of terminal alkynes [25,35]. Upon completion of the reaction, as confirmed by TLC, the mixture was quenched by the addition of 10 mL of water and subsequently extracted with ethyl acetate (EtOAc, 2 × 20 mL). The organic layer was dried over anhydrous sodium sulfate (Na_2_SO_4_), filtered, and concentrated under reduced pressure using a rotary evaporator to obtain a crude product. The residue was purified by column chromatography using EtOAc/hexane (10:1) as the eluent.

### 3.3. General Procedure for Synthesis of Compounds ***4a***–***p***

A mixture of the appropriate aryl aldehyde (9.42 mmol, 1.0 equiv.), ethyl acetoacetate (9.42 mmol, 1.0 equiv.), urea (11.77 mmol, 1.25 equiv.), and *p*-toluenesulfonic acid (1.45 mmol, 0.15 equiv.) was heated at 90 °C for 24 h, following the Biginelli condensation [26,36]. After completion of the reaction, as monitored by TLC, the mixture was cooled to room temperature, and the residue was purified by column chromatography using EtOAc/hexane (1:9, *v*/*v*) to obtain compounds **4a**–**p**.

Ethyl 4-(4-((4-(4-(4-methoxyphenyl)piperazin-1-yl)but-2-yn-1-yl)oxy)phenyl)-6-methyl-2-oxo-1,2,3,4-tetrahydropyrimidine-5-carboxylate (**4a**)

Brown oil (150 mg, 53% yield, over two steps). ^1^H NMR (400 MHz, CDCl_3_) δ 8.19 (s, 1H, NH), 7.25 (d, J = 8.5 Hz, 2H, CH), 6.94–6.90 (m, J = 6.92 Hz, 4H, CH), 6.86 (d, J = 9.0 Hz, 2H, CH), 5.95 (s, 1H, NH), 5.33 (d, J = 13.2 Hz, 1H, CH), 4.72 (s, 2H, OCH_2_), 4.12 (q, 2H, CH_2_), 3.78 (s, 3H, OCH_3_), 3.42 (s, 2H, CH_2_), 3.14 (t, 4H, CH_2_N), 2.74 (t, 4H, CH_2_N), 2.34 (s, 3H, CH_3_), 1.18 (t, J = 7.1 Hz, 3H, CH_3_). ^13^C NMR (100 MHz, CDCl_3_) δ 165.7 (COO), 157.3 (OCH_2_), 154.0 (C-O), 153.4 (NHCONH), 146.0 (alkene), 145.49 (C-N), 136.9 (C), 127.8 (Ar), 118.4 (Ar), 115.0 (Ar), 114.5 (Ar), 101.6 (alkene), 82.4, 80.3 (alkyne), 60.0, 56.2 (CH_2_O), 55.6, 55.1, 52.1 (CHNH), 50.5, 47.1, 18.6 (CH_3_), 14.2 (CH_3_). HRMS: *m*/*z* [M+H]^+^ calcd for C_29_H_35_N_4_O_5_: 519.2607; found: 519.2581.

Ethyl 4-(4-((4-(4-(4-fluorophenyl)piperazin-1-yl)but-2-yn-1-yl)oxy)phenyl)-6-methyl-2-oxo-1,2,3,4-tetrahydropyrimidine-5-carboxylate (**4b**)

Pale yellow oil (550 mg, 55% yield, over two steps). ^1^H NMR (400 MHz, DMSO-d_6_) δ 9.16 (s, 1H, NH), 7.68 (s, 1H, NH), 7.16 (d, J = 8.5 Hz, 2H, CH), 7.07–7.02 (m, 2H, CH), 6.97–6.93 (m, 4H, CH), 5.11 (d, J = 2.6 Hz, 1H, CH), 4.82 (s, 2H, OCH_2_), 4.09 (q, J = 7.1 Hz, 2H, CH_2_), 3.37 (s, 2H, CH_2_), 3.07 (m, 4H, CH_2_N), 2.57 (m, 4H, CH_2_N), 2.24 (s, 3H, CH_3_), 1.10 (t, J = 7.1 Hz, 3H, CH_3_). ^13^C NMR (100 MHz, DMSO-d_6_) δ 165.8 (COO), 157.0, 156.5 (d, ^1^J = 230 Hz, C-F), 152.6 (C-O), 148.6 (C=O), 148.3 (alkene), 138.2 (C-N), 127.8 (Ar), 117.65 (d, ^3^J = 8 Hz, C-F), 115.7 (d, ^2^J = 21 Hz, C-F), 115.1 (Ar), 100.0 (alkene), 83.1, 80.9 (alkyne), 59.6 (CH_2_), 56.1 (OCH_2_), 53.8 (CH), 51.6 (CHNH), 49.3 (CH_2_N), 46.6 (CH_2_N), 18.2 (CH_3_), 14.6 (CH_3_). ^19^F NMR (377 MHz, DMSO-d_6_) δ −125.60. HRMS: *m*/*z* [M+H]^+^ calcd for C_28_H_32_FN_4_O_4_: 507.2408; found: 507.2400.

Ethyl 4-(4-((4-(4-(2-fluorophenyl)piperazin-1-yl)but-2-yn-1-yl)oxy)phenyl)-6-methyl-2-oxo-1,2,3,4-tetrahydropyrimidine-5-carboxylate (**4c**)

Beige solid (120 mg, 27% yield over two steps). m.p. 161–162 °C. ^1^H NMR (400 MHz, DMSO-d_6_) δ 9.16 (s, 1H, NH), 7.67 (s, 1H, NH), 7.16 (d, J = 8.7 Hz, 2H, CH), 7.13–6.97 (m, 4H, CH), 6.94 (d, J = 8.7 Hz, 2H, CH), 5.11 (d, J = 3.1 Hz, 1H, CH), 4.82 (s, 2H, OCH_2_), 4.09 (q, J = 7.1 Hz, 2H, CH_2_), 3.38 (s, 2H, CH_2_), 3.01 (m, 4H, CH_2_N), 2.60 (m, 4H, CH_2_N), 2.24 (s, 3H, CH_3_), 1.09 (t, J = 7.1 Hz, 3H, CH_3_). ^13^C NMR (100 MHz, DMSO-d_6_) δ 165.8 (COO), 157.0, 155.4 (d, ^1^J = 243 Hz, C-F), 152.6 (C-O), 148.6 (C=O), 140.25 (d, ^2^J = 8 Hz, C-F), 138.2 (C-N), 127.8 (Ar), 125.25 (d, ^4^J = 4 Hz, C-F), 122.75 (d, ^3^J = 7 Hz, C-F), 119.74 (d, ^4^J = 3 Hz, C-F), 116.38 (d, ^2^J = 21 Hz, C-F), 115.1 (Ar), 99.9 (alkene), 83.1, 80.9 (alkyne), 59.6 (CH_2_), 56.1 (OCH_2_), 53.8 (CHNH), 51.7 (CH_2_), 50.4 (CH_2_N), 46.7 (CH_2_N), 18.2 (CH_3_), 14.6 (CH_3_). ^19^F NMR (377 MHz, DMSO-d_6_) δ −122.77. HRMS: *m*/*z* [M+H]^+^ calcd for C_28_H_32_FN_4_O_4_: 507.2408; found: 507.2385.

Ethyl 6-methyl-2-oxo-4-(4-((4-(4-(pyridin-2-yl)piperazin-1-yl)but-2-yn-1-yl)oxy)phenyl)-1,2,3,4-tetrahydropyrimidine-5-carboxylate (**4d**)

Yellow oil (370 mg, 65% yield, over two steps). ^1^H NMR (400 MHz, DMSO-d_6_) δ 9.16 (s, 1H, NH), 8.11 (s, 1H, NH), 7.68 (m, 1H, CH), 7.52 (t, J = 7.7 Hz, 1H, CH), 7.15 (d, J = 8.8 Hz, 2H, CH), 6.92 (d, J = 8.8 Hz, 2H, CH), 6.82 (m, 1H, CH), 6.63 (m, 1H, CH), 5.1 (s, 1H, CH), 4.80 (s, 2H, OCH_2_), 3.98 (q, J = 6.4 Hz, 2H, CH_2_), 3.47 (t, 4H, CH_2_N), 3.37 (s, 2H, CH_2_), 2.51 (t, 4H, CH_2_N), 2.24 (s, 3H, CH_3_), 1.10 (t, J = 6.4 Hz, 3H, CH_3_). ^13^C NMR (100 MHz, CDCl_3_) δ 165.8 (COO), 159.5 (COCH_2_), 156.9 (C=O), 152.6 (alkene), 148.6, 148.0, 138.2, 138.0 (C-N), 127.8 (Ar), 115.1 (Ar), 113.5, 107.6, 100.0 (alkene), 83.1, 80.8 (alkyne), 59.7, 56.0 (CH_2_O), 53.8, 51.5 (CHNH), 46.7, 44.9, 18.2 (CH_3_), 14.6 (CH_3_). HRMS: *m*/*z* [M+H]^+^ calcd for C_27_H_32_N_5_O_4_: 490.2454; found: 490.2442.

Ethyl 6-methyl-4-(4-((4-(4-methylpiperazin-1-yl)but-2-yn-1-yl)oxy)phenyl)-2-oxo-1,2,3,4-tetrahydropyrimidine-5-carboxylate (**4e**)

Brown solid (370 mg, 65% yield, over two steps). m.p. 64–65 °C. ^1^H NMR (400 MHz, CDCl_3_) δ 8.30 (s, 1H, NH), 7.24 (d, J = 8.5 Hz, 2H, CH), 6.93 (d, J = 8.5 Hz, 2H, CH), 6.23 (s, 1H, NH), 5.35 (s, 1H, CH), 4.70 (s, 2H, OCH_2_), 4.10 (q, J = 7.1 Hz, 2H, CH_2_), 3.33 (s, 2H, NCH_2_), 2.63–2.57 (m, 8H, CH_2_), 2.25 (s, 3H, CH_3_), 2.16 (s, 3H, CH_3_), 1.11 (t, J = 7.1 Hz, 3H, CH_3_). ^13^C NMR (100 MHz, CDCl_3_) δ 165.9 (COO), 157.3 (C-O), 153.4 (CONH), 146.3 (alkene), 137.1, 127.8, 115.2 (Ar), 101.4 (alkene), 82.6, 80.1 (alkyne), 60.0 (*CH_2_*CH_3_), 56.2 (CH), 55.1 (OCH_2_), 54.7 (CH_2_N), 51.4 (CH_2_N), 47.0 (CH_2_N), 45.7 (CH_3_), 18.6 (CH_3_), 14.2 (CH_3_). HRMS: *m*/*z* [M+H]^+^ calcd for C_23_H_31_N_4_O_4_: 427.2345; found: 427.2350.

Ethyl 4-(4-((4-(4-(2-(isopropylamino)-2-oxoethyl)piperazin-1-yl)but-2-yn-1-yl)oxy)phenyl)-6-methyl-2-oxo-1,2,3,4-tetrahydropyrimidine-5-carboxylate (**4f**)

Brown oil (160 mg, 55% yield, over two steps). ^1^H NMR (400 MHz, CDCl_3_) δ 8.38 (s, 1H, NH), 7.25 (d, J = 8.5 Hz, 2H, CH), 6.90 (d, J = 8.5 Hz, 2H, CH), 6.04 (s, 1H, NH), 5.36 (s, 1H, CH), 5.31 (s, 1H, NH), 4.71 (s, 2H, OCH_2_), 4.14–4.04 (m, 3H, CH_2_ and CH), 3.34 (s, 2H, CH_2_N), 2.98 (s, 2H, NCH_2_CO), 2.57 (s, 8H, CH_2_N), 2.34 (s, 3H, CH_3_), 1.27–1.17 (m, 9H, CH_3_). ^13^C NMR (100 MHz, DMSO-d_6_) δ 169.2 (CONH), 165.8 (COO), 157.3 (C-O), 153.4 (C=O), 146.3 (alkene), 137.0, 127.8, 115.0 (Ar), 101.5 (alkene), 82.5, 80.0 (alkyne), 61.4 (*CH_2_*CH_3_), 60.1 (NCH_2_CO), 56.2 (OCH_2_), 54.9 (CHNH), 53.2, 52.0 (piperazine), 47.2 (CH_2_N), 40.8 (CH), 22.8 (CH_3_), 18.8 (CH_3_), 14.2 (CH_2_CH_3_). HRMS: *m*/*z* [M+H]^+^ calcd for C_27_H_38_N_5_O_5_: 512.2873; found: 512.2898.

Ethyl 4-(4-((4-(benzyl(methyl)amino)but-2-yn-1-yl)oxy)phenyl)-6-methyl-2-oxo-1,2,3,4-tetrahydropyrimidine-5-carboxylate (**4g**)

Pale yellow oil (141 mg, 24% yield, over two steps). ^1^H NMR (400 MHz, CDCl_3_) δ 8.27 (s, 1H, NH), 7.33–7.25 (m, 7H, CH), 6.94 (d, J = 8.2 Hz, 2H, CH), 5.96 (s, 1H, NH), 5.35 (s, 1H, CH), 4.75 (s, 2H, CH_2_), 4.06 (q, J = 7.1 Hz, 2H, CH_2_), 3.53 (s, 2H, CH_2_), 3.34 (s, 2H, CH_2_), 2.32 (s, 6H, 2CH_3_), 1.16 (t, J = 7.1 Hz, 3H, CH_3_). ^13^C NMR (100 MHz, CDCl_3_) δ 165.7 (COO), 157.3 (COCH_2_), 153.6 (C=O), 146.2 (alkene), 138.0 (C-N), 137.0 (C-CH), 129.2 (Ar), 128.4 (Ar), 128.0 (Ar), 127.3 (Ar), 115.0 (Ar), 101.6 (alkene), 82.5, 80.3 (alkyne), 60.1, 60.0, 56.2 (CH_2_O), 55.1, (CHNH), 45.2, 41.9, 18.5 (CH_3_), 14.3 (CH_3_). HRMS: *m*/*z* [M+H]^+^ calcd for C_26_H_30_N_3_O_4_: 448.2236; found: 448.2215.

Ethyl 6-methyl-4-(4-((4-morpholinobut-2-yn-1-yl)oxy)phenyl)-2-oxo-1,2,3,4-tetrahydropyrimidine-5-carboxylate (**4h**)

Yellow solid (130 mg, 31% yield over two steps). m.p. 155–156 °C. ^1^H NMR (400 MHz, DMSO-d_6_) δ 9.16 (s, 1H, NH), 7.67 (s, 1H, NH), 7.16 (d, J = 8.5 Hz, 2H, CH), 6.93 (d, J = 8.5 Hz, 2H, CH), 5.10 (d, J = 2.7 Hz, 1H, CH), 4.81 (s, 2H, OCH_2_), 3.99 (q, J = 7.1 Hz, 2H, CH_2_), 3.55 (t, 4H, CH_2_O), 3.30 (s, 2H, CH_2_N), 2.39 (s, 4H, CH_2_N), 2.25 (s, 3H, CH_3_), 1.10 (t, J = 7.1 Hz, 3H, CH_3_). ^13^C NMR (100 MHz, DMSO-d_6_) δ 165.8 (COO), 156.9 (C-O), 152.6 (CONH), 148.6 (alkene), 138.2, 127.9, 115.2 (Ar), 100.0 (alkene), 83.0, 80.9 (alkyne), 66.5 (CH_2_O), 59.6 (CH_2_CH_3_), 56.1 (OCH_2_), 53.8 (CH), 52.1 (CH_2_NCH_2_), 46.9 (CH_2_N), 18.2 (CH_3_), 14.6 (CH_3_). HRMS: *m*/*z* [M+H]^+^ calcd for C_22_H_28_N_3_O_5_: 414.2029; found: 414.2002.

Ethyl 6-methyl-2-oxo-4-(4-((4-(piperidin-1-yl)but-2-yn-1-yl)oxy)phenyl)-1,2,3,4-tetrahydropyrimidine-5-carboxylate (**4i**)

Pale yellow oil (161 mg, 24% yield over two steps). ^1^H NMR (400 MHz, DMSO-d_6_) δ 9.16 (s, 1H, NH), 7.67 (s, 1H, NH), 7.16 (d, J = 8.6 Hz, 2H, CH), 6.93 (d, J = 8.6 Hz, 2H, CH), 5.10 (s, 1H, CH), 4.79 (s, 2H, CH_2_N), 3.99 (q, J = 7.2 Hz, 2H, CH_2_), 3.25 (s, 2H, CH_2_), 2.34 (t, 4H, CH_2_), 2.24 (s, 3H, CH_3_), 1.45–1.47 (m, 4H, CH_2_), 1.32 (s, 2H, CH_2_), 1.11 (t, J = 7.2 Hz, 3H, CH_3_). ^13^C NMR (100 MHz, DMSO-d_6_) δ 166.0 (COO), 156.9 (COCH_2_), 152.6 (C=O), 148.7 (alkene), 138.2 (C-N), 127.7 (Ar), 115.3 (Ar), 100.0 (alkene), 83.6, 80.5 (alkyne), 59.6 (*CH_2_*CH_3_), 56.1 (CH_2_O), 53.8 (CHNH), 52.9 (CH_2_), 47.4 (CH_2_N), 44.8, 25.8 (CH_2_), 18.2 (CH_3_), 14.6 (CH_3_). HRMS: *m*/*z* [M+H]^+^ calcd for C_23_H_30_N_3_O_4_: 412.2236; found: 412.2225.

Ethyl 4-(4-((4-(dicyclohexylamino)but-2-yn-1-yl)oxy)phenyl)-6-methyl-2-oxo-1,2,3,4-tetrahydropyrimidine-5-carboxylate (**4j**)

Orange oil (262 mg, 35% yield over two steps). ^1^H NMR (400 MHz, DMSO-d_6_) δ 9.15 (s, 1H, NH), 7.67 (s, 1H, NH), 7.16 (d, J = 8.4 Hz, 2H, CH), 6.92 (d, J = 8.4 Hz, 2H, CH), 5.10 (d, J = 2.4 Hz, 1H, CH), 4.74 (s, 2H, OCH_2_), 3.99 (q, J = 7.1 Hz, 2H, *CH_2_*CH_3_), 3.42 (s, 2H, NCH_2_), 2.63–2.58 (m, 2H, CH), 2.23 (s, 3H, CH_3_), 1.65–1.63 (m, 8H, CH_2_), 1.24–1.00 (m, 15H, CH_3_ and CH_2_). ^13^C NMR (100 MHz, DMSO-d_6_) δ 165.8 (COO), 157.0 (C-O), 152.7 (CONH), 148.6 (alkene), 138.0, 127.7, 115.1 (Ar), 100.0 (alkene), 87.8, 78.4 (alkyne), 59.6 (*CH_2_*CH_3_), 57.2 (CH), 56.1 (OCH_2_), 53.7 (CH), 31.4 (CH_2_CHCH_2_), 26.2 (CH_2_), 26.2, 26.0 (CH_2_), 18.2 (CH_3_), 14.6 (CH_3_). HRMS: *m*/*z* [M+H]^+^ calcd for C_30_H_42_N_3_O_4_: 508.3175; found: 508.3149.

Ethyl 4-(2-((4-(4-(4-methoxyphenyl)piperazin-1-yl)but-2-yn-1-yl)oxy)phenyl)-6-methyl-2-oxo-1,2,3,4-tetrahydropyrimidine-5-carboxylate (**4k**)

Brown oil (787 mg, 78% yield over two steps). ^1^H NMR (400 MHz, DMSO-d_6_) δ 9.12 (s, 1H, NH), 7.25–7.22 (m, 2H, CH), 7.12–7.08 (m, 2H, CH), 6.94–6.84 (m, 2H, CH), 6.83–6.80 (m, 2H, CH), 5.52 (d, J = 2.1 Hz, 1H, CH), 4.91 (m, 2H, CH_2_), 3.92 (q, J = 7.0 Hz, 2H, *CH_2_*CH_3_), 3.68 (s, 3H, OCH_3_), 3.38 (s, 2H, CH_2_), 3.00 (t, 4H, CH_2_N), 2.58 (t, 4H, CH_2_N), 2.29 (s, 3H, CH_3_), 1.04 (t, J = 7.0 Hz, 3H, CH_3_). ^13^C NMR (100 MHz, DMSO-d_6_) δ 165.9 (COO), 155.2 (C-O),153.39 (C-O), 152.7 (CONH), 149.5 (alkene), 145.9, 132.8, 128.9, 127.9, 121.4, 117.9, 114.7, 113.3 (Ar), 98.0 (alkene), 83.3, 81.1 (alkyne), 59.5 (*CH_2_*CH_3_), 56.6 (OCH_2_), 55.6 (OCH_3_), 51.8 (CH_2_N), 50.0 (CH_2_N), 49.4 (CH), 46.7 (CH_2_N), 18.2 (CH_3_), 14.5 (CH_3_). HRMS: *m*/*z* [M+H]^+^ calcd for C_29_H_35_N_4_O_5_: 519.2607; found: 519.2601.

Ethyl 4-(2-((4-(4-(4-fluorophenyl)piperazin-1-yl)but-2-yn-1-yl)oxy)phenyl)-6-methyl-2-oxo-1,2,3,4-tetrahydropyrimidine-5-carboxylate (**4l**)

Pale yellow solid (300 mg, 66% yield, over two steps). ^1^H NMR (400 MHz, DMSO-d_6_) δ 9.16 (s, 1H, NH), 7.68 (s, 1H, NH), 7.16 (d, J = 8.4 Hz, 2H, CH), 7.07–7.02 (d, J = 8.4 Hz, 2H, CH), 6.97–6.93 (m, 4H, CH), 5.11 (d, J = 2.2 Hz, 1H, CH), 4.82 (s, 2H, OCH_2_), 3.99 (q, J = 7.1 Hz, 2H, *CH_2_*CH_3_), 3.37 (s, 2H, CH_2_N), 3.07 (t, 4H, CH_2_N), 2.57 (t, 4H, CH_2_N), 2.24 (s, 3H, CH_3_), 1.10 (t, J = 7.1 Hz, 3H, CH_3_). ^13^C NMR (100 MHz, DMSO-d_6_) δ 165.8 (COO), 157.0 (C-O), 156.6 (d, ^1^J = 230 Hz, C-F), 152.6 (CONH), 148.5 (alkene), 148.4 (C-N), 138.2, 127.8, 117.65 (d, ^3^J = 8 Hz, C-F), 115.7 (d, ^2^J = 21 Hz, C-F), 115.1 (Ar), 99.9 (alkene), 83.1, 80.9 (alkyne), 59.6 (*CH_2_*CH_3_), 56.1 (OCH_2_), 53.8 (CH_2_N), 51.7 (CH_2_N), 49.3 (CH), 46.6 (CH_2_N), 18.2 (CH_3_), 14.6 (CH_3_). HRMS: *m*/*z* [M+H]^+^ calcd for C_28_H_32_FN_4_O_4_: 507.2408; found: 507.2406.

Ethyl 6-methyl-2-oxo-4-(2-((4-(4-(pyridin-2-yl)piperazin-1-yl)but-2-yn-1-yl)oxy)phenyl)-1,2,3,4-tetrahydropyrimidine-5-carboxylate (**4m**)

Brown oil (401 mg, 38% yield, over two steps). ^1^H NMR (400 MHz, DMSO-d_6_) δ 9.11 (s, 1H, NH), 8.10 (d, J = 4.4 Hz, 1H, NH), 7.52 (m, 1H, CH), 7.23–7.17 (m, 2H, CH), 7.09–7.07 (m, 2H, CH), 6.91–6.88 (m, 1H, CH), 6.88–6.80 (m, 1H, CH), 6.65–6.62 (m, 1H, CH), 5.50 (d, J = 4.4 Hz, 1H, CH), 4.85 (m, 2H, CH_2_), 3.92 (q, J = 7.1 Hz, 2H, *CH_2_*CH_3_), 3.47 (t, 4H, CH_2_N), 3.39 (s, 2H, OCH_2_), 2.51 (m, 4H, CH_2_N), 2.28 (s, 3H, CH_3_), 1.03 (t, J = 7.1 Hz, 3H, CH_3_). ^13^C NMR (100 MHz, DMSO-d_6_) δ 165.8 (COO), 159.4 (C-N), 155.1 (C-O), 152.5 (C=O), 149.4 (alkene), 148.0, 137.9, 132.8, 128.9, 127.8, 121.4, 113.4, 113.3, 107.52, (Ar), 98.0 (alkene), 83.2, 81.1 (alkyne), 59.4 (*CH_2_*CH_3_), 56.6 (CH_2_O), 51.5 (CH_2_N), 49.3 (CH), 46.8 (CH_2_N), 44.9 (CH_2_N), 18.2 (CH_3_), 14.5 (CH_3_). HRMS: *m*/*z* [M+H]^+^ calcd for C_27_H_32_N_5_O_4_: 490.2454; found: 490.2451.

Ethyl 4-(2-((4-(4-(2-(isopropylamino)-2-oxoethyl)piperazin-1-yl)but-2-yn-1-yl)oxy)phenyl)-6-methyl-2-oxo-1,2,3,4-tetrahydropyrimidine-5-carboxylate (**4n**)

Brown oil (160 mg, 48% yield, over two steps). ^1^H NMR (400 MHz, DMSO-d_6_) δ 9.11 (s, 1H, NH), 7.38 (m, 1H, CH), 7.22 (m, 2H, NH and CH), 7.08 (m, 2H, CH), 6.93–6.92 (m, 1H, CH), 5.50 (s, 1H, CH), 4.84 (q, J = 7.2 Hz, 2H, O*CH_2_*), 3.90 (m, 3H, CH and CH_2_), 3.31 (m, 2H, CH_2_), 2.85 (m, 2H, CH_2_N), 2.51 (m, 2H, CH_2_), 2.45 (m, 6H, CH_2_), 2.28 (s, 3H, CH_3_), 1.07–1.02 (m, 9H, CH_3_). ^13^C NMR (100 MHz, DMSO-d_6_) δ 168.4 (CONH), 166.0 (COO), 164.9, 155.2 (C=O), 152.7 (C-O), 149.6 (alkene), 132.6, 128.7, 127.9, 121.3, 113.2, (Ar), 97.8 (alkene), 83.7, 81.0 (alkyne), 61.7 (CH_2_CH_3_), 59.4 (OCH_2_CO), 56.7 (OCH_2_), 53.1 (CH_2_N), 51.7 (CH_2_N), 49.6 (CH), 46.8 (CH_2_N), 22.8 (CH_3_), 18.4 (CH_3_), 14.5 (CH_3_). HRMS: *m*/*z* [M+H]^+^ calcd for C_27_H_38_N_5_O_5_: 512.2873; found: 512.2862.

Ethyl 6-methyl-4-(2-((4-morpholinobut-2-yn-1-yl)oxy)phenyl)-2-oxo-1,2,3,4-tetrahydropyrimidine-5-carboxylate (**4o**)

Yellow solid (310 mg, 47% yield, over two steps). m.p. 99–100 °C. ^1^H NMR (400 MHz, DMSO-d_6_) δ 9.11 (s, 1H, NH), 7.23–7.22 (m, 1H, CH), 7.11–7.08 (m, 2H, CH), 6.94–6.90 (m, 1H, CH), 5.51 (s, 1H, CH), 4.87 (m, 2H, CH_2_), 3.92 (q, J = 7.1 Hz, 2H, *CH_2_*CH_3_), 3.57–3.55 (m, 4H, CH_2_O), 3.34 (s, 2H, CH_2_), 2.41 (m, 4H, CH_2_N), 2.28 (s, 3H, CH_3_), 1.04–1.02 (t, J = 7.1 Hz, 3H, CH_3_). ^13^C NMR (100 MHz, DMSO-d_6_) δ 165.8 (COO), 156.9 (C-O), 152.6 (CONH), 148.7 (alkene), 138.2, 127.8, 115.2 (Ar), 100.0 (alkene), 83.0, 80.9 (alkyne), 76.7, 66.5, 59.6 (*CH_2_*CH_3_), 56.1 (CH), 53.8 (OCH_2_), 52.1 (CH_2_N), 46.9 (CH_2_NCH_2_), 18.2 (CH_3_), 14.6 (CH_3_). HRMS: *m*/*z* [M+H]^+^ calcd for C_22_H_28_N_3_O_5_: 414.2029; found: 414.2003.

Ethyl 6-methyl-2-oxo-4-(2-((4-(piperidin-1-yl)but-2-yn-1-yl)oxy)phenyl)-1,2,3,4-tetrahydropyrimidine-5-carboxylate (**4p**)

Brown oil (514 mg, 64% yield, over two steps). ^1^H NMR (400 MHz, DMSO-d_6_) δ 8.64 (s, 1H, NH), 7.46–6.79 (m, 4H, CH), 5.15 (m, 1H, CH), 3.94 (q, J = 7.3 Hz, 2H, *CH_2_*CH_3_), 3.33 (s, 2H, OCH_2_), 3.26 (s, 2H, CH_2_), 2.55 (s, 4H, CH_2_N), 2.35 (s, 2H, CH_2_), 2.20 (s, 3H, CH_3_), 1.47–1.31 (s, 4H, CH_2_), 1.12 (t, J = 7.3 Hz, 3H, CH_2_*CH_3_*). ^13^C NMR (100 MHz, DMSO-d_6_) δ 167.6 (COO), 155.1 (C-O), 153.8 (CONH), 145.4 (alkene), 137.1, 130.6, 127.5, 121.0, 112.7 (Ar), 101.8 (alkene), 83.0, 80.9 (alkyne), 59.1 (*CH_2_*CH_3_), 56.1 (OCH_2_), 52.8 (CH_2_N), 47.5 (CH), 41.0, 34.5 (CH_2_N), 25.9 (CH_2_), 24.0, 18.5 (CH_3_), 14.6 (CH_3_). HRMS: *m*/*z* [M+H]^+^ calcd for C_23_H_30_N_3_O_4_: 412.2236; found: 412.2228.

### 3.4. Biological Assays

#### 3.4.1. Cell Cultures and Treatments

ER-positive (ER+) MCF-7 breast cancer cells were kindly donated by IRCCS Ospedale Policlinico San Martino (ICLC, Genova, Italy). NIH/3T3 murine fibroblasts were purchased from the American Type Culture Collection (Manassas, VA, USA).

NIH/3T3 and ER+ MCF-7 cells were maintained in Dulbecco’s Modified Eagle’s Medium (DMEM) supplemented with 10% fetal bovine serum (FBS), 100 U/mL penicillin/streptomycin, and 2 mM l-glutamine. Cells were cultured at 37 °C in a humidified incubator under an atmosphere of 5% CO_2_ and 95% air. Immediately before the experiments, the tested compounds were dissolved in dimethyl sulfoxide (DMSO) and diluted to the desired concentrations in the culture medium. The final DMSO concentration was always maintained at 0.1% (*v*/*v*) in all treatment groups. Cells treated with the vehicle alone (0.1% DMSO) were used as controls (CTR).

#### 3.4.2. Cell Toxicity and Antiproliferative Assays

Cellular biomass was assessed in NIH/3T3 fibroblasts and estrogen receptor-positive (ER+) MCF-7 breast cancer cells using the sulforhodamine B (SRB) assay, a colorimetric method based on quantification of total cellular protein content.

For the cytotoxicity assay, NIH/3T3 cells were seeded in 96-well plates at a density of 1.3 × 10^4^ cells/well and allowed to attach for 24 h. The cells were subsequently exposed to increasing concentrations of the tested compounds for 24 h, and the cellular biomass was determined using the SRB assay.

For the antiproliferative assay, MCF-7 cells were seeded in 24-well plates at a density of 3.0 × 10^4^ cells/well and incubated for 24 h to allow for cell attachment. The cells were then exposed to increasing concentrations of the selected compounds for six days, with the medium containing the compound renewed on day 3. The control cells received the corresponding concentration of vehicle alone (DMSO).

Following treatment, an SRB assay was performed according to a previously described protocol [37] based on the original method [38,39]. Briefly, the protein-bound dye was solubilized in 10 mM Tris-base solution, and absorbance was measured at 565 nm using a GloMax Discover System microplate reader TM397 (Promega Corporation, Madison, WI, USA).

For NIH/3T3 cells, the results were expressed as the concentration required to reduce the SRB signal by 50% relative to vehicle-treated controls, reported as the 50% cytotoxic concentration (CC_50_). For MCF-7 cells, the results were expressed as the concentration required to inhibit cell growth by 50% relative to the vehicle-treated controls and reported as the 50% inhibitory concentration (IC_50_). The CC_50_ and IC_50_ values, along with their 90% confidence limits (CL), were calculated using the Litchfield–Wilcoxon method [40]. All assays were performed in three independent biological experiments, each with three technical replicates per concentration.

NIH/3T3 fibroblasts were used as a first-tier basal-cytotoxicity comparator rather than as a tissue-matched normal breast model. The preliminary selectivity index (SI) was calculated as the ratio of the NIH/3T3 CC_50_ and the MCF-7 IC_50_. Because the two endpoints were obtained in different cell types and using different exposure times, these SI values were used only for preliminary compound prioritization and should not be interpreted as definitive measures of tumor selectivity.

#### 3.4.3. Colony-Formation Assay Under Continuous Exposure

The effect of the selected compounds on the colony-forming ability of MCF-7 breast cancer cells was evaluated using a colony formation assay under continuous exposure [41], adapted from the general principles of the clonogenic assay described by Franken et al. [42].

MCF-7 cells were seeded in 6-well plates at a density of 1.2 × 10^4^ cells/well and allowed to attach for 24 h. The cells were subsequently exposed to each selected compound at the corresponding MCF-7 IC_25_ concentration for six days. On day 3, the culture medium was replaced with a fresh medium containing the same concentration of the tested compound. Vehicle control cells were exposed to the corresponding concentration of DMSO alone and received fresh vehicle-containing medium on day 3.

At the end of the six-day exposure period, the medium was removed, and the cells were gently washed with DPBS. The colonies were fixed with 4% paraformaldehyde for 15 min and stained with 0.2% crystal violet solution. After removing the excess stain, the plates were washed, air-dried, and imaged under standardized acquisition conditions using an inverted microscope.

Colony numbers were quantified from the acquired images using ImageJ software (v. 1.54t, https://imagej.net/ij/, accessed on 14 September 2026), and the same image processing and counting parameters were applied to all experimental groups. The percentage of inhibition of colony formation was calculated relative to the vehicle-treated control using the following equation:

$$\mathrm{Colony}\text{-}\mathrm{formation}\;\mathrm{inhibition}\;(\%)=\left(1-\frac{N_{treated}}{N_{vehicle}}\right)\times 100$$

where *N*_treated_ and *N*_vehicle_ represent the mean number of colonies counted in the compound- and vehicle-treated wells.

Data are expressed as the mean percentage of colony formation inhibition ± standard deviation (SD) from three independent biological experiments, each performed in six replicates.

Because the compounds remained present throughout the six-day assay and no post-treatment drug-free recovery phase was included, this endpoint measured the inhibition of colony formation under continuous exposure and should not be interpreted as post-treatment clonogenic persistence.

### 3.5. Computational Methods

#### 3.5.1. Proteins and Ligands Preparation

The protein models were prepared as described previously [43]. Ligand preparation was performed using Open Babel (v. 3.1.1) [44], where 3D structures were generated from SMILES codes and minimized at physiological pH (7.4).

#### 3.5.2. Protein Models Validation

The PDB IDs used were 6ZY5 (human TOP2A DNA-binding/cleavage domain in complex with DNA and etoposide) for TOP2A, 6G6Y for Eg5/KIF11, 1SC7 for DNA topoisomerase I, 4AF3 for Aurora kinase B, 2V5X for HDAC8, 7U3M for HDAC10, and 8EXL for PI3Kα, respectively. Each structure was selected as a ligand-bound complex that allowed validation by redocking. The resolution and co-crystallized ligand of each entry are listed in Table S2. Because no crystal structure of human HDAC10 is available in the PDB, the HDAC10 entry (7U3M) was derived from *Danio rerio* HDAC10, which shares 47% sequence identity with the human enzyme. HDAC8 and HDAC10, which are Zn^2+^-dependent metalloenzymes, were docked using a zinc-aware protocol previously validated for this enzyme class [45]. All protein models were selected based on the low RMSD values obtained during the redocking of the co-crystallized ligand, with a value below 3 Å, which is a commonly applied acceptance threshold [46]. The RMSD values obtained were 2.12 Å for TOP2A, 0.23 Å for Eg5/KIF11, 1.14 Å for DNA topoisomerase I, 0.99 Å for Aurora kinase B, 2.64 Å for HDAC8, 1.10 Å for HDAC10, and 1.10 Å for PI3Kα. The RMSD values were computed using the obrms utility of Open Babel (v. 3.1.1) [44]. Both the reference co-crystallized ligand and generated docking poses were supplied as Protein Data Bank (PDB) files.

#### 3.5.3. Molecular Docking Analysis

The docking protocol used here is identical to that validated in our previous work [43]. The grid parameters were chosen to be sufficiently wide to avoid forcing the ligand–receptor interactions. The grid parameters used for docking all ligands were as follows: center x = 179.513, center y = 179.326, center z = 164.226, npts x = 40, npts y = 40, npts z = 28, and spacing = 1 for TOP2A; center x = 10.835, center y = 14.654, center z = 1.327, npts x = 18, npts y = 20, npts z = 16, and spacing = 1 for Eg5/KIF11; center x = 94.124, center y = −0.605, center z = 6.424, npts x = 28, npts y = 24, npts z = 28, and spacing = 1 for DNA topoisomerase I; center x = 24.150, center y = −21.445, center z = −10.239, npts x = 23, npts y = 26, npts z = 28, and spacing = 1 for Aurora kinase B; center x = 16.167, center y = 5.792, center z = 50.431, npts x = 25, npts y = 28, npts z = 35, and spacing = 1 for HDAC8; center x = −26.736, center y = −40.309, center z = 35.464, npts x = 25, npts y = 24, npts z = 24, and spacing = 1 for HDAC10; and center x = 17.499, center y = 63.448, center z = 23.864, npts x = 29, npts y = 40, npts z = 34, and spacing = 1 for PI3Kα.

#### 3.5.4. Eg5 Homology Model

The crystallographic structure of Eg5/KIF11 contained unresolved regions that could affect the subsequent analysis of protein dynamics. A complete model was therefore generated before the MD simulations. The human Eg5 sequence was obtained from UniProt (P52732) in FASTA format and processed using the automated homology-modeling tools implemented in YASARA [47]. The available experimental Eg5 structure was used as the main structural template, thereby preserving the experimentally determined architecture of the ligand-binding region, while residues absent from the crystal structure were modeled from sequence and structural homology. This procedure produced a full-length hybrid Eg5 model in which the resolved crystallographic core was retained, and the missing segments were reconstructed. The final model showed a YASARA Z-score of −1.218. Reconstruction of the unresolved regions, optimization of their geometry, and energy minimization were performed within the YASARA modeling workflow prior to use of the model in the MD simulations.

#### 3.5.5. Molecular Dynamics Simulations

MD simulations were performed as described in our previous studies [43,48]. MD simulations were performed for 200 ns. After the MD simulation, the root mean square deviation (RMSD), radius of gyration (R_g_), principal component analysis (PCA), and internal energy trends over time were evaluated using the md_analyze macro. To obtain comparative binding energy estimates for the simulated complexes, MM/PBSA analysis was performed over 200 ns trajectories using the md_analyzebindenergy macro. According to the YASARA energy convention used here, larger positive values correspond to more favorable calculated binding energies within the same receptor system.

#### 3.5.6. ADMET Predictions

Molecular weight, logP, TPSA, mutagenic, tumorigenic, reproductive, irritant, and PAINS pattern parameters were calculated using DataWarrior (v. 06.05.04, OpenMolecules, Allschwil, Switzerland). Lipinski and Veber violations, GI absorption, and BBB permeability were calculated using the SwissADME web tool (https://www.swissadme.ch/, accessed on 3 September 2026).

#### 3.5.7. Intermolecular Interactions Analysis

Ligand–protein interactions were analyzed using the BIOVIA Discovery Studio Visualizer (v. 25.1.0.24284, Dassault Systèmes Biovia Corp., San Diego, CA, USA). The co-crystallized structure was uploaded in PDB format, and all hydrogen atoms were added using the ‘Chemistry → Hydrogen → Add’ option in the upper toolbar. The docked pose was selected and defined as the ligand. The interactions were analyzed using the ‘Show 2D Diagram’ option.

#### 3.5.8. Target Prioritization and Statistics

Ligand-based target prediction for the seven biologically characterized compounds was performed using SuperPred 3.0 [29]. The seven selected targets were then evaluated using GNINA [49]. Agreement between the predicted p*K*_d_ values and the experimental pIC_50_ values (*n* = 7) was assessed using Pearson and Spearman correlation coefficients. Two-sided *p*-values were computed from the same data, and a Bonferroni factor of seven was applied to account for the number of targets tested (Pearson *p* = 0.0084 and Spearman *p* = 0.0068 for TOP2A before correction).

## 4. Conclusions

In summary, a focused library of DHPM-based hybrids was prepared using a modular synthetic route and evaluated using a phenotypic anticancer screening workflow. Biological profiling revealed that the series contained compounds with distinct response patterns, revealing assay-dependent differences between bulk-cell antiproliferative activity and colony formation inhibition upon continuous exposure to the compounds. Among the tested derivatives, compound **4m** emerged as the most balanced lead, exhibiting favorable activity against MCF-7 cells, acceptable behavior in the preliminary NIH/3T3 assay, and the highest mean inhibition of colony formation.

Rather than supporting an overly broad target-based claim, the findings are best interpreted within the phenotypic medicinal chemistry framework. Within this framework, computational analyses serve as a rational target deconvolution strategy for prioritizing plausible protein systems capable of explaining the observed biological ranking and guiding future optimizations. Overall, the present study highlights **4m** as a promising starting point for developing DHPM-based antiproliferative agents.

The SuperPred-guided deconvolution and GNINA screening of seven candidate targets ranked TOP2A as the target whose predicted affinities best tracked the experimental pIC_50_ values (Pearson r = 0.883, Spearman ρ = 0.893, *n* = 7), with Eg5/KIF11 as a secondary comparator that highlights compound-specific differences within the active subset. The 200 ns MD simulations of four representative compounds were more convergent for TOP2A than for Eg5 and were compatible with this ranking. These analyses rest on correlations over seven compounds, on one trajectory per complex, and on predicted rather than measured binding, and they do not validate the target.

From a lead-prioritization perspective, **4m** exhibited the highest mean colony-formation inhibition (68.3% at its IC_25_), a favorable predicted developability profile, and computational behavior compatible with the TOP2A-centered hypothesis. In contrast, **4a**, although equally potent in the SRB assay, reduced colony formation by 25.3% and matched a PAINS structural alert, which warrants experimental follow-up. Compound **4m** was identified as the most balanced lead in the series, whereas TOP2A remains a prioritized mechanistic hypothesis requiring direct experimental validation.

Several limitations define the next steps. Target engagement should be tested directly, for example, with topoisomerase II relaxation or decatenation assays and detection of cleavage complexes in MCF-7 cells, together with a counter-screen against Eg5. Cell-cycle analysis would show whether the antiproliferative effect is accompanied by accumulation in a specific phase. Selectivity should be re-evaluated in a non-tumorigenic human breast epithelial line, such as MCF-10A, under exposure times matched to the MCF-7 assays. Because the compounds were obtained and tested as racemates, resolution and separate testing of the enantiomers of **4m** is also needed. Finally, the structure–activity trends should be extended with further ortho/para pairs and terminal amines, and the predicted developability profile should be confirmed experimentally.

## Figures and Tables

**Table 1:** Structures and isolated yields of compounds **4a**–**p** *^a^*.

Comp	R	Yield % *^b^*	Comp	R	Yield % *^b^*
**4a**	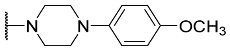	53	**4i**	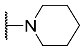	24
**4b**	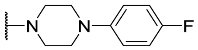	55	**4j**	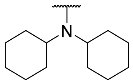	35
**4c**	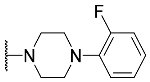	27	**4k**	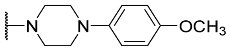	78
**4d**	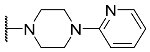	65	**4l**	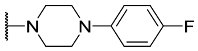	66
**4e**	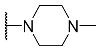	65	**4m**	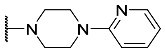	38
**4f**	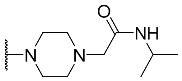	55	**4n**	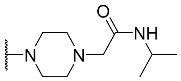	48
**4g**	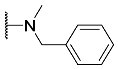	24	**4o**	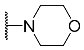	47
**4h**	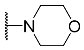	31	**4p**	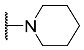	64

*^a^* Compounds **4a**–**j** have aryl groups in the *para*-position, and compounds **4k**–**p** have aryl groups in the *ortho*-position. *^b^* Over two steps.

**Table 2:** NIH/3T3 CC_50_ values of the screened compounds.

Compound	CC_50_ (µM)	90% CL Lower (µM)	90% CL Upper (µM)
**4a**	102.7	69.8	128.6
**4b**	34.7	24.4	49.2
**4c**	52.0	39.4	68.5
**4d**	45.3	36.4	56.3
**4e**	318.3	300.5	337.7
**4f**	330.3	321.6	357.7
**4g**	68.7	50.7	79.5
**4h**	272.2	214.5	345.5
**4i**	206.6	173.2	246.5
**4j**	16.8	11.2	25.3
**4k**	62.1	24.1	76.3
**4l**	52.7	42.3	65.7
**4m**	105.7	88.1	111.2
**4n**	62.5	50.3	77.7
**4o**	189.9	164.7	218.9
**4p**	67.2	29.4	82.3

**Table 3:** Antiproliferative IC_50_ values against MCF-7 cells and corresponding SIs *^a^*.

Compound	IC_50_ (µM)	90% CL Lower (µM)	90% CL Upper (µM)	SI
**4a**	10.7	8.8	13.1	9.6
**4e**	137.1	117.2	160.3	2.3
**4f**	115.8	67.0	199.9	2.9
**4h**	75.5	64.2	88.7	3.6
**4i**	43.6	37.9	50.2	4.7
**4m**	11.6	9.4	14.3	9.1
**4o**	151.3	118.3	193.7	1.3

*^a^* SI values were calculated as NIH/3T3 CC_50_/MCF-7 IC_50_. Because the two endpoints were obtained in different cell types and after different exposure times (24 h for NIH/3T3 and 6 days for MCF-7), these values should be regarded as preliminary indicators for compound prioritization rather than as definitive measures of tumor selectivity.

**Table 4:** Colony formation inhibition after six days of continuous exposure to compound-specific MCF-7 IC_25_ concentrations.

Compound	Concentration Tested (IC_25_, µM)	Colony-Formation Inhibition (%)	SD
**4a**	5.4	25.3	3.8
**4e**	79.6	53.6	5.4
**4f**	64.9	19.4	1.9
**4h**	43.0	52.3	10.5
**4i**	24.0	52.7	7.9
**4m**	6.9	68.3	17.1

**Table 5:** Preliminary target-screening results for the biologically characterized subset based on GNINA CNN affinity values, expressed as predicted affinity p*K*_d_, and their correlation with experimental pIC_50_ values (*n* = 7).

Comp.	pIC_50_	TOP2A	Eg5/KIF11	DNA Topoisomerase I	Aurora Kinase B	HDAC8	HDAC10	PI3Kα
**4a**	4.97	6.57	5.49	6.21	7.00	5.31	6.27	6.90
**4m**	4.94	6.29	5.81	6.46	7.12	5.35	6.31	6.61
**4i**	4.36	6.04	5.24	6.53	6.68	5.31	4.70	6.91
**4h**	4.12	5.77	5.46	5.68	6.58	5.25	5.41	6.84
**4f**	3.94	6.01	5.34	6.36	6.84	5.41	5.71	6.74
**4e**	3.86	5.88	5.08	6.07	6.69	5.20	5.41	6.62
**4o**	3.82	5.53	5.09	5.77	7.02	5.34	5.02	6.72
Pearson		0.883	0.806	0.500	0.503	0.170	0.679	0.220
Spearman		0.893	0.821	0.500	0.107	0.072	0.541	0.357

**Table 6:** Average MM/PBSA binding energies calculated over 200 ns molecular dynamics trajectories *^a^*.

Compound	Binding Energy Average (kcal/mol)
TOP2A	
**4a**	25.7
**4i**	46.7
**4m**	91.4
**4o**	45.1
Eg5	
**4a**	297.7
**4i**	−29.8
**4m**	287.3
**4o**	245.9

*^a^* Binding energy is derived from the potential and solvation energies of the isolated components minus those of the complex. According to the YASARA energy convention used here, higher positive values correspond to more favorable calculated binding energies within the same receptor system.

**Table 7:** Predicted physicochemical, drug-likeness, and ADMET profiles of **4a**, **4i**, and **4m**.

Parameter	4a	4i	4m
Physicochemical			
MW (g/mol)	518.612	411.5	489.574
LogP	3.3828	3.0122	2.8033
TPSA (Å^2^)	92.37	79.9	96.03
Lipinski Violations	1	0	0
Veber Violations	0	0	0
Pharmacokinetics			
GI Absorption	High	High	High
BBB Permeant	No	No	No
Toxicity/Liability			
Mutagenic	None	None	None
Tumorigenic	None	None	None
Reproductive Effects	None	None	None
Irritant	None	None	None
PAINS alerts	anil_di_alk_C(246)	None	None

## Data Availability

The raw data supporting the conclusions of this study will be made available by the authors upon reasonable request.

## Supplementary Materials

The following supporting information can be downloaded at: https://www.mdpi.com/article/10.3390/ijms27219618/s1.

## Abbreviations

The following abbreviations are used in this manuscript:

DHPMs	3,4-Dihydropyrimidin-2(1*H*)-ones
CC_50_	50% cytotoxic concentration
IC_50_	50% inhibitory concentration
CL	confidence limits
SI	selectivity index
SD	standard deviation
PDB	protein data bank
RMSD	root mean square deviation
R_g_	radius of gyration
PCA	principal component analysis
MD	molecular dynamics
MM/PBSA	molecular mechanics/Poisson–Boltzmann surface area
TOP2A	DNA topoisomerase IIα
Eg5/KIF11	kinesin spindle protein
SRB	sulforhodamine B
