# Supplementary material for: 3,4-Dihydropyrimidin-2(1H)-One Hybrids as Phenotypic Anti-Breast Cancer Agents: Synthesis, Colony-Formation Profiling and Computational Target Deconvolution
Source: Int J Mol Sci. 2026 Oct 27;27(21):9618. doi: 10.3390/ijms27219618 (PMC13763226; doi:10.3390/ijms27219618)
Supplement: Supplementary file 1 [file ijms-27-09618-s001.zip › ijms-4611220_SI_v10_revised.pdf]

Supplementary Materials

3,4-Dihydropyrimidin-2(1*H*)-one Hybrids as Phenotypic Anti-Breast Cancer Agents: Synthesis, Colony-Formation Profiling and Computational Target Deconvolution

Hadeel D. Sultan, Rocco Buccheri, Anas J. Rasras, Mohammad S. Alzubi *, Eyad A. Younes, Placido Mineo, Maria S. Molonia, Francesco Cimino, Antonio Rescifina *, Nicola Micale

Table of contents

Table S1. SuperPred 3.0 predictions for the six targets carried to the GNINA screening (probability, %). The SuperPred 3.0 export lists targets with a predicted probability of at least 50%. Eg5/KIF11 was not predicted for any compound.

Table S2. Protein structures used for docking and redocking validation.

Table S3. Intermolecular interactions between compounds **4a**, **4e**, **4f**, **4h**, **4i**, **4m**, and **4o** and the Eg5/KIF11 binding site.

Figure S1. Heatmap of the NIH/3T3 CC_{50} values and 90% confidence limits (CL) in Table 2. Colors indicate the CC_{50} value on a logarithmic scale (darker = less cytotoxic). Dots mark the compounds with $CC_{50} \geq 100$ μM that were retained for MCF-7 profiling.

Figure S2. Heatmap of the MCF-7 IC_{50} values with 90% CL and the selectivity indices (SI) in Table 3. Darker colors indicate higher potency (IC_{50}, logarithmic scale) and higher selectivity (SI).

Figure S3. Heatmap of the GNINA CNN affinity values (predicted pK_d) in Table 5 and of the Pearson and Spearman correlations with the experimental pIC_{50} (n = 7). The TOP2A column is outlined. Darker colors indicate higher values.

Figure S4. MD results for Eg5/KIF11. Left: total potential energy of the system as a function of time over 200 ns. Center: solute RMSD from the reference structure. Right: R_g as a function of time over 200 ns. Panels: (a) **4a**, (b) **4i**, (c) **4m**, (d) **4o**.

Paragraph S1. Principal component analysis of TOP2A and Eg5/KIF11 trajectories.

Figure S5. PCA of the MD trajectories of the ligand-TOP2A complexes. Left: PCA eigenvalue spectrum as a function of the eigenvalue number, showing that the first two components (PC1 and PC2) capture most of the conformational variance. Central: Time evolution of the PC1 (blue) and PC2 (orange) projections over 200 ns of simulations. Right: 2D scatter plots of the PC1 vs. PC2 conformational space. Panels: (a) **4a**, (b) **4i**, (c) **4m**, and (d) **4o**.

Figure S6. PCA of the MD trajectories of the ligand-Eg5 complexes. Left: PCA eigenvalue spectrum as a function of the eigenvalue number, showing that the first two components (PC1 and PC2) capture most of the conformational variance in the MD trajectories. Central: Time evolution of the PC1 (blue) and PC2 (orange) projections over 200 ns simulations. Right: 2D scatter plots of the PC1 vs. PC2 conformational space. Panels: (a) **4a**, (b) **4i**, (c) **4m**, and (d) **4o**.

^{1}H, ^{13}C, DEPT 135, and, where applicable, ^{19}F NMR spectra.

Table S1. SuperPred 3.0 predictions for the six targets carried to the GNINA screening (probability, %). The SuperPred 3.0 export lists targets with a predicted probability of at least 50%. Eg5/KIF11 was not predicted for any compound.

Target (ChEMBL ID)	4a	4e	4f	4h	4i	4m	4o	Model accuracy (%)
DNA topoisomerase I (T09826)	53.76	64.25	57.13	61.22	61.45	68.64	55.29	97
DNA topoisomerase II alpha (T17048)	66.92	74.67	80.21	83.72	76.03	79.52	85.53	89
Aurora kinase B/INCENP (T46781)	73.11	81.69	86.11	70.87	77.98	61.12	67.74	97.5
PI3-kinase p110-alpha/p85-alpha (T80276)	80.61	72.26	78.82*	79.60	75.14	68.80	71.84	94.3
Histone deacetylase 8 (T28887)	73.20	66.15	not predicted	70.07	64.20	68.82	83.16	94
Histone deacetylase 10 (T94324)	not predicted	56.4	78.88	82.61	70.36	78.26	83.16	90.1
Eg5/KIF11	not predicted	not predicted	not predicted	not predicted	not predicted	not predicted	not predicted	–

* For **4f**, SuperPred also lists a second PI3-kinase p110-alpha subunit entry with the same ChEMBL ID (59.53%).

Table S2. Protein structures used for docking and redocking validation.

Target	PDB ID	Method, resolution	Co-crystallized ligand	Redocking RMSD (Å)
TOP2A	6ZY5	Cryo-EM, 3.60 Å	Etoposide (EVP)	2.12
Eg5/KIF11	6G6Y	X-ray, 1.80 Å	thiadiazolidine inhibitor (EOK)	0.23
DNA topoisomerase I	1SC7	X-ray, 3.00 Å	Indenoisoquinoline MJ-II-38 (M38)	1.14
Aurora kinase B	4AF3	X-ray, 2.75 Å	VX-680 (VX6)	0.99
HDAC8	2V5X	X-ray, 2.25 Å	hydroxamate inhibitor (V5X)	2.64
HDAC10	7U3M	X-ray, 2.10 Å	N-methylpiperazine benzhydroxamic acid (L8C)	1.10
PI3Kα	8EXL	X-ray, 1.99 Å	Taselisib (799)	1.10

Table S3. Predicted intermolecular interactions between compounds **4a**, **4e**, **4f**, **4h**, **4i**, **4m**, and **4o** and the Eg5/KIF11 binding site.

Ligand	Hydrogen bonds	Charge	Aromatic/Hydrophobic
**4a**	Arg119, Arg221	Glu118	Pro121, Leu132, Ile136, Pro137, Leu214
**4m**	Glu118, Leu214	Glu116	Arg119, Ile136, Pro137, Tyr211, Ala218
**4i**	Arg119, Arg221	—	Ile136, Pro137, Leu214
**4h**	Arg119, Leu214	Glu118	Ile136, Pro137, Tyr211, Ala218
**4f**	Arg119, Arg221	Glu118	Phe113, Ile136, Pro137, Leu214
**4e**	Glu116, Arg221	Glu215	Arg119, Ile136, Pro137, Leu214, Ala218
**4o**	Gly117, Arg119	Glu118	Pro137, Tyr211, Ala218

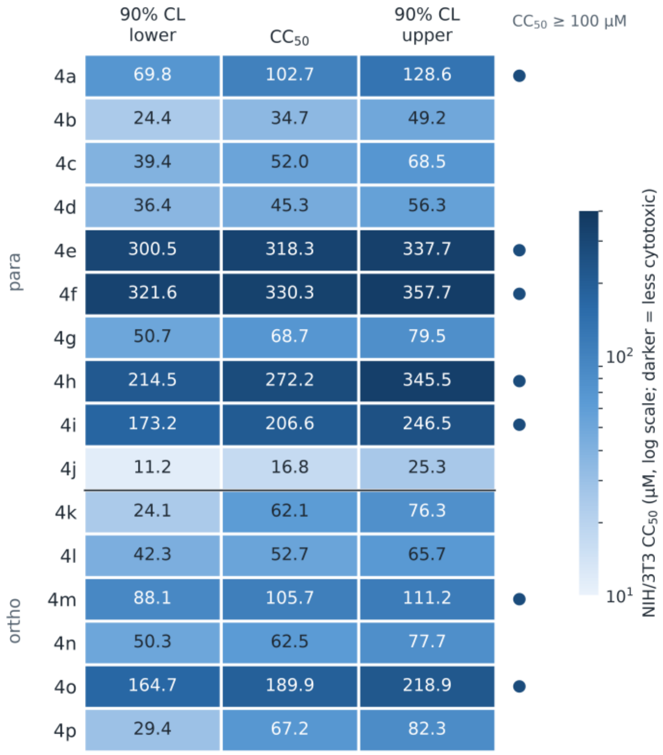

Figure S1. Heatmap of the NIH/3T3 CC_{50} values and 90% confidence limits (CL) in Table 2. Colors indicate the CC_{50} value on a logarithmic scale (darker = less cytotoxic). Dots mark the compounds with CC_{50} ≥100 μM that were retained for MCF-7 profiling.

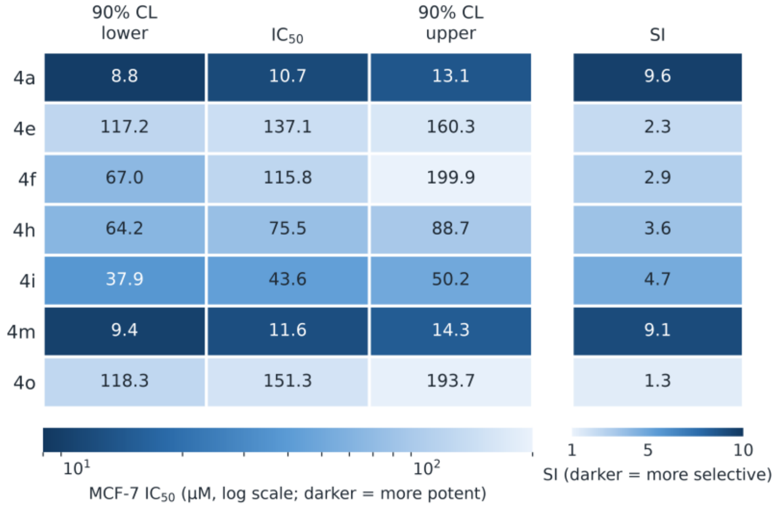

Figure S2. Heatmap of the MCF-7 IC_{50} values with 90% CL and the selectivity indices (SI) in Table 3. Darker colors indicate higher potency (IC_{50}, logarithmic scale) and higher selectivity (SI).

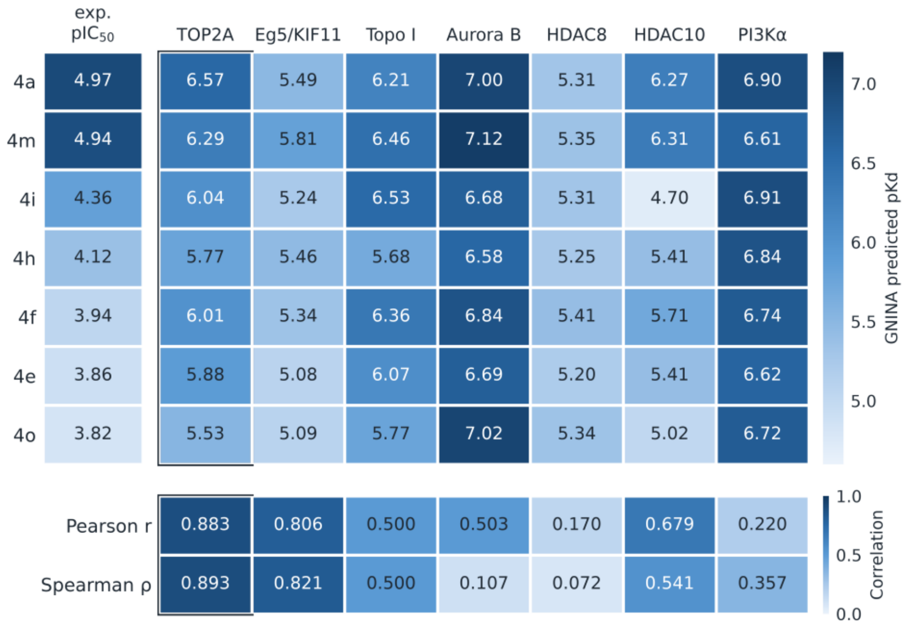

Figure S3. Heatmap of the GNINA CNN affinity values (predicted pK_d) in Table 5 and of the Pearson and Spearman correlations with the experimental pIC_{50} (n = 7). The TOP2A column is outlined. Darker colors indicate higher values.

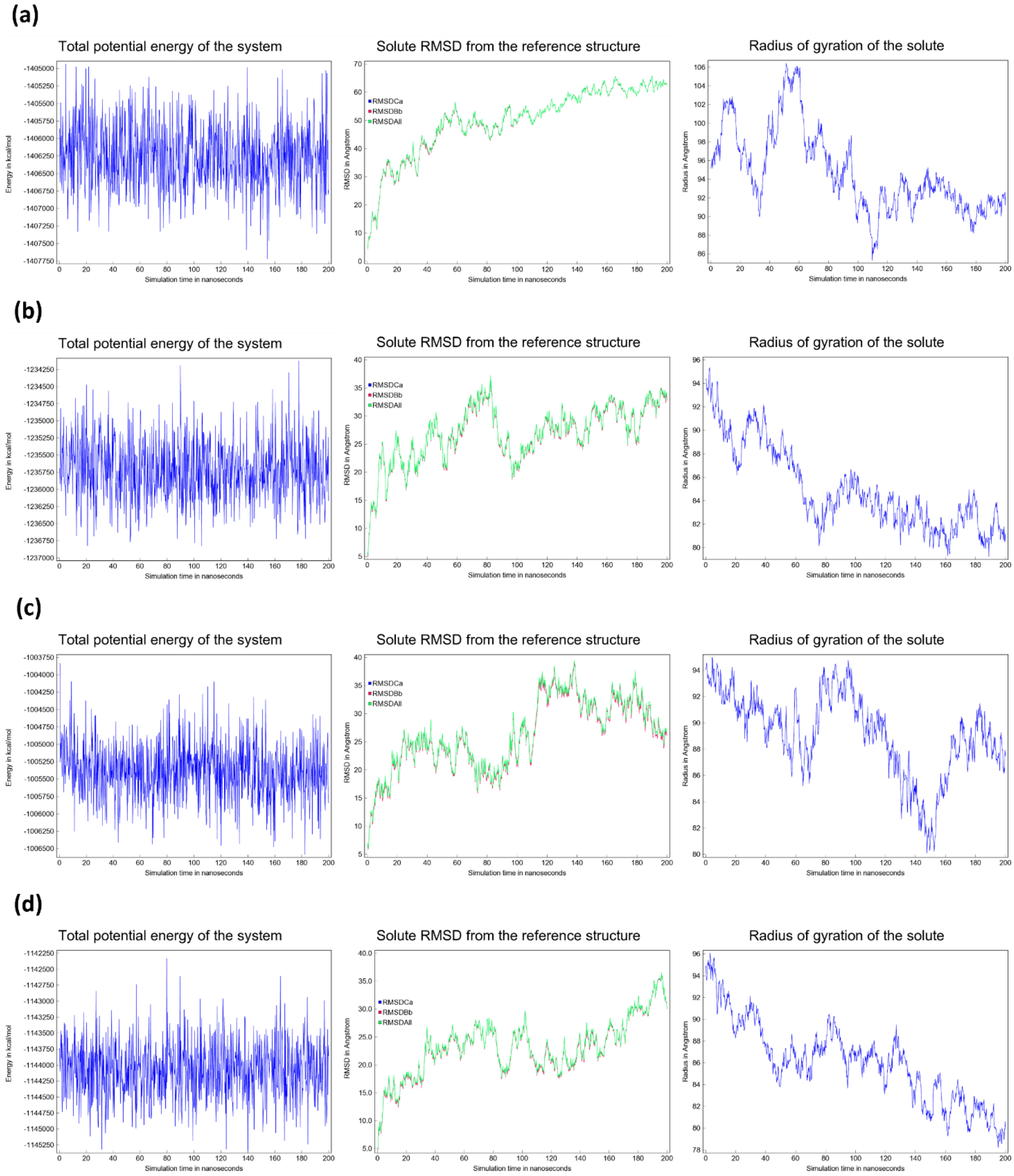

Figure S4. MD results for Eg5/KIF11. Left: total potential energy of the system as a function of time over 200 ns. Center: solute RMSD from the reference structure. Right: R_g as a function of time over 200 ns. Panels: (a) **4a**, (b) **4i**, (c) **4m**, (d) **4o**.

Paragraph S1. Principal component analysis of the TOP2A and Eg5/KIF11 trajectories

To further characterize the conformational behavior of the TOP2A complexes beyond the thermodynamic information provided by MM/PBSA, principal component analysis (PCA) was performed on the MD trajectories (Fig. S5). In all systems, the eigenvalue spectra exhibited a steep decay within the first few principal components, indicating that most of the collective motion was captured by PC1 and PC2 and that the trajectories were dominated by a limited number of essential conformational modes. However, clear differences emerged among the four ligand–TOP2A complexes. Compound **4a** displayed a pronounced drift along PC1 during the simulation, accompanied by the broadest conformational distribution and the presence of distinct clusters in the PC1–PC2 space, suggesting the progressive exploration of alternative conformational substates. This behavior is consistent with the relatively weak MM/PBSA binding energy and supports the notion of a

complex characterized by a less favorable average MM/PBSA estimate and broader conformational sampling. Compound **4i** showed a more gradual transition between conformational states, with a continuous redistribution of the trajectory across the essential subspace and moderate conformational heterogeneity, in agreement with its intermediate biological and thermodynamic profiles. In contrast, the TOP2A–**4m** complex sampled a more confined region of the essential conformational landscape, despite retaining measurable fluctuations along the principal components. The presence of well-populated and recurrent conformational basins indicates a stable dynamic equilibrium rather than structural instability, supporting the interpretation of **4m** as a ligand capable of maintaining a favorable adaptive intercalative binding mode throughout the course of the simulation. Finally, compound **4o** explored a broader and less densely populated conformational space than **4m**, although without the marked directional drift observed for **4a**, suggesting a less compact and more heterogeneous interaction pattern. Overall, the PCA showed that the complexes sampled distinct conformational regimes within the simulated interval. In particular, the restricted and recurrent conformational sampling observed for **4m** is consistent with the formation of the most confined TOP2A conformational landscape among the simulated systems, further supporting its prioritization as the most promising lead in the series.

To complement the conformational analysis of the TOP2A complexes, PCA was performed on the MD trajectories of the four ligand–Eg5 complexes selected for this target (**4a**, **4i**, **4m**, and **4o**) (Fig. S6). As observed for TOP2A, the eigenvalue spectra showed a rapid decay after the first two components, indicating that PC1 and PC2 captured the dominant collective motions of each trajectory. However, the conformational landscapes sampled by the four Eg5 complexes were markedly more heterogeneous and less convergent than those of their TOP2A counterparts. Compound **4a** displayed a broad and directionally drifted distribution in the PC1–PC2 space, consistent with its progressive, but incomplete, RMSD stabilization during the simulation. Compound **4m** showed a wider and more dispersed conformational sampling with no well-defined basins, in accordance with the biphasic R_g profile and the absence of a stable RMSD plateau observed for this compound against Eg5. Compound **4i** displayed a conformational sampling pattern broadly similar to that of **4m**, with a comparably wide and dispersed distribution in the PC1–PC2 space and no clearly defined recurrent basins, consistent with the resemblance between the two trajectories evident in Fig. S4. Compound **4o** explored a comparably broad and heterogeneous essential space. Notably, none of the four Eg5 complexes exhibited the compact, recurrent conformational sampling that characterized the TOP2A–**4m** system, and no clear ranking emerged from the PCA that paralleled the experimental phenotypic hierarchy. Overall, the Eg5 PCA landscapes lacked the compound-discriminating structure observed for TOP2A, providing no conformational basis for ranking the active subset and further supporting the secondary role assigned to this target.

Taken together, the MD simulations reinforce the prioritization of TOP2A as the primary mechanistic hypothesis for the active subset of compounds. In particular, TOP2A provides the most coherent dynamic framework for explaining the baseline antiproliferative ranking, whereas Eg5/KIF11 may still offer secondary information useful for differentiating some compound-specific interaction patterns, especially between **4m** and **4a**. Thus, the overall computational evidence supports a TOP2A-primary/Eg5-secondary interpretation rather than a model primarily centered on Eg5.

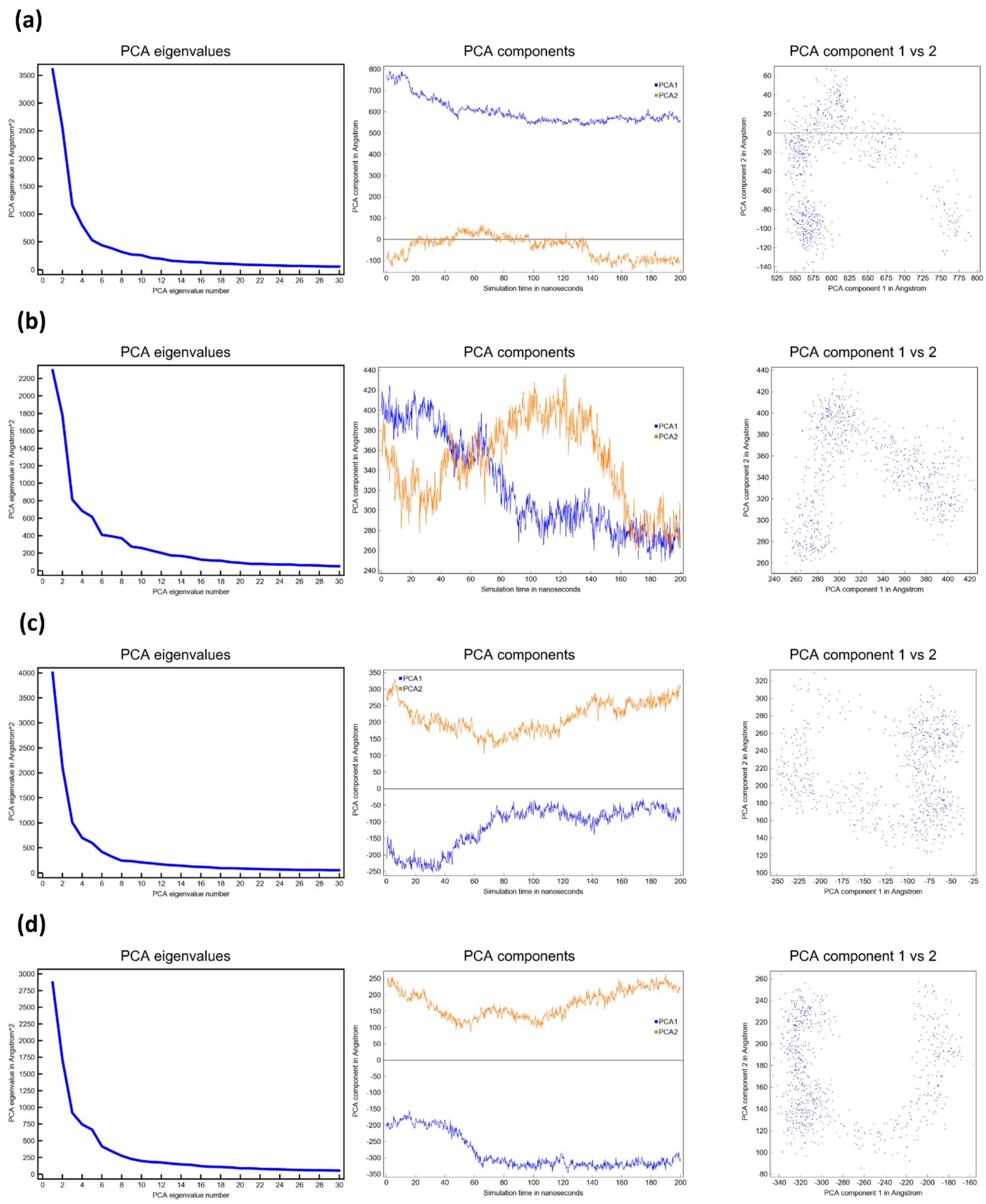

Figure S5. PCA of the MD trajectories of the ligand–TOP2A complexes. Left: PCA eigenvalue spectrum as a function of the eigenvalue number, showing that the first two components (PC1 and PC2) capture most of the conformational variance. Central: Time evolution of PC1 (blue) and PC2 (orange) projections over 200 ns simulations. Right: 2D scatter plots of PC1 vs. PC2 conformational space. Panels: (a) **4a**, (b) **4i**, (c) **4m**, and (d) **4o**.

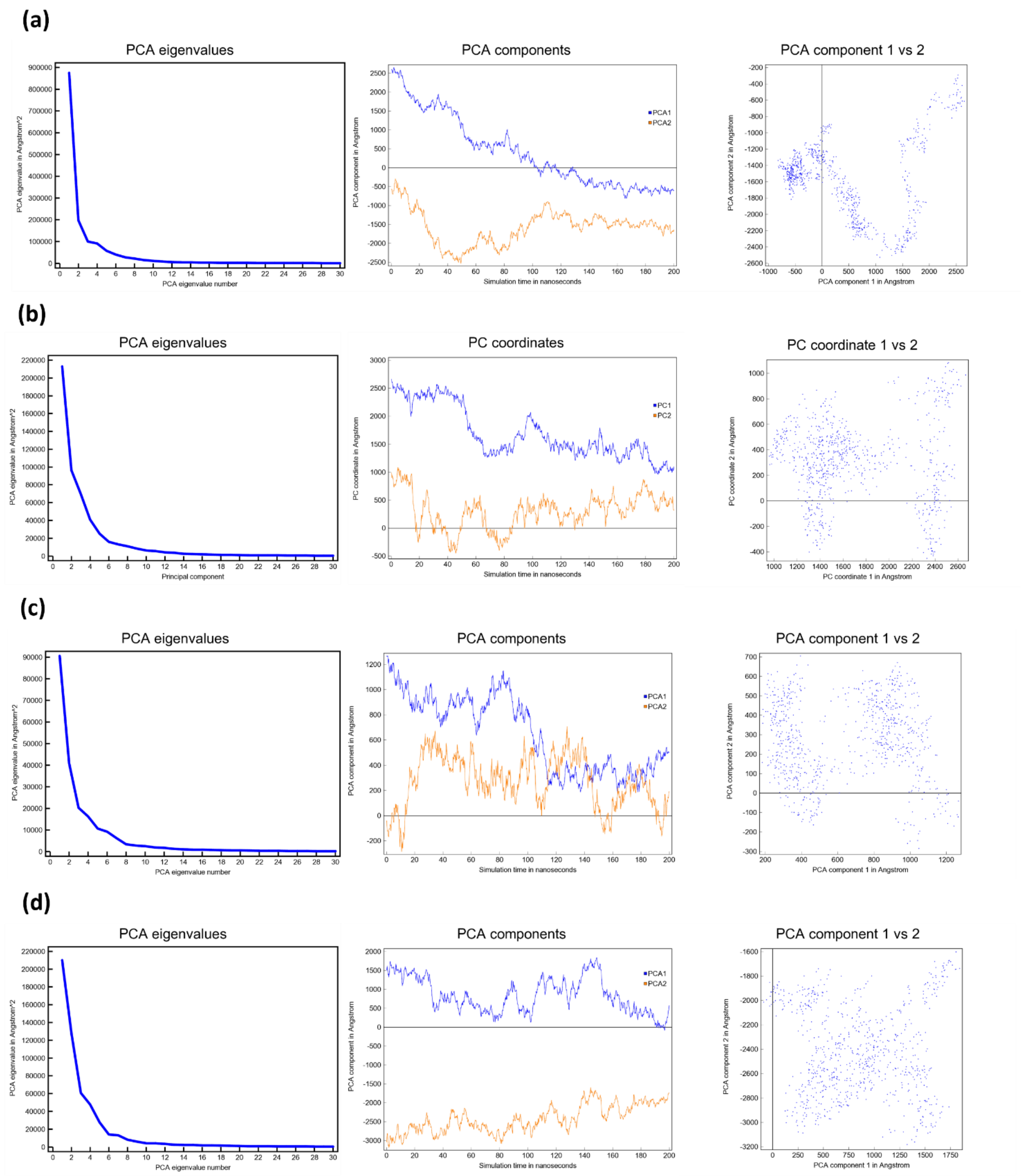

Figure S6. PCA of the MD trajectories of the ligand-Eg5 complexes. Left: PCA eigenvalue spectrum as a function of the eigenvalue number, showing that the first two components (PC1 and PC2) captured most of the conformational variance. Central: Time evolution of the PC1 (blue) and PC2 (orange) projections over 200 ns of simulations. Right: 2D scatter plots of PC1 vs. PC2 conformational spaces. Panels: (a) **4a**, (b) **4i**, (c) **4m**, and (d) **4o**.

^{1}H, ^{13}C, DEPT 135, and, where applicable, ^{19}F NMR spectra

4a

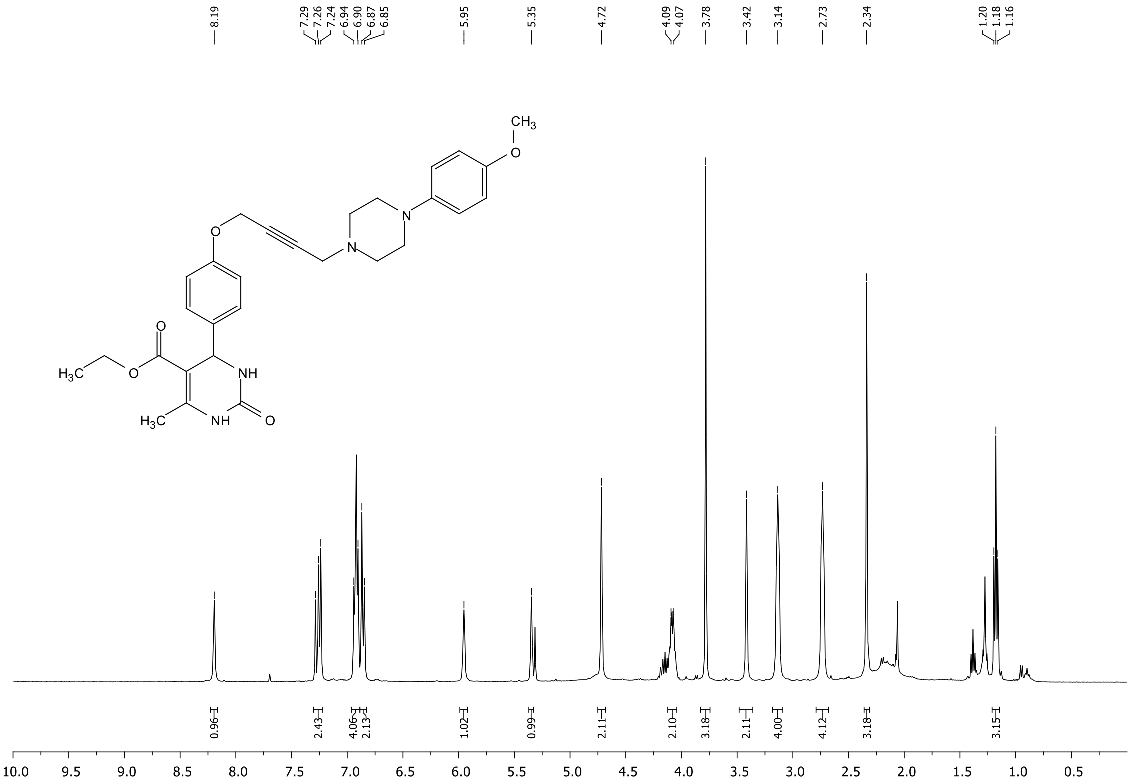

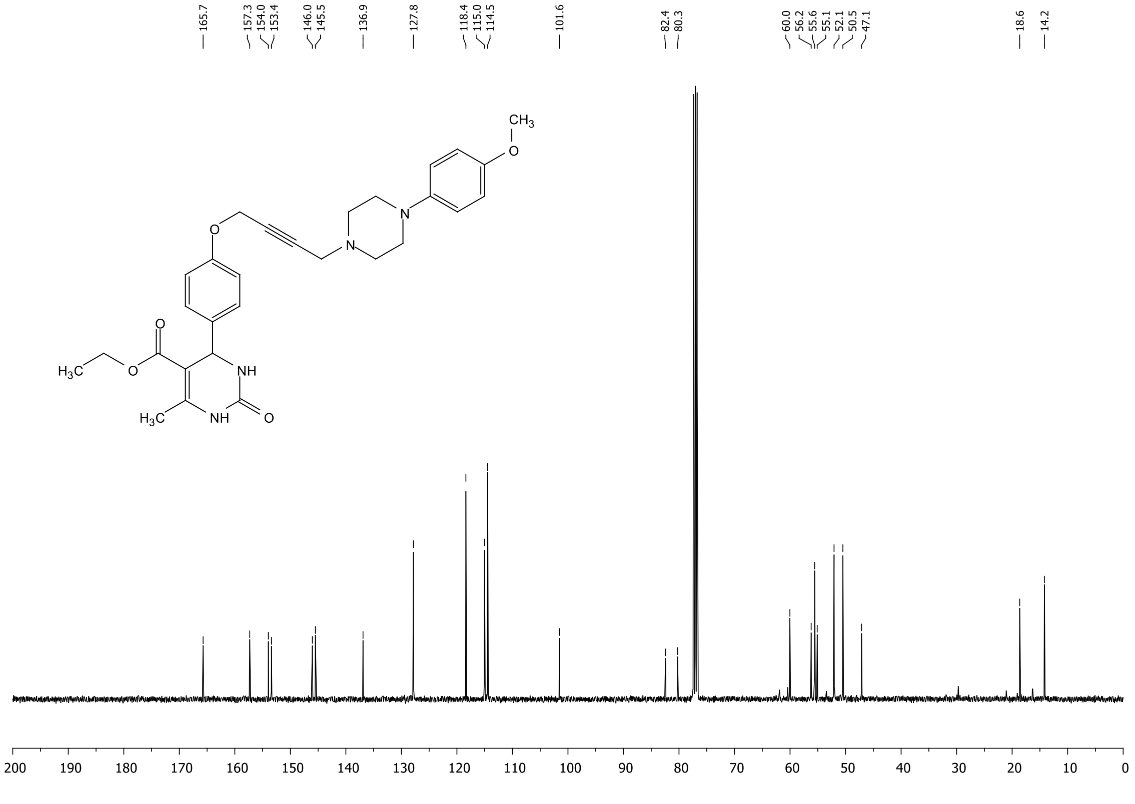

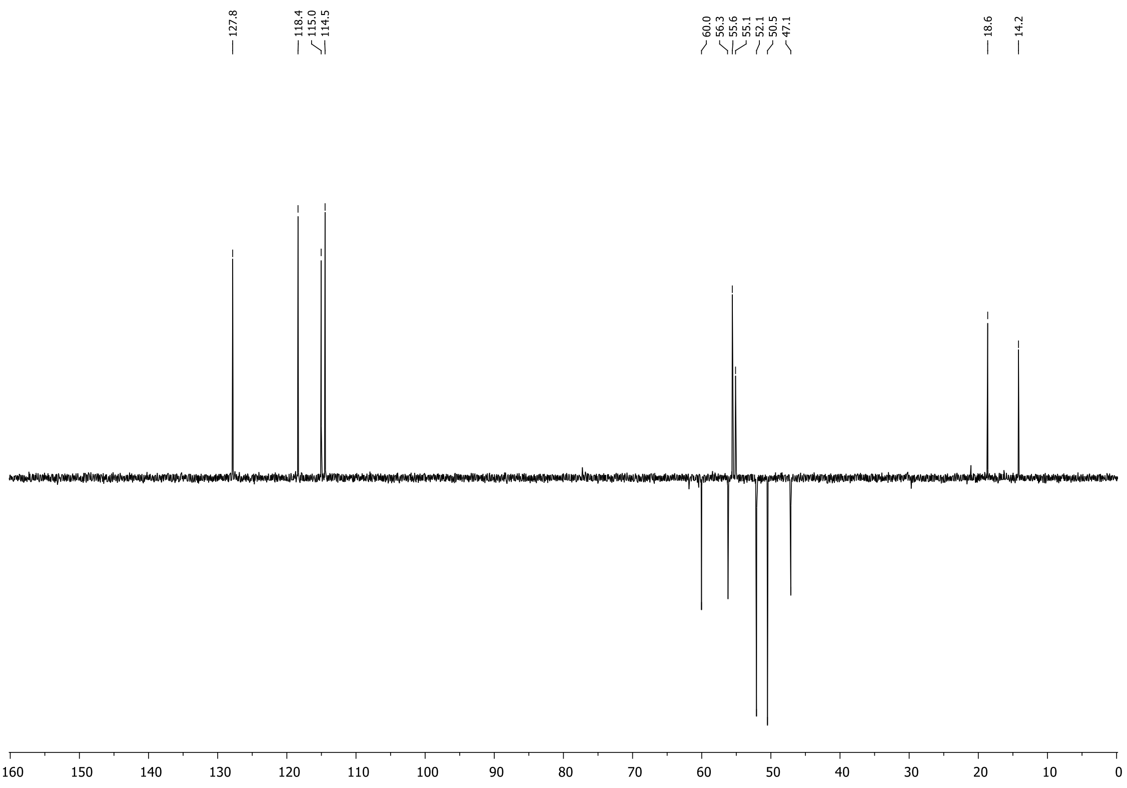
127.8
118.4
115.0
114.5
60.0
56.3
55.6
55.1
52.1
50.5
47.1
18.6
14.2
160 150 140 130 120 110 100 90 80 70 60 50 40 30 20 10 0

4b

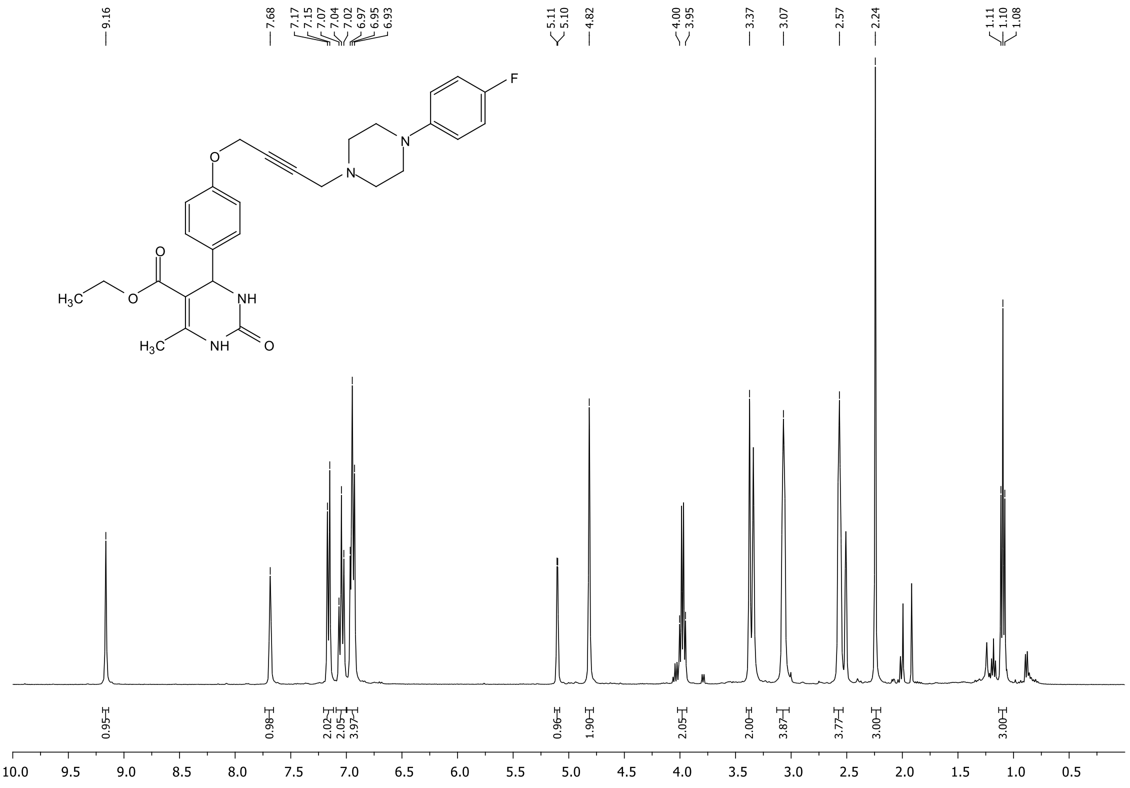
9.16
7.68
7.17 7.15 7.07 7.04 7.02 6.97 6.95 6.93
5.11 5.10
4.82
4.00 3.95
3.37
3.07
2.57
2.24
1.11 1.10 1.08
F
N
N
O
O
O
H3C
NH
H3C
NH
O
0.95
0.98
2.02 2.05 3.97
0.96
1.90
2.05
2.00
3.87
3.77
3.00
3.00
10.0 9.5 9.0 8.5 8.0 7.5 7.0 6.5 6.0 5.5 5.0 4.5 4.0 3.5 3.0 2.5 2.0 1.5 1.0 0.5

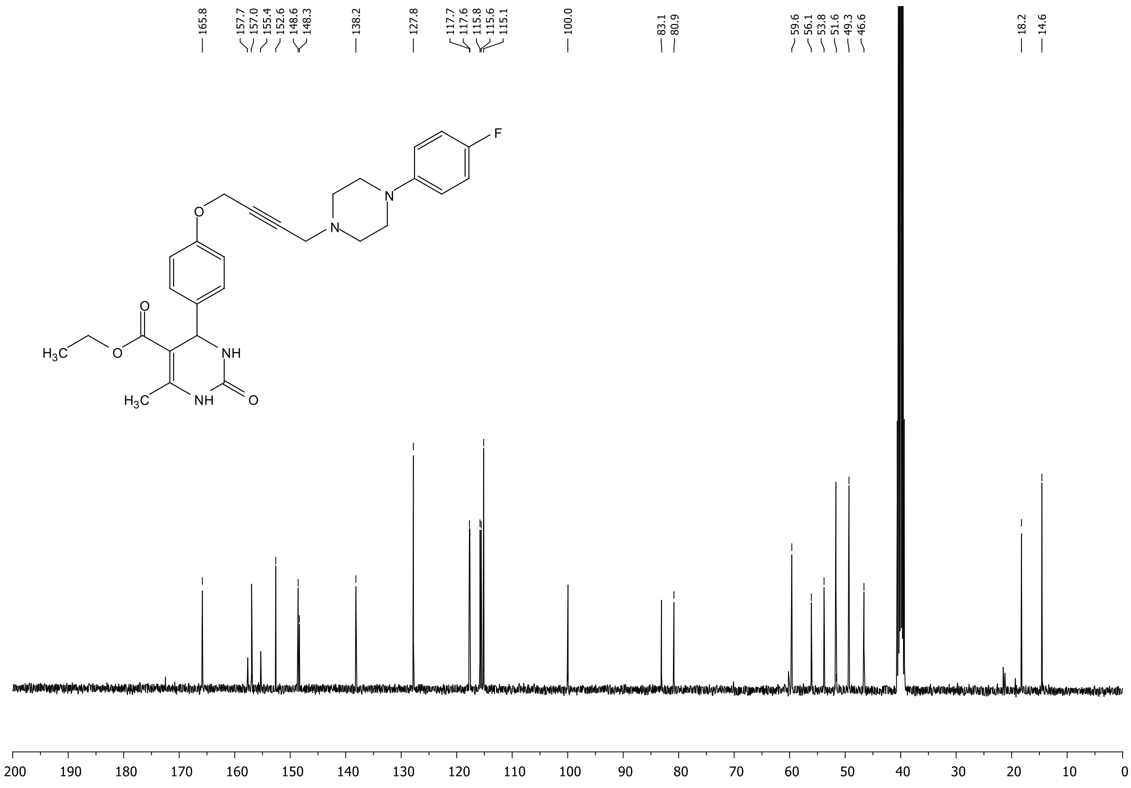
165.8
157.7 157.0 155.4 152.6 148.6 148.3
138.2
127.8
117.7 117.6 115.8 115.6 115.1
100.0
83.1 80.9
59.6 56.1 53.8 51.6 49.3 46.6
18.2
14.6
F
N
N
O
O
O
H3C
NH
H3C
NH
O
200 190 180 170 160 150 140 130 120 110 100 90 80 70 60 50 40 30 20 10 0

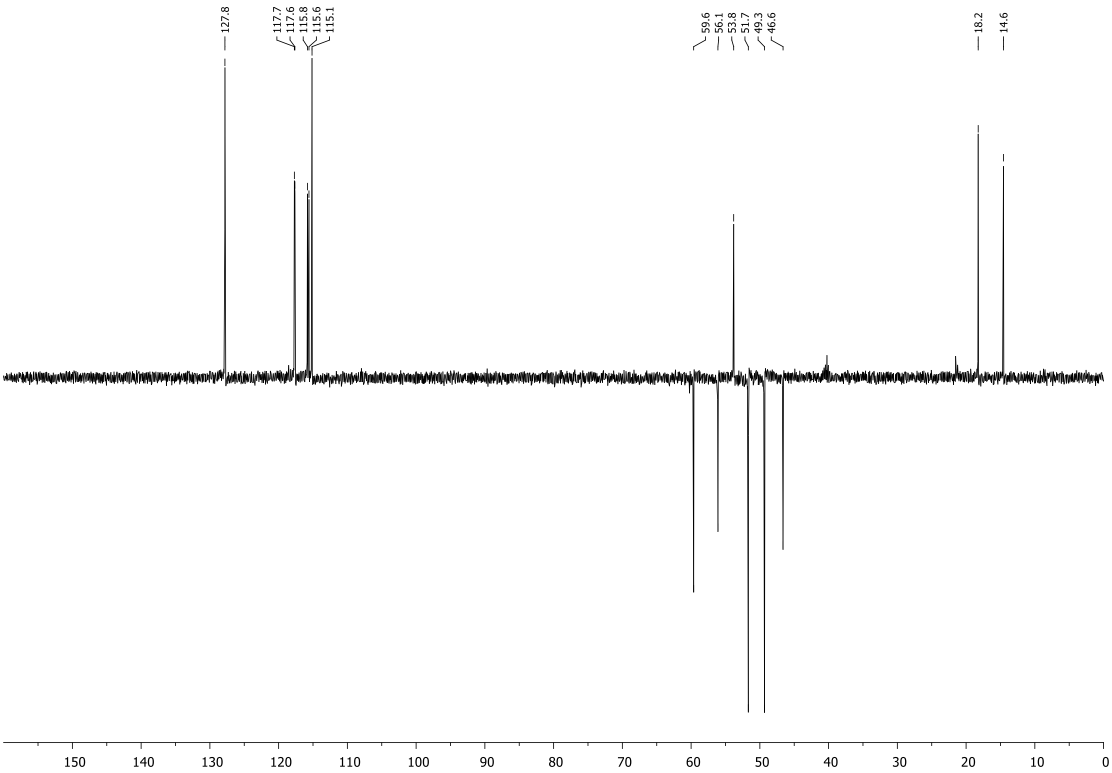
127.8
117.7
117.6
115.8
115.6
115.1
59.6
56.1
53.8
51.7
49.3
46.6
18.2
14.6
150 140 130 120 110 100 90 80 70 60 50 40 30 20 10 0

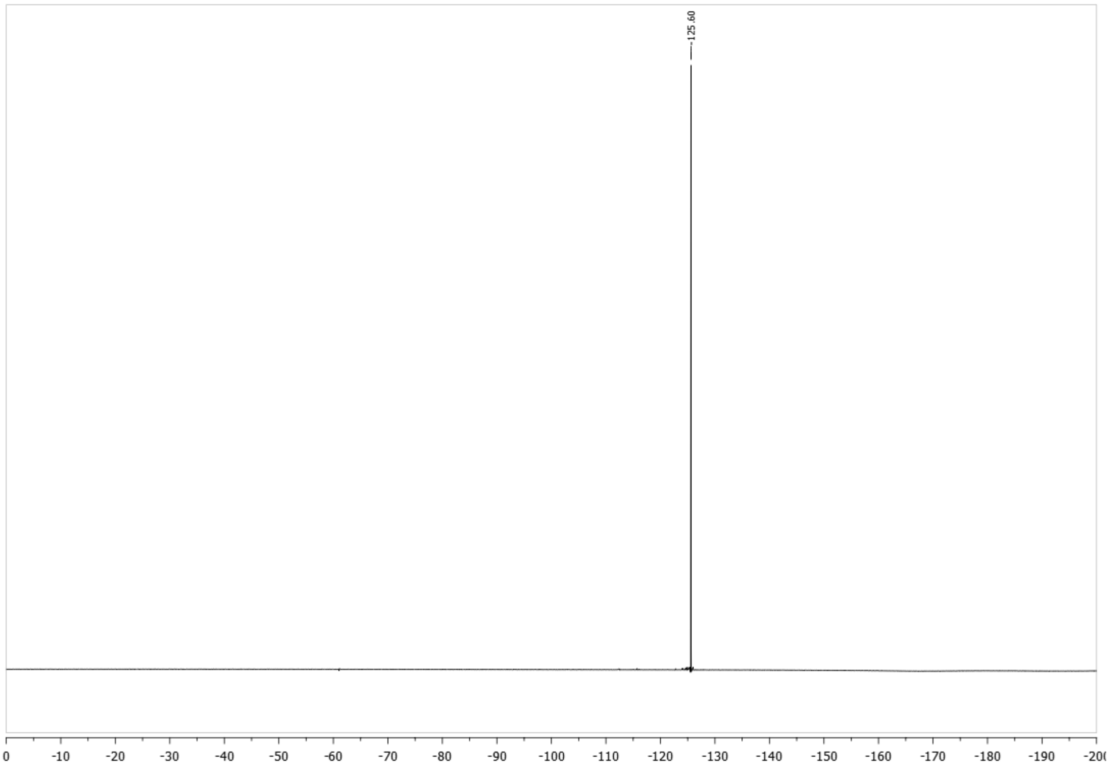
-125.60
0 -10 -20 -30 -40 -50 -60 -70 -80 -90 -100 -110 -120 -130 -140 -150 -160 -170 -180 -190 -200

4c

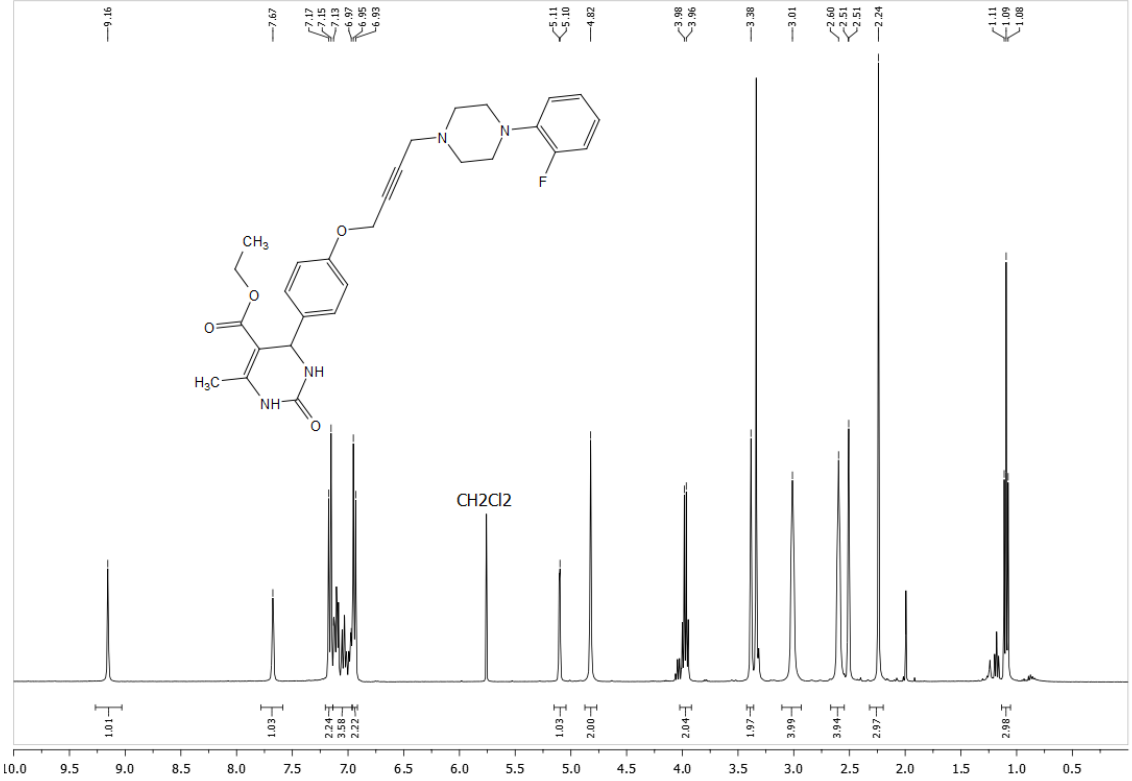

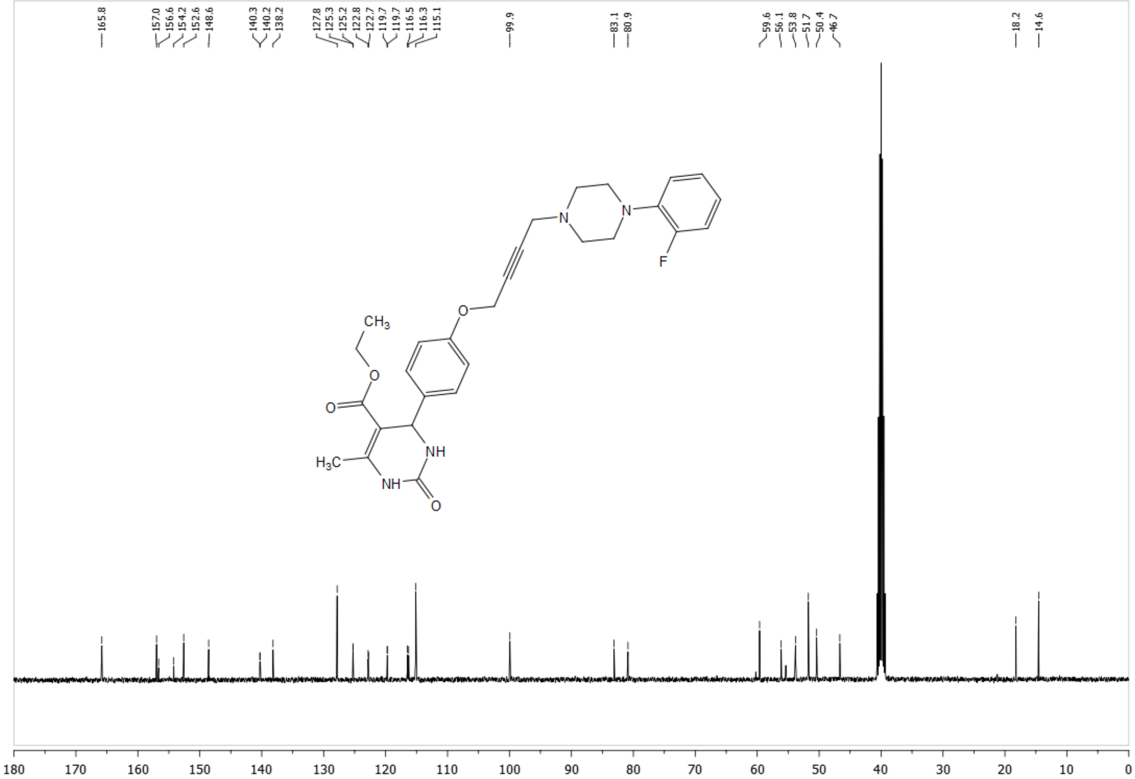

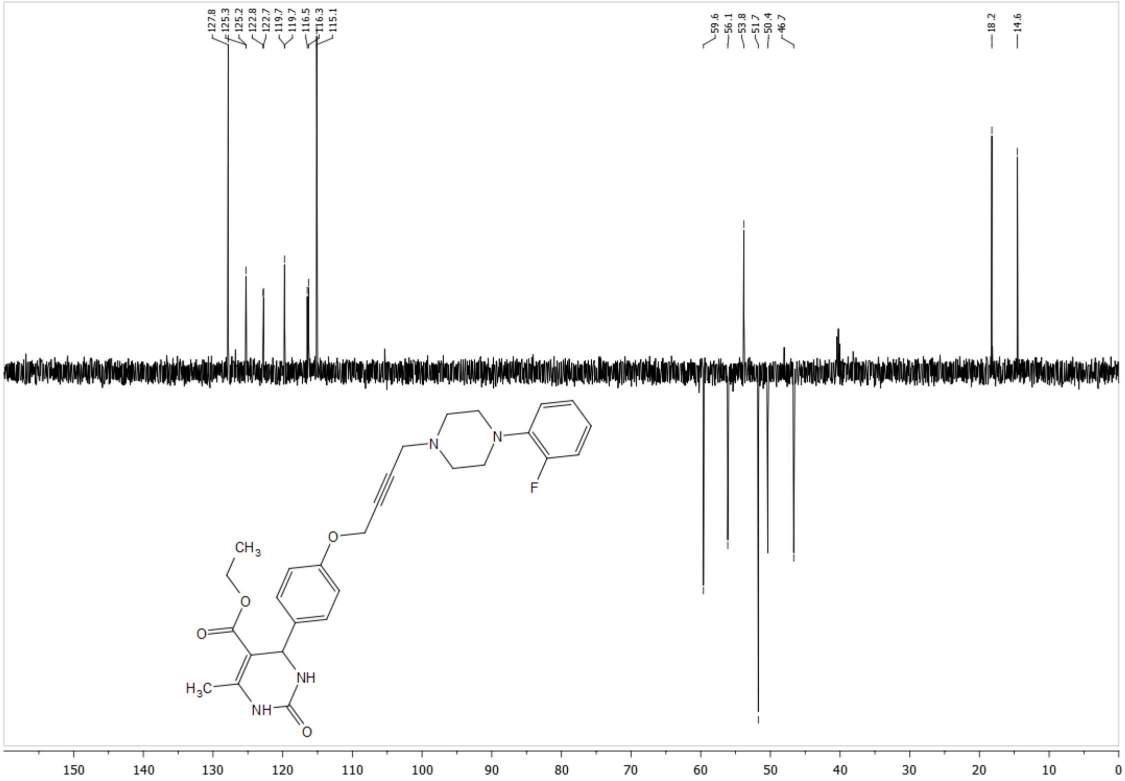
127.8
125.3
125.2
122.8
122.7
119.7
119.7
116.5
116.3
115.1
59.6
56.1
53.8
51.7
50.4
46.7
18.2
14.6
CH3
O
O
NH
H3C
NH
O
O
N
N
F
150
140
130
120
110
100
90
80
70
60
50
40
30
20
10
0

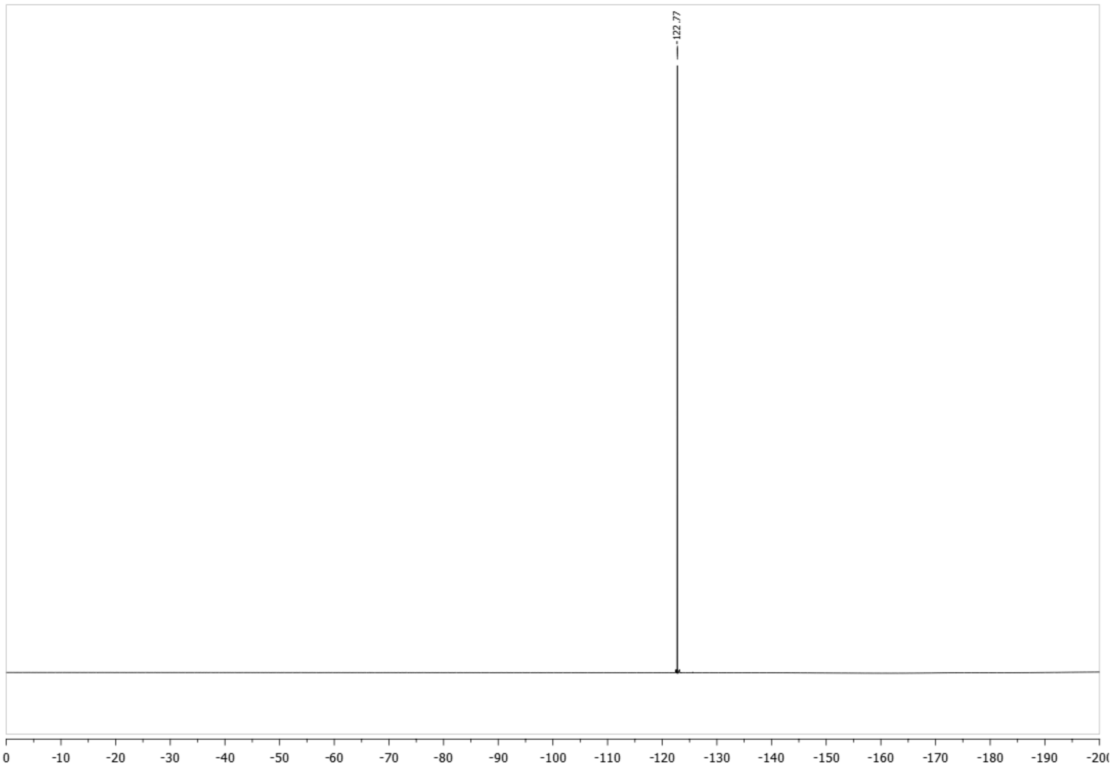
-122.77
0
-10
-20
-30
-40
-50
-60
-70
-80
-90
-100
-110
-120
-130
-140
-150
-160
-170
-180
-190

4d

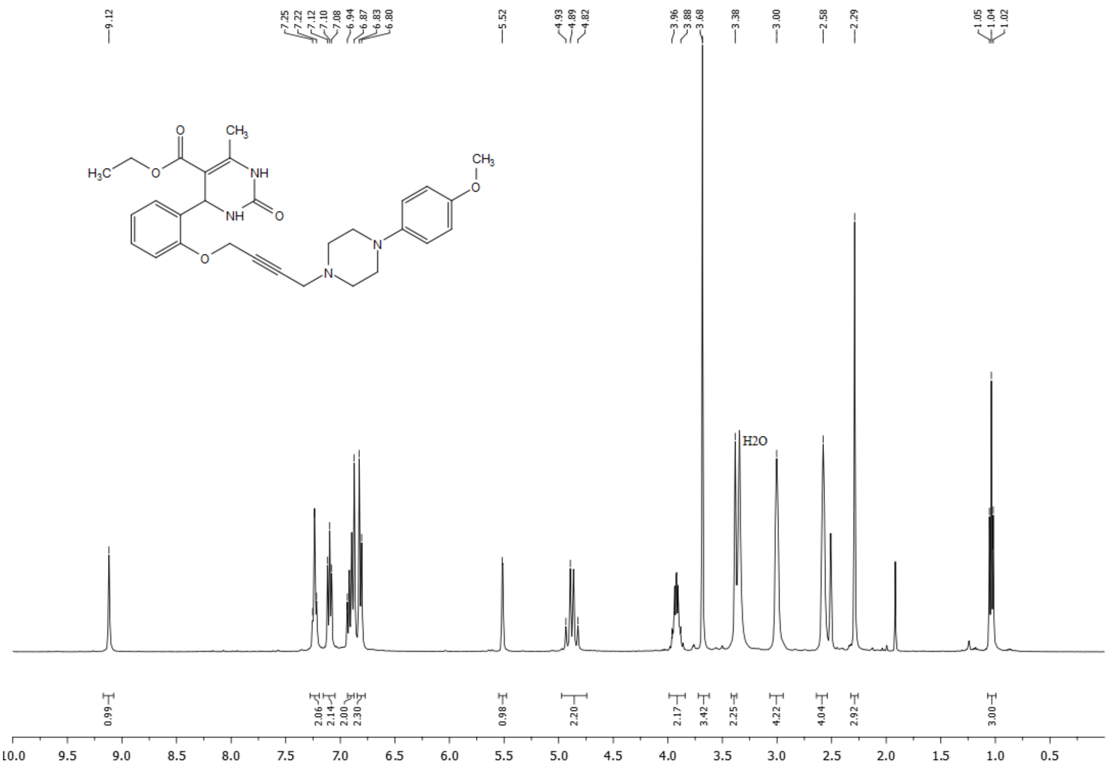
9.12
7.25
7.22
7.12
7.10
7.08
6.94
6.87
6.83
6.80
5.52
4.93
4.89
4.82
3.96
3.88
3.68
3.38
3.00
2.58
2.29
1.05
1.04
1.02
H2O
0.99
2.06
2.14
2.00
2.30
0.98
2.20
2.17
3.42
2.25
4.22
4.04
2.92
3.00

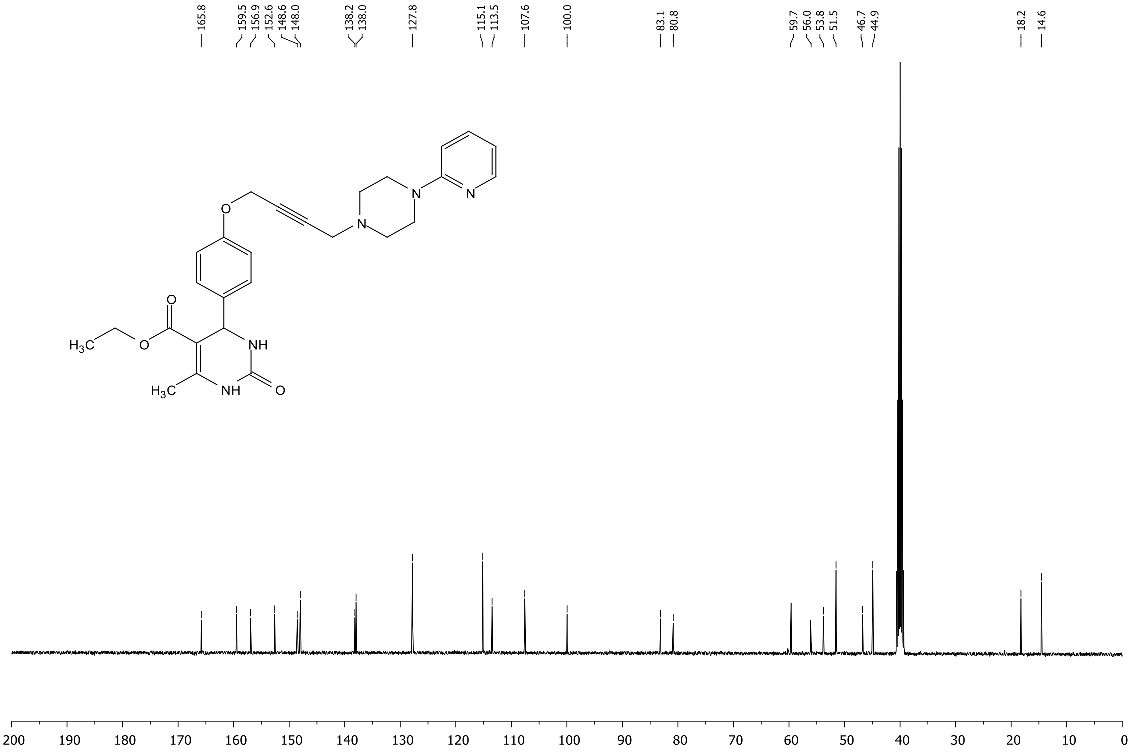
165.8
159.5
156.9
152.6
148.6
148.0
138.2
138.0
127.8
115.1
113.5
107.6
100.0
83.1
80.8
59.7
56.0
53.8
51.5
46.7
44.9
18.2
14.6

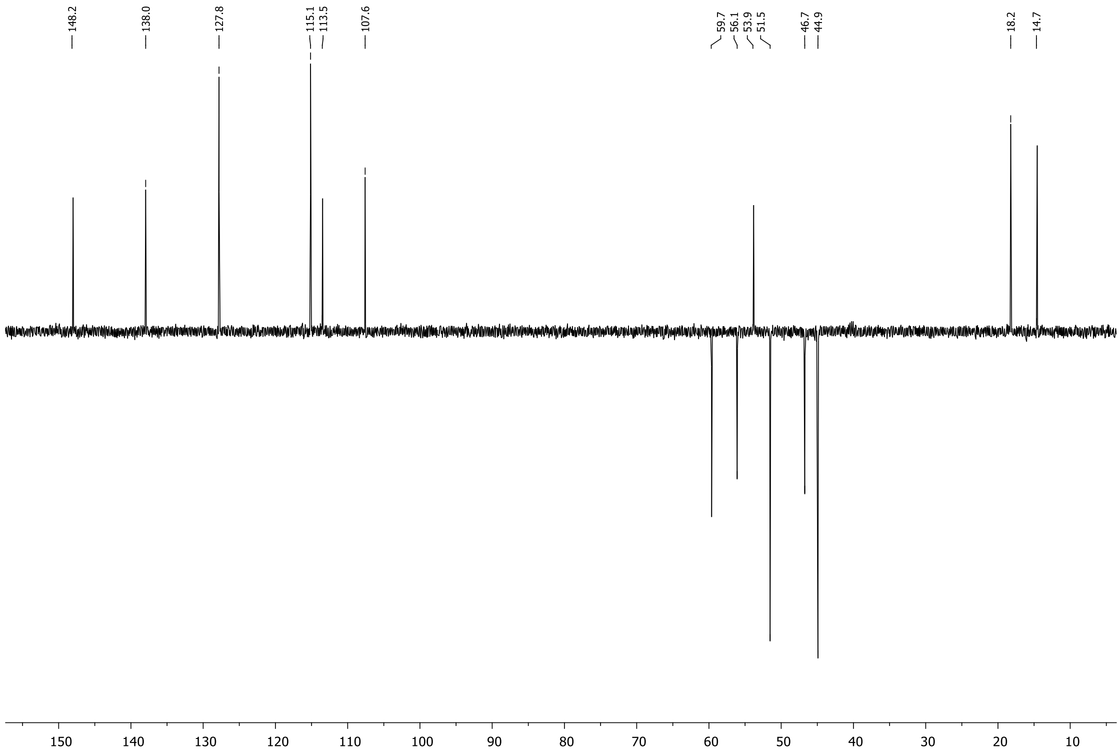
148.2
138.0
127.8
115.1
113.5
107.6
59.7
56.1
53.9
51.5
46.7
44.9
18.2
14.7
150 140 130 120 110 100 90 80 70 60 50 40 30 20 10

4e

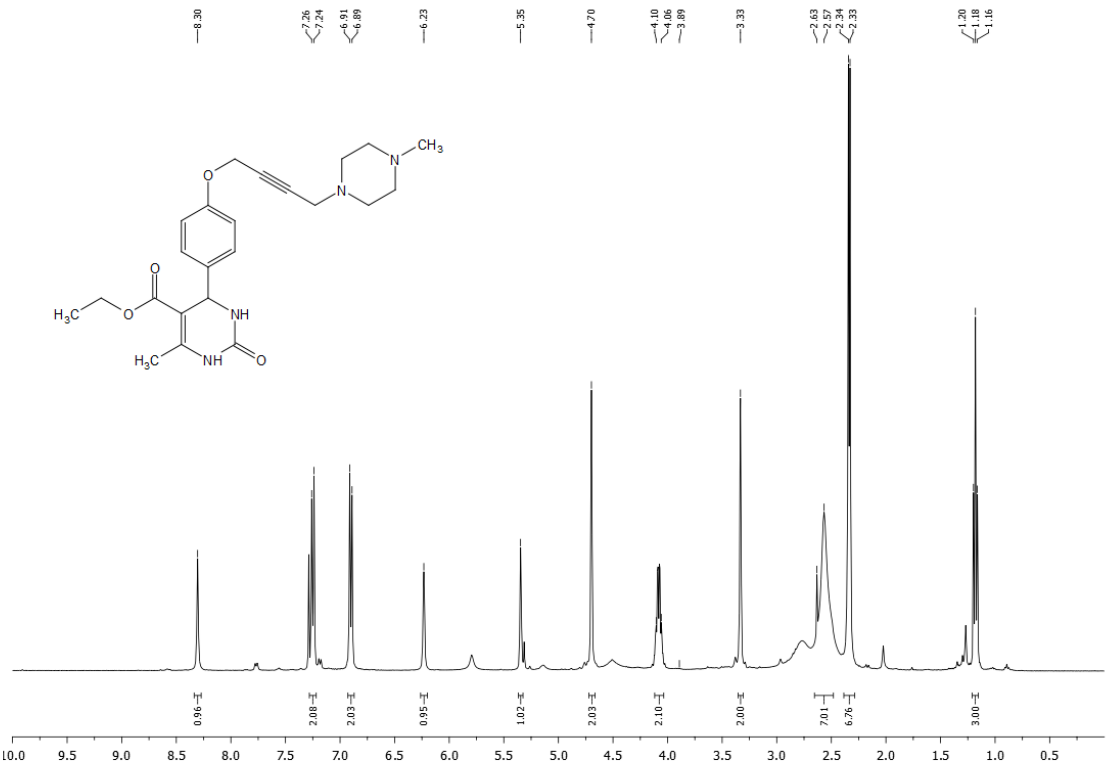
8.30
7.26
7.24
6.91
6.89
6.23
5.35
4.70
4.10
4.06
3.89
3.33
2.63
2.57
2.34
2.33
1.20
1.18
1.16
0.96
2.08
2.03
0.95
1.02
2.03
2.10
2.00
7.01
6.76
3.00
CH3
O
N
N
O
H3C
O
NH
H3C
NH
O

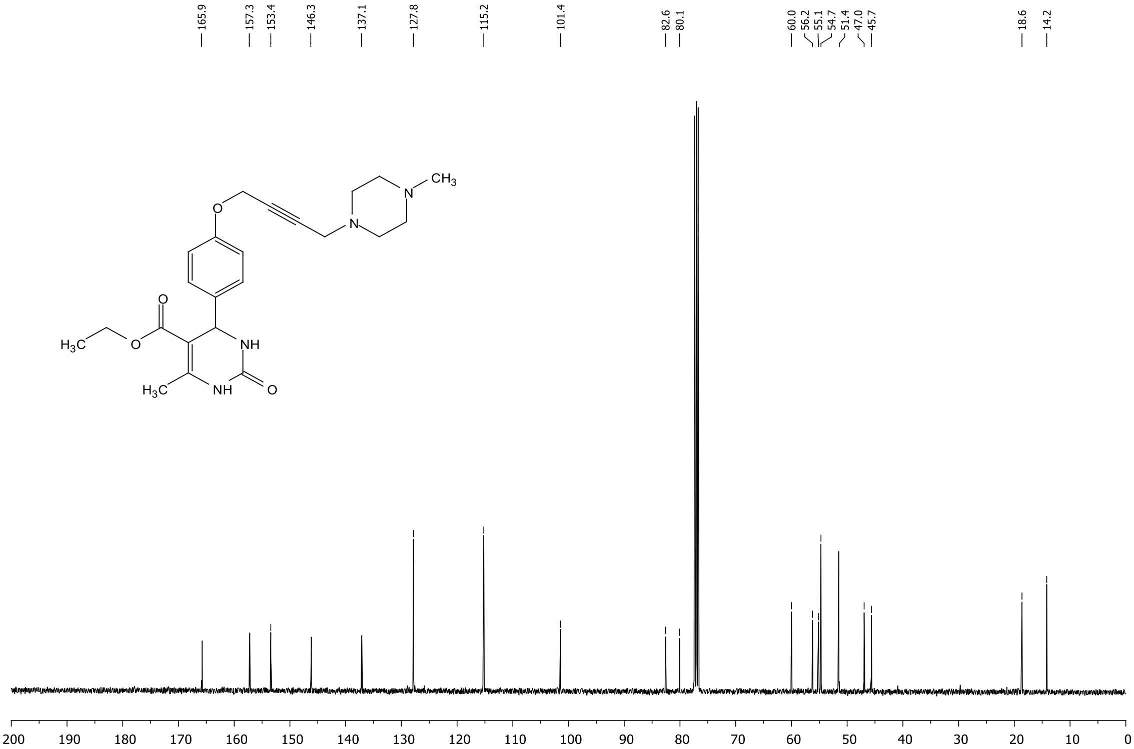
165.9
157.3
153.4
146.3
137.1
127.8
115.2
101.4
82.6
80.1
60.0
56.2
55.1
54.7
51.4
47.0
45.7
18.6
14.2
CH3
O
N
N
O
H3C
O
NH
H3C
NH
O

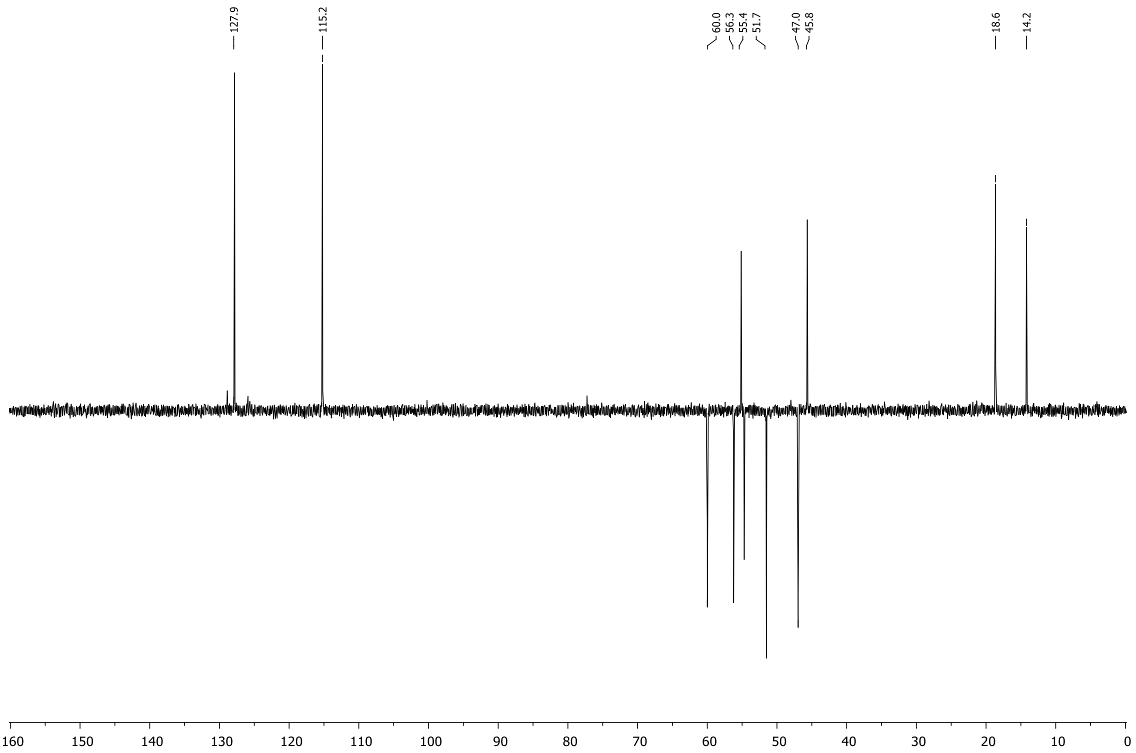
127.9
115.2
60.0
56.3
55.4
51.7
47.0
45.8
18.6
14.2
160
150
140
130
120
110
100
90
80
70
60
50
40
30
20
10
0

4f

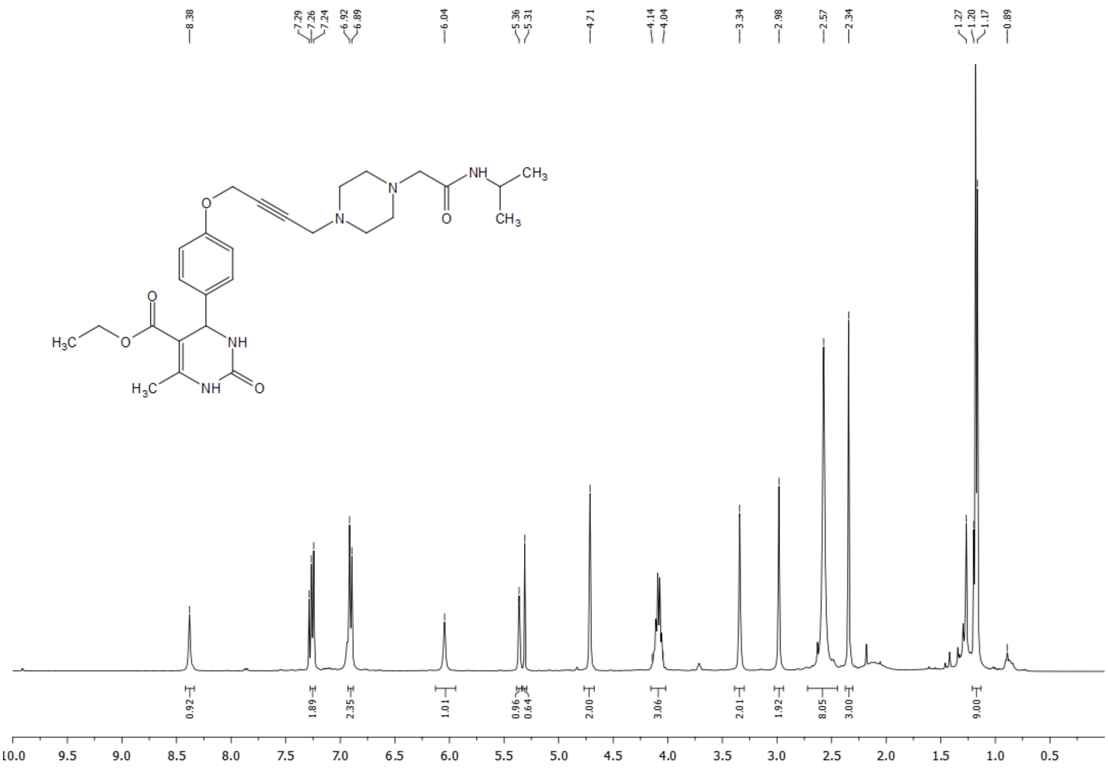

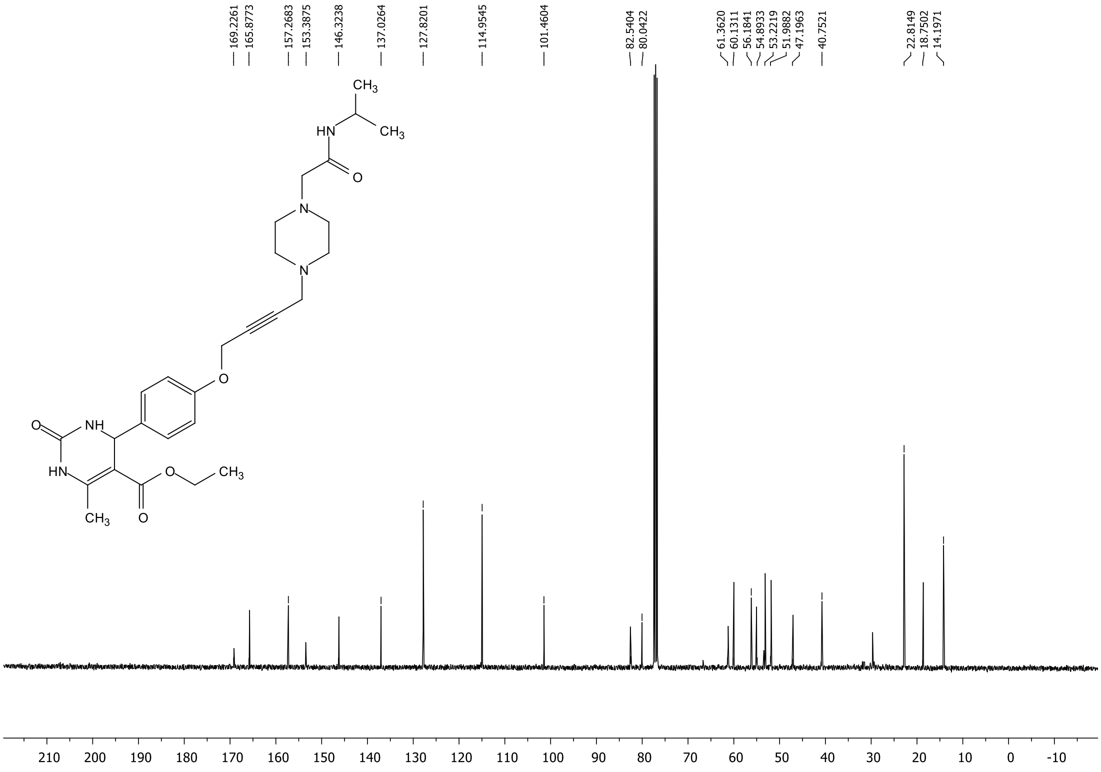

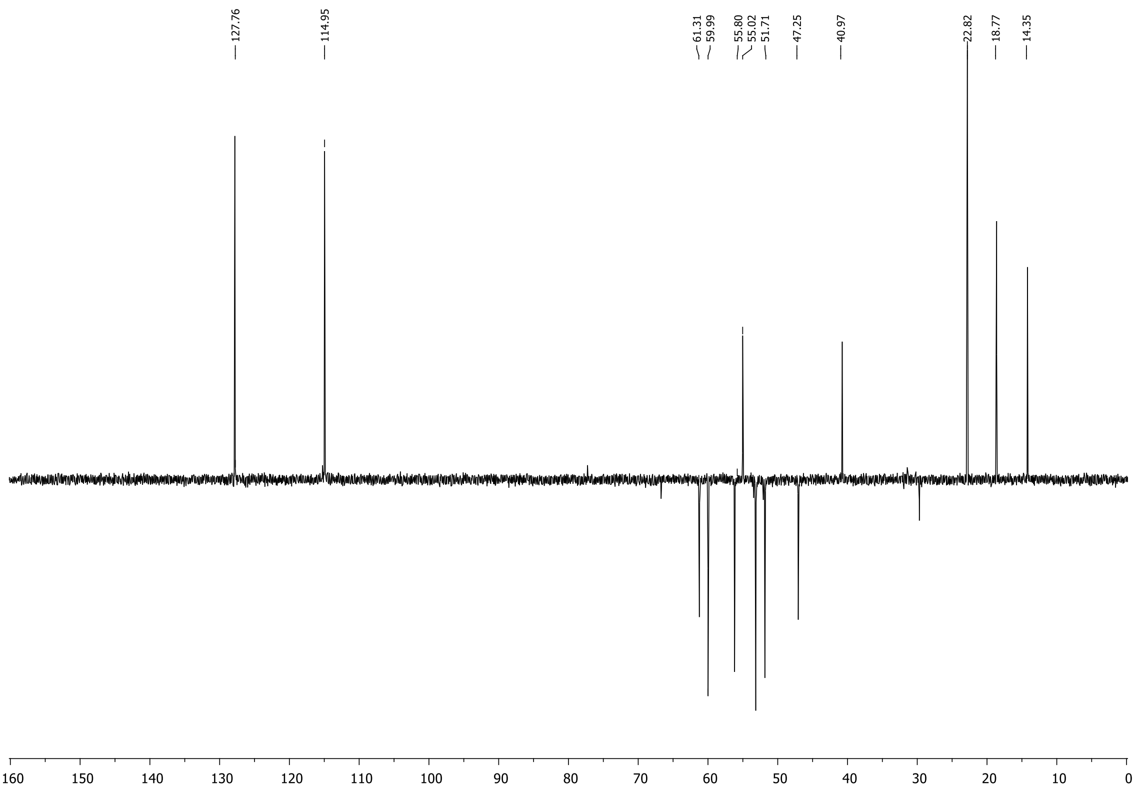
127.76
114.95
61.31
59.99
55.80
55.02
51.71
47.25
40.97
22.82
18.77
14.35
160
150
140
130
120
110
100
90
80
70
60
50
40
30
20
10
0

4g

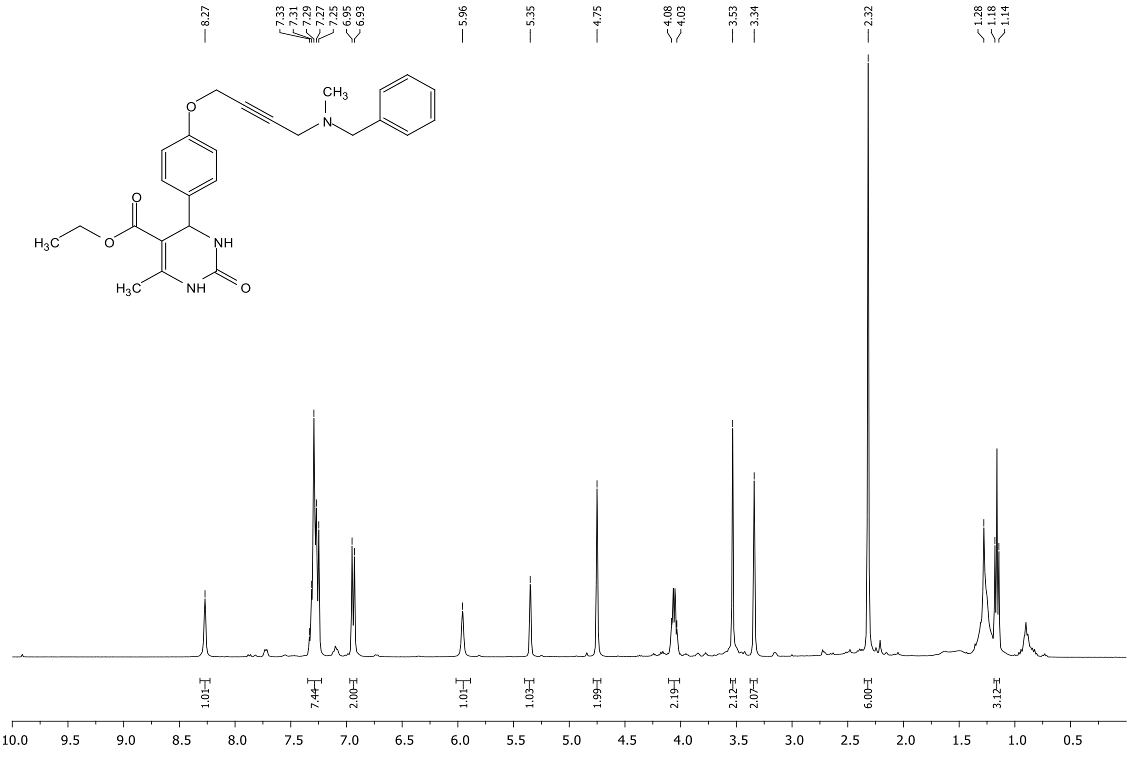
8.27
7.33
7.31
7.29
7.27
7.25
6.95
6.93
5.96
5.35
4.75
4.08
4.03
3.53
3.34
2.32
1.28
1.18
1.14
CH3
N
O
H3C
NH
NH
1.01
7.44
2.00
1.01
1.03
1.99
2.19
2.12
2.07
6.00
3.12
10.0
9.5
9.0
8.5
8.0
7.5
7.0
6.5
6.0
5.5
5.0
4.5
4.0
3.5
3.0
2.5
2.0
1.5
1.0
0.5

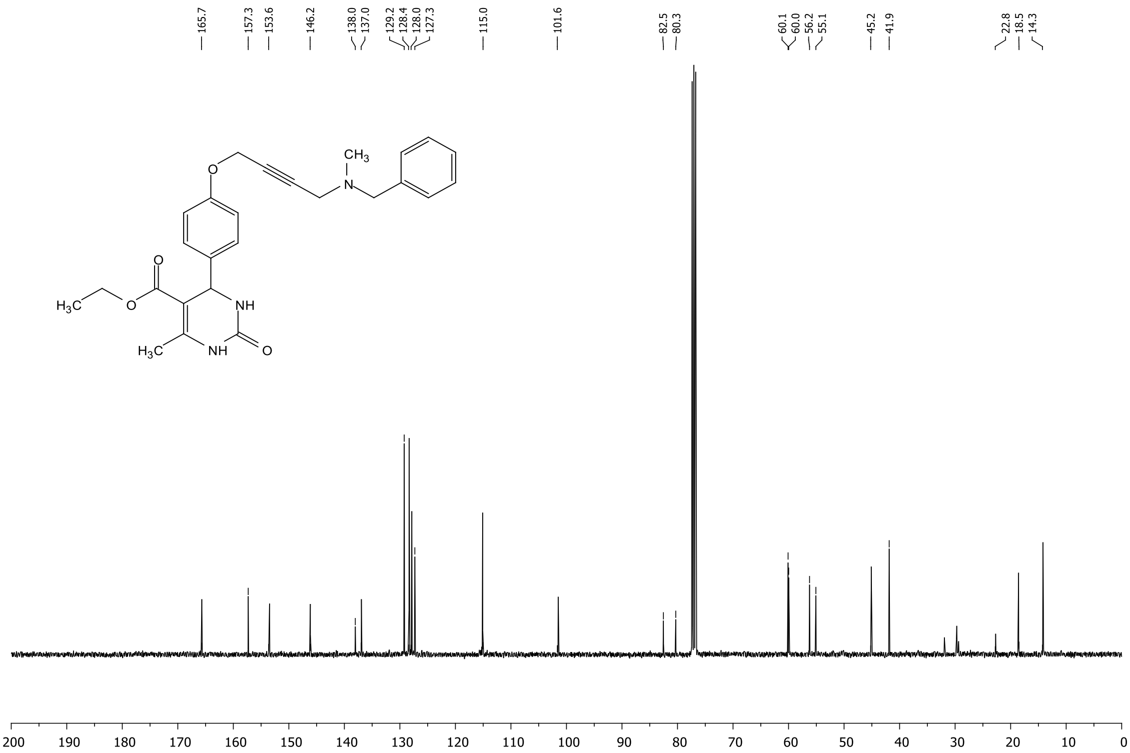
165.7
157.3
153.6
146.2
138.0
137.0
129.2
128.4
128.0
127.3
115.0
101.6
82.5
80.3
60.1
60.0
56.2
55.1
45.2
41.9
22.8
18.5
14.3
CH3
N
O
H3C
NH
NH
200
190
180
170
160
150
140
130
120
110
100
90
80
70
60
50
40
30
20
10
0

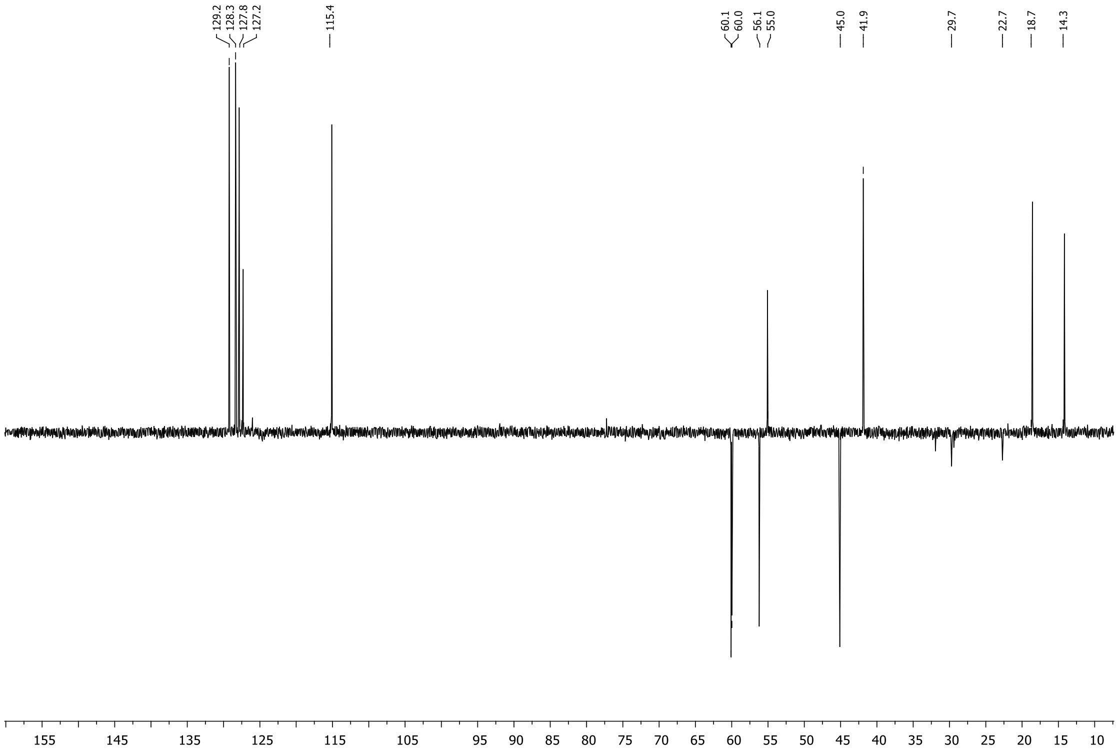
129.2
128.3
127.8
127.2
115.4
60.1
60.0
56.1
55.0
45.0
41.9
29.7
22.7
18.7
14.3
155 145 135 125 115 105 95 90 85 80 75 70 65 60 55 50 45 40 35 30 25 20 15 10

4h

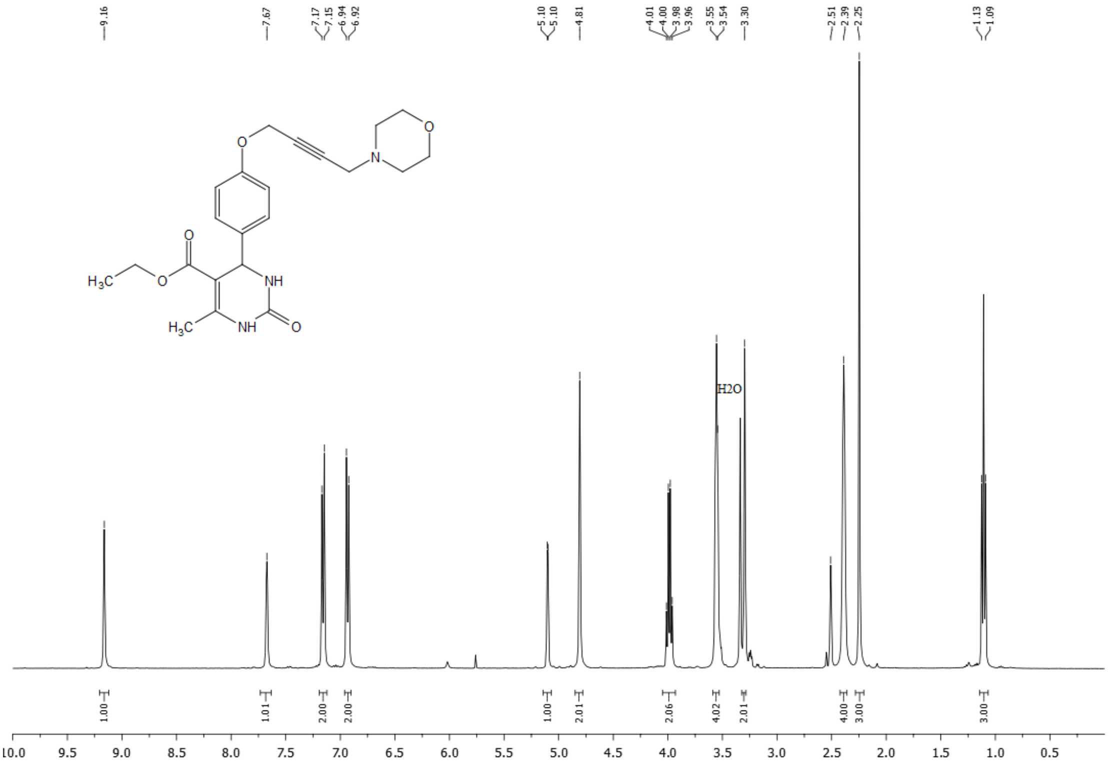
9.16
7.67
7.17
7.15
6.94
6.92
5.10
5.10
4.81
4.01
4.00
3.98
3.96
3.55
3.54
3.30
2.51
2.39
2.25
1.13
1.09
H2O
1.00
1.01
2.00
2.00
1.00
2.01
2.06
4.02
2.01
4.00
3.00
3.00
H_3C
H_3C
O
O
O
O
NH
NH
N
O

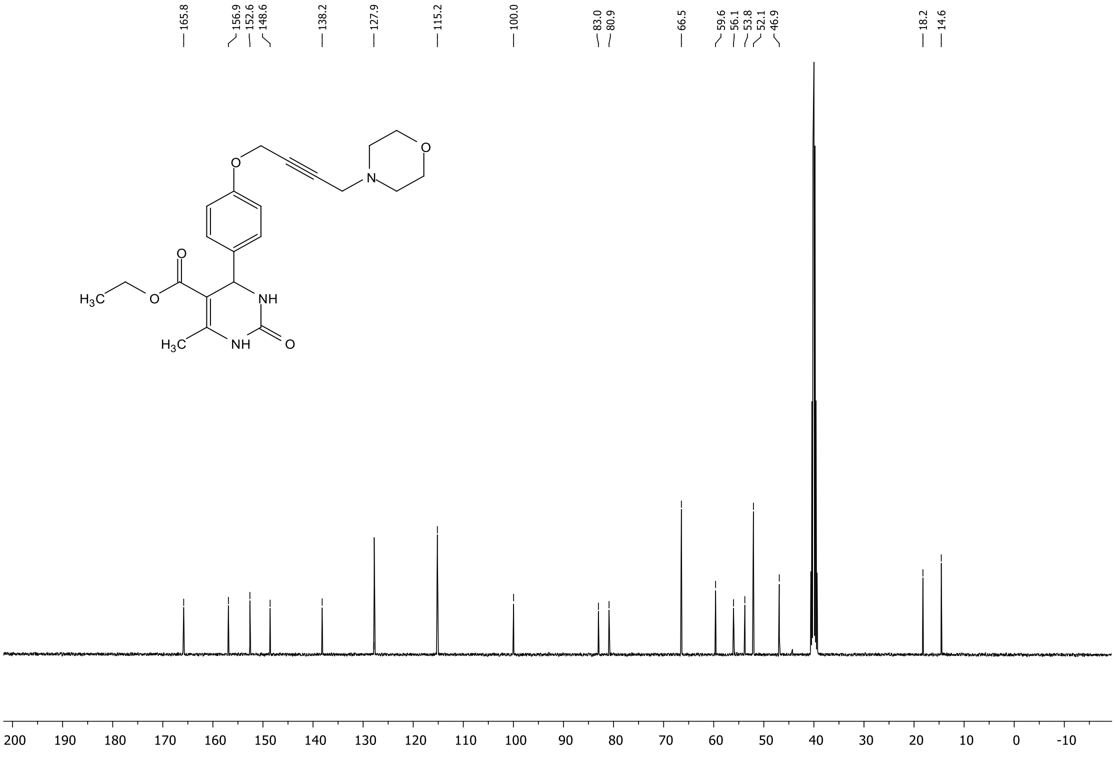
165.8
156.9
152.6
148.6
138.2
127.9
115.2
100.0
83.0
80.9
66.5
59.6
56.1
53.8
52.1
46.9
18.2
14.6
H_3C
H_3C
O
O
O
O
NH
NH
N
O

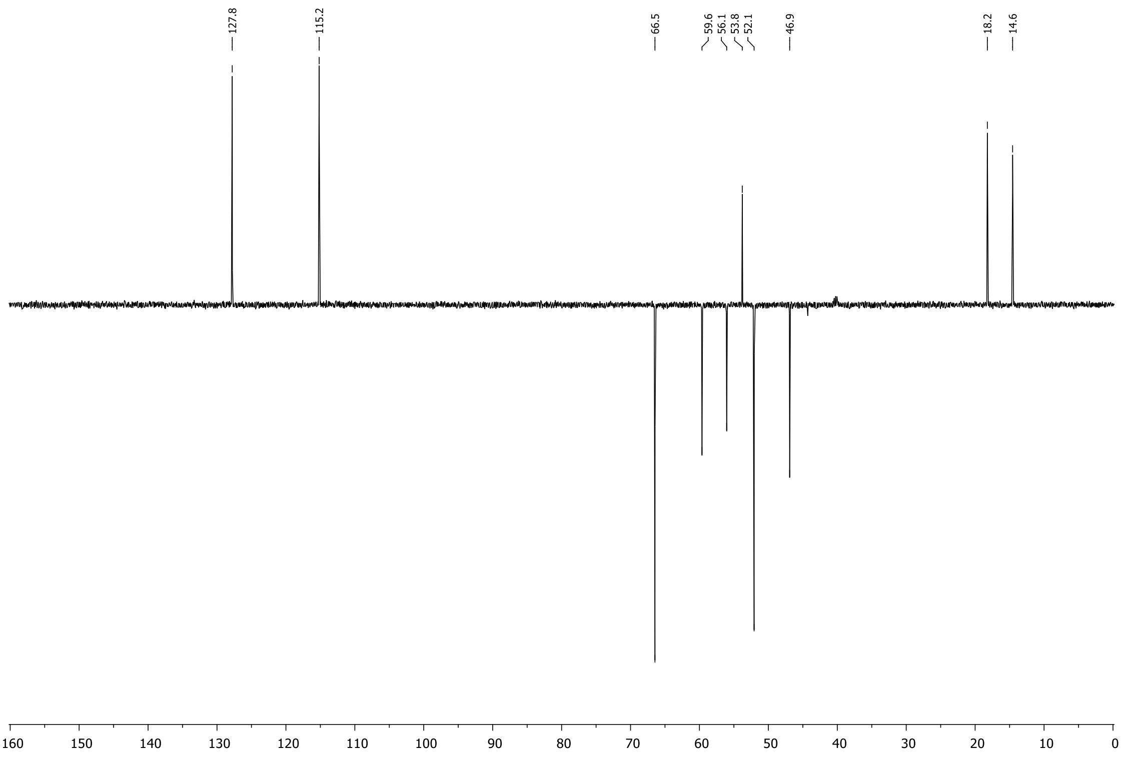
127.8
115.2
66.5
59.6
56.1
53.8
52.1
46.9
18.2
14.6
160
150
140
130
120
110
100
90
80
70
60
50
40
30
20
10
0

4i

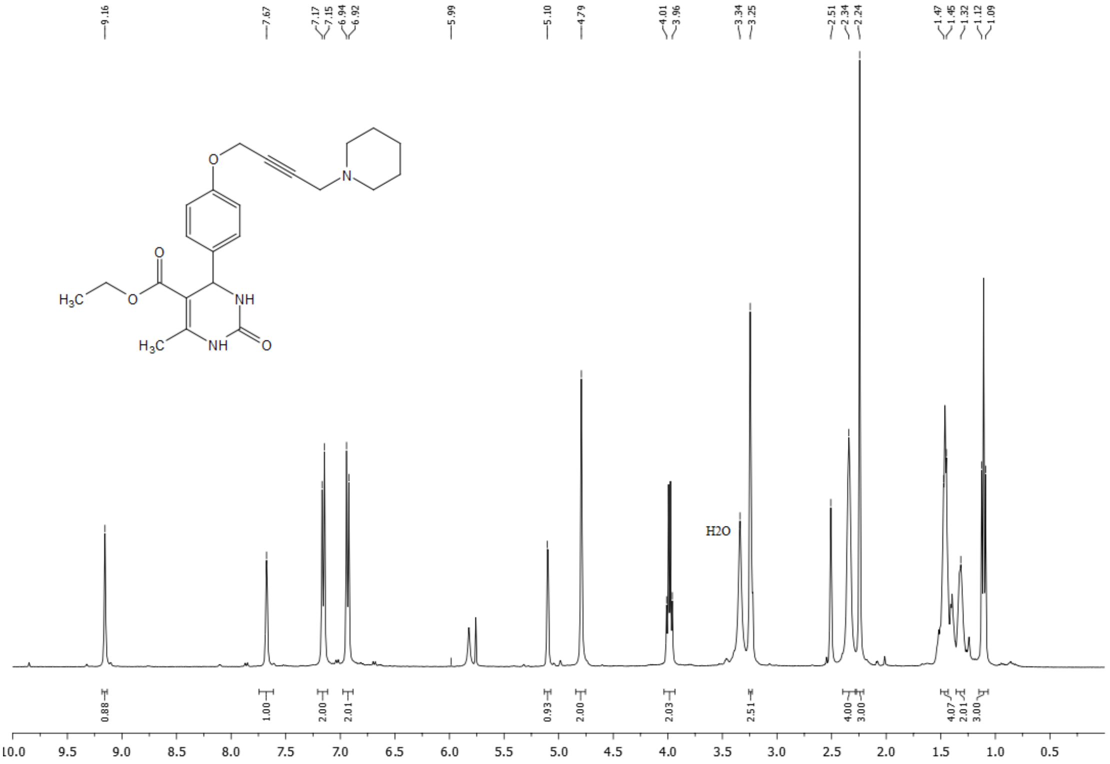
9.16
7.67
7.17
7.15
6.94
6.92
5.99
5.10
4.79
4.01
3.96
3.34
3.25
2.51
2.34
2.24
1.47
1.45
1.32
1.12
1.09
H2O
0.88
1.00
2.00
2.01
0.93
2.00
2.03
2.51
4.00
3.00
4.07
2.01
3.00
10.0 9.5 9.0 8.5 8.0 7.5 7.0 6.5 6.0 5.5 5.0 4.5 4.0 3.5 3.0 2.5 2.0 1.5 1.0 0.5

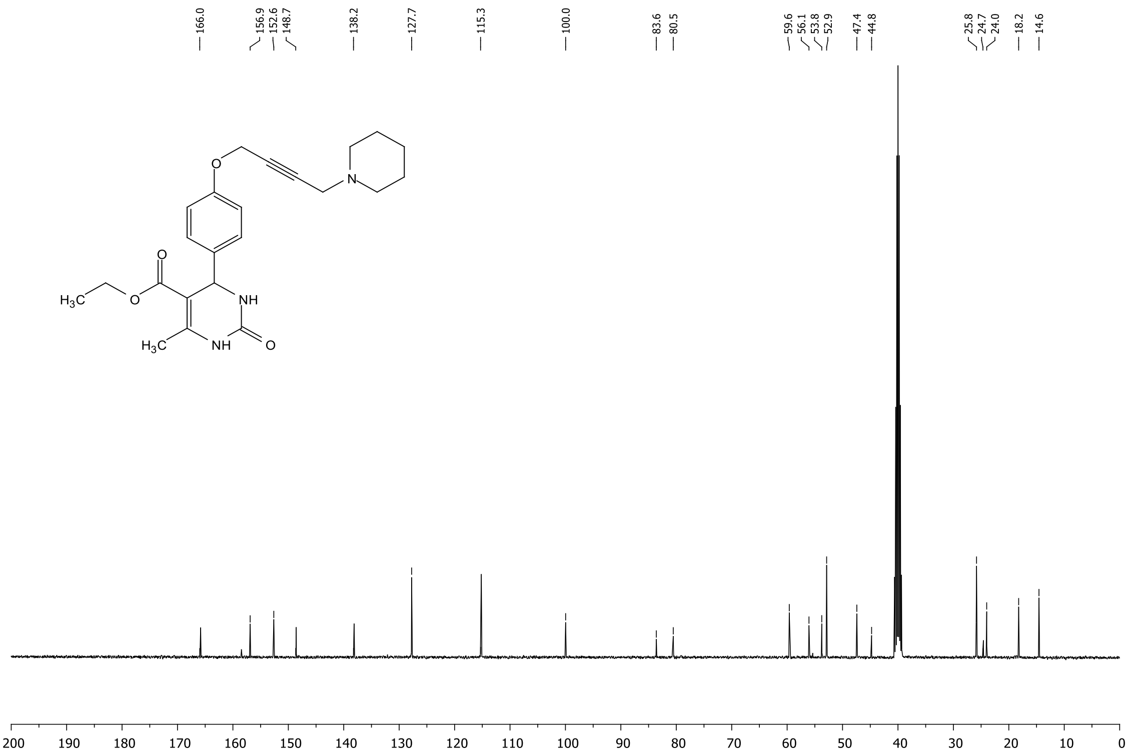
166.0
156.9
152.6
148.7
138.2
127.7
115.3
100.0
83.6
80.5
59.6
56.1
53.8
52.9
47.4
44.8
25.8
24.7
24.0
18.2
14.6
200 190 180 170 160 150 140 130 120 110 100 90 80 70 60 50 40 30 20 10 0

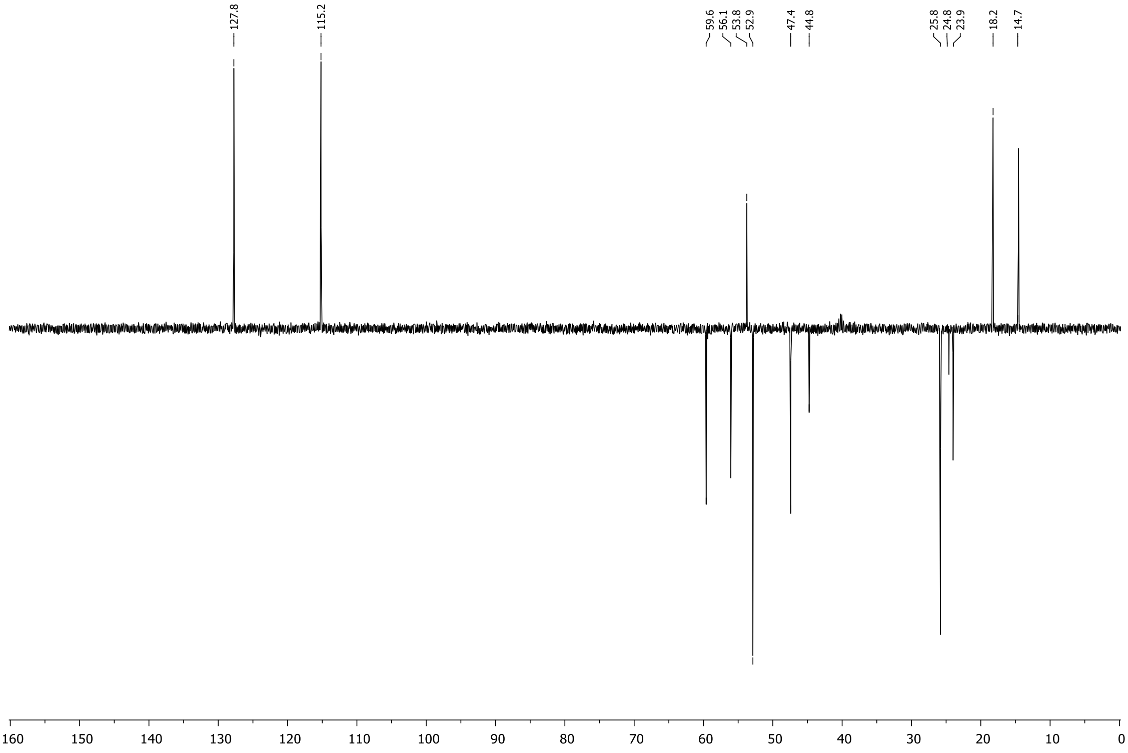
127.8
115.2
59.6
56.1
53.8
52.9
47.4
44.8
25.8
24.8
23.9
18.2
14.7
160
150
140
130
120
110
100
90
80
70
60
50
40
30
20
10
0

4j

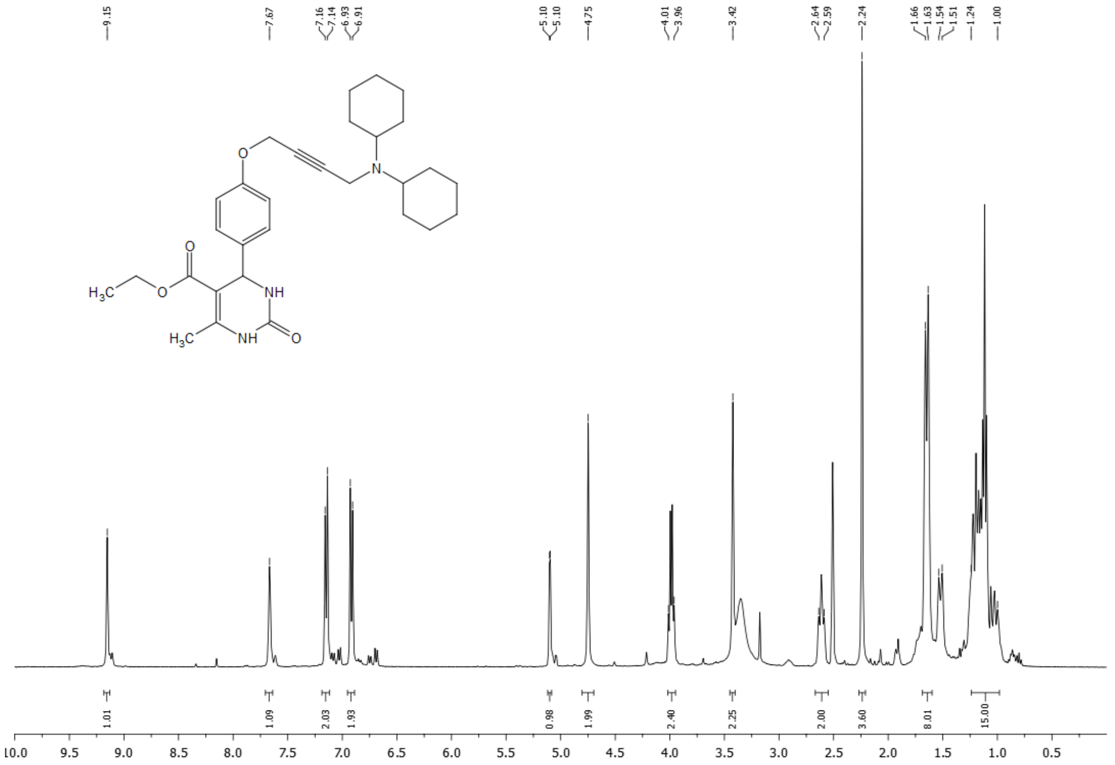
9.15
7.67
7.16
7.14
6.93
6.91
5.10
5.10
4.75
4.01
3.96
3.42
2.64
2.59
2.24
1.66
1.63
1.54
1.51
1.24
1.00
1.01
1.09
2.03
1.93
0.98
1.99
2.40
2.25
2.00
3.60
8.01
15.00

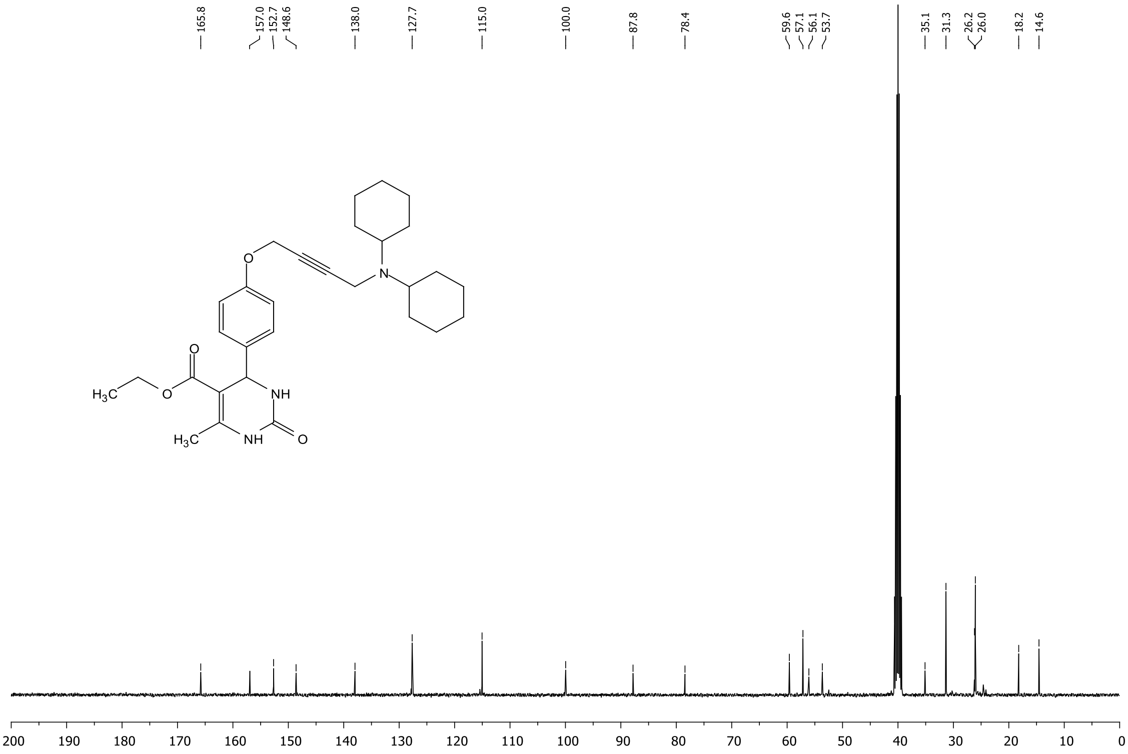
165.8
157.0
152.7
148.6
138.0
127.7
115.0
100.0
87.8
78.4
59.6
57.1
56.1
53.7
35.1
31.3
26.2
26.0
18.2
14.6

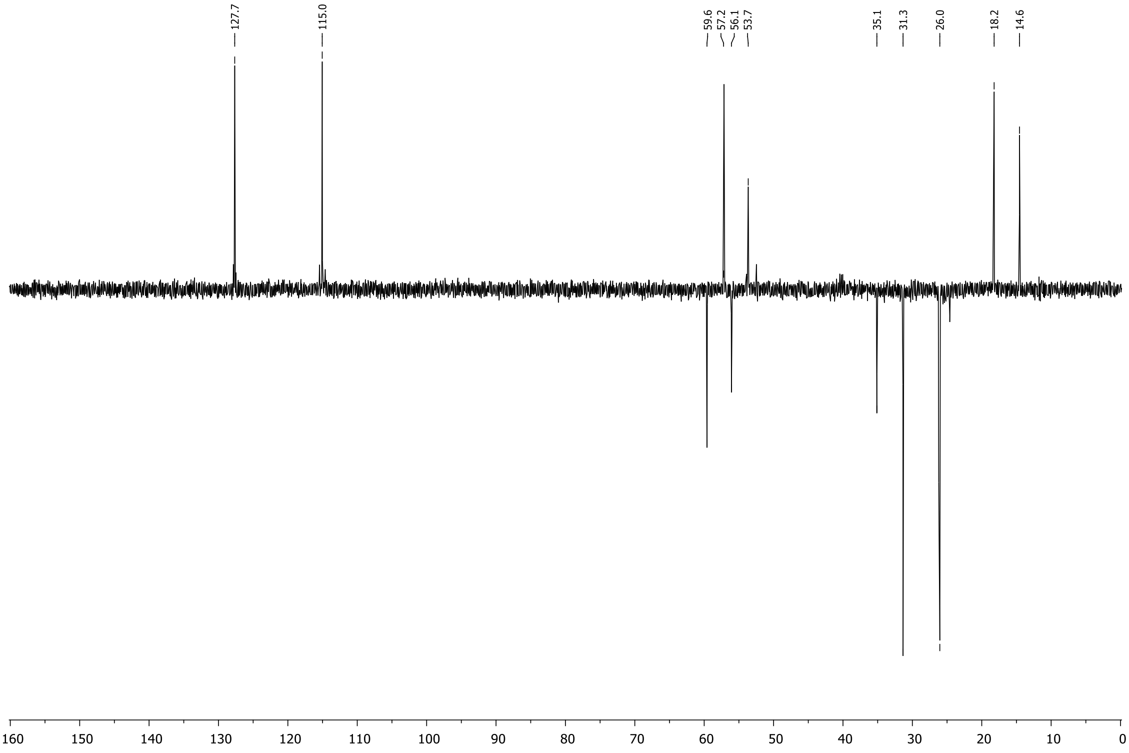
127.7
115.0
59.6
57.2
56.1
53.7
35.1
31.3
26.0
18.2
14.6
160
150
140
130
120
110
100
90
80
70
60
50
40
30
20
10
0

4k

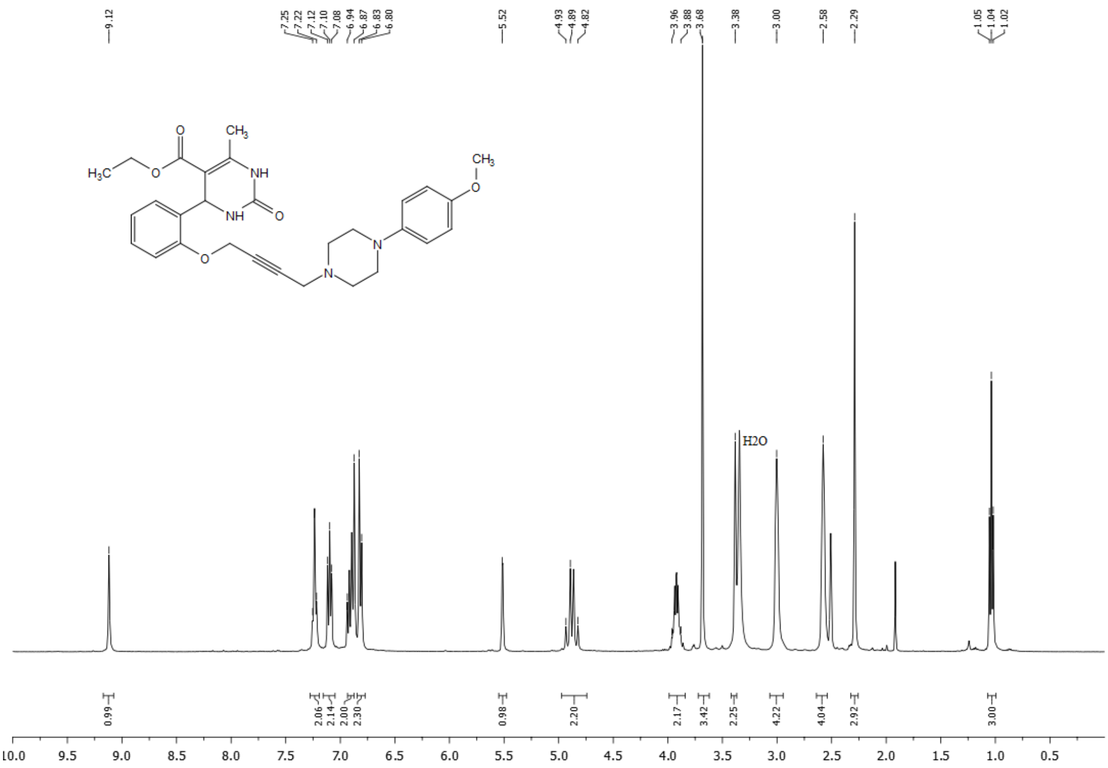

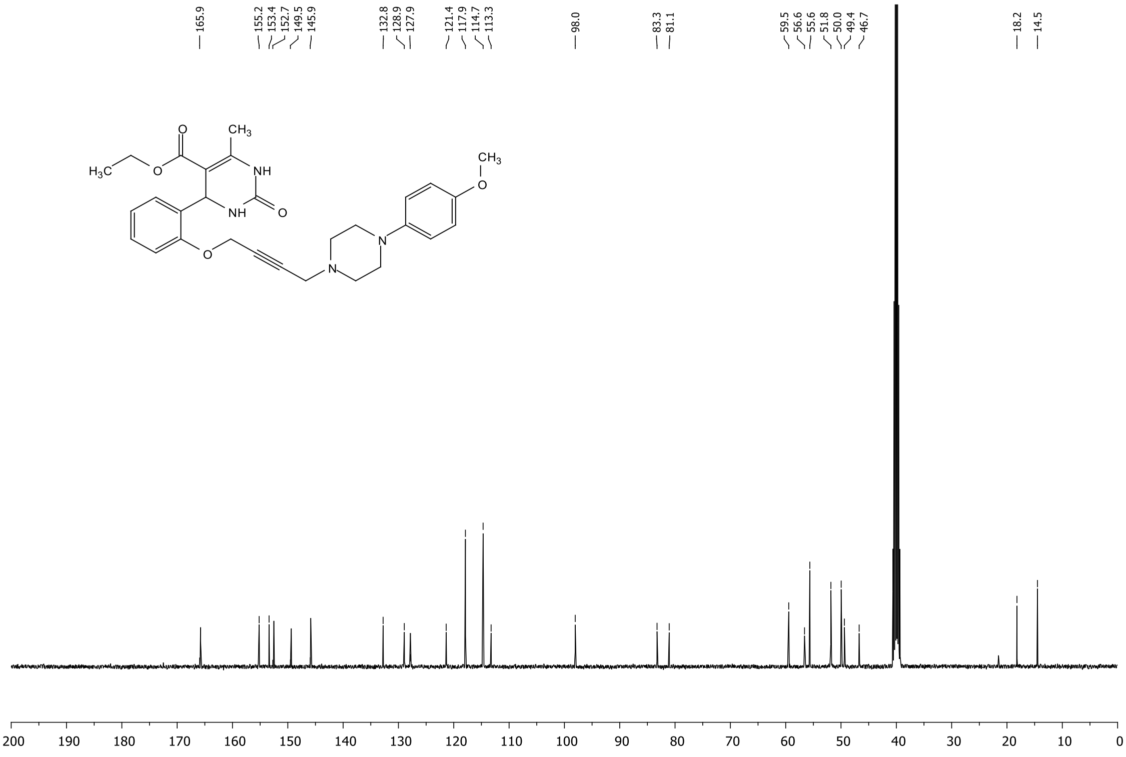

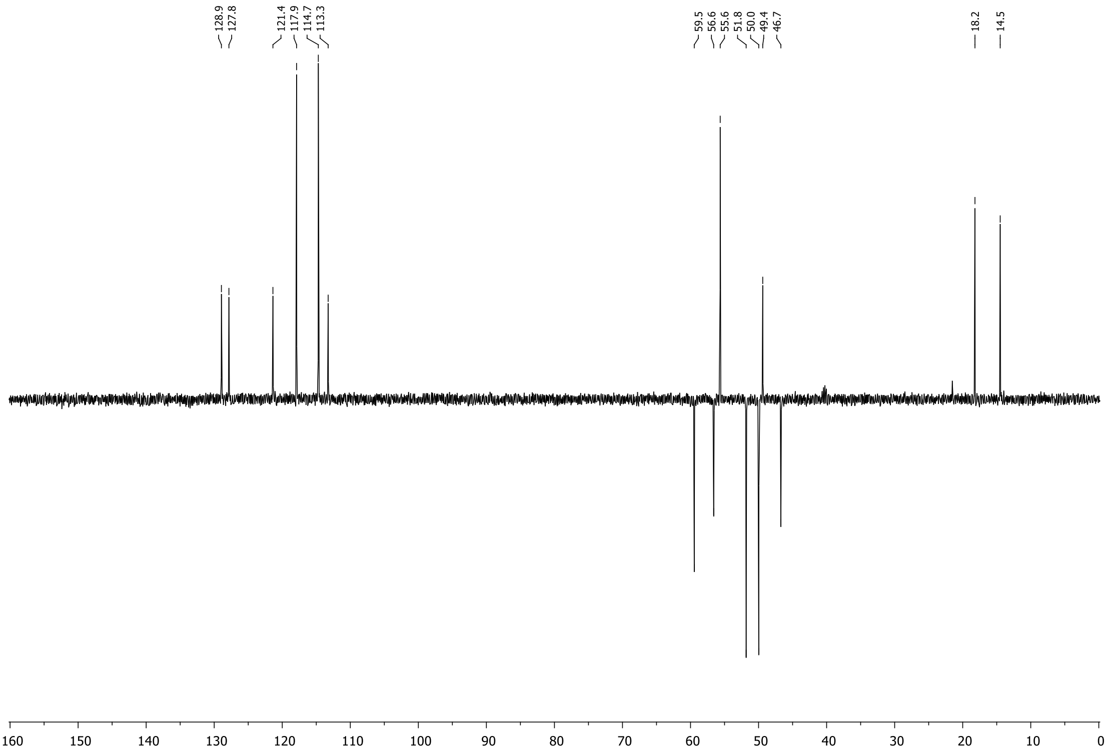
128.9
127.8
121.4
117.9
114.7
113.3
59.5
56.6
55.6
51.8
50.0
49.4
46.7
18.2
14.5
160
150
140
130
120
110
100
90
80
70
60
50
40
30
20
10
0

4l

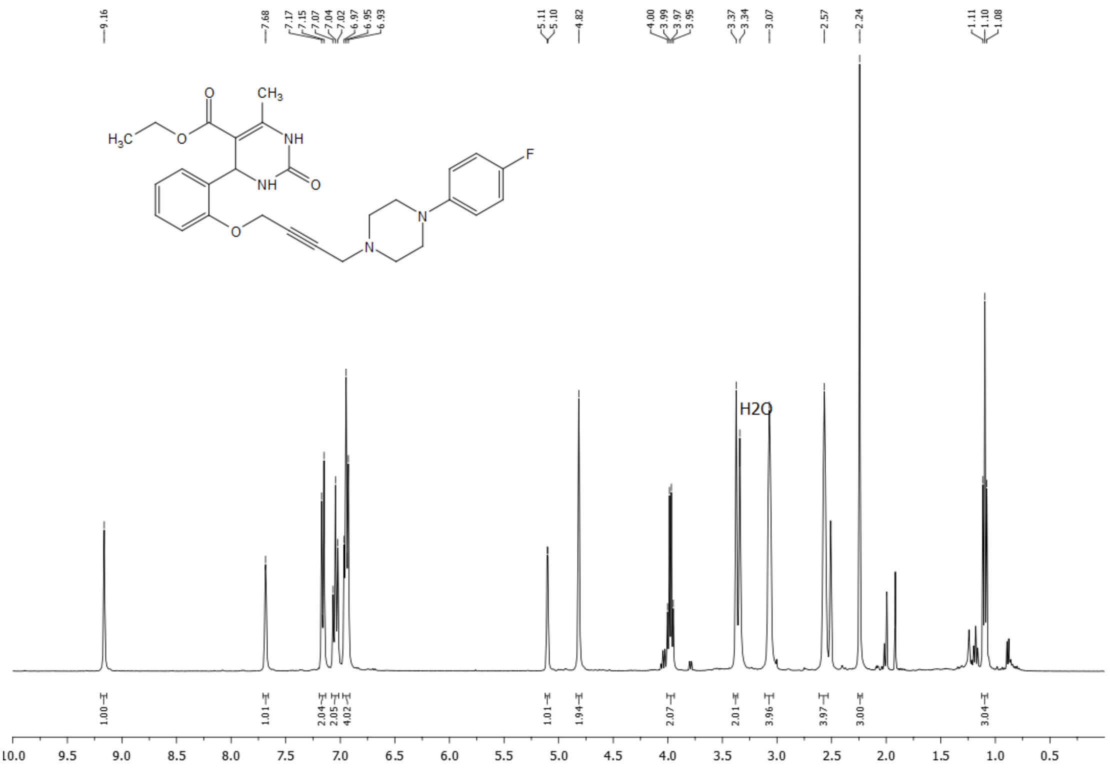

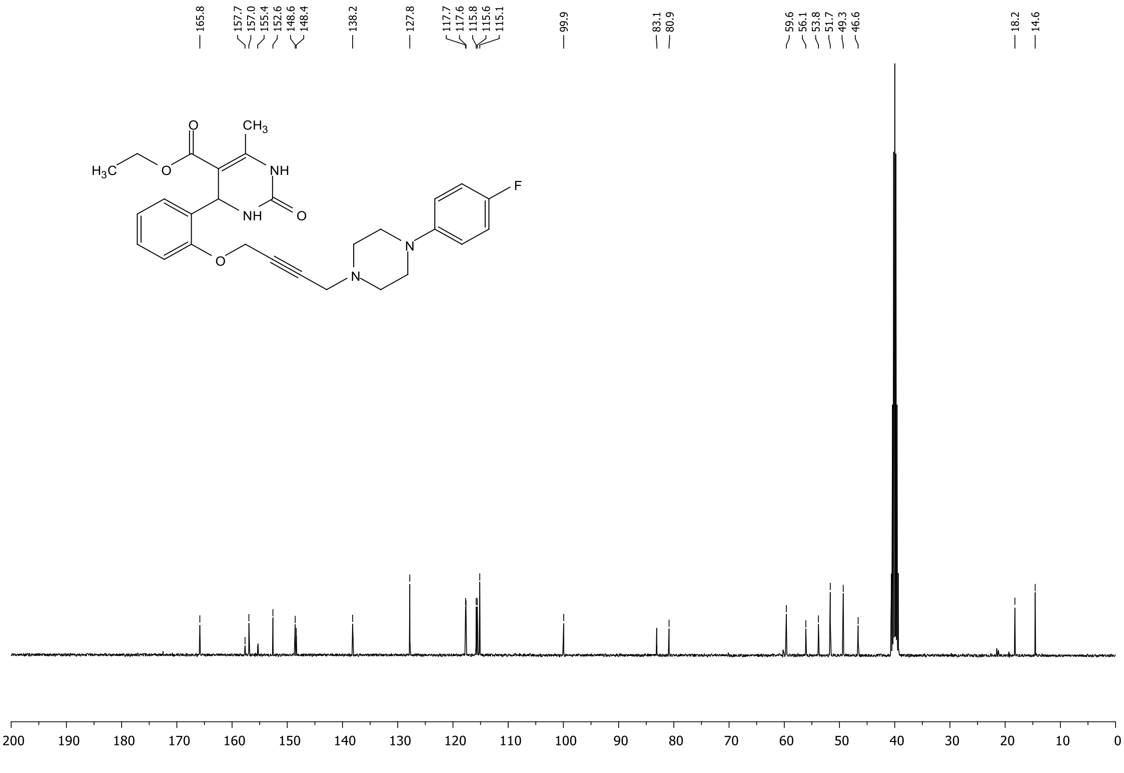

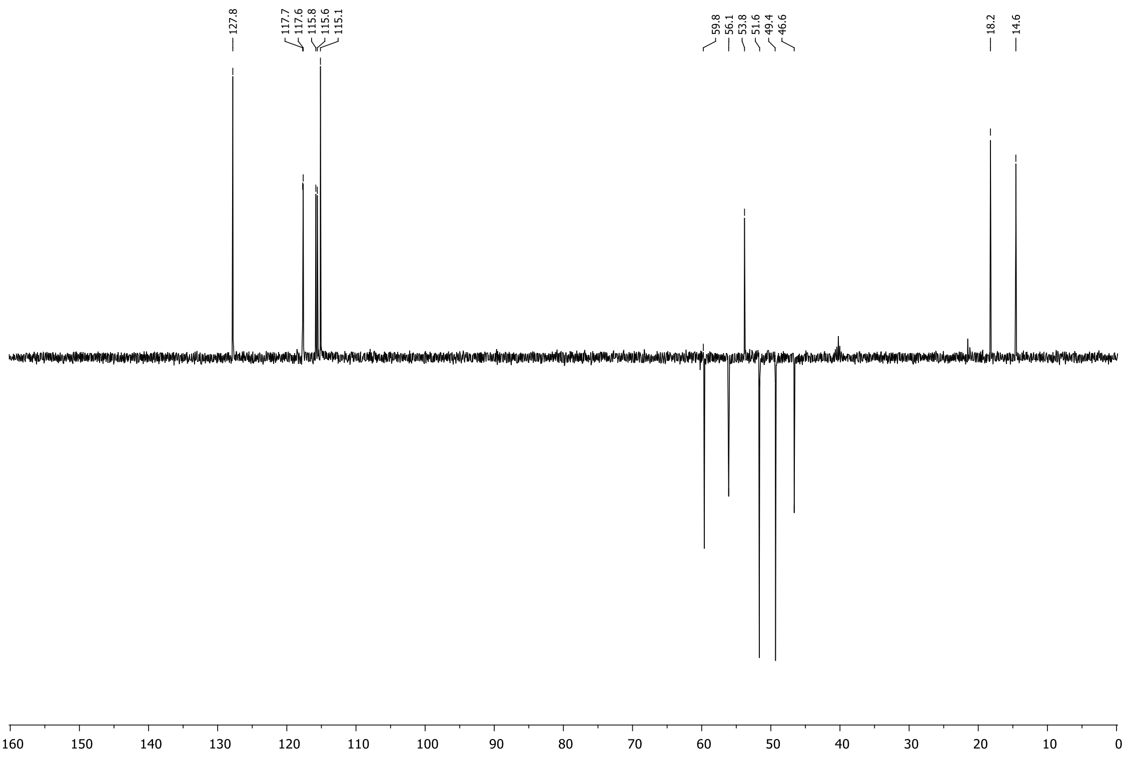
127.8
117.7
117.6
115.8
115.6
115.1
59.8
56.1
53.8
51.6
49.4
46.6
18.2
14.6
160
150
140
130
120
110
100
90
80
70
60
50
40
30
20
10
0

4m

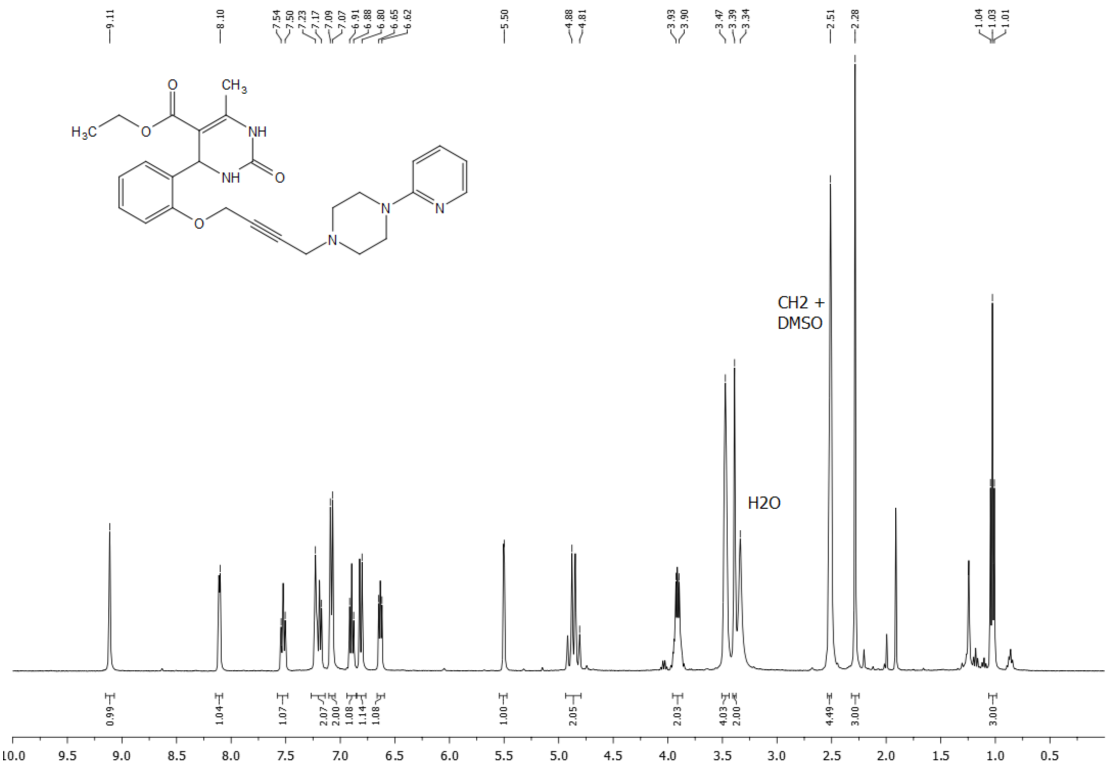

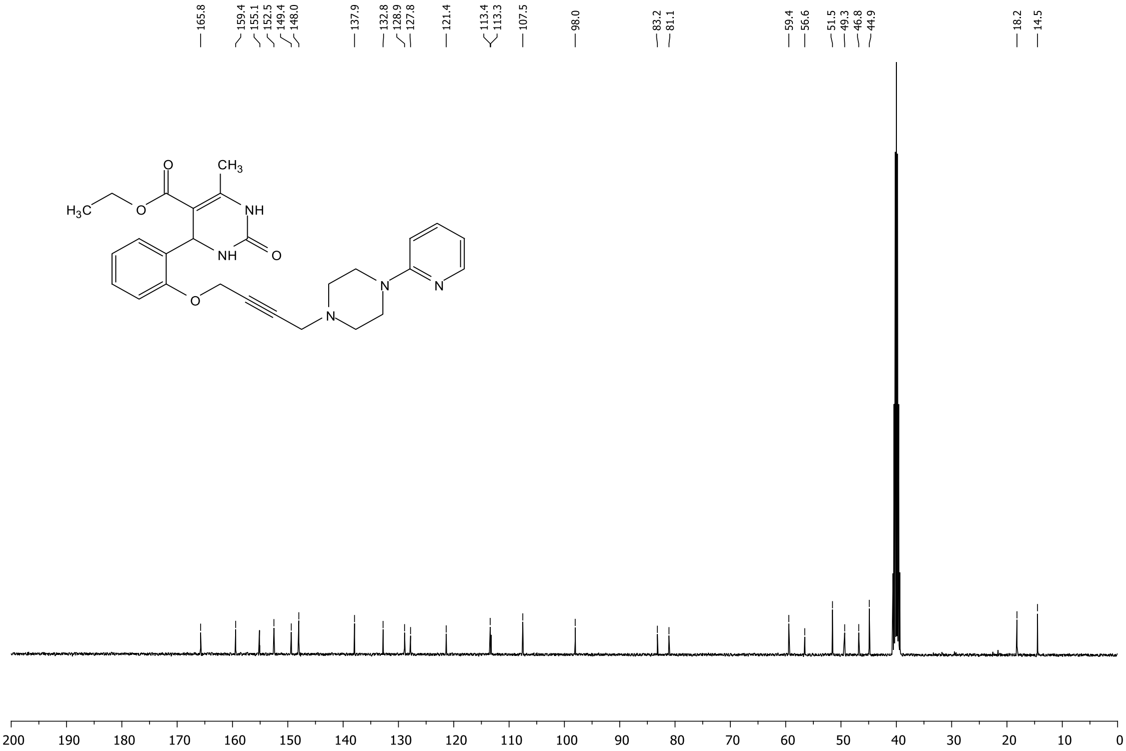

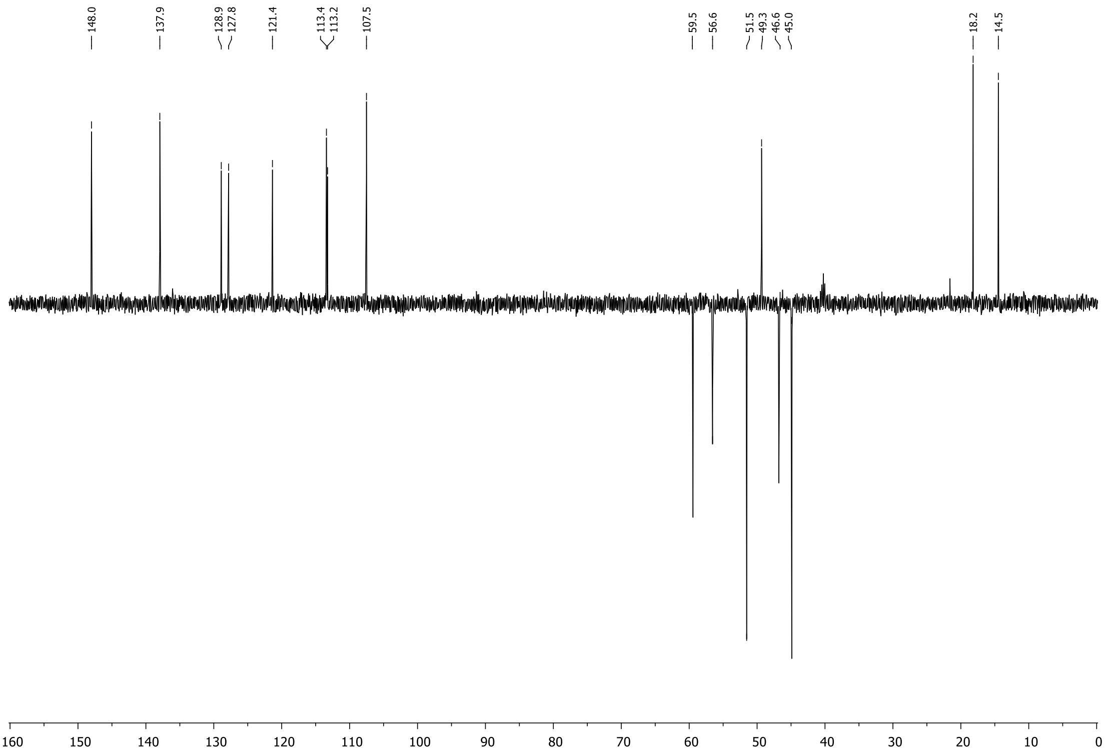

148.0
137.9
128.9
127.8
121.4
113.4
113.2
107.5
59.5
56.6
51.5
49.3
46.6
45.0
18.2
14.5
160 150 140 130 120 110 100 90 80 70 60 50 40 30 20 10 0

4n

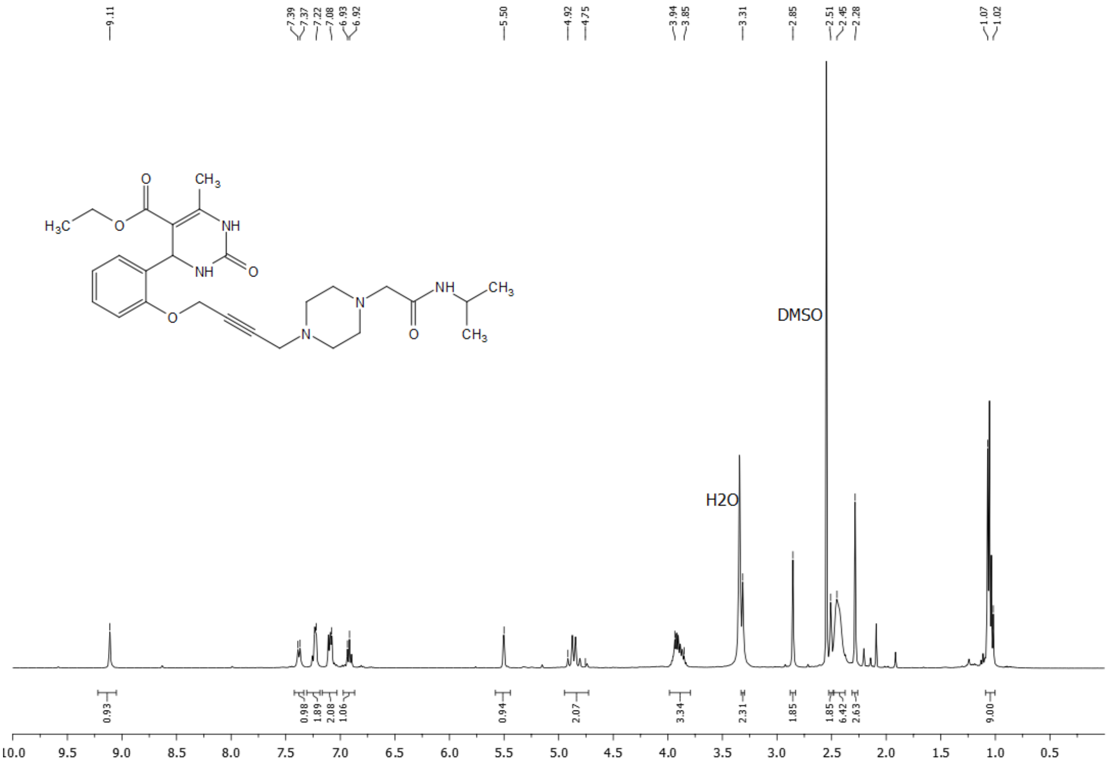

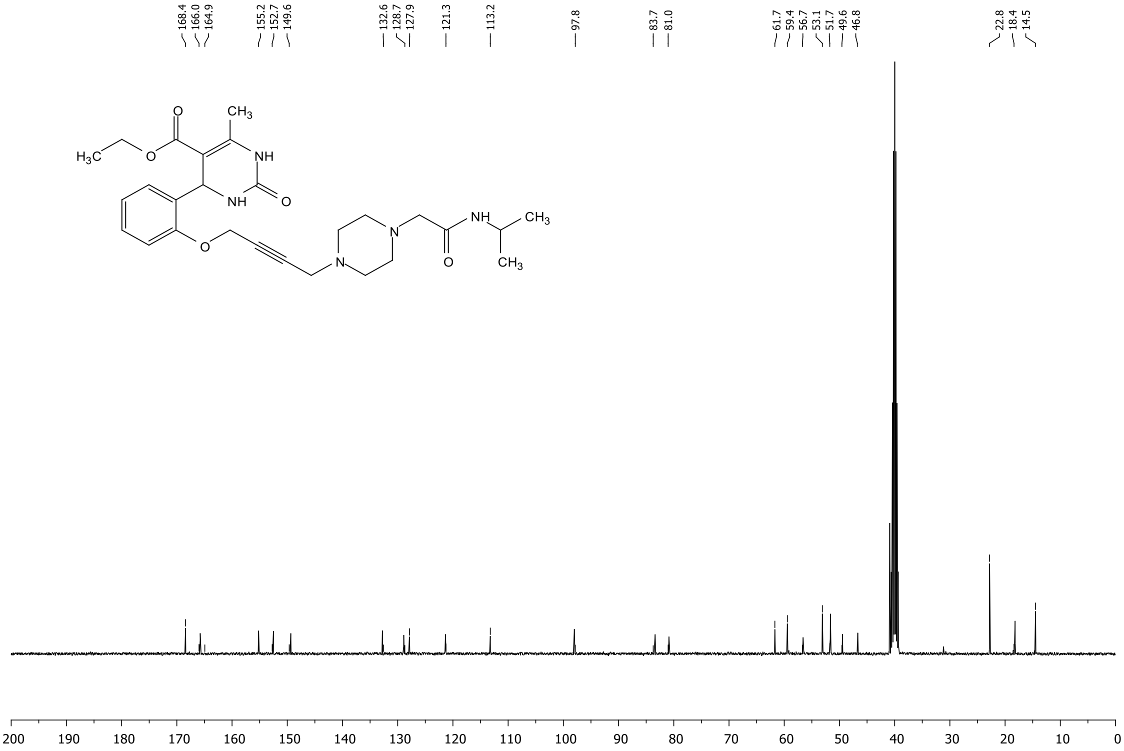

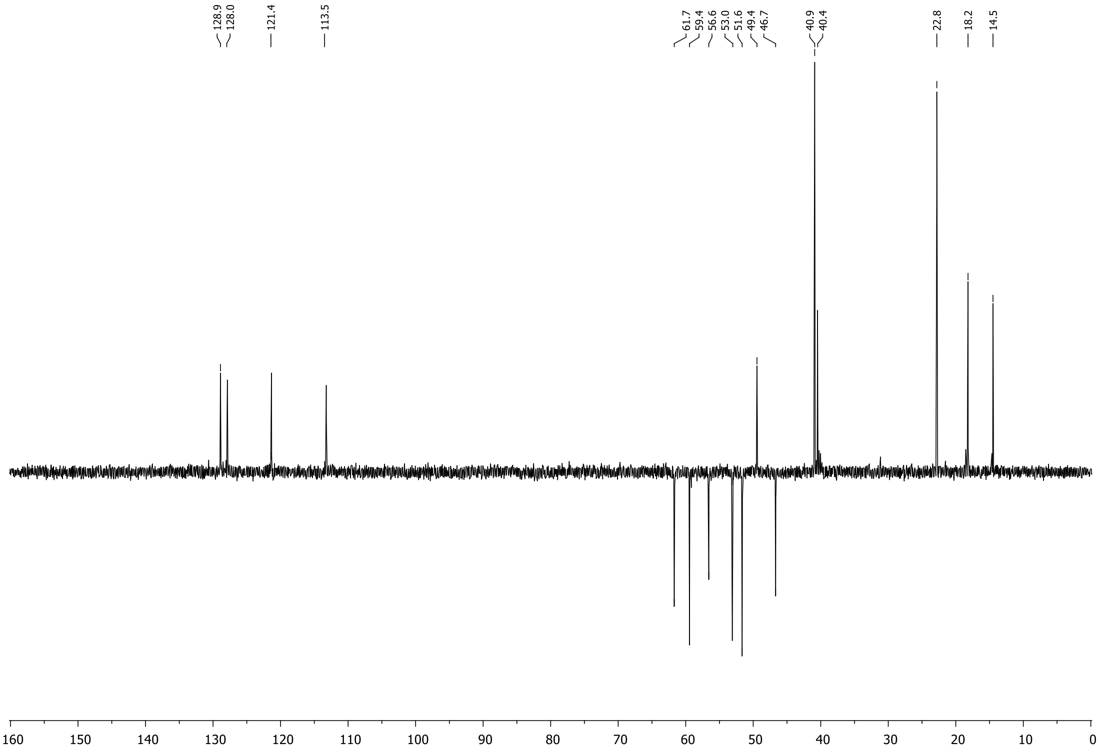
128.9
128.0
121.4
113.5
61.7
59.4
56.6
53.0
51.6
49.4
46.7
40.9
40.4
22.8
18.2
14.5
160 150 140 130 120 110 100 90 80 70 60 50 40 30 20 10 0

4o

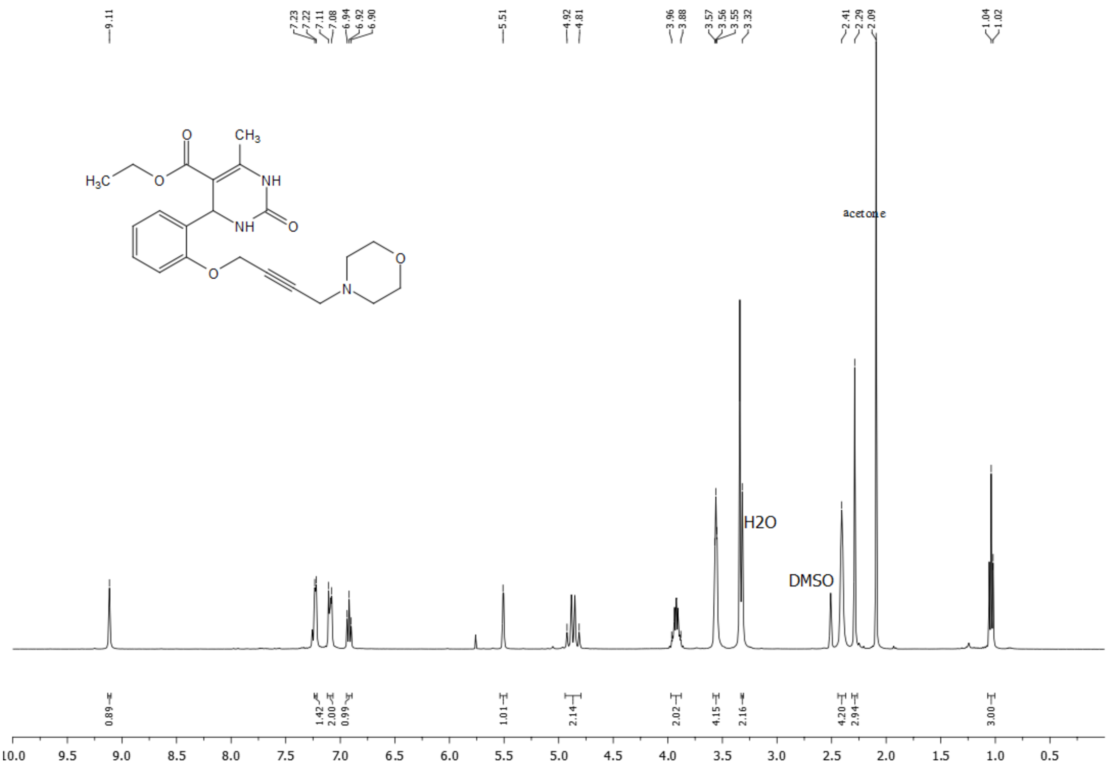
9.11
7.23
7.22
7.11
7.08
6.94
6.92
6.90
5.51
4.92
4.81
3.96
3.88
3.57
3.56
3.55
3.32
2.41
2.29
2.09
1.04
1.02
CH_3
H_3C
O
NH
acetone
H2O
DMSO
0.89
1.42
2.00
0.99
1.01
2.14
2.02
4.15
2.16
4.20
2.94
3.00
10.0 9.5 9.0 8.5 8.0 7.5 7.0 6.5 6.0 5.5 5.0 4.5 4.0 3.5 3.0 2.5 2.0 1.5 1.0 0.5

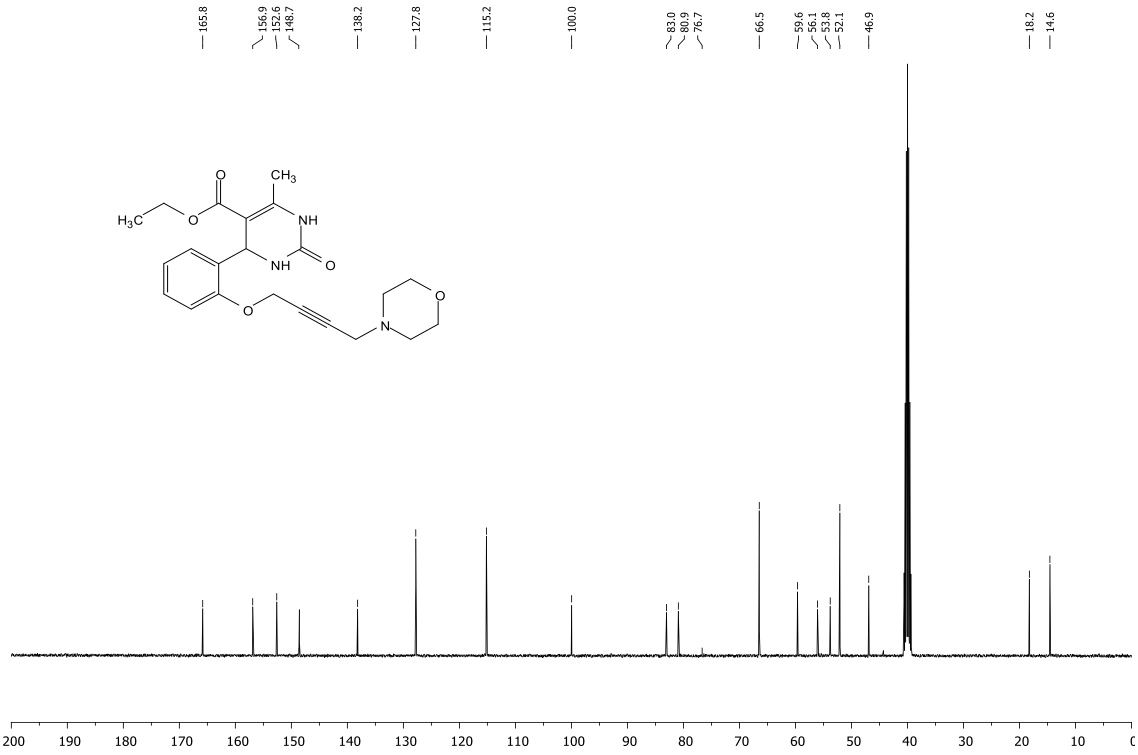
165.8
156.9
152.6
148.7
138.2
127.8
115.2
100.0
83.0
80.9
76.7
66.5
59.6
56.1
53.8
52.1
46.9
18.2
14.6
CH_3
H_3C
O
NH
N
200 190 180 170 160 150 140 130 120 110 100 90 80 70 60 50 40 30 20 10

4p

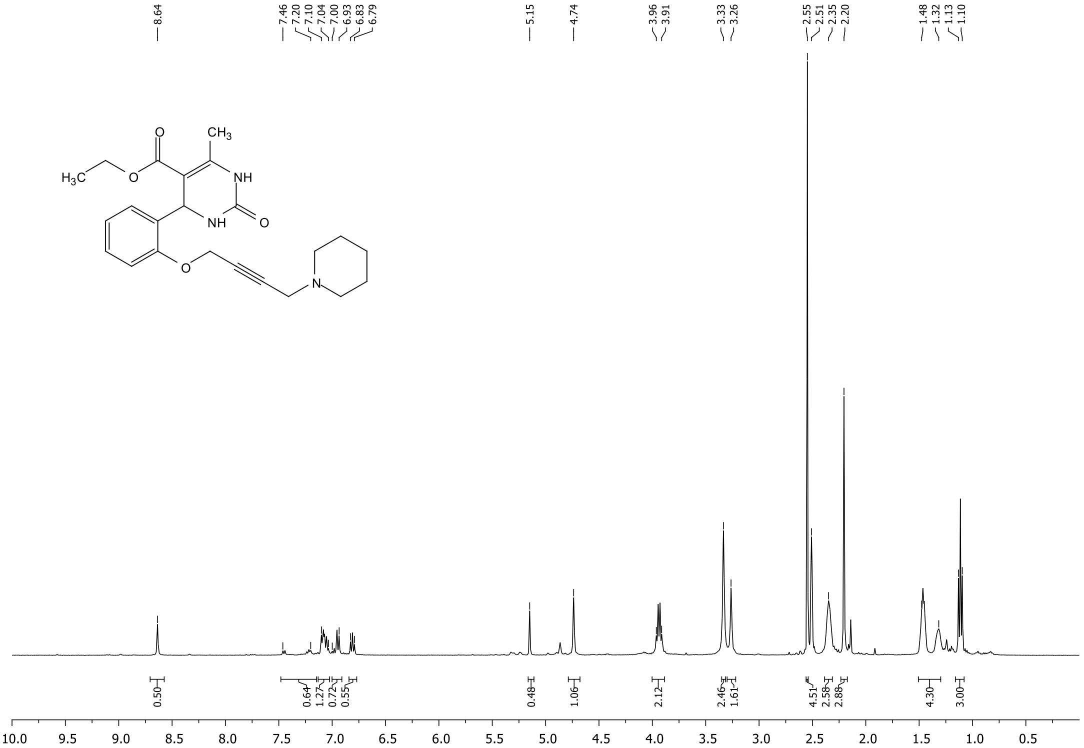

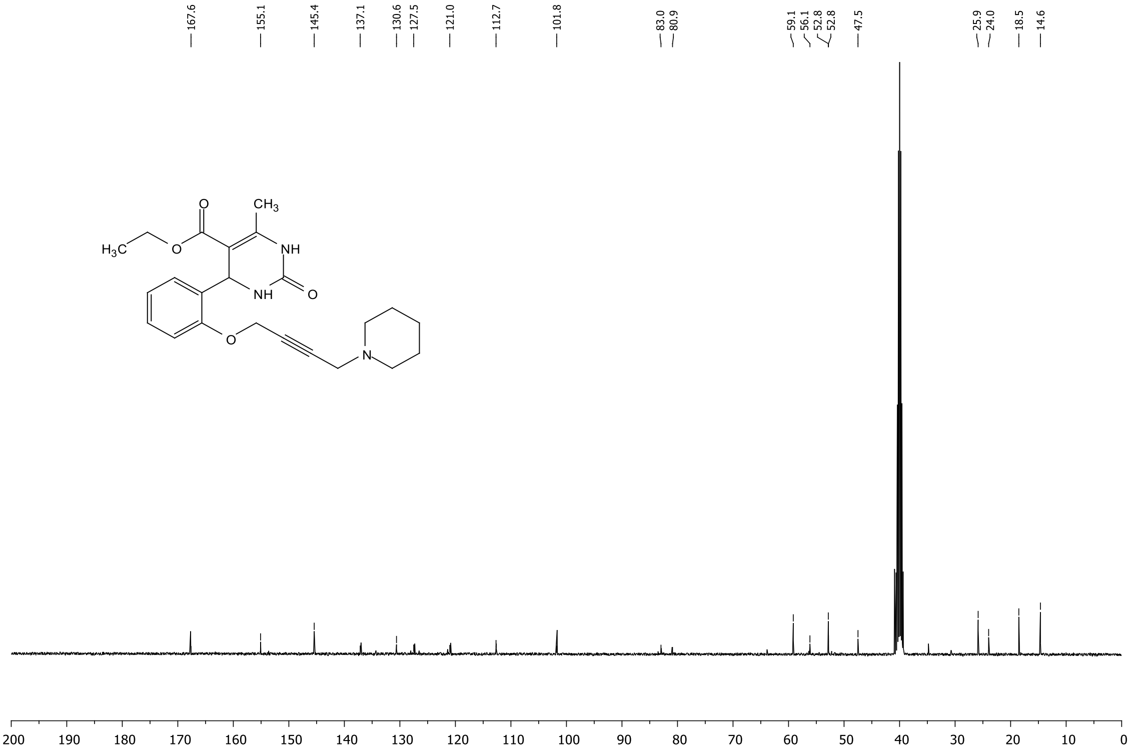

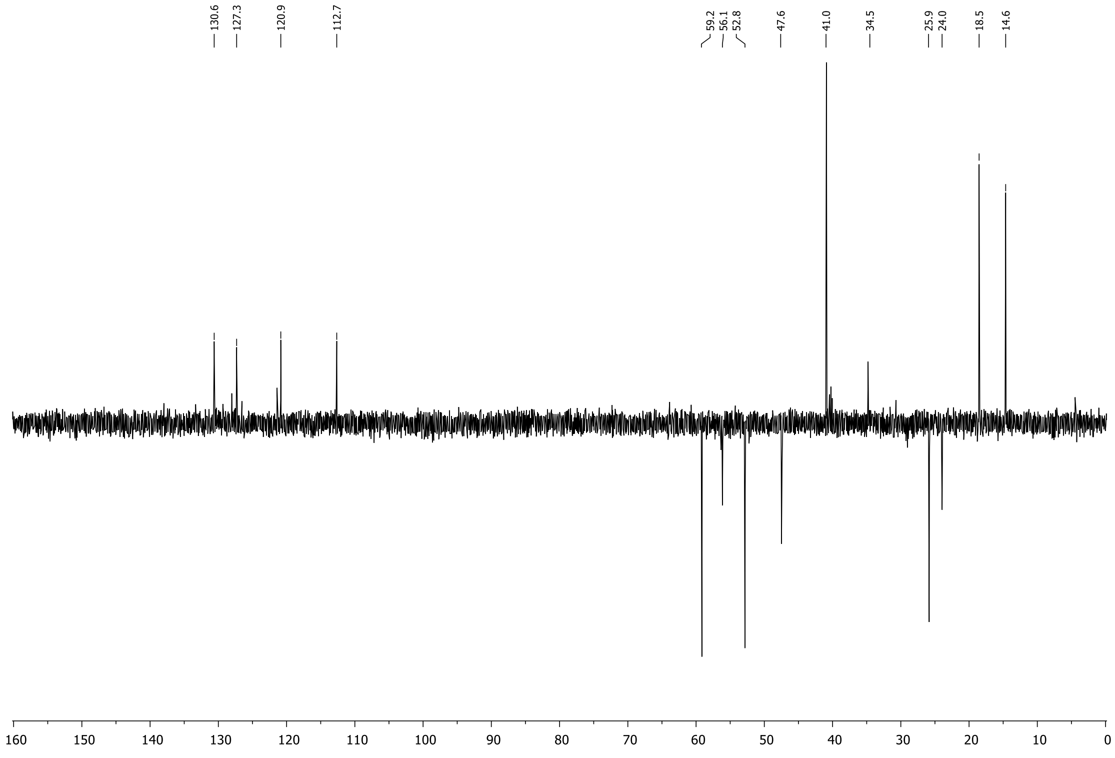
130.6
127.3
120.9
112.7
59.2
56.1
52.8
47.6
41.0
34.5
25.9
24.0
18.5
14.6
160
150
140
130
120
110
100
90
80
70
60
50
40
30
20
10
0
