# Supplementary material for: 3,4-Dihydropyrimidin-2(1H)-One Hybrids as Phenotypic Anti-Breast Cancer Agents: Synthesis, Colony-Formation Profiling and Computational Target Deconvolution
Source: Int J Mol Sci. 2026 Oct 27;27(21):9618. doi: 10.3390/ijms27219618 (PMC13763226; doi:10.3390/ijms27219618)
Supplement: Supplementary file 1 [file ijms-27-09618-s001.zip › Super-PRED_exported_data.zip!/Targets_4h.pdf]

Exported data

Target Name	ChEMBL-ID	Indication	Probability	Model accuracy
Cathepsin D	T67102	Hypertension [ICD-11: BA00-BA04]	96.02%	98.95%
Cathepsin D	T67102	Multiple sclerosis [ICD-11: 8A40]	96.02%	98.95%
Dihydrofolate reductase	T17345	Acute bacterial skin infection [ICD-11: 1C41]	94.57%	89.92%
Dihydrofolate reductase	T17345	Bacterial infection [ICD-11: 1A00-1C4Z]	94.57%	89.92%
Dihydrofolate reductase	T17345	Bladder cancer [ICD-11: 2C94]	94.57%	89.92%
Dihydrofolate reductase	T17345	Inflammatory bowel disease [ICD-11: DD72]	94.57%	89.92%
Dihydrofolate reductase	T17345	Pneumonia [ICD-11: CA40]	94.57%	89.92%
Dihydrofolate reductase	T17345	Pulmonary and extrapulmonary tuberculosis [ICD-11: 1B10.Z]	94.57%	89.92%
Dihydrofolate reductase	T17345	Rheumatoid arthritis [ICD-11: FA20]	94.57%	89.92%
Dihydrofolate reductase	T17345	Solid tumour/cancer [ICD-11: 2A00-2F9Z]	94.57%	89.92%
Dihydrofolate reductase	T17345	Urinary tract infection [ICD-11: GC08]	94.57%	89.92%
TGF-beta receptor type II	T49989	Solid tumour/cancer [ICD-11: 2A00-2F9Z]	90.26%	88.18%
TGF-beta receptor type II	T49989	Wound healing [ICD-11: EL8Y]	90.26%	88.18%
DNA topoisomerase II alpha	T17048	Solid tumour/cancer [ICD-11: 2A00-2F9Z]	83.72%	89%
G-protein coupled bile acid receptor 1	T86273	Metabolic disorder [ICD-11: 5C50-5D2Z]	83.62%	93.65%
G-protein coupled bile acid receptor 1	T86273	Type-2 diabetes [ICD-11: 5A11]	83.62%	93.65%
Sodium/hydrogen exchanger 1	T82028	Angina pectoris [ICD-11: BA40]	83.45%	90.24%
Sodium/hydrogen exchanger 1	T82028	Cardiac arrhythmias [ICD-11: BC9Z]	83.45%	90.24%
Sodium/hydrogen exchanger 1	T82028	Heart arrhythmia [ICD-11: BC65]	83.45%	90.24%
Sodium/hydrogen exchanger 1	T82028	Myocardial infarction [ICD-11: BA41-BA43]	83.45%	90.24%
Histone deacetylase 10	T94324	Solid tumour/cancer [ICD-11: 2A00-2F9Z]	82.61%	90.08%
Beta-glucuronidase	T96413	Mucopolysaccharidosis [ICD-11: 5C56.3]	82.19%	77.68%
Beta-glucuronidase	T96413	Periodontal disease [ICD-11: DA0C]	82.19%	77.68%
Casein kinase II alpha/beta	T51565	Cholangiocarcinoma [ICD-11: 2C12.10]	79.81%	99.23%
Casein kinase II alpha/beta	T51565	Solid tumour/cancer [ICD-11: 2A00-2F9Z]	79.81%	99.23%
Adenosine A2b receptor	T86679	Acute and chronic heart failure [ICD-11: BD1Z]	79.61%	98.59%
Adenosine A2b receptor	T86679	Alzheimer disease [ICD-11: 8A20]	79.61%	98.59%
Adenosine A2b receptor	T86679	Apnea [ICD-11: MD11.0]	79.61%	98.59%
Adenosine A2b receptor	T86679	Asthma [ICD-11: CA23]	79.61%	98.59%
Adenosine A2b receptor	T86679	Herpes simplex virus infection [ICD-11: 1F00]	79.61%	98.59%
Adenosine A2b receptor	T86679	Hypertension [ICD-11: BA00-BA04]	79.61%	98.59%
Adenosine A2b receptor	T86679	Multiple sclerosis [ICD-11: 8A40]	79.61%	98.59%

Target Name	ChEMBL-ID	Indication	Probability	Model accuracy
Adenosine A2b receptor	T86679	Non-insulin dependent diabetes [ICD-11: 5A11]	79.61%	98.59%
Adenosine A2b receptor	T86679	Non-small-cell lung cancer [ICD-11: 2C25.Y]	79.61%	98.59%
Adenosine A2b receptor	T86679	Parkinson disease [ICD-11: 8A00.0]	79.61%	98.59%
Adenosine A2b receptor	T86679	Paroxysmal supraventricular tachycardia [ICD-11: BC81.Z]	79.61%	98.59%
Adenosine A2b receptor	T86679	Solid tumour/cancer [ICD-11: 2A00-2F9Z]	79.61%	98.59%
PI3-kinase p110-alpha/p85-alpha	T80276	Breast cancer [ICD-11: 2C60-2C65]	79.6%	94.33%
PI3-kinase p110-alpha/p85-alpha	T80276	Follicular lymphoma [ICD-11: 2A80]	79.6%	94.33%
PI3-kinase p110-alpha/p85-alpha	T80276	Non-hodgkin lymphoma [ICD-11: 2B33.5]	79.6%	94.33%
PI3-kinase p110-alpha/p85-alpha	T80276	Prostate cancer [ICD-11: 2C82.0]	79.6%	94.33%
PI3-kinase p110-alpha/p85-alpha	T80276	Solid tumour/cancer [ICD-11: 2A00-2F9Z]	79.6%	94.33%
GABA-A receptor; alpha-1/beta-2/gamma-2	T08910	Alcohol dependence [ICD-11: 6C40.2]	78.51%	92.67%
GABA-A receptor; alpha-1/beta-2/gamma-2	T08910	Anaesthesia [ICD-11: 9A78.6]	78.51%	92.67%
GABA-A receptor; alpha-1/beta-2/gamma-2	T08910	Anxiety disorder [ICD-11: 6B00-6B0Z]	78.51%	92.67%
GABA-A receptor; alpha-1/beta-2/gamma-2	T08910	Anxiety disorder [ICD-11: 6B00-6B0Z]	78.51%	92.67%
GABA-A receptor; alpha-1/beta-2/gamma-2	T08910	Anxiety disorder [ICD-11: 6B00-6B0Z]	78.51%	92.67%
GABA-A receptor; alpha-1/beta-2/gamma-2	T08910	Chronic pain [ICD-11: MG30]	78.51%	92.67%
GABA-A receptor; alpha-1/beta-2/gamma-2	T08910	Cystitis [ICD-11: GC00]	78.51%	92.67%
GABA-A receptor; alpha-1/beta-2/gamma-2	T08910	Depression [ICD-11: 6A70-6A7Z]	78.51%	92.67%
GABA-A receptor; alpha-1/beta-2/gamma-2	T08910	Depression [ICD-11: 6A70-6A7Z]	78.51%	92.67%
GABA-A receptor; alpha-1/beta-2/gamma-2	T08910	Depression [ICD-11: 6A70-6A7Z]	78.51%	92.67%
GABA-A receptor; alpha-1/beta-2/gamma-2	T08910	Epilepsy [ICD-11: 8A60-8A68]	78.51%	92.67%
GABA-A receptor; alpha-1/beta-2/gamma-2	T08910	Epileptic seizures [ICD-11: 8A61-8A6Z]	78.51%	92.67%
GABA-A receptor; alpha-1/beta-2/gamma-2	T08910	Epileptic seizures [ICD-11: 8A61-8A6Z]	78.51%	92.67%
GABA-A receptor; alpha-1/beta-2/gamma-2	T08910	Epileptic seizures [ICD-11: 8A61-8A6Z]	78.51%	92.67%
GABA-A receptor; alpha-1/beta-2/gamma-2	T08910	Essential tremor or related tremors [ICD-11: 8A04.1]	78.51%	92.67%
GABA-A receptor; alpha-1/beta-2/gamma-2	T08910	Essential tremor or related tremors [ICD-11: 8A04.1]	78.51%	92.67%
GABA-A receptor; alpha-1/beta-2/gamma-2	T08910	Essential tremor or related tremors [ICD-11: 8A04.1]	78.51%	92.67%
GABA-A receptor; alpha-1/beta-2/gamma-2	T08910	Fibromyalgia [ICD-11: MG30.01]	78.51%	92.67%

Target Name	ChEMBL-ID	Indication	Probability	Model accuracy
GABA-A receptor; alpha-1/beta-2/gamma-2	T08910	Headache [ICD-11: 8A80-8A84]	78.51%	92.67%
GABA-A receptor; alpha-1/beta-2/gamma-2	T08910	Inflammation [ICD-11: 1A00-CA43.1]	78.51%	92.67%
GABA-A receptor; alpha-1/beta-2/gamma-2	T08910	Inflammation [ICD-11: 1A00-CA43.1]	78.51%	92.67%
GABA-A receptor; alpha-1/beta-2/gamma-2	T08910	Inflammation [ICD-11: 1A00-CA43.1]	78.51%	92.67%
GABA-A receptor; alpha-1/beta-2/gamma-2	T08910	Inflammatory bowel disease [ICD-11: DD72]	78.51%	92.67%
GABA-A receptor; alpha-1/beta-2/gamma-2	T08910	Inflammatory bowel disease [ICD-11: DD72]	78.51%	92.67%
GABA-A receptor; alpha-1/beta-2/gamma-2	T08910	Inflammatory bowel disease [ICD-11: DD72]	78.51%	92.67%
GABA-A receptor; alpha-1/beta-2/gamma-2	T08910	Insomnia [ICD-11: 7A00-7A0Z]	78.51%	92.67%
GABA-A receptor; alpha-1/beta-2/gamma-2	T08910	Insomnia [ICD-11: 7A00-7A0Z]	78.51%	92.67%
GABA-A receptor; alpha-1/beta-2/gamma-2	T08910	Intractable insomnia [ICD-11: 7A00]	78.51%	92.67%
GABA-A receptor; alpha-1/beta-2/gamma-2	T08910	Ischemic reperfusion injury [ICD-11: DB98.B]	78.51%	92.67%
GABA-A receptor; alpha-1/beta-2/gamma-2	T08910	Malaria [ICD-11: 1F40-1F45]	78.51%	92.67%
GABA-A receptor; alpha-1/beta-2/gamma-2	T08910	Muscle spasm [ICD-11: MB47.3]	78.51%	92.67%
GABA-A receptor; alpha-1/beta-2/gamma-2	T08910	Muscle spasm [ICD-11: MB47.3]	78.51%	92.67%
GABA-A receptor; alpha-1/beta-2/gamma-2	T08910	Muscle spasm [ICD-11: MB47.3]	78.51%	92.67%
GABA-A receptor; alpha-1/beta-2/gamma-2	T08910	Postpartum depression [ICD-11: 6E20.0]	78.51%	92.67%
GABA-A receptor; alpha-1/beta-2/gamma-2	T08910	Postpartum depression [ICD-11: 6E20.0]	78.51%	92.67%
GABA-A receptor; alpha-1/beta-2/gamma-2	T08910	Postpartum depression [ICD-11: 6E20.0]	78.51%	92.67%
GABA-A receptor; alpha-1/beta-2/gamma-2	T08910	Premenstrual syndrome [ICD-11: GA34.40]	78.51%	92.67%
GABA-A receptor; alpha-1/beta-2/gamma-2	T08910	Premenstrual syndrome [ICD-11: GA34.40]	78.51%	92.67%
GABA-A receptor; alpha-1/beta-2/gamma-2	T08910	Premenstrual syndrome [ICD-11: GA34.40]	78.51%	92.67%
GABA-A receptor; alpha-1/beta-2/gamma-2	T08910	Respiratory distress syndrome [ICD-11: CB00]	78.51%	92.67%
GABA-A receptor; alpha-1/beta-2/gamma-2	T08910	Status epilepticus seizure [ICD-11: 8A66.1Y]	78.51%	92.67%
GABA-A receptor; alpha-1/beta-2/gamma-2	T08910	Status epilepticus seizure [ICD-11: 8A66.1Y]	78.51%	92.67%
GABA-A receptor; alpha-1/beta-2/gamma-2	T08910	Status epilepticus seizure [ICD-11: 8A66.1Y]	78.51%	92.67%
Formyl peptide receptor 1	T87831	Inflammation [ICD-11: 1A00-CA43.1]	78.43%	93.56%
Formyl peptide receptor 1	T87831	Peptic ulcer [ICD-11: DA61]	78.43%	93.56%

Target Name	ChEMBL-ID	Indication	Probability	Model accuracy
Protein-tyrosine phosphatase 2C	T13057	Solid tumour/cancer [ICD-11: 2A00-2F9Z]	78.22%	94.42%
Glycine receptor subunit alpha-1	T50269	Muscle spasm [ICD-11: MB47.3]	78.03%	90.71%
Pregnane X receptor	T82702	Arteriosclerosis [ICD-11: BD40]	77.01%	94.73%
Nicotinamide phosphoribosyltransferase	T54582	leukaemia [ICD-11: 2A60-2B33]	76.35%	96.25%
Nicotinamide phosphoribosyltransferase	T54582	Solid tumour/cancer [ICD-11: 2A00-2F9Z]	76.35%	96.25%
DNA-(apurinic or apyrimidinic site) lyase	T13348	Glioma [ICD-11: 2A00.0]	75.42%	91.11%
DNA-(apurinic or apyrimidinic site) lyase	T13348	Melanoma [ICD-11: 2C30]	75.42%	91.11%
DNA-(apurinic or apyrimidinic site) lyase	T13348	Ocular cancer [ICD-11: 2D00-2D07]	75.42%	91.11%
DNA-(apurinic or apyrimidinic site) lyase	T13348	Solid tumour/cancer [ICD-11: 2A00-2F9Z]	75.42%	91.11%
Sodium channel protein type IV alpha subunit	T02546	Musculoskeletal disorder [ICD-11: FA00-FC0Z]	74.36%	92.32%
Sodium channel protein type IV alpha subunit	T02546	Neurological disorder [ICD-11: 6B60]	74.36%	92.32%
Sodium channel protein type IV alpha subunit	T02546	Pain [ICD-11: MG30-MG3Z]	74.36%	92.32%
Tyrosine-protein kinase receptor UFO	T82383	Acute myeloid leukaemia [ICD-11: 2A60]	73.43%	90.95%
Tyrosine-protein kinase receptor UFO	T82383	Breast cancer [ICD-11: 2C60-2C65]	73.43%	90.95%
Tyrosine-protein kinase receptor UFO	T82383	Metastatic malignant neoplasm [ICD-11: 2D50-2E09]	73.43%	90.95%
Tyrosine-protein kinase receptor UFO	T82383	Multiple myeloma [ICD-11: 2A83]	73.43%	90.95%
Tyrosine-protein kinase receptor UFO	T82383	Myelodysplastic syndrome [ICD-11: 2A37]	73.43%	90.95%
Tyrosine-protein kinase receptor UFO	T82383	Non-small-cell lung cancer [ICD-11: 2C25.Y]	73.43%	90.95%
Tyrosine-protein kinase receptor UFO	T82383	Solid tumour/cancer [ICD-11: 2A00-2F9Z]	73.43%	90.95%
Glutamate receptor ionotropic, AMPA 2	T42392	Diabetic neuropathy [ICD-11: 8C0Z]	73.12%	86.92%
Glutamate receptor ionotropic, AMPA 2	T42392	Parkinson disease [ICD-11: 8A00.0]	73.12%	86.92%
Cysteinyl leukotriene receptor 2	T74238	Asthma [ICD-11: CA23]	72.5%	98%
ADAM10	T31902	Breast cancer [ICD-11: 2C60-2C65]	71.31%	97.5%
ADAM10	T31902	Solid tumour/cancer [ICD-11: 2A00-2F9Z]	71.31%	97.5%
Aurora kinase B/Inner centromere protein	T46781	Acute myeloid leukaemia [ICD-11: 2A60]	70.87%	97.5%
Aurora kinase B/Inner centromere protein	T46781	Diabetic complication [ICD-11: 5A2Y]	70.87%	97.5%
Aurora kinase B/Inner centromere protein	T46781	Haematological malignancy [ICD-11: 2B33.Y]	70.87%	97.5%
Aurora kinase B/Inner centromere protein	T46781	Prostate cancer [ICD-11: 2C82.0]	70.87%	97.5%
Aurora kinase B/Inner centromere protein	T46781	Solid tumour/cancer [ICD-11: 2A00-2F9Z]	70.87%	97.5%

Target Name	ChEMBL-ID	Indication	Probability	Model accuracy
Histone deacetylase 8	T28887	Solid tumour/cancer [ICD-11: 2A00-2F9Z]	70.07%	93.99%
C5a anaphylatoxin chemotactic receptor	T15439	Atopic dermatitis [ICD-11: EA80]	69.27%	92.62%
C5a anaphylatoxin chemotactic receptor	T15439	Eosinophilic granulomatosis with polyangiitis [ICD-11: 4A44.A2]	69.27%	92.62%
C5a anaphylatoxin chemotactic receptor	T15439	Hereditary haemolytic anemia [ICD-11: 3A10.3]	69.27%	92.62%
C5a anaphylatoxin chemotactic receptor	T15439	Inflammation [ICD-11: 1A00-CA43.1]	69.27%	92.62%
C5a anaphylatoxin chemotactic receptor	T15439	Rheumatoid arthritis [ICD-11: FA20]	69.27%	92.62%
C5a anaphylatoxin chemotactic receptor	T15439	Vasculitis [ICD-11: 4A44]	69.27%	92.62%
C-X-C chemokine receptor type 4	T96079	Acute lymphoblastic leukaemia [ICD-11: 2A85]	68.01%	93.1%
C-X-C chemokine receptor type 4	T96079	Acute myeloid leukaemia [ICD-11: 2A60]	68.01%	93.1%
C-X-C chemokine receptor type 4	T96079	Autoimmune diabetes [ICD-11: 5A10]	68.01%	93.1%
C-X-C chemokine receptor type 4	T96079	B-cell chronic lymphocytic leukaemia [ICD-11: 2A82.00]	68.01%	93.1%
C-X-C chemokine receptor type 4	T96079	Bone marrow transplantation [ICD-11: QB63.6]	68.01%	93.1%
C-X-C chemokine receptor type 4	T96079	Breast cancer [ICD-11: 2C60-2C65]	68.01%	93.1%
C-X-C chemokine receptor type 4	T96079	Constitutional neutropenia [ICD-11: 4B00.0]	68.01%	93.1%
C-X-C chemokine receptor type 4	T96079	Haematological malignancy [ICD-11: 2B33.Y]	68.01%	93.1%
C-X-C chemokine receptor type 4	T96079	Human immunodeficiency virus infection [ICD-11: 1C62]	68.01%	93.1%
C-X-C chemokine receptor type 4	T96079	Macular degeneration [ICD-11: 9B78.3]	68.01%	93.1%
C-X-C chemokine receptor type 4	T96079	Melanoma [ICD-11: 2C30]	68.01%	93.1%
C-X-C chemokine receptor type 4	T96079	Merkel cell carcinoma [ICD-11: 2C34]	68.01%	93.1%
C-X-C chemokine receptor type 4	T96079	Multiple myeloma [ICD-11: 2A83]	68.01%	93.1%
C-X-C chemokine receptor type 4	T96079	Myelodysplastic syndrome [ICD-11: 2A37]	68.01%	93.1%
C-X-C chemokine receptor type 4	T96079	Non-hodgkin lymphoma [ICD-11: 2B33.5]	68.01%	93.1%
C-X-C chemokine receptor type 4	T96079	Pancreatic cancer [ICD-11: 2C10]	68.01%	93.1%
C-X-C chemokine receptor type 4	T96079	Peripheral vascular disease [ICD-11: BD4Z]	68.01%	93.1%
C-X-C chemokine receptor type 4	T96079	Psoriasis vulgaris [ICD-11: EA90]	68.01%	93.1%
C-X-C chemokine receptor type 4	T96079	Recurring respiratory infection [ICD-11: CA07-CA45]	68.01%	93.1%
C-X-C chemokine receptor type 4	T96079	Renal cell carcinoma [ICD-11: 2C90]	68.01%	93.1%
C-X-C chemokine receptor type 4	T96079	Sarcoma [ICD-11: 2A60-2C35]	68.01%	93.1%
C-X-C chemokine receptor type 4	T96079	Solid tumour/cancer [ICD-11: 2A00-2F9Z]	68.01%	93.1%
Cytochrome P450 3A4	T37848	Atopic dermatitis [ICD-11: EA80]	67.36%	91.19%

Target Name	ChEMBL-ID	Indication	Probability	Model accuracy
Cytochrome P450 3A4	T37848	Solid tumour/cancer [ICD-11: 2A00-2F9Z]	67.36%	91.19%
Amine oxidase, copper containing	T69619	Asthma [ICD-11: CA23]	65.49%	94%
Amine oxidase, copper containing	T69619	Atopic dermatitis [ICD-11: EA80]	65.49%	94%
Amine oxidase, copper containing	T69619	Central nervous system disease [ICD-11: 8A04-8D87]	65.49%	94%
Amine oxidase, copper containing	T69619	Cerebrovascular ischaemia [ICD-11: 8B1Z]	65.49%	94%
Amine oxidase, copper containing	T69619	Diabetic complication [ICD-11: 5A2Y]	65.49%	94%
Amine oxidase, copper containing	T69619	Diabetic macular edema [ICD-11: 9B71.02]	65.49%	94%
Amine oxidase, copper containing	T69619	Diabetic nephropathy [ICD-11: GB61.Z]	65.49%	94%
Amine oxidase, copper containing	T69619	Hypertension [ICD-11: BA00-BA04]	65.49%	94%
Amine oxidase, copper containing	T69619	Inflammation [ICD-11: 1A00-CA43.1]	65.49%	94%
Amine oxidase, copper containing	T69619	Psoriasis vulgaris [ICD-11: EA90]	65.49%	94%
Amine oxidase, copper containing	T69619	Pulmonary disease [ICD-11: 1B10-1F85]	65.49%	94%
Amine oxidase, copper containing	T69619	Vascular disease [ICD-11: BE2Z]	65.49%	94%
Transient receptor potential cation channel subfamily A member 1	T84040	Diabetic neuropathy [ICD-11: 8C0Z]	65.15%	92.17%
Transient receptor potential cation channel subfamily A member 1	T84040	Pain [ICD-11: MG30-MG3Z]	65.15%	92.17%
Transient receptor potential cation channel subfamily A member 1	T84040	Throat irritation [ICD-11: CA0Y]	65.15%	92.17%
Nitric oxide synthase, inducible	T02703	Artery stenosis [ICD-11: BD52]	63.64%	94.8%
Nitric oxide synthase, inducible	T02703	Asthma [ICD-11: CA23]	63.64%	94.8%
Nitric oxide synthase, inducible	T02703	Cardiovascular disease [ICD-11: BA00-BE2Z]	63.64%	94.8%
Nitric oxide synthase, inducible	T02703	Coronary artery disease [ICD-11: BA80]	63.64%	94.8%
Nitric oxide synthase, inducible	T02703	Diabetic kidney disease [ICD-11: GB61.Z]	63.64%	94.8%
Nitric oxide synthase, inducible	T02703	Diabetic retinopathy [ICD-11: 9B71.0]	63.64%	94.8%
Nitric oxide synthase, inducible	T02703	Diarrhea [ICD-11: ME05.1]	63.64%	94.8%
Nitric oxide synthase, inducible	T02703	Endotoxic shock [ICD-11: 1G41]	63.64%	94.8%
Nitric oxide synthase, inducible	T02703	Inflammation [ICD-11: 1A00-CA43.1]	63.64%	94.8%
Nitric oxide synthase, inducible	T02703	Insulin-resistant disorder [ICD-11: 5A44]	63.64%	94.8%
Nitric oxide synthase, inducible	T02703	Osteoarthritis [ICD-11: FA00-FA05]	63.64%	94.8%
Nitric oxide synthase, inducible	T02703	Postoperative pain [ICD-11: MG30-MG3Z]	63.64%	94.8%
Nitric oxide synthase, inducible	T02703	Rheumatoid arthritis [ICD-11: FA20]	63.64%	94.8%
Nitric oxide synthase, inducible	T02703	Sepsis [ICD-11: 1G40-1G41]	63.64%	94.8%
Nitric oxide synthase, inducible	T02703	Solid tumour/cancer [ICD-11: 2A00-2F9Z]	63.64%	94.8%
Sphingosine 1-phosphate receptor Edg-8	T50089	Multiple sclerosis [ICD-11: 8A40]	63.62%	100%
Methionine aminopeptidase 2	T75596	Autoimmune diabetes [ICD-11:	62.86%	97.28%

Target Name	ChEMBL-ID	Indication	Probability	Model accuracy
		5A10]		
Methionine aminopeptidase 2	T75596	Obesity [ICD-11: 5B81]	62.86%	97.28%
Methionine aminopeptidase 2	T75596	Solid tumour/cancer [ICD-11: 2A00-2F9Z]	62.86%	97.28%
Voltage-gated N-type calcium channel alpha-1B subunit	T38338	Anxiety disorder [ICD-11: 6B00-6B0Z]	61.85%	97.14%
Voltage-gated N-type calcium channel alpha-1B subunit	T38338	Cardiac failure [ICD-11: BD10-BD13]	61.85%	97.14%
Voltage-gated N-type calcium channel alpha-1B subunit	T38338	High blood pressure [ICD-11: BA00]	61.85%	97.14%
Voltage-gated N-type calcium channel alpha-1B subunit	T38338	Migraine [ICD-11: 8A80]	61.85%	97.14%
Voltage-gated N-type calcium channel alpha-1B subunit	T38338	Neuropathic pain [ICD-11: 8E43.0]	61.85%	97.14%
Voltage-gated N-type calcium channel alpha-1B subunit	T38338	Pain [ICD-11: MG30-MG3Z]	61.85%	97.14%
Voltage-gated N-type calcium channel alpha-1B subunit	T38338	Reperfusion injury [ICD-11: ND56.Z]	61.85%	97.14%
Voltage-gated N-type calcium channel alpha-1B subunit	T38338	Traumatic brain injury [ICD-11: NA07.Z]	61.85%	97.14%
Voltage-gated N-type calcium channel alpha-1B subunit	T38338	Vascular purpura [ICD-11: 3B60]	61.85%	97.14%
DNA topoisomerase I	T09826	Acquired immune deficiency syndrome [ICD-11: 1C62.3]	61.22%	97%
DNA topoisomerase I	T09826	Acute lymphoblastic leukaemia [ICD-11: 2A85]	61.22%	97%
DNA topoisomerase I	T09826	Bacterial infection [ICD-11: 1A00-1C4Z]	61.22%	97%
DNA topoisomerase I	T09826	Bladder cancer [ICD-11: 2C94]	61.22%	97%
DNA topoisomerase I	T09826	Breast cancer [ICD-11: 2C60-2C65]	61.22%	97%
DNA topoisomerase I	T09826	Colorectal cancer [ICD-11: 2B91.Z]	61.22%	97%
DNA topoisomerase I	T09826	Esophageal cancer [ICD-11: 2B70]	61.22%	97%
DNA topoisomerase I	T09826	Gastric adenocarcinoma [ICD-11: 2B72]	61.22%	97%
DNA topoisomerase I	T09826	Glioblastoma multiforme [ICD-11: 2A00.0]	61.22%	97%
DNA topoisomerase I	T09826	Human immunodeficiency virus infection [ICD-11: 1C62]	61.22%	97%
DNA topoisomerase I	T09826	Lung cancer [ICD-11: 2C25.0]	61.22%	97%
DNA topoisomerase I	T09826	Lymphoma [ICD-11: 2A80-2A86]	61.22%	97%
DNA topoisomerase I	T09826	Metastatic colorectal cancer [ICD-11: 2B91]	61.22%	97%
DNA topoisomerase I	T09826	Myelodysplastic syndrome [ICD-11: 2A37]	61.22%	97%
DNA topoisomerase I	T09826	Ovarian cancer [ICD-11: 2C73]	61.22%	97%
DNA topoisomerase I	T09826	Renal cell carcinoma [ICD-11: 2C90]	61.22%	97%
DNA topoisomerase I	T09826	Small-cell lung cancer [ICD-11: 2C25.Y]	61.22%	97%
DNA topoisomerase I	T09826	Solid tumour/cancer [ICD-11: 2A00-2F9Z]	61.22%	97%
Rho-associated protein kinase 1	T51282	Arteriosclerosis [ICD-11: BD40]	60.84%	95.55%

Target Name	ChEMBL-ID	Indication	Probability	Model accuracy
Rho-associated protein kinase 1	T51282	Chronic obstructive pulmonary disease [ICD-11: CA22]	60.84%	95.55%
Rho-associated protein kinase 1	T51282	Diabetic nephropathy [ICD-11: GB61.Z]	60.84%	95.55%
Rho-associated protein kinase 1	T51282	Inflammation [ICD-11: 1A00-CA43.1]	60.84%	95.55%
Rho-associated protein kinase 1	T51282	Microvascular angina [ICD-11: BA86]	60.84%	95.55%
Rho-associated protein kinase 1	T51282	Open-angle glaucoma [ICD-11: 9C61]	60.84%	95.55%
Rho-associated protein kinase 1	T51282	Pain [ICD-11: MG30-MG3Z]	60.84%	95.55%
Rho-associated protein kinase 1	T51282	Pulmonary arterial hypertension [ICD-11: BB01.0]	60.84%	95.55%
Rho-associated protein kinase 1	T51282	Solid tumour/cancer [ICD-11: 2A00-2F9Z]	60.84%	95.55%
Toll-like receptor 4	T81443	Allergy [ICD-11: 4A80-4A85]	60.29%	92.5%
Toll-like receptor 4	T81443	Autoimmune diabetes [ICD-11: 5A10]	60.29%	92.5%
Toll-like receptor 4	T81443	Hepatitis virus infection [ICD-11: 1E50-1E51]	60.29%	92.5%
Toll-like receptor 4	T81443	Melanoma [ICD-11: 2C30]	60.29%	92.5%
Toll-like receptor 4	T81443	Non-hodgkin lymphoma [ICD-11: 2B33.5]	60.29%	92.5%
Toll-like receptor 4	T81443	Prostate cancer [ICD-11: 2C82.0]	60.29%	92.5%
Toll-like receptor 4	T81443	Sepsis [ICD-11: 1G40-1G41]	60.29%	92.5%
Toll-like receptor 4	T81443	Solid tumour/cancer [ICD-11: 2A00-2F9Z]	60.29%	92.5%
Muscarinic acetylcholine receptor M4	T20709	Asthma [ICD-11: CA23]	60.2%	94.08%
Muscarinic acetylcholine receptor M4	T20709	Attention deficit hyperactivity disorder [ICD-11: 6A05.Z]	60.2%	94.08%
Muscarinic acetylcholine receptor M4	T20709	bronchial hyperreactivity [ICD-11: CB40]	60.2%	94.08%
Muscarinic acetylcholine receptor M4	T20709	Glaucoma/ocular hypertension [ICD-11: 9C61]	60.2%	94.08%
Muscarinic acetylcholine receptor M4	T20709	Mydriasis [ICD-11: LA11.62]	60.2%	94.08%
Muscarinic acetylcholine receptor M4	T20709	Nausea [ICD-11: MD90]	60.2%	94.08%
Macrophage migration inhibitory factor	T39977	Autoimmune diabetes [ICD-11: 5A10]	59.13%	80.78%
Macrophage migration inhibitory factor	T39977	Autoimmune disease [ICD-11: 4A40-4A45]	59.13%	80.78%
Macrophage migration inhibitory factor	T39977	Inflammation [ICD-11: 1A00-CA43.1]	59.13%	80.78%
Histone deacetylase 2	T51191	Dermatitis [ICD-11: EA80-EA89]	58.9%	94.75%
Histone deacetylase 2	T51191	Solid tumour/cancer [ICD-11: 2A00-2F9Z]	58.9%	94.75%
Histone deacetylase 4	T63816	Advanced stage follicular lymphoma [ICD-11: 2A80]	57.59%	92.97%
Histone deacetylase 4	T63816	Huntington disease [ICD-11: 8A01.10]	57.59%	92.97%
Histone deacetylase 4	T63816	Solid tumour/cancer [ICD-11: 2A00-2F9Z]	57.59%	92.97%

Target Name	ChEMBL-ID	Indication	Probability	Model accuracy
Integrin alpha-5/beta-1	T29879	Allergic rhinitis [ICD-11: CA08.0]	57.49%	96.39%
Integrin alpha-5/beta-1	T29879	Allergy [ICD-11: 4A80-4A85]	57.49%	96.39%
Integrin alpha-5/beta-1	T29879	Asthma [ICD-11: CA23]	57.49%	96.39%
Integrin alpha-5/beta-1	T29879	Macular degeneration [ICD-11: 9B78.3]	57.49%	96.39%
Integrin alpha-5/beta-1	T29879	Macular degeneration [ICD-11: 9B78.3]	57.49%	96.39%
Integrin alpha-5/beta-1	T29879	Multiple sclerosis [ICD-11: 8A40]	57.49%	96.39%
Integrin alpha-5/beta-1	T29879	Non-small-cell lung cancer [ICD-11: 2C25.Y]	57.49%	96.39%
Leukotriene A4 hydrolase	T03691	Acute myeloid leukaemia [ICD-11: 2A60]	57.28%	97.86%
Leukotriene A4 hydrolase	T03691	Chronic blistering skin disorder [ICD-11: ME63.3]	57.28%	97.86%
Leukotriene A4 hydrolase	T03691	Inflammation [ICD-11: 1A00-CA43.1]	57.28%	97.86%
Leukotriene A4 hydrolase	T03691	Myocardial infarction [ICD-11: BA41-BA43]	57.28%	97.86%
Delta opioid receptor	T58992	Alcohol dependence [ICD-11: 6C40.2]	57.26%	99.35%
Delta opioid receptor	T58992	Anxiety disorder [ICD-11: 6B00-6B0Z]	57.26%	99.35%
Delta opioid receptor	T58992	Back pain [ICD-11: ME84.Z]	57.26%	99.35%
Delta opioid receptor	T58992	Bladder disease [ICD-11: DC11-DC1Z]	57.26%	99.35%
Delta opioid receptor	T58992	Cerebral vasospasm [ICD-11: BA85.Z]	57.26%	99.35%
Delta opioid receptor	T58992	Cough [ICD-11: MD12]	57.26%	99.35%
Delta opioid receptor	T58992	Diarrhea [ICD-11: ME05.1]	57.26%	99.35%
Delta opioid receptor	T58992	Diarrhea-predominant irritable bowel syndrome [ICD-11: DD91.01]	57.26%	99.35%
Delta opioid receptor	T58992	Ischemic heart disease [ICD-11: BA40-BA6Z]	57.26%	99.35%
Delta opioid receptor	T58992	Major depressive disorder [ICD-11: 6A70.3]	57.26%	99.35%
Delta opioid receptor	T58992	Migraine [ICD-11: 8A80]	57.26%	99.35%
Delta opioid receptor	T58992	Obesity [ICD-11: 5B81]	57.26%	99.35%
Delta opioid receptor	T58992	Opioid dependence [ICD-11: 6C43.2Z]	57.26%	99.35%
Delta opioid receptor	T58992	Overactive bladder [ICD-11: GC50.0]	57.26%	99.35%
Delta opioid receptor	T58992	Pain [ICD-11: MG30-MG3Z]	57.26%	99.35%
Delta opioid receptor	T58992	Premature ejaculation [ICD-11: HA03.0Z]	57.26%	99.35%
Delta opioid receptor	T58992	Rheumatoid arthritis [ICD-11: FA20]	57.26%	99.35%
Delta opioid receptor	T58992	Substance use disorder [ICD-11: 6C4Z]	57.26%	99.35%
Delta opioid receptor	T58992	Urinary incontinence [ICD-11: MF50.2]	57.26%	99.35%
Platelet-derived growth factor receptor alpha	T53524	Acute myelogenous leukaemia [ICD-11: 2A41]	57.14%	91.07%
Platelet-derived growth factor receptor alpha	T53524	Acute myeloid leukaemia [ICD-11: 2A60]	57.14%	91.07%

Target Name	ChEMBL-ID	Indication	Probability	Model accuracy
Platelet-derived growth factor receptor alpha	T53524	Breast cancer [ICD-11: 2C60-2C65]	57.14%	91.07%
Platelet-derived growth factor receptor alpha	T53524	Colon cancer [ICD-11: 2B90.Z]	57.14%	91.07%
Platelet-derived growth factor receptor alpha	T53524	Colorectal cancer [ICD-11: 2B91.Z]	57.14%	91.07%
Platelet-derived growth factor receptor alpha	T53524	Gastric adenocarcinoma [ICD-11: 2B72]	57.14%	91.07%
Platelet-derived growth factor receptor alpha	T53524	Gastrointestinal cancer [ICD-11: 2C11]	57.14%	91.07%
Platelet-derived growth factor receptor alpha	T53524	Gastrointestinal stromal tumour [ICD-11: 2B5B]	57.14%	91.07%
Platelet-derived growth factor receptor alpha	T53524	Glioblastoma multiforme [ICD-11: 2A00.0]	57.14%	91.07%
Platelet-derived growth factor receptor alpha	T53524	Gram-positive bacterial infection [ICD-11: 1B74-1G40]	57.14%	91.07%
Platelet-derived growth factor receptor alpha	T53524	Lung cancer [ICD-11: 2C25.0]	57.14%	91.07%
Platelet-derived growth factor receptor alpha	T53524	Mast cell leukaemia [ICD-11: 2A21.00]	57.14%	91.07%
Platelet-derived growth factor receptor alpha	T53524	Multiple myeloma [ICD-11: 2A83]	57.14%	91.07%
Platelet-derived growth factor receptor alpha	T53524	Myelodysplastic syndrome [ICD-11: 2A37]	57.14%	91.07%
Platelet-derived growth factor receptor alpha	T53524	Non-small-cell lung cancer [ICD-11: 2C25.Y]	57.14%	91.07%
Platelet-derived growth factor receptor alpha	T53524	Pancreatic cancer [ICD-11: 2C10]	57.14%	91.07%
Platelet-derived growth factor receptor alpha	T53524	Prostate cancer [ICD-11: 2C82.0]	57.14%	91.07%
Platelet-derived growth factor receptor alpha	T53524	Recurrent glioblastoma [ICD-11: 2A00.00]	57.14%	91.07%
Platelet-derived growth factor receptor alpha	T53524	Soft tissue sarcoma [ICD-11: 2B57]	57.14%	91.07%
Platelet-derived growth factor receptor alpha	T53524	Solid tumour/cancer [ICD-11: 2A00-2F9Z]	57.14%	91.07%
Platelet-derived growth factor receptor alpha	T53524	Systemic mastocytosis [ICD-11: 2A21.0]	57.14%	91.07%
Platelet-derived growth factor receptor alpha	T53524	Thrombocytopenia [ICD-11: 3B64]	57.14%	91.07%
Phosphodiesterase 3A	T88975	Bronchial asthma [ICD-11: CA23]	55.84%	92.94%
Phosphodiesterase 3A	T88975	Cardiac failure [ICD-11: BD10-BD13]	55.84%	92.94%
Phosphodiesterase 3A	T88975	Cardiovascular disease [ICD-11: BA00-BE2Z]	55.84%	92.94%
Phosphodiesterase 3A	T88975	Congestive heart failure [ICD-11: BD10]	55.84%	92.94%
Phosphodiesterase 3A	T88975	Intermittent claudication [ICD-11: BD40.00]	55.84%	92.94%
Phosphodiesterase 3A	T88975	Thrombocythemia [ICD-11: 3B63]	55.84%	92.94%
Cyclin-dependent kinase 4/cyclin D1	T85799	Breast cancer [ICD-11: 2C60-2C65]	55.84%	98.99%
Cyclin-dependent kinase 4/cyclin D1	T85799	Haematological malignancy [ICD-11: 2B33.Y]	55.84%	98.99%

Target Name	ChEMBL-ID	Indication	Probability	Model accuracy
Cyclin-dependent kinase 4/cyclin D1	T85799	Mantle cell lymphoma [ICD-11: 2A85.5]	55.84%	98.99%
Cyclin-dependent kinase 4/cyclin D1	T85799	Myelofibrosis [ICD-11: 2A20.2]	55.84%	98.99%
Cyclin-dependent kinase 4/cyclin D1	T85799	Psoriasis vulgaris [ICD-11: EA90]	55.84%	98.99%
Cyclin-dependent kinase 4/cyclin D1	T85799	Relapsed or refractory chronic lymphocytic leukaemia [ICD-11: 2A82.0]	55.84%	98.99%
Cyclin-dependent kinase 4/cyclin D1	T85799	Retinoblastoma [ICD-11: 2D02.2]	55.84%	98.99%
Cyclin-dependent kinase 4/cyclin D1	T85799	Schizophrenia [ICD-11: 6A20]	55.84%	98.99%
Cyclin-dependent kinase 4/cyclin D1	T85799	Small-cell lung cancer [ICD-11: 2C25.Y]	55.84%	98.99%
Cyclin-dependent kinase 4/cyclin D1	T85799	Solid tumour/cancer [ICD-11: 2A00-2F9Z]	55.84%	98.99%
Cyclin-dependent kinase 4/cyclin D1	T85799	Solid tumour/cancer [ICD-11: 2A00-2F9Z]	55.84%	98.99%
Acyl-CoA desaturase	T10897	Bacterial infection [ICD-11: 1A00-1C4Z]	53.98%	97.5%
Acyl-CoA desaturase	T10897	Diabetic complication [ICD-11: 5A2Y]	53.98%	97.5%
Acyl-CoA desaturase	T10897	Lipid metabolism disorder [ICD-11: 5C52.Z]	53.98%	97.5%
Acyl-CoA desaturase	T10897	Type-2 diabetes [ICD-11: 5A11]	53.98%	97.5%
Neurokinin 2 receptor	T52790	Acute or chronic pain [ICD-11: MG30-MG31]	52.39%	98.89%
Neurokinin 2 receptor	T52790	Anxiety disorder [ICD-11: 6B00-6B0Z]	52.39%	98.89%
Neurokinin 2 receptor	T52790	Asthma [ICD-11: CA23]	52.39%	98.89%
Neurokinin 2 receptor	T52790	Atopic dermatitis [ICD-11: EA80]	52.39%	98.89%
Neurokinin 2 receptor	T52790	Chronic obstructive pulmonary disease [ICD-11: CA22]	52.39%	98.89%
Neurokinin 2 receptor	T52790	Depression [ICD-11: 6A70-6A7Z]	52.39%	98.89%
Neurokinin 2 receptor	T52790	Infantile colics [ICD-11: DD93.1]	52.39%	98.89%
Neurokinin 2 receptor	T52790	Irritable bowel syndrome [ICD-11: DD91.0]	52.39%	98.89%
Neurokinin 2 receptor	T52790	Pain [ICD-11: MG30-MG3Z]	52.39%	98.89%
Neurokinin 2 receptor	T52790	Postoperative ileus [ICD-11: DA91-DB30]	52.39%	98.89%
Neurokinin 2 receptor	T52790	Psoriasis vulgaris [ICD-11: EA90]	52.39%	98.89%
Neurokinin 2 receptor	T52790	Urinary incontinence [ICD-11: MF50.2]	52.39%	98.89%
Glutaminase kidney isoform, mitochondrial	T86734	Breast cancer [ICD-11: 2C60-2C65]	51.69%	100%
Glutaminase kidney isoform, mitochondrial	T86734	leukaemia [ICD-11: 2A60-2B33]	51.69%	100%
Glutaminase kidney isoform, mitochondrial	T86734	Renal cell carcinoma [ICD-11: 2C90]	51.69%	100%
Glutaminase kidney isoform, mitochondrial	T86734	Solid tumour/cancer [ICD-11: 2A00-2F9Z]	51.69%	100%
Cystic fibrosis transmembrane conductance regulator	T55654	Acute lung injury [ICD-11: NB32.3]	50.17%	95.71%
Cystic fibrosis transmembrane	T55654	Cystic fibrosis [ICD-11: CA25]	50.17%	95.71%

Target Name	ChEMBL-ID	Indication	Probability	Model accuracy
conductance regulator				
Cystic fibrosis transmembrane conductance regulator	T55654	Diarrhea [ICD-11: ME05.1]	50.17%	95.71%
Cystic fibrosis transmembrane conductance regulator	T55654	HIV-associated diarrhoea [ICD-11: 1A2Z]	50.17%	95.71%
