# Supplementary material for: 3,4-Dihydropyrimidin-2(1H)-One Hybrids as Phenotypic Anti-Breast Cancer Agents: Synthesis, Colony-Formation Profiling and Computational Target Deconvolution
Source: Int J Mol Sci. 2026 Oct 27;27(21):9618. doi: 10.3390/ijms27219618 (PMC13763226; doi:10.3390/ijms27219618)
Supplement: Supplementary file 1 [file ijms-27-09618-s001.zip › Super-PRED_exported_data.zip!/Targets_4i.pdf]

Exported data

Target Name	ChEMBL-ID	Indication	Probability	Model accuracy
Cathepsin D	T67102	Hypertension [ICD-11: BA00-BA04]	96.97%	98.95%
Cathepsin D	T67102	Multiple sclerosis [ICD-11: 8A40]	96.97%	98.95%
Dihydrofolate reductase	T17345	Acute bacterial skin infection [ICD-11: 1C41]	91.76%	89.92%
Dihydrofolate reductase	T17345	Bacterial infection [ICD-11: 1A00-1C4Z]	91.76%	89.92%
Dihydrofolate reductase	T17345	Bladder cancer [ICD-11: 2C94]	91.76%	89.92%
Dihydrofolate reductase	T17345	Inflammatory bowel disease [ICD-11: DD72]	91.76%	89.92%
Dihydrofolate reductase	T17345	Pneumonia [ICD-11: CA40]	91.76%	89.92%
Dihydrofolate reductase	T17345	Pulmonary and extrapulmonary tuberculosis [ICD-11: 1B10.Z]	91.76%	89.92%
Dihydrofolate reductase	T17345	Rheumatoid arthritis [ICD-11: FA20]	91.76%	89.92%
Dihydrofolate reductase	T17345	Solid tumour/cancer [ICD-11: 2A00-2F9Z]	91.76%	89.92%
Dihydrofolate reductase	T17345	Urinary tract infection [ICD-11: GC08]	91.76%	89.92%
GABA-A receptor; alpha-1/beta-2/ gamma-2	T08910	Alcohol dependence [ICD-11: 6C40.2]	85.47%	92.67%
GABA-A receptor; alpha-1/beta-2/ gamma-2	T08910	Anaesthesia [ICD-11: 9A78.6]	85.47%	92.67%
GABA-A receptor; alpha-1/beta-2/ gamma-2	T08910	Anxiety disorder [ICD-11: 6B00-6B0Z]	85.47%	92.67%
GABA-A receptor; alpha-1/beta-2/ gamma-2	T08910	Anxiety disorder [ICD-11: 6B00-6B0Z]	85.47%	92.67%
GABA-A receptor; alpha-1/beta-2/ gamma-2	T08910	Anxiety disorder [ICD-11: 6B00-6B0Z]	85.47%	92.67%
GABA-A receptor; alpha-1/beta-2/ gamma-2	T08910	Chronic pain [ICD-11: MG30]	85.47%	92.67%
GABA-A receptor; alpha-1/beta-2/ gamma-2	T08910	Cystitis [ICD-11: GC00]	85.47%	92.67%
GABA-A receptor; alpha-1/beta-2/ gamma-2	T08910	Depression [ICD-11: 6A70-6A7Z]	85.47%	92.67%
GABA-A receptor; alpha-1/beta-2/ gamma-2	T08910	Depression [ICD-11: 6A70-6A7Z]	85.47%	92.67%
GABA-A receptor; alpha-1/beta-2/ gamma-2	T08910	Depression [ICD-11: 6A70-6A7Z]	85.47%	92.67%
GABA-A receptor; alpha-1/beta-2/ gamma-2	T08910	Epilepsy [ICD-11: 8A60-8A68]	85.47%	92.67%
GABA-A receptor; alpha-1/beta-2/ gamma-2	T08910	Epileptic seizures [ICD-11: 8A61-8A6Z]	85.47%	92.67%
GABA-A receptor; alpha-1/beta-2/ gamma-2	T08910	Epileptic seizures [ICD-11: 8A61-8A6Z]	85.47%	92.67%
GABA-A receptor; alpha-1/beta-2/ gamma-2	T08910	Epileptic seizures [ICD-11: 8A61-8A6Z]	85.47%	92.67%
GABA-A receptor; alpha-1/beta-2/ gamma-2	T08910	Essential tremor or related tremors [ICD-11: 8A04.1]	85.47%	92.67%
GABA-A receptor; alpha-1/beta-2/ gamma-2	T08910	Essential tremor or related tremors [ICD-11: 8A04.1]	85.47%	92.67%
GABA-A receptor; alpha-1/beta-2/	T08910	Essential tremor or related tremors	85.47%	92.67%

Target Name	ChEMBL-ID	Indication	Probability	Model accuracy
gamma-2		[ICD-11: 8A04.1]		
GABA-A receptor; alpha-1/beta-2/ gamma-2	T08910	Fibromyalgia [ICD-11: MG30.01]	85.47%	92.67%
GABA-A receptor; alpha-1/beta-2/ gamma-2	T08910	Headache [ICD-11: 8A80-8A84]	85.47%	92.67%
GABA-A receptor; alpha-1/beta-2/ gamma-2	T08910	Inflammation [ICD-11: 1A00-CA43.1]	85.47%	92.67%
GABA-A receptor; alpha-1/beta-2/ gamma-2	T08910	Inflammation [ICD-11: 1A00-CA43.1]	85.47%	92.67%
GABA-A receptor; alpha-1/beta-2/ gamma-2	T08910	Inflammation [ICD-11: 1A00-CA43.1]	85.47%	92.67%
GABA-A receptor; alpha-1/beta-2/ gamma-2	T08910	Inflammatory bowel disease [ICD-11: DD72]	85.47%	92.67%
GABA-A receptor; alpha-1/beta-2/ gamma-2	T08910	Inflammatory bowel disease [ICD-11: DD72]	85.47%	92.67%
GABA-A receptor; alpha-1/beta-2/ gamma-2	T08910	Inflammatory bowel disease [ICD-11: DD72]	85.47%	92.67%
GABA-A receptor; alpha-1/beta-2/ gamma-2	T08910	Insomnia [ICD-11: 7A00-7A0Z]	85.47%	92.67%
GABA-A receptor; alpha-1/beta-2/ gamma-2	T08910	Insomnia [ICD-11: 7A00-7A0Z]	85.47%	92.67%
GABA-A receptor; alpha-1/beta-2/ gamma-2	T08910	Intractable insomnia [ICD-11: 7A00]	85.47%	92.67%
GABA-A receptor; alpha-1/beta-2/ gamma-2	T08910	Ischemic reperfusion injury [ICD-11: DB98.B]	85.47%	92.67%
GABA-A receptor; alpha-1/beta-2/ gamma-2	T08910	Malaria [ICD-11: 1F40-1F45]	85.47%	92.67%
GABA-A receptor; alpha-1/beta-2/ gamma-2	T08910	Muscle spasm [ICD-11: MB47.3]	85.47%	92.67%
GABA-A receptor; alpha-1/beta-2/ gamma-2	T08910	Muscle spasm [ICD-11: MB47.3]	85.47%	92.67%
GABA-A receptor; alpha-1/beta-2/ gamma-2	T08910	Muscle spasm [ICD-11: MB47.3]	85.47%	92.67%
GABA-A receptor; alpha-1/beta-2/ gamma-2	T08910	Postpartum depression [ICD-11: 6E20.0]	85.47%	92.67%
GABA-A receptor; alpha-1/beta-2/ gamma-2	T08910	Postpartum depression [ICD-11: 6E20.0]	85.47%	92.67%
GABA-A receptor; alpha-1/beta-2/ gamma-2	T08910	Postpartum depression [ICD-11: 6E20.0]	85.47%	92.67%
GABA-A receptor; alpha-1/beta-2/ gamma-2	T08910	Premenstrual syndrome [ICD-11: GA34.40]	85.47%	92.67%
GABA-A receptor; alpha-1/beta-2/ gamma-2	T08910	Premenstrual syndrome [ICD-11: GA34.40]	85.47%	92.67%
GABA-A receptor; alpha-1/beta-2/ gamma-2	T08910	Premenstrual syndrome [ICD-11: GA34.40]	85.47%	92.67%
GABA-A receptor; alpha-1/beta-2/ gamma-2	T08910	Respiratory distress syndrome [ICD-11: CB00]	85.47%	92.67%
GABA-A receptor; alpha-1/beta-2/ gamma-2	T08910	Status epilepticus seizure [ICD-11: 8A66.1Y]	85.47%	92.67%
GABA-A receptor; alpha-1/beta-2/ gamma-2	T08910	Status epilepticus seizure [ICD-11: 8A66.1Y]	85.47%	92.67%
GABA-A receptor; alpha-1/beta-2/ gamma-2	T08910	Status epilepticus seizure [ICD-11: 8A66.1Y]	85.47%	92.67%

Target Name	ChEMBL-ID	Indication	Probability	Model accuracy
Sodium/hydrogen exchanger 1	T82028	Angina pectoris [ICD-11: BA40]	85.37%	90.24%
Sodium/hydrogen exchanger 1	T82028	Cardiac arrhythmias [ICD-11: BC9Z]	85.37%	90.24%
Sodium/hydrogen exchanger 1	T82028	Heart arrhythmia [ICD-11: BC65]	85.37%	90.24%
Sodium/hydrogen exchanger 1	T82028	Myocardial infarction [ICD-11: BA41-BA43]	85.37%	90.24%
Casein kinase II alpha/beta	T51565	Cholangiocarcinoma [ICD-11: 2C12.10]	85.24%	99.23%
Casein kinase II alpha/beta	T51565	Solid tumour/cancer [ICD-11: 2A00-2F9Z]	85.24%	99.23%
Muscarinic acetylcholine receptor M4	T20709	Asthma [ICD-11: CA23]	85.14%	94.08%
Muscarinic acetylcholine receptor M4	T20709	Attention deficit hyperactivity disorder [ICD-11: 6A05.Z]	85.14%	94.08%
Muscarinic acetylcholine receptor M4	T20709	bronchial hyperreactivity [ICD-11: CB40]	85.14%	94.08%
Muscarinic acetylcholine receptor M4	T20709	Glaucoma/ocular hypertension [ICD-11: 9C61]	85.14%	94.08%
Muscarinic acetylcholine receptor M4	T20709	Mydriasis [ICD-11: LA11.62]	85.14%	94.08%
Muscarinic acetylcholine receptor M4	T20709	Nausea [ICD-11: MD90]	85.14%	94.08%
Beta-glucuronidase	T96413	Mucopolysaccharidosis [ICD-11: 5C56.3]	84.88%	77.68%
Beta-glucuronidase	T96413	Periodontal disease [ICD-11: DA0C]	84.88%	77.68%
Formyl peptide receptor 1	T87831	Inflammation [ICD-11: 1A00-CA43.1]	84.3%	93.56%
Formyl peptide receptor 1	T87831	Peptic ulcer [ICD-11: DA61]	84.3%	93.56%
C-X-C chemokine receptor type 4	T96079	Acute lymphoblastic leukaemia [ICD-11: 2A85]	81.43%	93.1%
C-X-C chemokine receptor type 4	T96079	Acute myeloid leukaemia [ICD-11: 2A60]	81.43%	93.1%
C-X-C chemokine receptor type 4	T96079	Autoimmune diabetes [ICD-11: 5A10]	81.43%	93.1%
C-X-C chemokine receptor type 4	T96079	B-cell chronic lymphocytic leukaemia [ICD-11: 2A82.00]	81.43%	93.1%
C-X-C chemokine receptor type 4	T96079	Bone marrow transplantation [ICD-11: QB63.6]	81.43%	93.1%
C-X-C chemokine receptor type 4	T96079	Breast cancer [ICD-11: 2C60-2C65]	81.43%	93.1%
C-X-C chemokine receptor type 4	T96079	Constitutional neutropenia [ICD-11: 4B00.0]	81.43%	93.1%
C-X-C chemokine receptor type 4	T96079	Haematological malignancy [ICD-11: 2B33.Y]	81.43%	93.1%
C-X-C chemokine receptor type 4	T96079	Human immunodeficiency virus infection [ICD-11: 1C62]	81.43%	93.1%
C-X-C chemokine receptor type 4	T96079	Macular degeneration [ICD-11: 9B78.3]	81.43%	93.1%
C-X-C chemokine receptor type 4	T96079	Melanoma [ICD-11: 2C30]	81.43%	93.1%
C-X-C chemokine receptor type 4	T96079	Merkel cell carcinoma [ICD-11: 2C34]	81.43%	93.1%
C-X-C chemokine receptor type 4	T96079	Multiple myeloma [ICD-11: 2A83]	81.43%	93.1%
C-X-C chemokine receptor type 4	T96079	Myelodysplastic syndrome [ICD-11: 2A37]	81.43%	93.1%
C-X-C chemokine receptor type 4	T96079	Non-hodgkin lymphoma [ICD-11: 2B33.5]	81.43%	93.1%

Target Name	ChEMBL-ID	Indication	Probability	Model accuracy
C-X-C chemokine receptor type 4	T96079	Pancreatic cancer [ICD-11: 2C10]	81.43%	93.1%
C-X-C chemokine receptor type 4	T96079	Peripheral vascular disease [ICD-11: BD4Z]	81.43%	93.1%
C-X-C chemokine receptor type 4	T96079	Psoriasis vulgaris [ICD-11: EA90]	81.43%	93.1%
C-X-C chemokine receptor type 4	T96079	Recurring respiratory infection [ICD-11: CA07-CA45]	81.43%	93.1%
C-X-C chemokine receptor type 4	T96079	Renal cell carcinoma [ICD-11: 2C90]	81.43%	93.1%
C-X-C chemokine receptor type 4	T96079	Sarcoma [ICD-11: 2A60-2C35]	81.43%	93.1%
C-X-C chemokine receptor type 4	T96079	Solid tumour/cancer [ICD-11: 2A00-2F9Z]	81.43%	93.1%
Acetylcholinesterase	T30082	Alcohol dependence [ICD-11: 6C40.2]	79.85%	94.45%
Acetylcholinesterase	T30082	Alzheimer disease [ICD-11: 8A20]	79.85%	94.45%
Acetylcholinesterase	T30082	Chronic glaucoma [ICD-11: 9C61.0Z]	79.85%	94.45%
Acetylcholinesterase	T30082	Cognitive impairment [ICD-11: 6D71]	79.85%	94.45%
Acetylcholinesterase	T30082	Dementia [ICD-11: 6D80-6D86]	79.85%	94.45%
Acetylcholinesterase	T30082	Functional dyspepsia [ICD-11: DD90.3]	79.85%	94.45%
Acetylcholinesterase	T30082	Helminth infection [ICD-11: 1F90.0]	79.85%	94.45%
Acetylcholinesterase	T30082	Myasthenia gravis [ICD-11: 8C6Y]	79.85%	94.45%
Acetylcholinesterase	T30082	Neurodegenerative disorder [ICD-11: 8A20-8A23]	79.85%	94.45%
Acetylcholinesterase	T30082	Open-angle glaucoma [ICD-11: 9C61]	79.85%	94.45%
Acetylcholinesterase	T30082	Pain [ICD-11: MG30-MG3Z]	79.85%	94.45%
Acetylcholinesterase	T30082	Parkinson disease [ICD-11: 8A00.0]	79.85%	94.45%
Acetylcholinesterase	T30082	Pediculus capitis infestation [ICD-11: 1G00.0]	79.85%	94.45%
Acetylcholinesterase	T30082	Poisoning due to pesticides and chemicals [ICD-11: NE6Z]	79.85%	94.45%
Acetylcholinesterase	T30082	Solid tumour/cancer [ICD-11: 2A00-2F9Z]	79.85%	94.45%
Acetylcholinesterase	T30082	Urinary dysfunction [ICD-11: GC2Z]	79.85%	94.45%
Acetylcholinesterase	T30082	Xerostomia [ICD-11: DA02.1]	79.85%	94.45%
Glycine receptor subunit alpha-1	T50269	Muscle spasm [ICD-11: MB47.3]	79.52%	90.71%
Aurora kinase B/Inner centromere protein	T46781	Acute myeloid leukaemia [ICD-11: 2A60]	77.98%	97.5%
Aurora kinase B/Inner centromere protein	T46781	Diabetic complication [ICD-11: 5A2Y]	77.98%	97.5%
Aurora kinase B/Inner centromere protein	T46781	Haematological malignancy [ICD-11: 2B33.Y]	77.98%	97.5%
Aurora kinase B/Inner centromere protein	T46781	Prostate cancer [ICD-11: 2C82.0]	77.98%	97.5%
Aurora kinase B/Inner centromere protein	T46781	Solid tumour/cancer [ICD-11: 2A00-2F9Z]	77.98%	97.5%
Nicotinamide phosphoribosyltransferase	T54582	leukaemia [ICD-11: 2A60-2B33]	77.87%	96.25%
Nicotinamide phosphoribosyltransferase	T54582	Solid tumour/cancer [ICD-11: 2A00-2F9Z]	77.87%	96.25%

Target Name	ChEMBL-ID	Indication	Probability	Model accuracy
G-protein coupled bile acid receptor 1	T86273	Metabolic disorder [ICD-11: 5C50-5D2Z]	77.73%	93.65%
G-protein coupled bile acid receptor 1	T86273	Type-2 diabetes [ICD-11: 5A11]	77.73%	93.65%
Protein-tyrosine phosphatase 2C	T13057	Solid tumour/cancer [ICD-11: 2A00-2F9Z]	77.39%	94.42%
DNA topoisomerase II alpha	T17048	Solid tumour/cancer [ICD-11: 2A00-2F9Z]	76.03%	89%
TGF-beta receptor type II	T49989	Solid tumour/cancer [ICD-11: 2A00-2F9Z]	75.74%	88.18%
TGF-beta receptor type II	T49989	Wound healing [ICD-11: EL8Y]	75.74%	88.18%
PI3-kinase p110-alpha/p85-alpha	T80276	Breast cancer [ICD-11: 2C60-2C65]	75.14%	94.33%
PI3-kinase p110-alpha/p85-alpha	T80276	Follicular lymphoma [ICD-11: 2A80]	75.14%	94.33%
PI3-kinase p110-alpha/p85-alpha	T80276	Non-hodgkin lymphoma [ICD-11: 2B33.5]	75.14%	94.33%
PI3-kinase p110-alpha/p85-alpha	T80276	Prostate cancer [ICD-11: 2C82.0]	75.14%	94.33%
PI3-kinase p110-alpha/p85-alpha	T80276	Solid tumour/cancer [ICD-11: 2A00-2F9Z]	75.14%	94.33%
Cysteinyl leukotriene receptor 2	T74238	Asthma [ICD-11: CA23]	73.66%	98%
Sphingosine 1-phosphate receptor Edg-8	T50089	Multiple sclerosis [ICD-11: 8A40]	73.58%	100%
Histone deacetylase 4	T63816	Advanced stage follicular lymphoma [ICD-11: 2A80]	72.57%	92.97%
Histone deacetylase 4	T63816	Huntington disease [ICD-11: 8A01.10]	72.57%	92.97%
Histone deacetylase 4	T63816	Solid tumour/cancer [ICD-11: 2A00-2F9Z]	72.57%	92.97%
C5a anaphylatoxin chemotactic receptor	T15439	Atopic dermatitis [ICD-11: EA80]	71.96%	92.62%
C5a anaphylatoxin chemotactic receptor	T15439	Eosinophilic granulomatosis with polyangiitis [ICD-11: 4A44.A2]	71.96%	92.62%
C5a anaphylatoxin chemotactic receptor	T15439	Hereditary haemolytic anemia [ICD-11: 3A10.3]	71.96%	92.62%
C5a anaphylatoxin chemotactic receptor	T15439	Inflammation [ICD-11: 1A00-CA43.1]	71.96%	92.62%
C5a anaphylatoxin chemotactic receptor	T15439	Rheumatoid arthritis [ICD-11: FA20]	71.96%	92.62%
C5a anaphylatoxin chemotactic receptor	T15439	Vasculitis [ICD-11: 4A44]	71.96%	92.62%
Tyrosine-protein kinase receptor UFO	T82383	Acute myeloid leukaemia [ICD-11: 2A60]	70.87%	90.95%
Tyrosine-protein kinase receptor UFO	T82383	Breast cancer [ICD-11: 2C60-2C65]	70.87%	90.95%
Tyrosine-protein kinase receptor UFO	T82383	Metastatic malignant neoplasm [ICD-11: 2D50-2E09]	70.87%	90.95%
Tyrosine-protein kinase receptor UFO	T82383	Multiple myeloma [ICD-11: 2A83]	70.87%	90.95%
Tyrosine-protein kinase receptor UFO	T82383	Myelodysplastic syndrome [ICD-11: 2A37]	70.87%	90.95%
Tyrosine-protein kinase receptor UFO	T82383	Non-small-cell lung cancer [ICD-11: 2C25.Y]	70.87%	90.95%
Tyrosine-protein kinase receptor UFO	T82383	Solid tumour/cancer [ICD-11: 2A00-2F9Z]	70.87%	90.95%
Sodium channel protein type IV alpha subunit	T02546	Musculoskeletal disorder [ICD-11: FA00-FC0Z]	70.41%	92.32%

Target Name	ChEMBL-ID	Indication	Probability	Model accuracy
Sodium channel protein type IV alpha subunit	T02546	Neurological disorder [ICD-11: 6B60]	70.41%	92.32%
Sodium channel protein type IV alpha subunit	T02546	Pain [ICD-11: MG30-MG3Z]	70.41%	92.32%
Histone deacetylase 10	T94324	Solid tumour/cancer [ICD-11: 2A00-2F9Z]	70.36%	90.08%
Signal transducer and activator of transcription 3	T29130	Brain cancer [ICD-11: 2A00]	70.33%	82.69%
Signal transducer and activator of transcription 3	T29130	Chronic lymphocytic leukaemia [ICD-11: 2A82.0]	70.33%	82.69%
Signal transducer and activator of transcription 3	T29130	Hepatocellular carcinoma [ICD-11: 2C12.02]	70.33%	82.69%
Signal transducer and activator of transcription 3	T29130	Immune System disease [ICD-11: 4A01-4B41]	70.33%	82.69%
Signal transducer and activator of transcription 3	T29130	Inflammation [ICD-11: 1A00-CA43.1]	70.33%	82.69%
Signal transducer and activator of transcription 3	T29130	Multiple myeloma [ICD-11: 2A83]	70.33%	82.69%
Signal transducer and activator of transcription 3	T29130	Psoriasis vulgaris [ICD-11: EA90]	70.33%	82.69%
Signal transducer and activator of transcription 3	T29130	Recurrent glioblastoma [ICD-11: 2A00.00]	70.33%	82.69%
Signal transducer and activator of transcription 3	T29130	Solid tumour/cancer [ICD-11: 2A00-2F9Z]	70.33%	82.69%
Signal transducer and activator of transcription 3	T29130	Ulcerative colitis [ICD-11: DD71]	70.33%	82.69%
Pregnane X receptor	T82702	Arteriosclerosis [ICD-11: BD40]	70.03%	94.73%
Amine oxidase, copper containing	T69619	Asthma [ICD-11: CA23]	69.78%	94%
Amine oxidase, copper containing	T69619	Atopic dermatitis [ICD-11: EA80]	69.78%	94%
Amine oxidase, copper containing	T69619	Central nervous system disease [ICD-11: 8A04-8D87]	69.78%	94%
Amine oxidase, copper containing	T69619	Cerebrovascular ischaemia [ICD-11: 8B1Z]	69.78%	94%
Amine oxidase, copper containing	T69619	Diabetic complication [ICD-11: 5A2Y]	69.78%	94%
Amine oxidase, copper containing	T69619	Diabetic macular edema [ICD-11: 9B71.02]	69.78%	94%
Amine oxidase, copper containing	T69619	Diabetic nephropathy [ICD-11: GB61.Z]	69.78%	94%
Amine oxidase, copper containing	T69619	Hypertension [ICD-11: BA00-BA04]	69.78%	94%
Amine oxidase, copper containing	T69619	Inflammation [ICD-11: 1A00-CA43.1]	69.78%	94%
Amine oxidase, copper containing	T69619	Psoriasis vulgaris [ICD-11: EA90]	69.78%	94%
Amine oxidase, copper containing	T69619	Pulmonary disease [ICD-11: 1B10-1F85]	69.78%	94%
Amine oxidase, copper containing	T69619	Vascular disease [ICD-11: BE2Z]	69.78%	94%
Rho-associated protein kinase 1	T51282	Arteriosclerosis [ICD-11: BD40]	69.69%	95.55%
Rho-associated protein kinase 1	T51282	Chronic obstructive pulmonary disease [ICD-11: CA22]	69.69%	95.55%
Rho-associated protein kinase 1	T51282	Diabetic nephropathy [ICD-11: GB61.Z]	69.69%	95.55%
Rho-associated protein kinase 1	T51282	Inflammation [ICD-11: 1A00-	69.69%	95.55%

Target Name	ChEMBL-ID	Indication	Probability	Model accuracy
		CA43.1]		
Rho-associated protein kinase 1	T51282	Microvascular angina [ICD-11: BA86]	69.69%	95.55%
Rho-associated protein kinase 1	T51282	Open-angle glaucoma [ICD-11: 9C61]	69.69%	95.55%
Rho-associated protein kinase 1	T51282	Pain [ICD-11: MG30-MG3Z]	69.69%	95.55%
Rho-associated protein kinase 1	T51282	Pulmonary arterial hypertension [ICD-11: BB01.0]	69.69%	95.55%
Rho-associated protein kinase 1	T51282	Solid tumour/cancer [ICD-11: 2A00-2F9Z]	69.69%	95.55%
Toll-like receptor 4	T81443	Allergy [ICD-11: 4A80-4A85]	69.62%	92.5%
Toll-like receptor 4	T81443	Autoimmune diabetes [ICD-11: 5A10]	69.62%	92.5%
Toll-like receptor 4	T81443	Hepatitis virus infection [ICD-11: 1E50-1E51]	69.62%	92.5%
Toll-like receptor 4	T81443	Melanoma [ICD-11: 2C30]	69.62%	92.5%
Toll-like receptor 4	T81443	Non-hodgkin lymphoma [ICD-11: 2B33.5]	69.62%	92.5%
Toll-like receptor 4	T81443	Prostate cancer [ICD-11: 2C82.0]	69.62%	92.5%
Toll-like receptor 4	T81443	Sepsis [ICD-11: 1G40-1G41]	69.62%	92.5%
Toll-like receptor 4	T81443	Solid tumour/cancer [ICD-11: 2A00-2F9Z]	69.62%	92.5%
ADAM10	T31902	Breast cancer [ICD-11: 2C60-2C65]	68.49%	97.5%
ADAM10	T31902	Solid tumour/cancer [ICD-11: 2A00-2F9Z]	68.49%	97.5%
Glutamate receptor ionotropic, AMPA 2	T42392	Diabetic neuropathy [ICD-11: 8C0Z]	68.22%	86.92%
Glutamate receptor ionotropic, AMPA 2	T42392	Parkinson disease [ICD-11: 8A00.0]	68.22%	86.92%
11-beta-hydroxysteroid dehydrogenase 2	T43721	Type-2 diabetes [ICD-11: 5A11]	68.02%	94.78%
Serotonin 7 (5-HT7) receptor	T79062	Alzheimer disease [ICD-11: 8A20]	66.79%	90.71%
Serotonin 7 (5-HT7) receptor	T79062	Attention deficit hyperactivity disorder [ICD-11: 6A05.Z]	66.79%	90.71%
Serotonin 7 (5-HT7) receptor	T79062	Bipolar disorder [ICD-11: 6A60]	66.79%	90.71%
Serotonin 7 (5-HT7) receptor	T79062	Major depressive disorder [ICD-11: 6A70.3]	66.79%	90.71%
Serotonin 7 (5-HT7) receptor	T79062	Parkinson disease [ICD-11: 8A00.0]	66.79%	90.71%
Serotonin 7 (5-HT7) receptor	T79062	Psychiatric disorder [ICD-11: 6E8Z]	66.79%	90.71%
Serotonin 7 (5-HT7) receptor	T79062	Schizophrenia [ICD-11: 6A20]	66.79%	90.71%
Serotonin 7 (5-HT7) receptor	T79062	Sleep-wake disorder [ICD-11: 7A00-7B2Z]	66.79%	90.71%
Platelet-derived growth factor receptor alpha	T53524	Acute myelogenous leukaemia [ICD-11: 2A41]	65.62%	91.07%
Platelet-derived growth factor receptor alpha	T53524	Acute myeloid leukaemia [ICD-11: 2A60]	65.62%	91.07%
Platelet-derived growth factor receptor alpha	T53524	Breast cancer [ICD-11: 2C60-2C65]	65.62%	91.07%
Platelet-derived growth factor receptor alpha	T53524	Colon cancer [ICD-11: 2B90.Z]	65.62%	91.07%
Platelet-derived growth factor	T53524	Colorectal cancer [ICD-11: 2B91.Z]	65.62%	91.07%

Target Name	ChEMBL-ID	Indication	Probability	Model accuracy
receptor alpha				
Platelet-derived growth factor receptor alpha	T53524	Gastric adenocarcinoma [ICD-11: 2B72]	65.62%	91.07%
Platelet-derived growth factor receptor alpha	T53524	Gastrointestinal cancer [ICD-11: 2C11]	65.62%	91.07%
Platelet-derived growth factor receptor alpha	T53524	Gastrointestinal stromal tumour [ICD-11: 2B5B]	65.62%	91.07%
Platelet-derived growth factor receptor alpha	T53524	Glioblastoma multiforme [ICD-11: 2A00.0]	65.62%	91.07%
Platelet-derived growth factor receptor alpha	T53524	Gram-positive bacterial infection [ICD-11: 1B74-1G40]	65.62%	91.07%
Platelet-derived growth factor receptor alpha	T53524	Lung cancer [ICD-11: 2C25.0]	65.62%	91.07%
Platelet-derived growth factor receptor alpha	T53524	Mast cell leukaemia [ICD-11: 2A21.00]	65.62%	91.07%
Platelet-derived growth factor receptor alpha	T53524	Multiple myeloma [ICD-11: 2A83]	65.62%	91.07%
Platelet-derived growth factor receptor alpha	T53524	Myelodysplastic syndrome [ICD-11: 2A37]	65.62%	91.07%
Platelet-derived growth factor receptor alpha	T53524	Non-small-cell lung cancer [ICD-11: 2C25.Y]	65.62%	91.07%
Platelet-derived growth factor receptor alpha	T53524	Pancreatic cancer [ICD-11: 2C10]	65.62%	91.07%
Platelet-derived growth factor receptor alpha	T53524	Prostate cancer [ICD-11: 2C82.0]	65.62%	91.07%
Platelet-derived growth factor receptor alpha	T53524	Recurrent glioblastoma [ICD-11: 2A00.00]	65.62%	91.07%
Platelet-derived growth factor receptor alpha	T53524	Soft tissue sarcoma [ICD-11: 2B57]	65.62%	91.07%
Platelet-derived growth factor receptor alpha	T53524	Solid tumour/cancer [ICD-11: 2A00-2F9Z]	65.62%	91.07%
Platelet-derived growth factor receptor alpha	T53524	Systemic mastocytosis [ICD-11: 2A21.0]	65.62%	91.07%
Platelet-derived growth factor receptor alpha	T53524	Thrombocytopenia [ICD-11: 3B64]	65.62%	91.07%
Transient receptor potential cation channel subfamily A member 1	T84040	Diabetic neuropathy [ICD-11: 8C0Z]	65.59%	92.17%
Transient receptor potential cation channel subfamily A member 1	T84040	Pain [ICD-11: MG30-MG3Z]	65.59%	92.17%
Transient receptor potential cation channel subfamily A member 1	T84040	Throat irritation [ICD-11: CA0Y]	65.59%	92.17%
Phosphodiesterase 7A	T39523	Pain [ICD-11: MG30-MG3Z]	65.43%	99.29%
Adenosine A2b receptor	T86679	Acute and chronic heart failure [ICD-11: BD1Z]	65.14%	98.59%
Adenosine A2b receptor	T86679	Alzheimer disease [ICD-11: 8A20]	65.14%	98.59%
Adenosine A2b receptor	T86679	Apnea [ICD-11: MD11.0]	65.14%	98.59%
Adenosine A2b receptor	T86679	Asthma [ICD-11: CA23]	65.14%	98.59%
Adenosine A2b receptor	T86679	Herpes simplex virus infection [ICD-11: 1F00]	65.14%	98.59%
Adenosine A2b receptor	T86679	Hypertension [ICD-11: BA00-BA04]	65.14%	98.59%
Adenosine A2b receptor	T86679	Multiple sclerosis [ICD-11: 8A40]	65.14%	98.59%
Adenosine A2b receptor	T86679	Non-insulin dependent diabetes	65.14%	98.59%

Target Name	ChEMBL-ID	Indication	Probability	Model accuracy
		[ICD-11: 5A11]		
Adenosine A2b receptor	T86679	Non-small-cell lung cancer [ICD-11: 2C25.Y]	65.14%	98.59%
Adenosine A2b receptor	T86679	Parkinson disease [ICD-11: 8A00.0]	65.14%	98.59%
Adenosine A2b receptor	T86679	Paroxysmal supraventricular tachycardia [ICD-11: BC81.Z]	65.14%	98.59%
Adenosine A2b receptor	T86679	Solid tumour/cancer [ICD-11: 2A00-2F9Z]	65.14%	98.59%
Histone deacetylase 8	T28887	Solid tumour/cancer [ICD-11: 2A00-2F9Z]	64.2%	93.99%
Cytochrome P450 3A4	T37848	Atopic dermatitis [ICD-11: EA80]	63.5%	91.19%
Cytochrome P450 3A4	T37848	Solid tumour/cancer [ICD-11: 2A00-2F9Z]	63.5%	91.19%
DNA-(apurinic or apyrimidinic site) lyase	T13348	Glioma [ICD-11: 2A00.0]	63.49%	91.11%
DNA-(apurinic or apyrimidinic site) lyase	T13348	Melanoma [ICD-11: 2C30]	63.49%	91.11%
DNA-(apurinic or apyrimidinic site) lyase	T13348	Ocular cancer [ICD-11: 2D00-2D07]	63.49%	91.11%
DNA-(apurinic or apyrimidinic site) lyase	T13348	Solid tumour/cancer [ICD-11: 2A00-2F9Z]	63.49%	91.11%
Methionine aminopeptidase 2	T75596	Autoimmune diabetes [ICD-11: 5A10]	61.97%	97.28%
Methionine aminopeptidase 2	T75596	Obesity [ICD-11: 5B81]	61.97%	97.28%
Methionine aminopeptidase 2	T75596	Solid tumour/cancer [ICD-11: 2A00-2F9Z]	61.97%	97.28%
Ghrelin receptor	T59604	Alcohol dependence [ICD-11: 6C40.2]	61.94%	92%
Ghrelin receptor	T59604	Anorexia nervosa cachexia [ICD-11: 6B80]	61.94%	92%
Ghrelin receptor	T59604	Cachexia [ICD-11: MG20]	61.94%	92%
Ghrelin receptor	T59604	Carbohydrate metabolism disorder [ICD-11: 5C51.Z]	61.94%	92%
Ghrelin receptor	T59604	Fibromyalgia [ICD-11: MG30.01]	61.94%	92%
Ghrelin receptor	T59604	Frailty [ICD-11: MG2A]	61.94%	92%
Ghrelin receptor	T59604	Functional bowel disorder [ICD-11: DD91]	61.94%	92%
Ghrelin receptor	T59604	Gastric motility disorder [ICD-11: DA21]	61.94%	92%
Ghrelin receptor	T59604	Gastrointestinal disease [ICD-11: DE2Z]	61.94%	92%
Ghrelin receptor	T59604	Gastroparesis [ICD-11: DA41.00]	61.94%	92%
Ghrelin receptor	T59604	Growth hormone deficiency [ICD-11: 5A61.3]	61.94%	92%
Ghrelin receptor	T59604	Ileus [ICD-11: DA93.0]	61.94%	92%
Ghrelin receptor	T59604	Metabolic disorder [ICD-11: 5C50-5D2Z]	61.94%	92%
Ghrelin receptor	T59604	Obesity [ICD-11: 5B81]	61.94%	92%
Ghrelin receptor	T59604	Postoperative ileus [ICD-11: DA91-DB30]	61.94%	92%
Ghrelin receptor	T59604	Solid tumour/cancer [ICD-11:	61.94%	92%

Target Name	ChEMBL-ID	Indication	Probability	Model accuracy
		2A00-2F9Z]		
Ghrelin receptor	T59604	Somatotropin deficiency [ICD-11: 5A61.0]	61.94%	92%
Ghrelin receptor	T59604	Type-2 diabetes [ICD-11: 5A11]	61.94%	92%
PI3-kinase p110-delta subunit	T67849	Arthritis [ICD-11: FA20]	61.78%	96.47%
PI3-kinase p110-delta subunit	T67849	Breast cancer [ICD-11: 2C60-2C65]	61.78%	96.47%
PI3-kinase p110-delta subunit	T67849	Chronic lymphocytic leukaemia [ICD-11: 2A82.0]	61.78%	96.47%
PI3-kinase p110-delta subunit	T67849	Coronavirus Disease 2019 (COVID-19) [ICD-11: 1D6Y]	61.78%	96.47%
PI3-kinase p110-delta subunit	T67849	Cutaneous T-cell lymphoma [ICD-11: 2B01]	61.78%	96.47%
PI3-kinase p110-delta subunit	T67849	Diffuse large B-cell lymphoma [ICD-11: 2A81]	61.78%	96.47%
PI3-kinase p110-delta subunit	T67849	Follicular lymphoma [ICD-11: 2A80]	61.78%	96.47%
PI3-kinase p110-delta subunit	T67849	Follicular lymphoma [ICD-11: 2A80]	61.78%	96.47%
PI3-kinase p110-delta subunit	T67849	Hematologic tumour [ICD-11: 2B33.Y]	61.78%	96.47%
PI3-kinase p110-delta subunit	T67849	Lymphoma [ICD-11: 2A80-2A86]	61.78%	96.47%
PI3-kinase p110-delta subunit	T67849	Mantle cell lymphoma [ICD-11: 2A85.5]	61.78%	96.47%
PI3-kinase p110-delta subunit	T67849	Marginal zone lymphoma [ICD-11: 2A85.0]	61.78%	96.47%
PI3-kinase p110-delta subunit	T67849	Myeloid leukaemia [ICD-11: 2B33.1]	61.78%	96.47%
PI3-kinase p110-delta subunit	T67849	Non-hodgkin lymphoma [ICD-11: 2B33.5]	61.78%	96.47%
PI3-kinase p110-delta subunit	T67849	Solid tumour/cancer [ICD-11: 2A00-2F9Z]	61.78%	96.47%
DNA topoisomerase I	T09826	Acquired immune deficiency syndrome [ICD-11: 1C62.3]	61.45%	97%
DNA topoisomerase I	T09826	Acute lymphoblastic leukaemia [ICD-11: 2A85]	61.45%	97%
DNA topoisomerase I	T09826	Bacterial infection [ICD-11: 1A00-1C4Z]	61.45%	97%
DNA topoisomerase I	T09826	Bladder cancer [ICD-11: 2C94]	61.45%	97%
DNA topoisomerase I	T09826	Breast cancer [ICD-11: 2C60-2C65]	61.45%	97%
DNA topoisomerase I	T09826	Colorectal cancer [ICD-11: 2B91.Z]	61.45%	97%
DNA topoisomerase I	T09826	Esophageal cancer [ICD-11: 2B70]	61.45%	97%
DNA topoisomerase I	T09826	Gastric adenocarcinoma [ICD-11: 2B72]	61.45%	97%
DNA topoisomerase I	T09826	Glioblastoma multiforme [ICD-11: 2A00.0]	61.45%	97%
DNA topoisomerase I	T09826	Human immunodeficiency virus infection [ICD-11: 1C62]	61.45%	97%
DNA topoisomerase I	T09826	Lung cancer [ICD-11: 2C25.0]	61.45%	97%
DNA topoisomerase I	T09826	Lymphoma [ICD-11: 2A80-2A86]	61.45%	97%
DNA topoisomerase I	T09826	Metastatic colorectal cancer [ICD-11: 2B91]	61.45%	97%
DNA topoisomerase I	T09826	Myelodysplastic syndrome [ICD-11: 2A37]	61.45%	97%
DNA topoisomerase I	T09826	Ovarian cancer [ICD-11: 2C73]	61.45%	97%

Target Name	ChEMBL-ID	Indication	Probability	Model accuracy
DNA topoisomerase I	T09826	Renal cell carcinoma [ICD-11: 2C90]	61.45%	97%
DNA topoisomerase I	T09826	Small-cell lung cancer [ICD-11: 2C25.Y]	61.45%	97%
DNA topoisomerase I	T09826	Solid tumour/cancer [ICD-11: 2A00-2F9Z]	61.45%	97%
Multidrug resistance-associated protein 1	T11288	Gout [ICD-11: FA25]	60.66%	96.37%
Peptidyl-prolyl cis-trans isomerase NIMA-interacting 1	T16308	Nasopharyngeal carcinoma [ICD-11: 2B6B]	59.89%	91.71%
Peptidyl-prolyl cis-trans isomerase NIMA-interacting 1	T16308	Non-small-cell lung cancer [ICD-11: 2C25.Y]	59.89%	91.71%
Integrin alpha-5/beta-1	T29879	Allergic rhinitis [ICD-11: CA08.0]	59.63%	96.39%
Integrin alpha-5/beta-1	T29879	Allergy [ICD-11: 4A80-4A85]	59.63%	96.39%
Integrin alpha-5/beta-1	T29879	Asthma [ICD-11: CA23]	59.63%	96.39%
Integrin alpha-5/beta-1	T29879	Macular degeneration [ICD-11: 9B78.3]	59.63%	96.39%
Integrin alpha-5/beta-1	T29879	Macular degeneration [ICD-11: 9B78.3]	59.63%	96.39%
Integrin alpha-5/beta-1	T29879	Multiple sclerosis [ICD-11: 8A40]	59.63%	96.39%
Integrin alpha-5/beta-1	T29879	Non-small-cell lung cancer [ICD-11: 2C25.Y]	59.63%	96.39%
Serotonin 2c (5-HT2c) receptor	T83813	Alcohol dependence [ICD-11: 6C40.2]	58.41%	89.62%
Serotonin 2c (5-HT2c) receptor	T83813	Alzheimer disease [ICD-11: 8A20]	58.41%	89.62%
Serotonin 2c (5-HT2c) receptor	T83813	Anxiety disorder [ICD-11: 6B00-6B0Z]	58.41%	89.62%
Serotonin 2c (5-HT2c) receptor	T83813	Attention deficit hyperactivity disorder [ICD-11: 6A05.Z]	58.41%	89.62%
Serotonin 2c (5-HT2c) receptor	T83813	Depression [ICD-11: 6A70-6A7Z]	58.41%	89.62%
Serotonin 2c (5-HT2c) receptor	T83813	Diabetic complication [ICD-11: 5A2Y]	58.41%	89.62%
Serotonin 2c (5-HT2c) receptor	T83813	Drug abuse [ICD-11: 6C4G.1Z]	58.41%	89.62%
Serotonin 2c (5-HT2c) receptor	T83813	Dyskinesia [ICD-11: MB47.4]	58.41%	89.62%
Serotonin 2c (5-HT2c) receptor	T83813	Generalized anxiety disorder [ICD-11: 6B00]	58.41%	89.62%
Serotonin 2c (5-HT2c) receptor	T83813	Hyperprolactinaemia [ICD-11: 5A60.1]	58.41%	89.62%
Serotonin 2c (5-HT2c) receptor	T83813	Major depressive disorder [ICD-11: 6A70.3]	58.41%	89.62%
Serotonin 2c (5-HT2c) receptor	T83813	Metabolic disorder [ICD-11: 5C50-5D2Z]	58.41%	89.62%
Serotonin 2c (5-HT2c) receptor	T83813	Migraine [ICD-11: 8A80]	58.41%	89.62%
Serotonin 2c (5-HT2c) receptor	T83813	Mood disorder [ICD-11: 6A60-6E23]	58.41%	89.62%
Serotonin 2c (5-HT2c) receptor	T83813	Neurological disorder [ICD-11: 6B60]	58.41%	89.62%
Serotonin 2c (5-HT2c) receptor	T83813	Non-hodgkin lymphoma [ICD-11: 2B33.5]	58.41%	89.62%
Serotonin 2c (5-HT2c) receptor	T83813	Obesity [ICD-11: 5B81]	58.41%	89.62%
Serotonin 2c (5-HT2c) receptor	T83813	Pain [ICD-11: MG30-MG3Z]	58.41%	89.62%
Serotonin 2c (5-HT2c) receptor	T83813	Parkinson disease [ICD-11: 8A00.0]	58.41%	89.62%
Serotonin 2c (5-HT2c) receptor	T83813	Primary insomnia [ICD-11: 7A00]	58.41%	89.62%

Target Name	ChEMBL-ID	Indication	Probability	Model accuracy
Serotonin 2c (5-HT2c) receptor	T83813	Psychotic disorder [ICD-11: 6A20-6A25]	58.41%	89.62%
Serotonin 2c (5-HT2c) receptor	T83813	Schizophrenia [ICD-11: 6A20]	58.41%	89.62%
Serotonin 2c (5-HT2c) receptor	T83813	Sleep-wake disorder [ICD-11: 7A00-7B2Z]	58.41%	89.62%
Nitric-oxide synthase, endothelial	T06046	Angina pectoris [ICD-11: BA40]	58.21%	86%
Nitric-oxide synthase, endothelial	T06046	Brain injury [ICD-11: NA07.Z]	58.21%	86%
Nitric-oxide synthase, endothelial	T06046	Coronary artery disease [ICD-11: BA80]	58.21%	86%
Nitric-oxide synthase, endothelial	T06046	Hypertension [ICD-11: BA00-BA04]	58.21%	86%
Nitric-oxide synthase, endothelial	T06046	Hypotension [ICD-11: BA20-BA21]	58.21%	86%
Nitric-oxide synthase, endothelial	T06046	Myocardial infarction [ICD-11: BA41-BA43]	58.21%	86%
Nitric-oxide synthase, endothelial	T06046	Pulmonary hypertension [ICD-11: BB01]	58.21%	86%
Cyclin-dependent kinase 5/CDK5 activator 1	T20973	Obesity [ICD-11: 5B81]	57.54%	93.03%
Glutathione S-transferase Pi	T21669	Myelodysplastic syndrome [ICD-11: 2A37]	53.75%	93.81%
Glutathione S-transferase Pi	T21669	Solid tumour/cancer [ICD-11: 2A00-2F9Z]	53.75%	93.81%
Tissue-type plasminogen activator	T45299	Bleeding disorder [ICD-11: GA20-GA21]	52.6%	93.33%
Tissue-type plasminogen activator	T45299	Postoperative hemorrhage [ICD-11: NE81.0Z]	52.6%	93.33%
Thyroid hormone receptor alpha	T79591	Congestive heart failure [ICD-11: BD10]	52.15%	99.15%
Thyroid hormone receptor alpha	T79591	High blood cholesterol level [ICD-11: 5C80.00]	52.15%	99.15%
Thyroid hormone receptor alpha	T79591	Hypothyroidism [ICD-11: 5A00]	52.15%	99.15%
Thyroid hormone receptor alpha	T79591	Wound healing [ICD-11: EL8Y]	52.15%	99.15%
Signal transducer and activator of transcription 1-alpha/beta	T64205	Skin infection [ICD-11: 1F28-1G0Z]	51.96%	72.62%
Glutaminase kidney isoform, mitochondrial	T86734	Breast cancer [ICD-11: 2C60-2C65]	51.51%	100%
Glutaminase kidney isoform, mitochondrial	T86734	leukaemia [ICD-11: 2A60-2B33]	51.51%	100%
Glutaminase kidney isoform, mitochondrial	T86734	Renal cell carcinoma [ICD-11: 2C90]	51.51%	100%
Glutaminase kidney isoform, mitochondrial	T86734	Solid tumour/cancer [ICD-11: 2A00-2F9Z]	51.51%	100%
Hexokinase type IV	T87166	Diabetic complication [ICD-11: 5A2Y]	51.3%	91.96%
Hexokinase type IV	T87166	Obesity [ICD-11: 5B81]	51.3%	91.96%
Hexokinase type IV	T87166	Type-2 diabetes [ICD-11: 5A11]	51.3%	91.96%
Kappa opioid receptor	T60693	Alcohol dependence [ICD-11: 6C40.2]	50.71%	98.1%
Kappa opioid receptor	T60693	Bipolar disorder [ICD-11: 6A60]	50.71%	98.1%
Kappa opioid receptor	T60693	Cerebral vasospasm [ICD-11: BA85.Z]	50.71%	98.1%
Kappa opioid receptor	T60693	Chronic pain [ICD-11: MG30]	50.71%	98.1%

Target Name	ChEMBL-ID	Indication	Probability	Model accuracy
Kappa opioid receptor	T60693	Diarrhea-predominant irritable bowel syndrome [ICD-11: DD91.01]	50.71%	98.1%
Kappa opioid receptor	T60693	Erythema [ICD-11: ME64.0]	50.71%	98.1%
Kappa opioid receptor	T60693	Inflammatory bowel disease [ICD-11: DD72]	50.71%	98.1%
Kappa opioid receptor	T60693	Major depressive disorder [ICD-11: 6A70.3]	50.71%	98.1%
Kappa opioid receptor	T60693	Multiple myeloma [ICD-11: 2A83]	50.71%	98.1%
Kappa opioid receptor	T60693	Neurodegenerative disorder [ICD-11: 8A20-8A23]	50.71%	98.1%
Kappa opioid receptor	T60693	Obesity [ICD-11: 5B81]	50.71%	98.1%
Kappa opioid receptor	T60693	Opioid dependence [ICD-11: 6C43.2Z]	50.71%	98.1%
Kappa opioid receptor	T60693	Pain [ICD-11: MG30-MG3Z]	50.71%	98.1%
Kappa opioid receptor	T60693	Pruritus [ICD-11: EC90]	50.71%	98.1%
Kappa opioid receptor	T60693	Substance use disorder [ICD-11: 6C4Z]	50.71%	98.1%
Kappa opioid receptor	T60693	Uremic pruritus [ICD-11: EC90.10]	50.71%	98.1%
Nitric oxide synthase, inducible	T02703	Artery stenosis [ICD-11: BD52]	50.68%	94.8%
Nitric oxide synthase, inducible	T02703	Asthma [ICD-11: CA23]	50.68%	94.8%
Nitric oxide synthase, inducible	T02703	Cardiovascular disease [ICD-11: BA00-BE2Z]	50.68%	94.8%
Nitric oxide synthase, inducible	T02703	Coronary artery disease [ICD-11: BA80]	50.68%	94.8%
Nitric oxide synthase, inducible	T02703	Diabetic kidney disease [ICD-11: GB61.Z]	50.68%	94.8%
Nitric oxide synthase, inducible	T02703	Diabetic retinopathy [ICD-11: 9B71.0]	50.68%	94.8%
Nitric oxide synthase, inducible	T02703	Diarrhea [ICD-11: ME05.1]	50.68%	94.8%
Nitric oxide synthase, inducible	T02703	Endotoxic shock [ICD-11: 1G41]	50.68%	94.8%
Nitric oxide synthase, inducible	T02703	Inflammation [ICD-11: 1A00-CA43.1]	50.68%	94.8%
Nitric oxide synthase, inducible	T02703	Insulin-resistant disorder [ICD-11: 5A44]	50.68%	94.8%
Nitric oxide synthase, inducible	T02703	Osteoarthritis [ICD-11: FA00-FA05]	50.68%	94.8%
Nitric oxide synthase, inducible	T02703	Postoperative pain [ICD-11: MG30-MG3Z]	50.68%	94.8%
Nitric oxide synthase, inducible	T02703	Rheumatoid arthritis [ICD-11: FA20]	50.68%	94.8%
Nitric oxide synthase, inducible	T02703	Sepsis [ICD-11: 1G40-1G41]	50.68%	94.8%
Nitric oxide synthase, inducible	T02703	Solid tumour/cancer [ICD-11: 2A00-2F9Z]	50.68%	94.8%
Platelet-derived growth factor receptor	T53524	Acute myelogenous leukaemia [ICD-11: 2A41]	50.63%	71.67%
Platelet-derived growth factor receptor	T53524	Acute myeloid leukaemia [ICD-11: 2A60]	50.63%	71.67%
Platelet-derived growth factor receptor	T53524	Alzheimer disease [ICD-11: 8A20]	50.63%	71.67%
Platelet-derived growth factor receptor	T53524	Brain injury [ICD-11: NA07.Z]	50.63%	71.67%
Platelet-derived growth factor receptor	T53524	Breast cancer [ICD-11: 2C60-2C65]	50.63%	71.67%

Target Name	ChEMBL-ID	Indication	Probability	Model accuracy
Platelet-derived growth factor receptor	T53524	Colon cancer [ICD-11: 2B90.Z]	50.63%	71.67%
Platelet-derived growth factor receptor	T53524	Colorectal cancer [ICD-11: 2B91.Z]	50.63%	71.67%
Platelet-derived growth factor receptor	T53524	Diabetic complication [ICD-11: 5A2Y]	50.63%	71.67%
Platelet-derived growth factor receptor	T53524	Gastric adenocarcinoma [ICD-11: 2B72]	50.63%	71.67%
Platelet-derived growth factor receptor	T53524	Gastrointestinal cancer [ICD-11: 2C11]	50.63%	71.67%
Platelet-derived growth factor receptor	T53524	Gastrointestinal stromal tumour [ICD-11: 2B5B]	50.63%	71.67%
Platelet-derived growth factor receptor	T53524	Glioblastoma multiforme [ICD-11: 2A00.0]	50.63%	71.67%
Platelet-derived growth factor receptor	T53524	Gram-positive bacterial infection [ICD-11: 1B74-1G40]	50.63%	71.67%
Platelet-derived growth factor receptor	T53524	Gram-positive bacterial infection [ICD-11: 1B74-1G40]	50.63%	71.67%
Platelet-derived growth factor receptor	T53524	Hepatocellular carcinoma [ICD-11: 2C12.02]	50.63%	71.67%
Platelet-derived growth factor receptor	T53524	Lung cancer [ICD-11: 2C25.0]	50.63%	71.67%
Platelet-derived growth factor receptor	T53524	Mast cell leukaemia [ICD-11: 2A21.00]	50.63%	71.67%
Platelet-derived growth factor receptor	T53524	Multiple myeloma [ICD-11: 2A83]	50.63%	71.67%
Platelet-derived growth factor receptor	T53524	Multiple myeloma [ICD-11: 2A83]	50.63%	71.67%
Platelet-derived growth factor receptor	T53524	Myelodysplastic syndrome [ICD-11: 2A37]	50.63%	71.67%
Platelet-derived growth factor receptor	T53524	Myelodysplastic syndrome [ICD-11: 2A37]	50.63%	71.67%
Platelet-derived growth factor receptor	T53524	Non-small-cell lung cancer [ICD-11: 2C25.Y]	50.63%	71.67%
Platelet-derived growth factor receptor	T53524	Non-small-cell lung cancer [ICD-11: 2C25.Y]	50.63%	71.67%
Platelet-derived growth factor receptor	T53524	Pancreatic cancer [ICD-11: 2C10]	50.63%	71.67%
Platelet-derived growth factor receptor	T53524	Prostate cancer [ICD-11: 2C82.0]	50.63%	71.67%
Platelet-derived growth factor receptor	T53524	Recurrent glioblastoma [ICD-11: 2A00.00]	50.63%	71.67%
Platelet-derived growth factor receptor	T53524	Renal cell carcinoma [ICD-11: 2C90]	50.63%	71.67%
Platelet-derived growth factor receptor	T53524	Soft tissue sarcoma [ICD-11: 2B57]	50.63%	71.67%
Platelet-derived growth factor receptor	T53524	Solid tumour/cancer [ICD-11: 2A00-2F9Z]	50.63%	71.67%
Platelet-derived growth factor receptor	T53524	Solid tumour/cancer [ICD-11: 2A00-2F9Z]	50.63%	71.67%
Platelet-derived growth factor receptor	T53524	Systemic mastocytosis [ICD-11: 2A21.0]	50.63%	71.67%
Platelet-derived growth factor	T53524	Thrombocytopenia [ICD-11: 3B64]	50.63%	71.67%

Target Name	ChEMBL-ID	Indication	Probability	Model accuracy
receptor				
Platelet-derived growth factor receptor	T53524	Thrombocytopenia [ICD-11: 3B64]	50.63%	71.67%
Phosphodiesterase 3A	T88975	Bronchial asthma [ICD-11: CA23]	50.27%	92.94%
Phosphodiesterase 3A	T88975	Cardiac failure [ICD-11: BD10-BD13]	50.27%	92.94%
Phosphodiesterase 3A	T88975	Cardiovascular disease [ICD-11: BA00-BE2Z]	50.27%	92.94%
Phosphodiesterase 3A	T88975	Congestive heart failure [ICD-11: BD10]	50.27%	92.94%
Phosphodiesterase 3A	T88975	Intermittent claudication [ICD-11: BD40.00]	50.27%	92.94%
Phosphodiesterase 3A	T88975	Thrombocythemia [ICD-11: 3B63]	50.27%	92.94%
