# Supplementary material for: 3,4-Dihydropyrimidin-2(1H)-One Hybrids as Phenotypic Anti-Breast Cancer Agents: Synthesis, Colony-Formation Profiling and Computational Target Deconvolution
Source: Int J Mol Sci. 2026 Oct 27;27(21):9618. doi: 10.3390/ijms27219618 (PMC13763226; doi:10.3390/ijms27219618)
Supplement: Supplementary file 1 [file ijms-27-09618-s001.zip › Super-PRED_exported_data.zip!/Targets_4m.pdf]

Exported data

Target Name	ChEMBL-ID	Indication	Probability	Model accuracy
Cathepsin D	T67102	Hypertension [ICD-11: BA00-BA04]	98.04%	98.95%
Cathepsin D	T67102	Multiple sclerosis [ICD-11: 8A40]	98.04%	98.95%
Dihydrofolate reductase	T17345	Acute bacterial skin infection [ICD-11: 1C41]	91.61%	89.92%
Dihydrofolate reductase	T17345	Bacterial infection [ICD-11: 1A00-1C4Z]	91.61%	89.92%
Dihydrofolate reductase	T17345	Bladder cancer [ICD-11: 2C94]	91.61%	89.92%
Dihydrofolate reductase	T17345	Inflammatory bowel disease [ICD-11: DD72]	91.61%	89.92%
Dihydrofolate reductase	T17345	Pneumonia [ICD-11: CA40]	91.61%	89.92%
Dihydrofolate reductase	T17345	Pulmonary and extrapulmonary tuberculosis [ICD-11: 1B10.Z]	91.61%	89.92%
Dihydrofolate reductase	T17345	Rheumatoid arthritis [ICD-11: FA20]	91.61%	89.92%
Dihydrofolate reductase	T17345	Solid tumour/cancer [ICD-11: 2A00-2F9Z]	91.61%	89.92%
Dihydrofolate reductase	T17345	Urinary tract infection [ICD-11: GC08]	91.61%	89.92%
G-protein coupled bile acid receptor 1	T86273	Metabolic disorder [ICD-11: 5C50-5D2Z]	91.34%	93.65%
G-protein coupled bile acid receptor 1	T86273	Type-2 diabetes [ICD-11: 5A11]	91.34%	93.65%
C-X-C chemokine receptor type 4	T96079	Acute lymphoblastic leukaemia [ICD-11: 2A85]	89.95%	93.1%
C-X-C chemokine receptor type 4	T96079	Acute myeloid leukaemia [ICD-11: 2A60]	89.95%	93.1%
C-X-C chemokine receptor type 4	T96079	Autoimmune diabetes [ICD-11: 5A10]	89.95%	93.1%
C-X-C chemokine receptor type 4	T96079	B-cell chronic lymphocytic leukaemia [ICD-11: 2A82.00]	89.95%	93.1%
C-X-C chemokine receptor type 4	T96079	Bone marrow transplantation [ICD-11: QB63.6]	89.95%	93.1%
C-X-C chemokine receptor type 4	T96079	Breast cancer [ICD-11: 2C60-2C65]	89.95%	93.1%
C-X-C chemokine receptor type 4	T96079	Constitutional neutropenia [ICD-11: 4B00.0]	89.95%	93.1%
C-X-C chemokine receptor type 4	T96079	Haematological malignancy [ICD-11: 2B33.Y]	89.95%	93.1%
C-X-C chemokine receptor type 4	T96079	Human immunodeficiency virus infection [ICD-11: 1C62]	89.95%	93.1%
C-X-C chemokine receptor type 4	T96079	Macular degeneration [ICD-11: 9B78.3]	89.95%	93.1%
C-X-C chemokine receptor type 4	T96079	Melanoma [ICD-11: 2C30]	89.95%	93.1%
C-X-C chemokine receptor type 4	T96079	Merkel cell carcinoma [ICD-11: 2C34]	89.95%	93.1%
C-X-C chemokine receptor type 4	T96079	Multiple myeloma [ICD-11: 2A83]	89.95%	93.1%
C-X-C chemokine receptor type 4	T96079	Myelodysplastic syndrome [ICD-11: 2A37]	89.95%	93.1%
C-X-C chemokine receptor type 4	T96079	Non-hodgkin lymphoma [ICD-11: 2B33.5]	89.95%	93.1%
C-X-C chemokine receptor type 4	T96079	Pancreatic cancer [ICD-11: 2C10]	89.95%	93.1%
C-X-C chemokine receptor type 4	T96079	Peripheral vascular disease [ICD-11: BD4Z]	89.95%	93.1%
C-X-C chemokine receptor type 4	T96079	Psoriasis vulgaris [ICD-11: EA90]	89.95%	93.1%
C-X-C chemokine receptor type 4	T96079	Recurring respiratory infection	89.95%	93.1%

Target Name	ChEMBL-ID	Indication	Probability	Model accuracy
		[ICD-11: CA07-CA45]		
C-X-C chemokine receptor type 4	T96079	Renal cell carcinoma [ICD-11: 2C90]	89.95%	93.1%
C-X-C chemokine receptor type 4	T96079	Sarcoma [ICD-11: 2A60-2C35]	89.95%	93.1%
C-X-C chemokine receptor type 4	T96079	Solid tumour/cancer [ICD-11: 2A00-2F9Z]	89.95%	93.1%
Histone deacetylase 4	T63816	Advanced stage follicular lymphoma [ICD-11: 2A80]	89.95%	92.97%
Histone deacetylase 4	T63816	Huntington disease [ICD-11: 8A01.10]	89.95%	92.97%
Histone deacetylase 4	T63816	Solid tumour/cancer [ICD-11: 2A00-2F9Z]	89.95%	92.97%
Histone deacetylase 2	T51191	Dermatitis [ICD-11: EA80-EA89]	89.33%	94.75%
Histone deacetylase 2	T51191	Solid tumour/cancer [ICD-11: 2A00-2F9Z]	89.33%	94.75%
Casein kinase II alpha/beta	T51565	Cholangiocarcinoma [ICD-11: 2C12.10]	86.99%	99.23%
Casein kinase II alpha/beta	T51565	Solid tumour/cancer [ICD-11: 2A00-2F9Z]	86.99%	99.23%
GABA-A receptor; alpha-1/beta-2/ gamma-2	T08910	Alcohol dependence [ICD-11: 6C40.2]	86.78%	92.67%
GABA-A receptor; alpha-1/beta-2/ gamma-2	T08910	Anaesthesia [ICD-11: 9A78.6]	86.78%	92.67%
GABA-A receptor; alpha-1/beta-2/ gamma-2	T08910	Anxiety disorder [ICD-11: 6B00-6B0Z]	86.78%	92.67%
GABA-A receptor; alpha-1/beta-2/ gamma-2	T08910	Anxiety disorder [ICD-11: 6B00-6B0Z]	86.78%	92.67%
GABA-A receptor; alpha-1/beta-2/ gamma-2	T08910	Anxiety disorder [ICD-11: 6B00-6B0Z]	86.78%	92.67%
GABA-A receptor; alpha-1/beta-2/ gamma-2	T08910	Chronic pain [ICD-11: MG30]	86.78%	92.67%
GABA-A receptor; alpha-1/beta-2/ gamma-2	T08910	Cystitis [ICD-11: GC00]	86.78%	92.67%
GABA-A receptor; alpha-1/beta-2/ gamma-2	T08910	Depression [ICD-11: 6A70-6A7Z]	86.78%	92.67%
GABA-A receptor; alpha-1/beta-2/ gamma-2	T08910	Depression [ICD-11: 6A70-6A7Z]	86.78%	92.67%
GABA-A receptor; alpha-1/beta-2/ gamma-2	T08910	Depression [ICD-11: 6A70-6A7Z]	86.78%	92.67%
GABA-A receptor; alpha-1/beta-2/ gamma-2	T08910	Epilepsy [ICD-11: 8A60-8A68]	86.78%	92.67%
GABA-A receptor; alpha-1/beta-2/ gamma-2	T08910	Epileptic seizures [ICD-11: 8A61-8A6Z]	86.78%	92.67%
GABA-A receptor; alpha-1/beta-2/ gamma-2	T08910	Epileptic seizures [ICD-11: 8A61-8A6Z]	86.78%	92.67%
GABA-A receptor; alpha-1/beta-2/ gamma-2	T08910	Epileptic seizures [ICD-11: 8A61-8A6Z]	86.78%	92.67%
GABA-A receptor; alpha-1/beta-2/ gamma-2	T08910	Essential tremor or related tremors [ICD-11: 8A04.1]	86.78%	92.67%
GABA-A receptor; alpha-1/beta-2/ gamma-2	T08910	Essential tremor or related tremors [ICD-11: 8A04.1]	86.78%	92.67%
GABA-A receptor; alpha-1/beta-2/ gamma-2	T08910	Essential tremor or related tremors [ICD-11: 8A04.1]	86.78%	92.67%
GABA-A receptor; alpha-1/beta-2/	T08910	Fibromyalgia [ICD-11: MG30.01]	86.78%	92.67%

Target Name	ChEMBL-ID	Indication	Probability	Model accuracy
gamma-2				
GABA-A receptor; alpha-1/beta-2/ gamma-2	T08910	Headache [ICD-11: 8A80-8A84]	86.78%	92.67%
GABA-A receptor; alpha-1/beta-2/ gamma-2	T08910	Inflammation [ICD-11: 1A00-CA43.1]	86.78%	92.67%
GABA-A receptor; alpha-1/beta-2/ gamma-2	T08910	Inflammation [ICD-11: 1A00-CA43.1]	86.78%	92.67%
GABA-A receptor; alpha-1/beta-2/ gamma-2	T08910	Inflammation [ICD-11: 1A00-CA43.1]	86.78%	92.67%
GABA-A receptor; alpha-1/beta-2/ gamma-2	T08910	Inflammatory bowel disease [ICD-11: DD72]	86.78%	92.67%
GABA-A receptor; alpha-1/beta-2/ gamma-2	T08910	Inflammatory bowel disease [ICD-11: DD72]	86.78%	92.67%
GABA-A receptor; alpha-1/beta-2/ gamma-2	T08910	Inflammatory bowel disease [ICD-11: DD72]	86.78%	92.67%
GABA-A receptor; alpha-1/beta-2/ gamma-2	T08910	Insomnia [ICD-11: 7A00-7A0Z]	86.78%	92.67%
GABA-A receptor; alpha-1/beta-2/ gamma-2	T08910	Insomnia [ICD-11: 7A00-7A0Z]	86.78%	92.67%
GABA-A receptor; alpha-1/beta-2/ gamma-2	T08910	Intractable insomnia [ICD-11: 7A00]	86.78%	92.67%
GABA-A receptor; alpha-1/beta-2/ gamma-2	T08910	Ischemic reperfusion injury [ICD-11: DB98.B]	86.78%	92.67%
GABA-A receptor; alpha-1/beta-2/ gamma-2	T08910	Malaria [ICD-11: 1F40-1F45]	86.78%	92.67%
GABA-A receptor; alpha-1/beta-2/ gamma-2	T08910	Muscle spasm [ICD-11: MB47.3]	86.78%	92.67%
GABA-A receptor; alpha-1/beta-2/ gamma-2	T08910	Muscle spasm [ICD-11: MB47.3]	86.78%	92.67%
GABA-A receptor; alpha-1/beta-2/ gamma-2	T08910	Muscle spasm [ICD-11: MB47.3]	86.78%	92.67%
GABA-A receptor; alpha-1/beta-2/ gamma-2	T08910	Postpartum depression [ICD-11: 6E20.0]	86.78%	92.67%
GABA-A receptor; alpha-1/beta-2/ gamma-2	T08910	Postpartum depression [ICD-11: 6E20.0]	86.78%	92.67%
GABA-A receptor; alpha-1/beta-2/ gamma-2	T08910	Postpartum depression [ICD-11: 6E20.0]	86.78%	92.67%
GABA-A receptor; alpha-1/beta-2/ gamma-2	T08910	Premenstrual syndrome [ICD-11: GA34.40]	86.78%	92.67%
GABA-A receptor; alpha-1/beta-2/ gamma-2	T08910	Premenstrual syndrome [ICD-11: GA34.40]	86.78%	92.67%
GABA-A receptor; alpha-1/beta-2/ gamma-2	T08910	Premenstrual syndrome [ICD-11: GA34.40]	86.78%	92.67%
GABA-A receptor; alpha-1/beta-2/ gamma-2	T08910	Respiratory distress syndrome [ICD-11: CB00]	86.78%	92.67%
GABA-A receptor; alpha-1/beta-2/ gamma-2	T08910	Status epilepticus seizure [ICD-11: 8A66.1Y]	86.78%	92.67%
GABA-A receptor; alpha-1/beta-2/ gamma-2	T08910	Status epilepticus seizure [ICD-11: 8A66.1Y]	86.78%	92.67%
GABA-A receptor; alpha-1/beta-2/ gamma-2	T08910	Status epilepticus seizure [ICD-11: 8A66.1Y]	86.78%	92.67%
Beta-glucuronidase	T96413	Mucopolysaccharidosis [ICD-11: 5C56.3]	86.46%	77.68%

Target Name	ChEMBL-ID	Indication	Probability	Model accuracy
Beta-glucuronidase	T96413	Periodontal disease [ICD-11: DA0C]	86.46%	77.68%
Sodium/hydrogen exchanger 1	T82028	Angina pectoris [ICD-11: BA40]	85.43%	90.24%
Sodium/hydrogen exchanger 1	T82028	Cardiac arrhythmias [ICD-11: BC9Z]	85.43%	90.24%
Sodium/hydrogen exchanger 1	T82028	Heart arrhythmia [ICD-11: BC65]	85.43%	90.24%
Sodium/hydrogen exchanger 1	T82028	Myocardial infarction [ICD-11: BA41-BA43]	85.43%	90.24%
Anandamide amidohydrolase	T11754	Anaesthesia [ICD-11: 9A78.6]	83.72%	97.53%
Anandamide amidohydrolase	T11754	Anxiety disorder [ICD-11: 6B00-6B0Z]	83.72%	97.53%
Anandamide amidohydrolase	T11754	Inflammation [ICD-11: 1A00-CA43.1]	83.72%	97.53%
Anandamide amidohydrolase	T11754	Liver disease [ICD-11: DB90-BD99]	83.72%	97.53%
Anandamide amidohydrolase	T11754	Major depressive disorder [ICD-11: 6A70.3]	83.72%	97.53%
Anandamide amidohydrolase	T11754	Neuropathic pain [ICD-11: 8E43.0]	83.72%	97.53%
Anandamide amidohydrolase	T11754	Pain [ICD-11: MG30-MG3Z]	83.72%	97.53%
Anandamide amidohydrolase	T11754	Social anxiety disorder [ICD-11: 6B04]	83.72%	97.53%
Serotonin 2c (5-HT2c) receptor	T83813	Alcohol dependence [ICD-11: 6C40.2]	80.05%	89.62%
Serotonin 2c (5-HT2c) receptor	T83813	Alzheimer disease [ICD-11: 8A20]	80.05%	89.62%
Serotonin 2c (5-HT2c) receptor	T83813	Anxiety disorder [ICD-11: 6B00-6B0Z]	80.05%	89.62%
Serotonin 2c (5-HT2c) receptor	T83813	Attention deficit hyperactivity disorder [ICD-11: 6A05.Z]	80.05%	89.62%
Serotonin 2c (5-HT2c) receptor	T83813	Depression [ICD-11: 6A70-6A7Z]	80.05%	89.62%
Serotonin 2c (5-HT2c) receptor	T83813	Diabetic complication [ICD-11: 5A2Y]	80.05%	89.62%
Serotonin 2c (5-HT2c) receptor	T83813	Drug abuse [ICD-11: 6C4G.1Z]	80.05%	89.62%
Serotonin 2c (5-HT2c) receptor	T83813	Dyskinesia [ICD-11: MB47.4]	80.05%	89.62%
Serotonin 2c (5-HT2c) receptor	T83813	Generalized anxiety disorder [ICD-11: 6B00]	80.05%	89.62%
Serotonin 2c (5-HT2c) receptor	T83813	Hyperprolactinaemia [ICD-11: 5A60.1]	80.05%	89.62%
Serotonin 2c (5-HT2c) receptor	T83813	Major depressive disorder [ICD-11: 6A70.3]	80.05%	89.62%
Serotonin 2c (5-HT2c) receptor	T83813	Metabolic disorder [ICD-11: 5C50-5D2Z]	80.05%	89.62%
Serotonin 2c (5-HT2c) receptor	T83813	Migraine [ICD-11: 8A80]	80.05%	89.62%
Serotonin 2c (5-HT2c) receptor	T83813	Mood disorder [ICD-11: 6A60-6E23]	80.05%	89.62%
Serotonin 2c (5-HT2c) receptor	T83813	Neurological disorder [ICD-11: 6B60]	80.05%	89.62%
Serotonin 2c (5-HT2c) receptor	T83813	Non-hodgkin lymphoma [ICD-11: 2B33.5]	80.05%	89.62%
Serotonin 2c (5-HT2c) receptor	T83813	Obesity [ICD-11: 5B81]	80.05%	89.62%
Serotonin 2c (5-HT2c) receptor	T83813	Pain [ICD-11: MG30-MG3Z]	80.05%	89.62%
Serotonin 2c (5-HT2c) receptor	T83813	Parkinson disease [ICD-11: 8A00.0]	80.05%	89.62%
Serotonin 2c (5-HT2c) receptor	T83813	Primary insomnia [ICD-11: 7A00]	80.05%	89.62%
Serotonin 2c (5-HT2c) receptor	T83813	Psychotic disorder [ICD-11: 6A20-6A25]	80.05%	89.62%
Serotonin 2c (5-HT2c) receptor	T83813	Schizophrenia [ICD-11: 6A20]	80.05%	89.62%
Serotonin 2c (5-HT2c) receptor	T83813	Sleep-wake disorder [ICD-11: 7A00-7B2Z]	80.05%	89.62%
ADAM10	T31902	Breast cancer [ICD-11: 2C60-2C65]	79.71%	97.5%
ADAM10	T31902	Solid tumour/cancer [ICD-11: 2A00-2F9Z]	79.71%	97.5%
DNA topoisomerase II alpha	T17048	Solid tumour/cancer [ICD-11:	79.52%	89%

Target Name	ChEMBL-ID	Indication	Probability	Model accuracy
		2A00-2F9Z]		
Histone deacetylase 10	T94324	Solid tumour/cancer [ICD-11: 2A00-2F9Z]	78.26%	90.08%
Formyl peptide receptor 1	T87831	Inflammation [ICD-11: 1A00-CA43.1]	77.85%	93.56%
Formyl peptide receptor 1	T87831	Peptic ulcer [ICD-11: DA61]	77.85%	93.56%
Transient receptor potential cation channel subfamily A member 1	T84040	Diabetic neuropathy [ICD-11: 8C0Z]	77.64%	92.17%
Transient receptor potential cation channel subfamily A member 1	T84040	Pain [ICD-11: MG30-MG3Z]	77.64%	92.17%
Transient receptor potential cation channel subfamily A member 1	T84040	Throat irritation [ICD-11: CA0Y]	77.64%	92.17%
Muscarinic acetylcholine receptor M4	T20709	Asthma [ICD-11: CA23]	76.84%	94.08%
Muscarinic acetylcholine receptor M4	T20709	Attention deficit hyperactivity disorder [ICD-11: 6A05.Z]	76.84%	94.08%
Muscarinic acetylcholine receptor M4	T20709	bronchial hyperreactivity [ICD-11: CB40]	76.84%	94.08%
Muscarinic acetylcholine receptor M4	T20709	Glaucoma/ocular hypertension [ICD-11: 9C61]	76.84%	94.08%
Muscarinic acetylcholine receptor M4	T20709	Mydriasis [ICD-11: LA11.62]	76.84%	94.08%
Muscarinic acetylcholine receptor M4	T20709	Nausea [ICD-11: MD90]	76.84%	94.08%
Cyclin-dependent kinase 5/CDK5 activator 1	T20973	Obesity [ICD-11: 5B81]	75.89%	93.03%
DNA-(apurinic or apyrimidinic site) lyase	T13348	Glioma [ICD-11: 2A00.0]	75.66%	91.11%
DNA-(apurinic or apyrimidinic site) lyase	T13348	Melanoma [ICD-11: 2C30]	75.66%	91.11%
DNA-(apurinic or apyrimidinic site) lyase	T13348	Ocular cancer [ICD-11: 2D00-2D07]	75.66%	91.11%
DNA-(apurinic or apyrimidinic site) lyase	T13348	Solid tumour/cancer [ICD-11: 2A00-2F9Z]	75.66%	91.11%
Glycine receptor subunit alpha-1	T50269	Muscle spasm [ICD-11: MB47.3]	73.79%	90.71%
Sodium channel protein type III alpha subunit	T76937	Angina pectoris [ICD-11: BA40]	70.61%	96.9%
Sodium channel protein type III alpha subunit	T76937	Neurological disorder [ICD-11: 6B60]	70.61%	96.9%
Sodium channel protein type III alpha subunit	T76937	Pain [ICD-11: MG30-MG3Z]	70.61%	96.9%
Protein-tyrosine phosphatase 2C	T13057	Solid tumour/cancer [ICD-11: 2A00-2F9Z]	70.44%	94.42%
Histone deacetylase 8	T28887	Solid tumour/cancer [ICD-11: 2A00-2F9Z]	68.82%	93.99%
PI3-kinase p110-alpha/p85-alpha	T80276	Breast cancer [ICD-11: 2C60-2C65]	68.8%	94.33%
PI3-kinase p110-alpha/p85-alpha	T80276	Follicular lymphoma [ICD-11: 2A80]	68.8%	94.33%
PI3-kinase p110-alpha/p85-alpha	T80276	Non-hodgkin lymphoma [ICD-11: 2B33.5]	68.8%	94.33%
PI3-kinase p110-alpha/p85-alpha	T80276	Prostate cancer [ICD-11: 2C82.0]	68.8%	94.33%
PI3-kinase p110-alpha/p85-alpha	T80276	Solid tumour/cancer [ICD-11: 2A00-2F9Z]	68.8%	94.33%
DNA topoisomerase I	T09826	Acquired immune deficiency	68.64%	97%

Target Name	ChEMBL-ID	Indication	Probability	Model accuracy
		syndrome [ICD-11: 1C62.3]		
DNA topoisomerase I	T09826	Acute lymphoblastic leukaemia [ICD-11: 2A85]	68.64%	97%
DNA topoisomerase I	T09826	Bacterial infection [ICD-11: 1A00-1C4Z]	68.64%	97%
DNA topoisomerase I	T09826	Bladder cancer [ICD-11: 2C94]	68.64%	97%
DNA topoisomerase I	T09826	Breast cancer [ICD-11: 2C60-2C65]	68.64%	97%
DNA topoisomerase I	T09826	Colorectal cancer [ICD-11: 2B91.Z]	68.64%	97%
DNA topoisomerase I	T09826	Esophageal cancer [ICD-11: 2B70]	68.64%	97%
DNA topoisomerase I	T09826	Gastric adenocarcinoma [ICD-11: 2B72]	68.64%	97%
DNA topoisomerase I	T09826	Glioblastoma multiforme [ICD-11: 2A00.0]	68.64%	97%
DNA topoisomerase I	T09826	Human immunodeficiency virus infection [ICD-11: 1C62]	68.64%	97%
DNA topoisomerase I	T09826	Lung cancer [ICD-11: 2C25.0]	68.64%	97%
DNA topoisomerase I	T09826	Lymphoma [ICD-11: 2A80-2A86]	68.64%	97%
DNA topoisomerase I	T09826	Metastatic colorectal cancer [ICD-11: 2B91]	68.64%	97%
DNA topoisomerase I	T09826	Myelodysplastic syndrome [ICD-11: 2A37]	68.64%	97%
DNA topoisomerase I	T09826	Ovarian cancer [ICD-11: 2C73]	68.64%	97%
DNA topoisomerase I	T09826	Renal cell carcinoma [ICD-11: 2C90]	68.64%	97%
DNA topoisomerase I	T09826	Small-cell lung cancer [ICD-11: 2C25.Y]	68.64%	97%
DNA topoisomerase I	T09826	Solid tumour/cancer [ICD-11: 2A00-2F9Z]	68.64%	97%
C5a anaphylatoxin chemotactic receptor	T15439	Atopic dermatitis [ICD-11: EA80]	66.13%	92.62%
C5a anaphylatoxin chemotactic receptor	T15439	Eosinophilic granulomatosis with polyangiitis [ICD-11: 4A44.A2]	66.13%	92.62%
C5a anaphylatoxin chemotactic receptor	T15439	Hereditary haemolytic anemia [ICD-11: 3A10.3]	66.13%	92.62%
C5a anaphylatoxin chemotactic receptor	T15439	Inflammation [ICD-11: 1A00-CA43.1]	66.13%	92.62%
C5a anaphylatoxin chemotactic receptor	T15439	Rheumatoid arthritis [ICD-11: FA20]	66.13%	92.62%
C5a anaphylatoxin chemotactic receptor	T15439	Vasculitis [ICD-11: 4A44]	66.13%	92.62%
Bromodomain testis-specific protein	T90985	Testicular cancer [ICD-11: 2C80]	64.95%	89.76%
Acetyl-CoA carboxylase 2	T08922	Bacterial infection [ICD-11: 1A00-1C4Z]	64.77%	98%
Acetyl-CoA carboxylase 2	T08922	Obesity [ICD-11: 5B81]	64.77%	98%
Acetyl-CoA carboxylase 2	T08922	Parkinson disease [ICD-11: 8A00.0]	64.77%	98%
Acetyl-CoA carboxylase 2	T08922	Sebaceous gland disease [ICD-11: EM0Z]	64.77%	98%
Acetyl-CoA carboxylase 2	T08922	Type-2 diabetes [ICD-11: 5A11]	64.77%	98%
TGF-beta receptor type II	T49989	Solid tumour/cancer [ICD-11: 2A00-2F9Z]	64.7%	88.18%
TGF-beta receptor type II	T49989	Wound healing [ICD-11: EL8Y]	64.7%	88.18%

Target Name	ChEMBL-ID	Indication	Probability	Model accuracy
Integrin alpha-5/beta-1	T29879	Allergic rhinitis [ICD-11: CA08.0]	63.35%	96.39%
Integrin alpha-5/beta-1	T29879	Allergy [ICD-11: 4A80-4A85]	63.35%	96.39%
Integrin alpha-5/beta-1	T29879	Asthma [ICD-11: CA23]	63.35%	96.39%
Integrin alpha-5/beta-1	T29879	Macular degeneration [ICD-11: 9B78.3]	63.35%	96.39%
Integrin alpha-5/beta-1	T29879	Macular degeneration [ICD-11: 9B78.3]	63.35%	96.39%
Integrin alpha-5/beta-1	T29879	Multiple sclerosis [ICD-11: 8A40]	63.35%	96.39%
Integrin alpha-5/beta-1	T29879	Non-small-cell lung cancer [ICD-11: 2C25.Y]	63.35%	96.39%
Signal transducer and activator of transcription 3	T29130	Brain cancer [ICD-11: 2A00]	62.61%	82.69%
Signal transducer and activator of transcription 3	T29130	Chronic lymphocytic leukaemia [ICD-11: 2A82.0]	62.61%	82.69%
Signal transducer and activator of transcription 3	T29130	Hepatocellular carcinoma [ICD-11: 2C12.02]	62.61%	82.69%
Signal transducer and activator of transcription 3	T29130	Immune System disease [ICD-11: 4A01-4B41]	62.61%	82.69%
Signal transducer and activator of transcription 3	T29130	Inflammation [ICD-11: 1A00-CA43.1]	62.61%	82.69%
Signal transducer and activator of transcription 3	T29130	Multiple myeloma [ICD-11: 2A83]	62.61%	82.69%
Signal transducer and activator of transcription 3	T29130	Psoriasis vulgaris [ICD-11: EA90]	62.61%	82.69%
Signal transducer and activator of transcription 3	T29130	Recurrent glioblastoma [ICD-11: 2A00.00]	62.61%	82.69%
Signal transducer and activator of transcription 3	T29130	Solid tumour/cancer [ICD-11: 2A00-2F9Z]	62.61%	82.69%
Signal transducer and activator of transcription 3	T29130	Ulcerative colitis [ICD-11: DD71]	62.61%	82.69%
Toll-like receptor 8	T48703	Allergic rhinitis [ICD-11: CA08.0]	62.49%	96.25%
Toll-like receptor 8	T48703	Allergy [ICD-11: 4A80-4A85]	62.49%	96.25%
Toll-like receptor 8	T48703	Autoimmune diabetes [ICD-11: 5A10]	62.49%	96.25%
Toll-like receptor 8	T48703	Diffuse large B-cell lymphoma [ICD-11: 2A81]	62.49%	96.25%
Toll-like receptor 8	T48703	Herpes simplex virus infection [ICD-11: 1F00]	62.49%	96.25%
Toll-like receptor 8	T48703	Melanoma [ICD-11: 2C30]	62.49%	96.25%
Toll-like receptor 8	T48703	Solid tumour/cancer [ICD-11: 2A00-2F9Z]	62.49%	96.25%
Toll-like receptor 8	T48703	Systemic lupus erythematosus [ICD-11: 4A40.0]	62.49%	96.25%
Cyclin-dependent kinase 1/cyclin B	T49898	Acute lymphoblastic leukaemia [ICD-11: 2A85]	61.71%	96%
Cyclin-dependent kinase 1/cyclin B	T49898	Allergic inflammation [ICD-11: 4A80-4A85]	61.71%	96%
Cyclin-dependent kinase 1/cyclin B	T49898	Breast cancer [ICD-11: 2C60-2C65]	61.71%	96%
Cyclin-dependent kinase 1/cyclin B	T49898	Graft rejection [ICD-11: NE84]	61.71%	96%
Cyclin-dependent kinase 1/cyclin B	T49898	Haematological malignancy [ICD-11: 2B33.Y]	61.71%	96%
Cyclin-dependent kinase 1/cyclin B	T49898	Mantle cell lymphoma [ICD-11: 2A85.5]	61.71%	96%
Cyclin-dependent kinase 1/cyclin B	T49898	Nasopharyngeal carcinoma [ICD-11:	61.71%	96%

Target Name	ChEMBL-ID	Indication	Probability	Model accuracy
		2B6B]		
Cyclin-dependent kinase 1/cyclin B	T49898	Non-small-cell lung cancer [ICD-11: 2C25.Y]	61.71%	96%
Cyclin-dependent kinase 1/cyclin B	T49898	Obesity [ICD-11: 5B81]	61.71%	96%
Cyclin-dependent kinase 1/cyclin B	T49898	Pain [ICD-11: MG30-MG3Z]	61.71%	96%
Cyclin-dependent kinase 1/cyclin B	T49898	Solid tumour/cancer [ICD-11: 2A00-2F9Z]	61.71%	96%
Relaxin receptor 1	T73696	Acute heart failure [ICD-11: BD10-BD13]	61.48%	82.5%
Acetylcholinesterase	T30082	Alcohol dependence [ICD-11: 6C40.2]	61.41%	94.45%
Acetylcholinesterase	T30082	Alzheimer disease [ICD-11: 8A20]	61.41%	94.45%
Acetylcholinesterase	T30082	Chronic glaucoma [ICD-11: 9C61.0Z]	61.41%	94.45%
Acetylcholinesterase	T30082	Cognitive impairment [ICD-11: 6D71]	61.41%	94.45%
Acetylcholinesterase	T30082	Dementia [ICD-11: 6D80-6D86]	61.41%	94.45%
Acetylcholinesterase	T30082	Functional dyspepsia [ICD-11: DD90.3]	61.41%	94.45%
Acetylcholinesterase	T30082	Helminth infection [ICD-11: 1F90.0]	61.41%	94.45%
Acetylcholinesterase	T30082	Myasthenia gravis [ICD-11: 8C6Y]	61.41%	94.45%
Acetylcholinesterase	T30082	Neurodegenerative disorder [ICD-11: 8A20-8A23]	61.41%	94.45%
Acetylcholinesterase	T30082	Open-angle glaucoma [ICD-11: 9C61]	61.41%	94.45%
Acetylcholinesterase	T30082	Pain [ICD-11: MG30-MG3Z]	61.41%	94.45%
Acetylcholinesterase	T30082	Parkinson disease [ICD-11: 8A00.0]	61.41%	94.45%
Acetylcholinesterase	T30082	Pediculus capitis infestation [ICD-11: 1G00.0]	61.41%	94.45%
Acetylcholinesterase	T30082	Poisoning due to pesticides and chemicals [ICD-11: NE6Z]	61.41%	94.45%
Acetylcholinesterase	T30082	Solid tumour/cancer [ICD-11: 2A00-2F9Z]	61.41%	94.45%
Acetylcholinesterase	T30082	Urinary dysfunction [ICD-11: GC2Z]	61.41%	94.45%
Acetylcholinesterase	T30082	Xerostomia [ICD-11: DA02.1]	61.41%	94.45%
11-beta-hydroxysteroid dehydrogenase 2	T43721	Type-2 diabetes [ICD-11: 5A11]	61.15%	94.78%
Aurora kinase B/Inner centromere protein	T46781	Acute myeloid leukaemia [ICD-11: 2A60]	61.12%	97.5%
Aurora kinase B/Inner centromere protein	T46781	Diabetic complication [ICD-11: 5A2Y]	61.12%	97.5%
Aurora kinase B/Inner centromere protein	T46781	Haematological malignancy [ICD-11: 2B33.Y]	61.12%	97.5%
Aurora kinase B/Inner centromere protein	T46781	Prostate cancer [ICD-11: 2C82.0]	61.12%	97.5%
Aurora kinase B/Inner centromere protein	T46781	Solid tumour/cancer [ICD-11: 2A00-2F9Z]	61.12%	97.5%
Pregnane X receptor	T82702	Arteriosclerosis [ICD-11: BD40]	60.53%	94.73%
Tissue factor pathway inhibitor	T78890	Factor IX deficiency [ICD-11: 3B11]	60.07%	97.33%
Tissue factor pathway inhibitor	T78890	Sepsis [ICD-11: 1G40-1G41]	60.07%	97.33%
PI3-kinase p110-delta subunit	T67849	Arthritis [ICD-11: FA20]	59.59%	96.47%
PI3-kinase p110-delta subunit	T67849	Breast cancer [ICD-11: 2C60-2C65]	59.59%	96.47%
PI3-kinase p110-delta subunit	T67849	Chronic lymphocytic leukaemia [ICD-11: 2A82.0]	59.59%	96.47%
PI3-kinase p110-delta subunit	T67849	Coronavirus Disease 2019 (COVID-19)	59.59%	96.47%

Target Name	ChEMBL-ID	Indication	Probability	Model accuracy
		[ICD-11: 1D6Y]		
PI3-kinase p110-delta subunit	T67849	Cutaneous T-cell lymphoma [ICD-11: 2B01]	59.59%	96.47%
PI3-kinase p110-delta subunit	T67849	Diffuse large B-cell lymphoma [ICD-11: 2A81]	59.59%	96.47%
PI3-kinase p110-delta subunit	T67849	Follicular lymphoma [ICD-11: 2A80]	59.59%	96.47%
PI3-kinase p110-delta subunit	T67849	Follicular lymphoma [ICD-11: 2A80]	59.59%	96.47%
PI3-kinase p110-delta subunit	T67849	Hematologic tumour [ICD-11: 2B33.Y]	59.59%	96.47%
PI3-kinase p110-delta subunit	T67849	Lymphoma [ICD-11: 2A80-2A86]	59.59%	96.47%
PI3-kinase p110-delta subunit	T67849	Mantle cell lymphoma [ICD-11: 2A85.5]	59.59%	96.47%
PI3-kinase p110-delta subunit	T67849	Marginal zone lymphoma [ICD-11: 2A85.0]	59.59%	96.47%
PI3-kinase p110-delta subunit	T67849	Myeloid leukaemia [ICD-11: 2B33.1]	59.59%	96.47%
PI3-kinase p110-delta subunit	T67849	Non-hodgkin lymphoma [ICD-11: 2B33.5]	59.59%	96.47%
PI3-kinase p110-delta subunit	T67849	Solid tumour/cancer [ICD-11: 2A00-2F9Z]	59.59%	96.47%
Voltage-gated N-type calcium channel alpha-1B subunit	T38338	Anxiety disorder [ICD-11: 6B00-6B0Z]	59.34%	97.14%
Voltage-gated N-type calcium channel alpha-1B subunit	T38338	Cardiac failure [ICD-11: BD10-BD13]	59.34%	97.14%
Voltage-gated N-type calcium channel alpha-1B subunit	T38338	High blood pressure [ICD-11: BA00]	59.34%	97.14%
Voltage-gated N-type calcium channel alpha-1B subunit	T38338	Migraine [ICD-11: 8A80]	59.34%	97.14%
Voltage-gated N-type calcium channel alpha-1B subunit	T38338	Neuropathic pain [ICD-11: 8E43.0]	59.34%	97.14%
Voltage-gated N-type calcium channel alpha-1B subunit	T38338	Pain [ICD-11: MG30-MG3Z]	59.34%	97.14%
Voltage-gated N-type calcium channel alpha-1B subunit	T38338	Reperfusion injury [ICD-11: ND56.Z]	59.34%	97.14%
Voltage-gated N-type calcium channel alpha-1B subunit	T38338	Traumatic brain injury [ICD-11: NA07.Z]	59.34%	97.14%
Voltage-gated N-type calcium channel alpha-1B subunit	T38338	Vascular purpura [ICD-11: 3B60]	59.34%	97.14%
Serotonin 7 (5-HT7) receptor	T79062	Alzheimer disease [ICD-11: 8A20]	58.17%	90.71%
Serotonin 7 (5-HT7) receptor	T79062	Attention deficit hyperactivity disorder [ICD-11: 6A05.Z]	58.17%	90.71%
Serotonin 7 (5-HT7) receptor	T79062	Bipolar disorder [ICD-11: 6A60]	58.17%	90.71%
Serotonin 7 (5-HT7) receptor	T79062	Major depressive disorder [ICD-11: 6A70.3]	58.17%	90.71%
Serotonin 7 (5-HT7) receptor	T79062	Parkinson disease [ICD-11: 8A00.0]	58.17%	90.71%
Serotonin 7 (5-HT7) receptor	T79062	Psychiatric disorder [ICD-11: 6E8Z]	58.17%	90.71%
Serotonin 7 (5-HT7) receptor	T79062	Schizophrenia [ICD-11: 6A20]	58.17%	90.71%
Serotonin 7 (5-HT7) receptor	T79062	Sleep-wake disorder [ICD-11: 7A00-7B2Z]	58.17%	90.71%
G-protein coupled receptor 55	T87670	Attention deficit hyperactivity disorder [ICD-11: 6A05.Z]	56.86%	78.15%
Cyclin-dependent kinase 4/cyclin D1	T85799	Breast cancer [ICD-11: 2C60-2C65]	56.72%	98.99%

Target Name	ChEMBL-ID	Indication	Probability	Model accuracy
Cyclin-dependent kinase 4/cyclin D1	T85799	Haematological malignancy [ICD-11: 2B33.Y]	56.72%	98.99%
Cyclin-dependent kinase 4/cyclin D1	T85799	Mantle cell lymphoma [ICD-11: 2A85.5]	56.72%	98.99%
Cyclin-dependent kinase 4/cyclin D1	T85799	Myelofibrosis [ICD-11: 2A20.2]	56.72%	98.99%
Cyclin-dependent kinase 4/cyclin D1	T85799	Psoriasis vulgaris [ICD-11: EA90]	56.72%	98.99%
Cyclin-dependent kinase 4/cyclin D1	T85799	Relapsed or refractory chronic lymphocytic leukaemia [ICD-11: 2A82.0]	56.72%	98.99%
Cyclin-dependent kinase 4/cyclin D1	T85799	Retinoblastoma [ICD-11: 2D02.2]	56.72%	98.99%
Cyclin-dependent kinase 4/cyclin D1	T85799	Schizophrenia [ICD-11: 6A20]	56.72%	98.99%
Cyclin-dependent kinase 4/cyclin D1	T85799	Small-cell lung cancer [ICD-11: 2C25.Y]	56.72%	98.99%
Cyclin-dependent kinase 4/cyclin D1	T85799	Solid tumour/cancer [ICD-11: 2A00-2F9Z]	56.72%	98.99%
Cyclin-dependent kinase 4/cyclin D1	T85799	Solid tumour/cancer [ICD-11: 2A00-2F9Z]	56.72%	98.99%
Glutaminase kidney isoform, mitochondrial	T86734	Breast cancer [ICD-11: 2C60-2C65]	54.47%	100%
Glutaminase kidney isoform, mitochondrial	T86734	leukaemia [ICD-11: 2A60-2B33]	54.47%	100%
Glutaminase kidney isoform, mitochondrial	T86734	Renal cell carcinoma [ICD-11: 2C90]	54.47%	100%
Glutaminase kidney isoform, mitochondrial	T86734	Solid tumour/cancer [ICD-11: 2A00-2F9Z]	54.47%	100%
Sphingosine 1-phosphate receptor Edg-8	T50089	Multiple sclerosis [ICD-11: 8A40]	53.89%	100%
Complement factor D	T66383	Age-related macular degeneration [ICD-11: 9B75.0]	52.13%	98%
Complement factor D	T66383	Autoimmune diabetes [ICD-11: 5A10]	52.13%	98%
Complement factor D	T66383	Glomerulonephritis [ICD-11: 5D00.Y]	52.13%	98%
Cysteinyl leukotriene receptor 2	T74238	Asthma [ICD-11: CA23]	51.5%	98%
Nitric oxide synthase, inducible	T02703	Artery stenosis [ICD-11: BD52]	51.48%	94.8%
Nitric oxide synthase, inducible	T02703	Asthma [ICD-11: CA23]	51.48%	94.8%
Nitric oxide synthase, inducible	T02703	Cardiovascular disease [ICD-11: BA00-BE2Z]	51.48%	94.8%
Nitric oxide synthase, inducible	T02703	Coronary artery disease [ICD-11: BA80]	51.48%	94.8%
Nitric oxide synthase, inducible	T02703	Diabetic kidney disease [ICD-11: GB61.Z]	51.48%	94.8%
Nitric oxide synthase, inducible	T02703	Diabetic retinopathy [ICD-11: 9B71.0]	51.48%	94.8%
Nitric oxide synthase, inducible	T02703	Diarrhea [ICD-11: ME05.1]	51.48%	94.8%
Nitric oxide synthase, inducible	T02703	Endotoxic shock [ICD-11: 1G41]	51.48%	94.8%
Nitric oxide synthase, inducible	T02703	Inflammation [ICD-11: 1A00-CA43.1]	51.48%	94.8%
Nitric oxide synthase, inducible	T02703	Insulin-resistant disorder [ICD-11: 5A44]	51.48%	94.8%
Nitric oxide synthase, inducible	T02703	Osteoarthritis [ICD-11: FA00-FA05]	51.48%	94.8%

Target Name	ChEMBL-ID	Indication	Probability	Model accuracy
Nitric oxide synthase, inducible	T02703	Postoperative pain [ICD-11: MG30-MG3Z]	51.48%	94.8%
Nitric oxide synthase, inducible	T02703	Rheumatoid arthritis [ICD-11: FA20]	51.48%	94.8%
Nitric oxide synthase, inducible	T02703	Sepsis [ICD-11: 1G40-1G41]	51.48%	94.8%
Nitric oxide synthase, inducible	T02703	Solid tumour/cancer [ICD-11: 2A00-2F9Z]	51.48%	94.8%
Serotonin 2a (5-HT2a) receptor	T32060	Addictive disorder [ICD-11: 6C50-6C5Z]	51.18%	91.43%
Serotonin 2a (5-HT2a) receptor	T32060	Alcohol dependence [ICD-11: 6C40.2]	51.18%	91.43%
Serotonin 2a (5-HT2a) receptor	T32060	Alzheimer disease [ICD-11: 8A20]	51.18%	91.43%
Serotonin 2a (5-HT2a) receptor	T32060	Anxiety disorder [ICD-11: 6B00-6B0Z]	51.18%	91.43%
Serotonin 2a (5-HT2a) receptor	T32060	Attention deficit hyperactivity disorder [ICD-11: 6A05.Z]	51.18%	91.43%
Serotonin 2a (5-HT2a) receptor	T32060	Benign prostatic hyperplasia [ICD-11: GA90]	51.18%	91.43%
Serotonin 2a (5-HT2a) receptor	T32060	Bipolar disorder [ICD-11: 6A60]	51.18%	91.43%
Serotonin 2a (5-HT2a) receptor	T32060	Cardiovascular disease [ICD-11: BA00-BE2Z]	51.18%	91.43%
Serotonin 2a (5-HT2a) receptor	T32060	Central nervous system disease [ICD-11: 8A04-8D87]	51.18%	91.43%
Serotonin 2a (5-HT2a) receptor	T32060	Cerebrovascular ischaemia [ICD-11: 8B1Z]	51.18%	91.43%
Serotonin 2a (5-HT2a) receptor	T32060	Depression [ICD-11: 6A70-6A7Z]	51.18%	91.43%
Serotonin 2a (5-HT2a) receptor	T32060	Diabetic complication [ICD-11: 5A2Y]	51.18%	91.43%
Serotonin 2a (5-HT2a) receptor	T32060	Diabetic nephropathy [ICD-11: GB61.Z]	51.18%	91.43%
Serotonin 2a (5-HT2a) receptor	T32060	Erythropoietic porphyrias [ICD-11: 5C58.12]	51.18%	91.43%
Serotonin 2a (5-HT2a) receptor	T32060	Female sexual arousal dysfunction [ICD-11: HA01.1]	51.18%	91.43%
Serotonin 2a (5-HT2a) receptor	T32060	Fibromyalgia [ICD-11: MG30.01]	51.18%	91.43%
Serotonin 2a (5-HT2a) receptor	T32060	Glaucoma/ocular hypertension [ICD-11: 9C61]	51.18%	91.43%
Serotonin 2a (5-HT2a) receptor	T32060	Hemorrhoids [ICD-11: DB60]	51.18%	91.43%
Serotonin 2a (5-HT2a) receptor	T32060	Hyperprolactinaemia [ICD-11: 5A60.1]	51.18%	91.43%
Serotonin 2a (5-HT2a) receptor	T32060	Hypertension [ICD-11: BA00-BA04]	51.18%	91.43%
Serotonin 2a (5-HT2a) receptor	T32060	Idiopathic pulmonary fibrosis [ICD-11: CB03.4]	51.18%	91.43%
Serotonin 2a (5-HT2a) receptor	T32060	Insomnia [ICD-11: 7A00-7A0Z]	51.18%	91.43%
Serotonin 2a (5-HT2a) receptor	T32060	Lewy body dementia [ICD-11: 6D82]	51.18%	91.43%
Serotonin 2a (5-HT2a) receptor	T32060	Major depressive disorder [ICD-11: 6A70.3]	51.18%	91.43%
Serotonin 2a (5-HT2a) receptor	T32060	Migraine [ICD-11: 8A80]	51.18%	91.43%
Serotonin 2a (5-HT2a) receptor	T32060	Mood disorder [ICD-11: 6A60-6E23]	51.18%	91.43%
Serotonin 2a (5-HT2a) receptor	T32060	Pancreatic cancer [ICD-11: 2C10]	51.18%	91.43%
Serotonin 2a (5-HT2a) receptor	T32060	Pancreatitis [ICD-11: DC31-DC34]	51.18%	91.43%
Serotonin 2a (5-HT2a) receptor	T32060	Parkinson disease [ICD-11: 8A00.0]	51.18%	91.43%
Serotonin 2a (5-HT2a) receptor	T32060	Post-traumatic stress disorder [ICD-11: 6B40]	51.18%	91.43%
Serotonin 2a (5-HT2a) receptor	T32060	Primary insomnia [ICD-11: 7A00]	51.18%	91.43%
Serotonin 2a (5-HT2a) receptor	T32060	Psychiatric disorder [ICD-11: 6E8Z]	51.18%	91.43%

Target Name	ChEMBL-ID	Indication	Probability	Model accuracy
Serotonin 2a (5-HT2a) receptor	T32060	Psychotic disorder [ICD-11: 6A20-6A25]	51.18%	91.43%
Serotonin 2a (5-HT2a) receptor	T32060	Schizoaffective disorder [ICD-11: 6A21]	51.18%	91.43%
Serotonin 2a (5-HT2a) receptor	T32060	Schizophrenia [ICD-11: 6A20]	51.18%	91.43%
Serotonin 2a (5-HT2a) receptor	T32060	Sleep-wake disorder [ICD-11: 7A00-7B2Z]	51.18%	91.43%
Serotonin 2a (5-HT2a) receptor	T32060	Social phobia [ICD-11: 6B04]	51.18%	91.43%
Serotonin 2a (5-HT2a) receptor	T32060	Type-2 diabetes [ICD-11: 5A11]	51.18%	91.43%
