# Supplementary material for: 3,4-Dihydropyrimidin-2(1H)-One Hybrids as Phenotypic Anti-Breast Cancer Agents: Synthesis, Colony-Formation Profiling and Computational Target Deconvolution
Source: Int J Mol Sci. 2026 Oct 27;27(21):9618. doi: 10.3390/ijms27219618 (PMC13763226; doi:10.3390/ijms27219618)
Supplement: Supplementary file 1 [file ijms-27-09618-s001.zip › Super-PRED_exported_data.zip!/Targets_4o.pdf]

Exported data

Target Name	ChEMBL-ID	Indication	Probability	Model accuracy
Cathepsin D	T67102	Hypertension [ICD-11: BA00-BA04]	97.54%	98.95%
Cathepsin D	T67102	Multiple sclerosis [ICD-11: 8A40]	97.54%	98.95%
DNA topoisomerase II alpha	T17048	Solid tumour/cancer [ICD-11: 2A00-2F9Z]	85.53%	89%
TGF-beta receptor type II	T49989	Solid tumour/cancer [ICD-11: 2A00-2F9Z]	84.71%	88.18%
TGF-beta receptor type II	T49989	Wound healing [ICD-11: EL8Y]	84.71%	88.18%
ADAM10	T31902	Breast cancer [ICD-11: 2C60-2C65]	84.15%	97.5%
ADAM10	T31902	Solid tumour/cancer [ICD-11: 2A00-2F9Z]	84.15%	97.5%
G-protein coupled bile acid receptor 1	T86273	Metabolic disorder [ICD-11: 5C50-5D2Z]	84.03%	93.65%
G-protein coupled bile acid receptor 1	T86273	Type-2 diabetes [ICD-11: 5A11]	84.03%	93.65%
Sodium/hydrogen exchanger 1	T82028	Angina pectoris [ICD-11: BA40]	83.69%	90.24%
Sodium/hydrogen exchanger 1	T82028	Cardiac arrhythmias [ICD-11: BC9Z]	83.69%	90.24%
Sodium/hydrogen exchanger 1	T82028	Heart arrhythmia [ICD-11: BC65]	83.69%	90.24%
Sodium/hydrogen exchanger 1	T82028	Myocardial infarction [ICD-11: BA41-BA43]	83.69%	90.24%
Dihydrofolate reductase	T17345	Acute bacterial skin infection [ICD-11: 1C41]	83.55%	89.92%
Dihydrofolate reductase	T17345	Bacterial infection [ICD-11: 1A00-1C4Z]	83.55%	89.92%
Dihydrofolate reductase	T17345	Bladder cancer [ICD-11: 2C94]	83.55%	89.92%
Dihydrofolate reductase	T17345	Inflammatory bowel disease [ICD-11: DD72]	83.55%	89.92%
Dihydrofolate reductase	T17345	Pneumonia [ICD-11: CA40]	83.55%	89.92%
Dihydrofolate reductase	T17345	Pulmonary and extrapulmonary tuberculosis [ICD-11: 1B10.Z]	83.55%	89.92%
Dihydrofolate reductase	T17345	Rheumatoid arthritis [ICD-11: FA20]	83.55%	89.92%
Dihydrofolate reductase	T17345	Solid tumour/cancer [ICD-11: 2A00-2F9Z]	83.55%	89.92%
Dihydrofolate reductase	T17345	Urinary tract infection [ICD-11: GC08]	83.55%	89.92%
Histone deacetylase 8	T28887	Solid tumour/cancer [ICD-11: 2A00-2F9Z]	83.16%	93.99%
Histone deacetylase 10	T94324	Solid tumour/cancer [ICD-11: 2A00-2F9Z]	83.16%	90.08%
Beta-glucuronidase	T96413	Mucopolysaccharidosis [ICD-11: 5C56.3]	82.78%	77.68%
Beta-glucuronidase	T96413	Periodontal disease [ICD-11: DA0C]	82.78%	77.68%
Histone deacetylase 2	T51191	Dermatitis [ICD-11: EA80-EA89]	82.28%	94.75%
Histone deacetylase 2	T51191	Solid tumour/cancer [ICD-11: 2A00-2F9Z]	82.28%	94.75%
Formyl peptide receptor 1	T87831	Inflammation [ICD-11: 1A00-CA43.1]	81.25%	93.56%
Formyl peptide receptor 1	T87831	Peptic ulcer [ICD-11: DA61]	81.25%	93.56%
Transient receptor potential cation channel subfamily A member 1	T84040	Diabetic neuropathy [ICD-11: 8C0Z]	80.39%	92.17%
Transient receptor potential cation	T84040	Pain [ICD-11: MG30-MG3Z]	80.39%	92.17%

Target Name	ChEMBL-ID	Indication	Probability	Model accuracy
channel subfamily A member 1				
Transient receptor potential cation channel subfamily A member 1	T84040	Throat irritation [ICD-11: CA0Y]	80.39%	92.17%
GABA-A receptor; alpha-1/beta-2/gamma-2	T08910	Alcohol dependence [ICD-11: 6C40.2]	79.06%	92.67%
GABA-A receptor; alpha-1/beta-2/gamma-2	T08910	Anaesthesia [ICD-11: 9A78.6]	79.06%	92.67%
GABA-A receptor; alpha-1/beta-2/gamma-2	T08910	Anxiety disorder [ICD-11: 6B00-6B0Z]	79.06%	92.67%
GABA-A receptor; alpha-1/beta-2/gamma-2	T08910	Anxiety disorder [ICD-11: 6B00-6B0Z]	79.06%	92.67%
GABA-A receptor; alpha-1/beta-2/gamma-2	T08910	Anxiety disorder [ICD-11: 6B00-6B0Z]	79.06%	92.67%
GABA-A receptor; alpha-1/beta-2/gamma-2	T08910	Chronic pain [ICD-11: MG30]	79.06%	92.67%
GABA-A receptor; alpha-1/beta-2/gamma-2	T08910	Cystitis [ICD-11: GC00]	79.06%	92.67%
GABA-A receptor; alpha-1/beta-2/gamma-2	T08910	Depression [ICD-11: 6A70-6A7Z]	79.06%	92.67%
GABA-A receptor; alpha-1/beta-2/gamma-2	T08910	Depression [ICD-11: 6A70-6A7Z]	79.06%	92.67%
GABA-A receptor; alpha-1/beta-2/gamma-2	T08910	Depression [ICD-11: 6A70-6A7Z]	79.06%	92.67%
GABA-A receptor; alpha-1/beta-2/gamma-2	T08910	Epilepsy [ICD-11: 8A60-8A68]	79.06%	92.67%
GABA-A receptor; alpha-1/beta-2/gamma-2	T08910	Epileptic seizures [ICD-11: 8A61-8A6Z]	79.06%	92.67%
GABA-A receptor; alpha-1/beta-2/gamma-2	T08910	Epileptic seizures [ICD-11: 8A61-8A6Z]	79.06%	92.67%
GABA-A receptor; alpha-1/beta-2/gamma-2	T08910	Epileptic seizures [ICD-11: 8A61-8A6Z]	79.06%	92.67%
GABA-A receptor; alpha-1/beta-2/gamma-2	T08910	Essential tremor or related tremors [ICD-11: 8A04.1]	79.06%	92.67%
GABA-A receptor; alpha-1/beta-2/gamma-2	T08910	Essential tremor or related tremors [ICD-11: 8A04.1]	79.06%	92.67%
GABA-A receptor; alpha-1/beta-2/gamma-2	T08910	Essential tremor or related tremors [ICD-11: 8A04.1]	79.06%	92.67%
GABA-A receptor; alpha-1/beta-2/gamma-2	T08910	Fibromyalgia [ICD-11: MG30.01]	79.06%	92.67%
GABA-A receptor; alpha-1/beta-2/gamma-2	T08910	Headache [ICD-11: 8A80-8A84]	79.06%	92.67%
GABA-A receptor; alpha-1/beta-2/gamma-2	T08910	Inflammation [ICD-11: 1A00-CA43.1]	79.06%	92.67%
GABA-A receptor; alpha-1/beta-2/gamma-2	T08910	Inflammation [ICD-11: 1A00-CA43.1]	79.06%	92.67%
GABA-A receptor; alpha-1/beta-2/gamma-2	T08910	Inflammation [ICD-11: 1A00-CA43.1]	79.06%	92.67%
GABA-A receptor; alpha-1/beta-2/gamma-2	T08910	Inflammatory bowel disease [ICD-11: DD72]	79.06%	92.67%
GABA-A receptor; alpha-1/beta-2/gamma-2	T08910	Inflammatory bowel disease [ICD-11: DD72]	79.06%	92.67%
GABA-A receptor; alpha-1/beta-2/gamma-2	T08910	Inflammatory bowel disease [ICD-11: DD72]	79.06%	92.67%

Target Name	ChEMBL-ID	Indication	Probability	Model accuracy
GABA-A receptor; alpha-1/beta-2/gamma-2	T08910	Insomnia [ICD-11: 7A00-7A0Z]	79.06%	92.67%
GABA-A receptor; alpha-1/beta-2/gamma-2	T08910	Insomnia [ICD-11: 7A00-7A0Z]	79.06%	92.67%
GABA-A receptor; alpha-1/beta-2/gamma-2	T08910	Intractable insomnia [ICD-11: 7A00]	79.06%	92.67%
GABA-A receptor; alpha-1/beta-2/gamma-2	T08910	Ischemic reperfusion injury [ICD-11: DB98.B]	79.06%	92.67%
GABA-A receptor; alpha-1/beta-2/gamma-2	T08910	Malaria [ICD-11: 1F40-1F45]	79.06%	92.67%
GABA-A receptor; alpha-1/beta-2/gamma-2	T08910	Muscle spasm [ICD-11: MB47.3]	79.06%	92.67%
GABA-A receptor; alpha-1/beta-2/gamma-2	T08910	Muscle spasm [ICD-11: MB47.3]	79.06%	92.67%
GABA-A receptor; alpha-1/beta-2/gamma-2	T08910	Muscle spasm [ICD-11: MB47.3]	79.06%	92.67%
GABA-A receptor; alpha-1/beta-2/gamma-2	T08910	Postpartum depression [ICD-11: 6E20.0]	79.06%	92.67%
GABA-A receptor; alpha-1/beta-2/gamma-2	T08910	Postpartum depression [ICD-11: 6E20.0]	79.06%	92.67%
GABA-A receptor; alpha-1/beta-2/gamma-2	T08910	Postpartum depression [ICD-11: 6E20.0]	79.06%	92.67%
GABA-A receptor; alpha-1/beta-2/gamma-2	T08910	Premenstrual syndrome [ICD-11: GA34.40]	79.06%	92.67%
GABA-A receptor; alpha-1/beta-2/gamma-2	T08910	Premenstrual syndrome [ICD-11: GA34.40]	79.06%	92.67%
GABA-A receptor; alpha-1/beta-2/gamma-2	T08910	Premenstrual syndrome [ICD-11: GA34.40]	79.06%	92.67%
GABA-A receptor; alpha-1/beta-2/gamma-2	T08910	Respiratory distress syndrome [ICD-11: CB00]	79.06%	92.67%
GABA-A receptor; alpha-1/beta-2/gamma-2	T08910	Status epilepticus seizure [ICD-11: 8A66.1Y]	79.06%	92.67%
GABA-A receptor; alpha-1/beta-2/gamma-2	T08910	Status epilepticus seizure [ICD-11: 8A66.1Y]	79.06%	92.67%
GABA-A receptor; alpha-1/beta-2/gamma-2	T08910	Status epilepticus seizure [ICD-11: 8A66.1Y]	79.06%	92.67%
Casein kinase II alpha/beta	T51565	Cholangiocarcinoma [ICD-11: 2C12.10]	78.84%	99.23%
Casein kinase II alpha/beta	T51565	Solid tumour/cancer [ICD-11: 2A00-2F9Z]	78.84%	99.23%
Voltage-gated N-type calcium channel alpha-1B subunit	T38338	Anxiety disorder [ICD-11: 6B00-6B0Z]	75.57%	97.14%
Voltage-gated N-type calcium channel alpha-1B subunit	T38338	Cardiac failure [ICD-11: BD10-BD13]	75.57%	97.14%
Voltage-gated N-type calcium channel alpha-1B subunit	T38338	High blood pressure [ICD-11: BA00]	75.57%	97.14%
Voltage-gated N-type calcium channel alpha-1B subunit	T38338	Migraine [ICD-11: 8A80]	75.57%	97.14%
Voltage-gated N-type calcium channel alpha-1B subunit	T38338	Neuropathic pain [ICD-11: 8E43.0]	75.57%	97.14%
Voltage-gated N-type calcium channel alpha-1B subunit	T38338	Pain [ICD-11: MG30-MG3Z]	75.57%	97.14%
Voltage-gated N-type calcium channel	T38338	Reperfusion injury [ICD-11: ND56.Z]	75.57%	97.14%

Target Name	ChEMBL-ID	Indication	Probability	Model accuracy
alpha-1B subunit				
Voltage-gated N-type calcium channel alpha-1B subunit	T38338	Traumatic brain injury [ICD-11: NA07.Z]	75.57%	97.14%
Voltage-gated N-type calcium channel alpha-1B subunit	T38338	Vascular purpura [ICD-11: 3B60]	75.57%	97.14%
DNA-(apurinic or apyrimidinic site) lyase	T13348	Glioma [ICD-11: 2A00.0]	74.62%	91.11%
DNA-(apurinic or apyrimidinic site) lyase	T13348	Melanoma [ICD-11: 2C30]	74.62%	91.11%
DNA-(apurinic or apyrimidinic site) lyase	T13348	Ocular cancer [ICD-11: 2D00-2D07]	74.62%	91.11%
DNA-(apurinic or apyrimidinic site) lyase	T13348	Solid tumour/cancer [ICD-11: 2A00-2F9Z]	74.62%	91.11%
PI3-kinase p110-alpha/p85-alpha	T80276	Breast cancer [ICD-11: 2C60-2C65]	71.84%	94.33%
PI3-kinase p110-alpha/p85-alpha	T80276	Follicular lymphoma [ICD-11: 2A80]	71.84%	94.33%
PI3-kinase p110-alpha/p85-alpha	T80276	Non-hodgkin lymphoma [ICD-11: 2B33.5]	71.84%	94.33%
PI3-kinase p110-alpha/p85-alpha	T80276	Prostate cancer [ICD-11: 2C82.0]	71.84%	94.33%
PI3-kinase p110-alpha/p85-alpha	T80276	Solid tumour/cancer [ICD-11: 2A00-2F9Z]	71.84%	94.33%
Glycine receptor subunit alpha-1	T50269	Muscle spasm [ICD-11: MB47.3]	70.33%	90.71%
Cyclin-dependent kinase 5/CDK5 activator 1	T20973	Obesity [ICD-11: 5B81]	67.99%	93.03%
C5a anaphylatoxin chemotactic receptor	T15439	Atopic dermatitis [ICD-11: EA80]	67.95%	92.62%
C5a anaphylatoxin chemotactic receptor	T15439	Eosinophilic granulomatosis with polyangiitis [ICD-11: 4A44.A2]	67.95%	92.62%
C5a anaphylatoxin chemotactic receptor	T15439	Hereditary haemolytic anemia [ICD-11: 3A10.3]	67.95%	92.62%
C5a anaphylatoxin chemotactic receptor	T15439	Inflammation [ICD-11: 1A00-CA43.1]	67.95%	92.62%
C5a anaphylatoxin chemotactic receptor	T15439	Rheumatoid arthritis [ICD-11: FA20]	67.95%	92.62%
C5a anaphylatoxin chemotactic receptor	T15439	Vasculitis [ICD-11: 4A44]	67.95%	92.62%
Aurora kinase B/Inner centromere protein	T46781	Acute myeloid leukaemia [ICD-11: 2A60]	67.74%	97.5%
Aurora kinase B/Inner centromere protein	T46781	Diabetic complication [ICD-11: 5A2Y]	67.74%	97.5%
Aurora kinase B/Inner centromere protein	T46781	Haematological malignancy [ICD-11: 2B33.Y]	67.74%	97.5%
Aurora kinase B/Inner centromere protein	T46781	Prostate cancer [ICD-11: 2C82.0]	67.74%	97.5%
Aurora kinase B/Inner centromere protein	T46781	Solid tumour/cancer [ICD-11: 2A00-2F9Z]	67.74%	97.5%
Relaxin receptor 1	T73696	Acute heart failure [ICD-11: BD10-BD13]	65.08%	82.5%
Histone deacetylase 4	T63816	Advanced stage follicular lymphoma [ICD-11: 2A80]	65.06%	92.97%
Histone deacetylase 4	T63816	Huntington disease [ICD-11: 8A01.10]	65.06%	92.97%
Histone deacetylase 4	T63816	Solid tumour/cancer [ICD-11:	65.06%	92.97%

Target Name	ChEMBL-ID	Indication	Probability	Model accuracy
		2A00-2F9Z]		
Signal transducer and activator of transcription 3	T29130	Brain cancer [ICD-11: 2A00]	63.23%	82.69%
Signal transducer and activator of transcription 3	T29130	Chronic lymphocytic leukaemia [ICD-11: 2A82.0]	63.23%	82.69%
Signal transducer and activator of transcription 3	T29130	Hepatocellular carcinoma [ICD-11: 2C12.02]	63.23%	82.69%
Signal transducer and activator of transcription 3	T29130	Immune System disease [ICD-11: 4A01-4B41]	63.23%	82.69%
Signal transducer and activator of transcription 3	T29130	Inflammation [ICD-11: 1A00-CA43.1]	63.23%	82.69%
Signal transducer and activator of transcription 3	T29130	Multiple myeloma [ICD-11: 2A83]	63.23%	82.69%
Signal transducer and activator of transcription 3	T29130	Psoriasis vulgaris [ICD-11: EA90]	63.23%	82.69%
Signal transducer and activator of transcription 3	T29130	Recurrent glioblastoma [ICD-11: 2A00.00]	63.23%	82.69%
Signal transducer and activator of transcription 3	T29130	Solid tumour/cancer [ICD-11: 2A00-2F9Z]	63.23%	82.69%
Signal transducer and activator of transcription 3	T29130	Ulcerative colitis [ICD-11: DD71]	63.23%	82.69%
Tyrosine-protein kinase receptor UFO	T82383	Acute myeloid leukaemia [ICD-11: 2A60]	63.11%	90.95%
Tyrosine-protein kinase receptor UFO	T82383	Breast cancer [ICD-11: 2C60-2C65]	63.11%	90.95%
Tyrosine-protein kinase receptor UFO	T82383	Metastatic malignant neoplasm [ICD-11: 2D50-2E09]	63.11%	90.95%
Tyrosine-protein kinase receptor UFO	T82383	Multiple myeloma [ICD-11: 2A83]	63.11%	90.95%
Tyrosine-protein kinase receptor UFO	T82383	Myelodysplastic syndrome [ICD-11: 2A37]	63.11%	90.95%
Tyrosine-protein kinase receptor UFO	T82383	Non-small-cell lung cancer [ICD-11: 2C25.Y]	63.11%	90.95%
Tyrosine-protein kinase receptor UFO	T82383	Solid tumour/cancer [ICD-11: 2A00-2F9Z]	63.11%	90.95%
Pregnane X receptor	T82702	Arteriosclerosis [ICD-11: BD40]	63.03%	94.73%
PI3-kinase p110-delta subunit	T67849	Arthritis [ICD-11: FA20]	62.75%	96.47%
PI3-kinase p110-delta subunit	T67849	Breast cancer [ICD-11: 2C60-2C65]	62.75%	96.47%
PI3-kinase p110-delta subunit	T67849	Chronic lymphocytic leukaemia [ICD-11: 2A82.0]	62.75%	96.47%
PI3-kinase p110-delta subunit	T67849	Coronavirus Disease 2019 (COVID-19) [ICD-11: 1D6Y]	62.75%	96.47%
PI3-kinase p110-delta subunit	T67849	Cutaneous T-cell lymphoma [ICD-11: 2B01]	62.75%	96.47%
PI3-kinase p110-delta subunit	T67849	Diffuse large B-cell lymphoma [ICD-11: 2A81]	62.75%	96.47%
PI3-kinase p110-delta subunit	T67849	Follicular lymphoma [ICD-11: 2A80]	62.75%	96.47%
PI3-kinase p110-delta subunit	T67849	Follicular lymphoma [ICD-11: 2A80]	62.75%	96.47%
PI3-kinase p110-delta subunit	T67849	Hematologic tumour [ICD-11: 2B33.Y]	62.75%	96.47%
PI3-kinase p110-delta subunit	T67849	Lymphoma [ICD-11: 2A80-2A86]	62.75%	96.47%
PI3-kinase p110-delta subunit	T67849	Mantle cell lymphoma [ICD-11: 2A85.5]	62.75%	96.47%

Target Name	ChEMBL-ID	Indication	Probability	Model accuracy
PI3-kinase p110-delta subunit	T67849	Marginal zone lymphoma [ICD-11: 2A85.0]	62.75%	96.47%
PI3-kinase p110-delta subunit	T67849	Myeloid leukaemia [ICD-11: 2B33.1]	62.75%	96.47%
PI3-kinase p110-delta subunit	T67849	Non-hodgkin lymphoma [ICD-11: 2B33.5]	62.75%	96.47%
PI3-kinase p110-delta subunit	T67849	Solid tumour/cancer [ICD-11: 2A00-2F9Z]	62.75%	96.47%
Platelet-derived growth factor receptor alpha	T53524	Acute myelogenous leukaemia [ICD-11: 2A41]	62.3%	91.07%
Platelet-derived growth factor receptor alpha	T53524	Acute myeloid leukaemia [ICD-11: 2A60]	62.3%	91.07%
Platelet-derived growth factor receptor alpha	T53524	Breast cancer [ICD-11: 2C60-2C65]	62.3%	91.07%
Platelet-derived growth factor receptor alpha	T53524	Colon cancer [ICD-11: 2B90.Z]	62.3%	91.07%
Platelet-derived growth factor receptor alpha	T53524	Colorectal cancer [ICD-11: 2B91.Z]	62.3%	91.07%
Platelet-derived growth factor receptor alpha	T53524	Gastric adenocarcinoma [ICD-11: 2B72]	62.3%	91.07%
Platelet-derived growth factor receptor alpha	T53524	Gastrointestinal cancer [ICD-11: 2C11]	62.3%	91.07%
Platelet-derived growth factor receptor alpha	T53524	Gastrointestinal stromal tumour [ICD-11: 2B5B]	62.3%	91.07%
Platelet-derived growth factor receptor alpha	T53524	Glioblastoma multiforme [ICD-11: 2A00.0]	62.3%	91.07%
Platelet-derived growth factor receptor alpha	T53524	Gram-positive bacterial infection [ICD-11: 1B74-1G40]	62.3%	91.07%
Platelet-derived growth factor receptor alpha	T53524	Lung cancer [ICD-11: 2C25.0]	62.3%	91.07%
Platelet-derived growth factor receptor alpha	T53524	Mast cell leukaemia [ICD-11: 2A21.00]	62.3%	91.07%
Platelet-derived growth factor receptor alpha	T53524	Multiple myeloma [ICD-11: 2A83]	62.3%	91.07%
Platelet-derived growth factor receptor alpha	T53524	Myelodysplastic syndrome [ICD-11: 2A37]	62.3%	91.07%
Platelet-derived growth factor receptor alpha	T53524	Non-small-cell lung cancer [ICD-11: 2C25.Y]	62.3%	91.07%
Platelet-derived growth factor receptor alpha	T53524	Pancreatic cancer [ICD-11: 2C10]	62.3%	91.07%
Platelet-derived growth factor receptor alpha	T53524	Prostate cancer [ICD-11: 2C82.0]	62.3%	91.07%
Platelet-derived growth factor receptor alpha	T53524	Recurrent glioblastoma [ICD-11: 2A00.00]	62.3%	91.07%
Platelet-derived growth factor receptor alpha	T53524	Soft tissue sarcoma [ICD-11: 2B57]	62.3%	91.07%
Platelet-derived growth factor receptor alpha	T53524	Solid tumour/cancer [ICD-11: 2A00-2F9Z]	62.3%	91.07%
Platelet-derived growth factor receptor alpha	T53524	Systemic mastocytosis [ICD-11: 2A21.0]	62.3%	91.07%
Platelet-derived growth factor receptor alpha	T53524	Thrombocytopenia [ICD-11: 3B64]	62.3%	91.07%
Sodium channel protein type III alpha subunit	T76937	Angina pectoris [ICD-11: BA40]	62.17%	96.9%

Target Name	ChEMBL-ID	Indication	Probability	Model accuracy
Sodium channel protein type III alpha subunit	T76937	Neurological disorder [ICD-11: 6B60]	62.17%	96.9%
Sodium channel protein type III alpha subunit	T76937	Pain [ICD-11: MG30-MG3Z]	62.17%	96.9%
Acetyl-CoA carboxylase 2	T08922	Bacterial infection [ICD-11: 1A00-1C4Z]	60.8%	98%
Acetyl-CoA carboxylase 2	T08922	Obesity [ICD-11: 5B81]	60.8%	98%
Acetyl-CoA carboxylase 2	T08922	Parkinson disease [ICD-11: 8A00.0]	60.8%	98%
Acetyl-CoA carboxylase 2	T08922	Sebaceous gland disease [ICD-11: EM0Z]	60.8%	98%
Acetyl-CoA carboxylase 2	T08922	Type-2 diabetes [ICD-11: 5A11]	60.8%	98%
Muscarinic acetylcholine receptor M4	T20709	Asthma [ICD-11: CA23]	60.67%	94.08%
Muscarinic acetylcholine receptor M4	T20709	Attention deficit hyperactivity disorder [ICD-11: 6A05.Z]	60.67%	94.08%
Muscarinic acetylcholine receptor M4	T20709	bronchial hyperreactivity [ICD-11: CB40]	60.67%	94.08%
Muscarinic acetylcholine receptor M4	T20709	Glaucoma/ocular hypertension [ICD-11: 9C61]	60.67%	94.08%
Muscarinic acetylcholine receptor M4	T20709	Mydriasis [ICD-11: LA11.62]	60.67%	94.08%
Muscarinic acetylcholine receptor M4	T20709	Nausea [ICD-11: MD90]	60.67%	94.08%
Macrophage migration inhibitory factor	T39977	Autoimmune diabetes [ICD-11: 5A10]	59.4%	80.78%
Macrophage migration inhibitory factor	T39977	Autoimmune disease [ICD-11: 4A40-4A45]	59.4%	80.78%
Macrophage migration inhibitory factor	T39977	Inflammation [ICD-11: 1A00-CA43.1]	59.4%	80.78%
Serotonin 2c (5-HT2c) receptor	T83813	Alcohol dependence [ICD-11: 6C40.2]	59.34%	89.62%
Serotonin 2c (5-HT2c) receptor	T83813	Alzheimer disease [ICD-11: 8A20]	59.34%	89.62%
Serotonin 2c (5-HT2c) receptor	T83813	Anxiety disorder [ICD-11: 6B00-6B0Z]	59.34%	89.62%
Serotonin 2c (5-HT2c) receptor	T83813	Attention deficit hyperactivity disorder [ICD-11: 6A05.Z]	59.34%	89.62%
Serotonin 2c (5-HT2c) receptor	T83813	Depression [ICD-11: 6A70-6A7Z]	59.34%	89.62%
Serotonin 2c (5-HT2c) receptor	T83813	Diabetic complication [ICD-11: 5A2Y]	59.34%	89.62%
Serotonin 2c (5-HT2c) receptor	T83813	Drug abuse [ICD-11: 6C4G.1Z]	59.34%	89.62%
Serotonin 2c (5-HT2c) receptor	T83813	Dyskinesia [ICD-11: MB47.4]	59.34%	89.62%
Serotonin 2c (5-HT2c) receptor	T83813	Generalized anxiety disorder [ICD-11: 6B00]	59.34%	89.62%
Serotonin 2c (5-HT2c) receptor	T83813	Hyperprolactinaemia [ICD-11: 5A60.1]	59.34%	89.62%
Serotonin 2c (5-HT2c) receptor	T83813	Major depressive disorder [ICD-11: 6A70.3]	59.34%	89.62%
Serotonin 2c (5-HT2c) receptor	T83813	Metabolic disorder [ICD-11: 5C50-5D2Z]	59.34%	89.62%
Serotonin 2c (5-HT2c) receptor	T83813	Migraine [ICD-11: 8A80]	59.34%	89.62%
Serotonin 2c (5-HT2c) receptor	T83813	Mood disorder [ICD-11: 6A60-6E23]	59.34%	89.62%
Serotonin 2c (5-HT2c) receptor	T83813	Neurological disorder [ICD-11: 6B60]	59.34%	89.62%
Serotonin 2c (5-HT2c) receptor	T83813	Non-hodgkin lymphoma [ICD-11:	59.34%	89.62%

Target Name	ChEMBL-ID	Indication	Probability	Model accuracy
		2B33.5]		
Serotonin 2c (5-HT2c) receptor	T83813	Obesity [ICD-11: 5B81]	59.34%	89.62%
Serotonin 2c (5-HT2c) receptor	T83813	Pain [ICD-11: MG30-MG3Z]	59.34%	89.62%
Serotonin 2c (5-HT2c) receptor	T83813	Parkinson disease [ICD-11: 8A00.0]	59.34%	89.62%
Serotonin 2c (5-HT2c) receptor	T83813	Primary insomnia [ICD-11: 7A00]	59.34%	89.62%
Serotonin 2c (5-HT2c) receptor	T83813	Psychotic disorder [ICD-11: 6A20-6A25]	59.34%	89.62%
Serotonin 2c (5-HT2c) receptor	T83813	Schizophrenia [ICD-11: 6A20]	59.34%	89.62%
Serotonin 2c (5-HT2c) receptor	T83813	Sleep-wake disorder [ICD-11: 7A00-7B2Z]	59.34%	89.62%
Cytochrome P450 3A4	T37848	Atopic dermatitis [ICD-11: EA80]	58.71%	91.19%
Cytochrome P450 3A4	T37848	Solid tumour/cancer [ICD-11: 2A00-2F9Z]	58.71%	91.19%
Cysteinyl leukotriene receptor 2	T74238	Asthma [ICD-11: CA23]	58.3%	98%
Sodium channel protein type IV alpha subunit	T02546	Musculoskeletal disorder [ICD-11: FA00-FC0Z]	57.02%	92.32%
Sodium channel protein type IV alpha subunit	T02546	Neurological disorder [ICD-11: 6B60]	57.02%	92.32%
Sodium channel protein type IV alpha subunit	T02546	Pain [ICD-11: MG30-MG3Z]	57.02%	92.32%
11-beta-hydroxysteroid dehydrogenase 2	T43721	Type-2 diabetes [ICD-11: 5A11]	56.22%	94.78%
Integrin alpha-5/beta-1	T29879	Allergic rhinitis [ICD-11: CA08.0]	55.99%	96.39%
Integrin alpha-5/beta-1	T29879	Allergy [ICD-11: 4A80-4A85]	55.99%	96.39%
Integrin alpha-5/beta-1	T29879	Asthma [ICD-11: CA23]	55.99%	96.39%
Integrin alpha-5/beta-1	T29879	Macular degeneration [ICD-11: 9B78.3]	55.99%	96.39%
Integrin alpha-5/beta-1	T29879	Macular degeneration [ICD-11: 9B78.3]	55.99%	96.39%
Integrin alpha-5/beta-1	T29879	Multiple sclerosis [ICD-11: 8A40]	55.99%	96.39%
Integrin alpha-5/beta-1	T29879	Non-small-cell lung cancer [ICD-11: 2C25.Y]	55.99%	96.39%
DNA topoisomerase I	T09826	Acquired immune deficiency syndrome [ICD-11: 1C62.3]	55.29%	97%
DNA topoisomerase I	T09826	Acute lymphoblastic leukaemia [ICD-11: 2A85]	55.29%	97%
DNA topoisomerase I	T09826	Bacterial infection [ICD-11: 1A00-1C4Z]	55.29%	97%
DNA topoisomerase I	T09826	Bladder cancer [ICD-11: 2C94]	55.29%	97%
DNA topoisomerase I	T09826	Breast cancer [ICD-11: 2C60-2C65]	55.29%	97%
DNA topoisomerase I	T09826	Colorectal cancer [ICD-11: 2B91.Z]	55.29%	97%
DNA topoisomerase I	T09826	Esophageal cancer [ICD-11: 2B70]	55.29%	97%
DNA topoisomerase I	T09826	Gastric adenocarcinoma [ICD-11: 2B72]	55.29%	97%
DNA topoisomerase I	T09826	Glioblastoma multiforme [ICD-11: 2A00.0]	55.29%	97%
DNA topoisomerase I	T09826	Human immunodeficiency virus infection [ICD-11: 1C62]	55.29%	97%
DNA topoisomerase I	T09826	Lung cancer [ICD-11: 2C25.0]	55.29%	97%
DNA topoisomerase I	T09826	Lymphoma [ICD-11: 2A80-2A86]	55.29%	97%

Target Name	ChEMBL-ID	Indication	Probability	Model accuracy
DNA topoisomerase I	T09826	Metastatic colorectal cancer [ICD-11: 2B91]	55.29%	97%
DNA topoisomerase I	T09826	Myelodysplastic syndrome [ICD-11: 2A37]	55.29%	97%
DNA topoisomerase I	T09826	Ovarian cancer [ICD-11: 2C73]	55.29%	97%
DNA topoisomerase I	T09826	Renal cell carcinoma [ICD-11: 2C90]	55.29%	97%
DNA topoisomerase I	T09826	Small-cell lung cancer [ICD-11: 2C25.Y]	55.29%	97%
DNA topoisomerase I	T09826	Solid tumour/cancer [ICD-11: 2A00-2F9Z]	55.29%	97%
Toll-like receptor 8	T48703	Allergic rhinitis [ICD-11: CA08.0]	55.11%	96.25%
Toll-like receptor 8	T48703	Allergy [ICD-11: 4A80-4A85]	55.11%	96.25%
Toll-like receptor 8	T48703	Autoimmune diabetes [ICD-11: 5A10]	55.11%	96.25%
Toll-like receptor 8	T48703	Diffuse large B-cell lymphoma [ICD-11: 2A81]	55.11%	96.25%
Toll-like receptor 8	T48703	Herpes simplex virus infection [ICD-11: 1F00]	55.11%	96.25%
Toll-like receptor 8	T48703	Melanoma [ICD-11: 2C30]	55.11%	96.25%
Toll-like receptor 8	T48703	Solid tumour/cancer [ICD-11: 2A00-2F9Z]	55.11%	96.25%
Toll-like receptor 8	T48703	Systemic lupus erythematosus [ICD-11: 4A40.0]	55.11%	96.25%
Cyclin-dependent kinase 1/cyclin B	T49898	Acute lymphoblastic leukaemia [ICD-11: 2A85]	54.86%	96%
Cyclin-dependent kinase 1/cyclin B	T49898	Allergic inflammation [ICD-11: 4A80-4A85]	54.86%	96%
Cyclin-dependent kinase 1/cyclin B	T49898	Breast cancer [ICD-11: 2C60-2C65]	54.86%	96%
Cyclin-dependent kinase 1/cyclin B	T49898	Graft rejection [ICD-11: NE84]	54.86%	96%
Cyclin-dependent kinase 1/cyclin B	T49898	Haematological malignancy [ICD-11: 2B33.Y]	54.86%	96%
Cyclin-dependent kinase 1/cyclin B	T49898	Mantle cell lymphoma [ICD-11: 2A85.5]	54.86%	96%
Cyclin-dependent kinase 1/cyclin B	T49898	Nasopharyngeal carcinoma [ICD-11: 2B6B]	54.86%	96%
Cyclin-dependent kinase 1/cyclin B	T49898	Non-small-cell lung cancer [ICD-11: 2C25.Y]	54.86%	96%
Cyclin-dependent kinase 1/cyclin B	T49898	Obesity [ICD-11: 5B81]	54.86%	96%
Cyclin-dependent kinase 1/cyclin B	T49898	Pain [ICD-11: MG30-MG3Z]	54.86%	96%
Cyclin-dependent kinase 1/cyclin B	T49898	Solid tumour/cancer [ICD-11: 2A00-2F9Z]	54.86%	96%
Protein-tyrosine phosphatase 2C	T13057	Solid tumour/cancer [ICD-11: 2A00-2F9Z]	54.54%	94.42%
Tissue factor pathway inhibitor	T78890	Factor IX deficiency [ICD-11: 3B11]	54.31%	97.33%
Tissue factor pathway inhibitor	T78890	Sepsis [ICD-11: 1G40-1G41]	54.31%	97.33%
C-X-C chemokine receptor type 4	T96079	Acute lymphoblastic leukaemia [ICD-11: 2A85]	53.32%	93.1%
C-X-C chemokine receptor type 4	T96079	Acute myeloid leukaemia [ICD-11: 2A60]	53.32%	93.1%
C-X-C chemokine receptor type 4	T96079	Autoimmune diabetes [ICD-11:	53.32%	93.1%

Target Name	ChEMBL-ID	Indication	Probability	Model accuracy
		5A10]		
C-X-C chemokine receptor type 4	T96079	B-cell chronic lymphocytic leukaemia [ICD-11: 2A82.00]	53.32%	93.1%
C-X-C chemokine receptor type 4	T96079	Bone marrow transplantation [ICD-11: QB63.6]	53.32%	93.1%
C-X-C chemokine receptor type 4	T96079	Breast cancer [ICD-11: 2C60-2C65]	53.32%	93.1%
C-X-C chemokine receptor type 4	T96079	Constitutional neutropenia [ICD-11: 4B00.0]	53.32%	93.1%
C-X-C chemokine receptor type 4	T96079	Haematological malignancy [ICD-11: 2B33.Y]	53.32%	93.1%
C-X-C chemokine receptor type 4	T96079	Human immunodeficiency virus infection [ICD-11: 1C62]	53.32%	93.1%
C-X-C chemokine receptor type 4	T96079	Macular degeneration [ICD-11: 9B78.3]	53.32%	93.1%
C-X-C chemokine receptor type 4	T96079	Melanoma [ICD-11: 2C30]	53.32%	93.1%
C-X-C chemokine receptor type 4	T96079	Merkel cell carcinoma [ICD-11: 2C34]	53.32%	93.1%
C-X-C chemokine receptor type 4	T96079	Multiple myeloma [ICD-11: 2A83]	53.32%	93.1%
C-X-C chemokine receptor type 4	T96079	Myelodysplastic syndrome [ICD-11: 2A37]	53.32%	93.1%
C-X-C chemokine receptor type 4	T96079	Non-hodgkin lymphoma [ICD-11: 2B33.5]	53.32%	93.1%
C-X-C chemokine receptor type 4	T96079	Pancreatic cancer [ICD-11: 2C10]	53.32%	93.1%
C-X-C chemokine receptor type 4	T96079	Peripheral vascular disease [ICD-11: BD4Z]	53.32%	93.1%
C-X-C chemokine receptor type 4	T96079	Psoriasis vulgaris [ICD-11: EA90]	53.32%	93.1%
C-X-C chemokine receptor type 4	T96079	Recurring respiratory infection [ICD-11: CA07-CA45]	53.32%	93.1%
C-X-C chemokine receptor type 4	T96079	Renal cell carcinoma [ICD-11: 2C90]	53.32%	93.1%
C-X-C chemokine receptor type 4	T96079	Sarcoma [ICD-11: 2A60-2C35]	53.32%	93.1%
C-X-C chemokine receptor type 4	T96079	Solid tumour/cancer [ICD-11: 2A00-2F9Z]	53.32%	93.1%
Plasminogen activator inhibitor-1	T15556	Asthma [ICD-11: CA23]	51.71%	83%
Plasminogen activator inhibitor-1	T15556	Solid tumour/cancer [ICD-11: 2A00-2F9Z]	51.71%	83%
Plasminogen activator inhibitor-1	T15556	Thrombosis [ICD-11: DB61-GB90]	51.71%	83%
Nicotinamide phosphoribosyltransferase	T54582	leukaemia [ICD-11: 2A60-2B33]	51.5%	96.25%
Nicotinamide phosphoribosyltransferase	T54582	Solid tumour/cancer [ICD-11: 2A00-2F9Z]	51.5%	96.25%
Acetylcholinesterase	T30082	Alcohol dependence [ICD-11: 6C40.2]	50.58%	94.45%
Acetylcholinesterase	T30082	Alzheimer disease [ICD-11: 8A20]	50.58%	94.45%
Acetylcholinesterase	T30082	Chronic glaucoma [ICD-11: 9C61.0Z]	50.58%	94.45%
Acetylcholinesterase	T30082	Cognitive impairment [ICD-11: 6D71]	50.58%	94.45%
Acetylcholinesterase	T30082	Dementia [ICD-11: 6D80-6D86]	50.58%	94.45%
Acetylcholinesterase	T30082	Functional dyspepsia [ICD-11: DD90.3]	50.58%	94.45%

Target Name	ChEMBL-ID	Indication	Probability	Model accuracy
Acetylcholinesterase	T30082	Helminth infection [ICD-11: 1F90.0]	50.58%	94.45%
Acetylcholinesterase	T30082	Myasthenia gravis [ICD-11: 8C6Y]	50.58%	94.45%
Acetylcholinesterase	T30082	Neurodegenerative disorder [ICD-11: 8A20-8A23]	50.58%	94.45%
Acetylcholinesterase	T30082	Open-angle glaucoma [ICD-11: 9C61]	50.58%	94.45%
Acetylcholinesterase	T30082	Pain [ICD-11: MG30-MG3Z]	50.58%	94.45%
Acetylcholinesterase	T30082	Parkinson disease [ICD-11: 8A00.0]	50.58%	94.45%
Acetylcholinesterase	T30082	Pediculus capitis infestation [ICD-11: 1G00.0]	50.58%	94.45%
Acetylcholinesterase	T30082	Poisoning due to pesticides and chemicals [ICD-11: NE6Z]	50.58%	94.45%
Acetylcholinesterase	T30082	Solid tumour/cancer [ICD-11: 2A00-2F9Z]	50.58%	94.45%
Acetylcholinesterase	T30082	Urinary dysfunction [ICD-11: GC2Z]	50.58%	94.45%
Acetylcholinesterase	T30082	Xerostomia [ICD-11: DA02.1]	50.58%	94.45%
