# Supplementary material for: 3,4-Dihydropyrimidin-2(1H)-One Hybrids as Phenotypic Anti-Breast Cancer Agents: Synthesis, Colony-Formation Profiling and Computational Target Deconvolution
Source: Int J Mol Sci. 2026 Oct 27;27(21):9618. doi: 10.3390/ijms27219618 (PMC13763226; doi:10.3390/ijms27219618)
Supplement: Supplementary file 1 [file ijms-27-09618-s001.zip › Super-PRED_exported_data.zip!/Targets_4a.pdf]

Exported data

Target Name	ChEMBL-ID	Indication	Probability	Model accuracy
Cathepsin D	T67102	Hypertension [ICD-11: BA00-BA04]	95.34%	98.95%
Cathepsin D	T67102	Multiple sclerosis [ICD-11: 8A40]	95.34%	98.95%
Dihydrofolate reductase	T17345	Acute bacterial skin infection [ICD-11: 1C41]	86.11%	89.92%
Dihydrofolate reductase	T17345	Bacterial infection [ICD-11: 1A00-1C4Z]	86.11%	89.92%
Dihydrofolate reductase	T17345	Bladder cancer [ICD-11: 2C94]	86.11%	89.92%
Dihydrofolate reductase	T17345	Inflammatory bowel disease [ICD-11: DD72]	86.11%	89.92%
Dihydrofolate reductase	T17345	Pneumonia [ICD-11: CA40]	86.11%	89.92%
Dihydrofolate reductase	T17345	Pulmonary and extrapulmonary tuberculosis [ICD-11: 1B10.Z]	86.11%	89.92%
Dihydrofolate reductase	T17345	Rheumatoid arthritis [ICD-11: FA20]	86.11%	89.92%
Dihydrofolate reductase	T17345	Solid tumour/cancer [ICD-11: 2A00-2F9Z]	86.11%	89.92%
Dihydrofolate reductase	T17345	Urinary tract infection [ICD-11: GC08]	86.11%	89.92%
Tyrosine-protein kinase receptor UFO	T82383	Acute myeloid leukaemia [ICD-11: 2A60]	85.06%	90.95%
Tyrosine-protein kinase receptor UFO	T82383	Breast cancer [ICD-11: 2C60-2C65]	85.06%	90.95%
Tyrosine-protein kinase receptor UFO	T82383	Metastatic malignant neoplasm [ICD-11: 2D50-2E09]	85.06%	90.95%
Tyrosine-protein kinase receptor UFO	T82383	Multiple myeloma [ICD-11: 2A83]	85.06%	90.95%
Tyrosine-protein kinase receptor UFO	T82383	Myelodysplastic syndrome [ICD-11: 2A37]	85.06%	90.95%
Tyrosine-protein kinase receptor UFO	T82383	Non-small-cell lung cancer [ICD-11: 2C25.Y]	85.06%	90.95%
Tyrosine-protein kinase receptor UFO	T82383	Solid tumour/cancer [ICD-11: 2A00-2F9Z]	85.06%	90.95%
TGF-beta receptor type II	T49989	Solid tumour/cancer [ICD-11: 2A00-2F9Z]	84.65%	88.18%
TGF-beta receptor type II	T49989	Wound healing [ICD-11: EL8Y]	84.65%	88.18%
Casein kinase II alpha/beta	T51565	Cholangiocarcinoma [ICD-11: 2C12.10]	83.88%	99.23%
Casein kinase II alpha/beta	T51565	Solid tumour/cancer [ICD-11: 2A00-2F9Z]	83.88%	99.23%
Beta-glucuronidase	T96413	Mucopolysaccharidosis [ICD-11: 5C56.3]	83.28%	77.68%
Beta-glucuronidase	T96413	Periodontal disease [ICD-11: DA0C]	83.28%	77.68%
Anandamide amidohydrolase	T11754	Anaesthesia [ICD-11: 9A78.6]	83.07%	97.53%
Anandamide amidohydrolase	T11754	Anxiety disorder [ICD-11: 6B00-6B0Z]	83.07%	97.53%
Anandamide amidohydrolase	T11754	Inflammation [ICD-11: 1A00-CA43.1]	83.07%	97.53%
Anandamide amidohydrolase	T11754	Liver disease [ICD-11: DB90-BD99]	83.07%	97.53%
Anandamide amidohydrolase	T11754	Major depressive disorder [ICD-11: 6A70.3]	83.07%	97.53%
Anandamide amidohydrolase	T11754	Neuropathic pain [ICD-11: 8E43.0]	83.07%	97.53%
Anandamide amidohydrolase	T11754	Pain [ICD-11: MG30-MG3Z]	83.07%	97.53%
Anandamide amidohydrolase	T11754	Social anxiety disorder [ICD-11:	83.07%	97.53%

Target Name	ChEMBL-ID	Indication	Probability	Model accuracy
		6B04]		
GABA-A receptor; alpha-1/beta-2/gamma-2	T08910	Alcohol dependence [ICD-11: 6C40.2]	82.03%	92.67%
GABA-A receptor; alpha-1/beta-2/gamma-2	T08910	Anaesthesia [ICD-11: 9A78.6]	82.03%	92.67%
GABA-A receptor; alpha-1/beta-2/gamma-2	T08910	Anxiety disorder [ICD-11: 6B00-6B0Z]	82.03%	92.67%
GABA-A receptor; alpha-1/beta-2/gamma-2	T08910	Anxiety disorder [ICD-11: 6B00-6B0Z]	82.03%	92.67%
GABA-A receptor; alpha-1/beta-2/gamma-2	T08910	Anxiety disorder [ICD-11: 6B00-6B0Z]	82.03%	92.67%
GABA-A receptor; alpha-1/beta-2/gamma-2	T08910	Chronic pain [ICD-11: MG30]	82.03%	92.67%
GABA-A receptor; alpha-1/beta-2/gamma-2	T08910	Cystitis [ICD-11: GC00]	82.03%	92.67%
GABA-A receptor; alpha-1/beta-2/gamma-2	T08910	Depression [ICD-11: 6A70-6A7Z]	82.03%	92.67%
GABA-A receptor; alpha-1/beta-2/gamma-2	T08910	Depression [ICD-11: 6A70-6A7Z]	82.03%	92.67%
GABA-A receptor; alpha-1/beta-2/gamma-2	T08910	Depression [ICD-11: 6A70-6A7Z]	82.03%	92.67%
GABA-A receptor; alpha-1/beta-2/gamma-2	T08910	Epilepsy [ICD-11: 8A60-8A68]	82.03%	92.67%
GABA-A receptor; alpha-1/beta-2/gamma-2	T08910	Epileptic seizures [ICD-11: 8A61-8A6Z]	82.03%	92.67%
GABA-A receptor; alpha-1/beta-2/gamma-2	T08910	Epileptic seizures [ICD-11: 8A61-8A6Z]	82.03%	92.67%
GABA-A receptor; alpha-1/beta-2/gamma-2	T08910	Epileptic seizures [ICD-11: 8A61-8A6Z]	82.03%	92.67%
GABA-A receptor; alpha-1/beta-2/gamma-2	T08910	Essential tremor or related tremors [ICD-11: 8A04.1]	82.03%	92.67%
GABA-A receptor; alpha-1/beta-2/gamma-2	T08910	Essential tremor or related tremors [ICD-11: 8A04.1]	82.03%	92.67%
GABA-A receptor; alpha-1/beta-2/gamma-2	T08910	Essential tremor or related tremors [ICD-11: 8A04.1]	82.03%	92.67%
GABA-A receptor; alpha-1/beta-2/gamma-2	T08910	Fibromyalgia [ICD-11: MG30.01]	82.03%	92.67%
GABA-A receptor; alpha-1/beta-2/gamma-2	T08910	Headache [ICD-11: 8A80-8A84]	82.03%	92.67%
GABA-A receptor; alpha-1/beta-2/gamma-2	T08910	Inflammation [ICD-11: 1A00-CA43.1]	82.03%	92.67%
GABA-A receptor; alpha-1/beta-2/gamma-2	T08910	Inflammation [ICD-11: 1A00-CA43.1]	82.03%	92.67%
GABA-A receptor; alpha-1/beta-2/gamma-2	T08910	Inflammation [ICD-11: 1A00-CA43.1]	82.03%	92.67%
GABA-A receptor; alpha-1/beta-2/gamma-2	T08910	Inflammatory bowel disease [ICD-11: DD72]	82.03%	92.67%
GABA-A receptor; alpha-1/beta-2/gamma-2	T08910	Inflammatory bowel disease [ICD-11: DD72]	82.03%	92.67%
GABA-A receptor; alpha-1/beta-2/gamma-2	T08910	Inflammatory bowel disease [ICD-11: DD72]	82.03%	92.67%
GABA-A receptor; alpha-1/beta-2/gamma-2	T08910	Insomnia [ICD-11: 7A00-7A0Z]	82.03%	92.67%

Target Name	ChEMBL-ID	Indication	Probability	Model accuracy
GABA-A receptor; alpha-1/beta-2/ gamma-2	T08910	Insomnia [ICD-11: 7A00-7A0Z]	82.03%	92.67%
GABA-A receptor; alpha-1/beta-2/ gamma-2	T08910	Intractable insomnia [ICD-11: 7A00]	82.03%	92.67%
GABA-A receptor; alpha-1/beta-2/ gamma-2	T08910	Ischemic reperfusion injury [ICD-11: DB98.B]	82.03%	92.67%
GABA-A receptor; alpha-1/beta-2/ gamma-2	T08910	Malaria [ICD-11: 1F40-1F45]	82.03%	92.67%
GABA-A receptor; alpha-1/beta-2/ gamma-2	T08910	Muscle spasm [ICD-11: MB47.3]	82.03%	92.67%
GABA-A receptor; alpha-1/beta-2/ gamma-2	T08910	Muscle spasm [ICD-11: MB47.3]	82.03%	92.67%
GABA-A receptor; alpha-1/beta-2/ gamma-2	T08910	Muscle spasm [ICD-11: MB47.3]	82.03%	92.67%
GABA-A receptor; alpha-1/beta-2/ gamma-2	T08910	Postpartum depression [ICD-11: 6E20.0]	82.03%	92.67%
GABA-A receptor; alpha-1/beta-2/ gamma-2	T08910	Postpartum depression [ICD-11: 6E20.0]	82.03%	92.67%
GABA-A receptor; alpha-1/beta-2/ gamma-2	T08910	Postpartum depression [ICD-11: 6E20.0]	82.03%	92.67%
GABA-A receptor; alpha-1/beta-2/ gamma-2	T08910	Premenstrual syndrome [ICD-11: GA34.40]	82.03%	92.67%
GABA-A receptor; alpha-1/beta-2/ gamma-2	T08910	Premenstrual syndrome [ICD-11: GA34.40]	82.03%	92.67%
GABA-A receptor; alpha-1/beta-2/ gamma-2	T08910	Premenstrual syndrome [ICD-11: GA34.40]	82.03%	92.67%
GABA-A receptor; alpha-1/beta-2/ gamma-2	T08910	Respiratory distress syndrome [ICD-11: CB00]	82.03%	92.67%
GABA-A receptor; alpha-1/beta-2/ gamma-2	T08910	Status epilepticus seizure [ICD-11: 8A66.1Y]	82.03%	92.67%
GABA-A receptor; alpha-1/beta-2/ gamma-2	T08910	Status epilepticus seizure [ICD-11: 8A66.1Y]	82.03%	92.67%
GABA-A receptor; alpha-1/beta-2/ gamma-2	T08910	Status epilepticus seizure [ICD-11: 8A66.1Y]	82.03%	92.67%
DNA-(apurinic or apyrimidinic site) lyase	T13348	Glioma [ICD-11: 2A00.0]	81.45%	91.11%
DNA-(apurinic or apyrimidinic site) lyase	T13348	Melanoma [ICD-11: 2C30]	81.45%	91.11%
DNA-(apurinic or apyrimidinic site) lyase	T13348	Ocular cancer [ICD-11: 2D00-2D07]	81.45%	91.11%
DNA-(apurinic or apyrimidinic site) lyase	T13348	Solid tumour/cancer [ICD-11: 2A00-2F9Z]	81.45%	91.11%
PI3-kinase p110-alpha/p85-alpha	T80276	Breast cancer [ICD-11: 2C60-2C65]	80.61%	94.33%
PI3-kinase p110-alpha/p85-alpha	T80276	Follicular lymphoma [ICD-11: 2A80]	80.61%	94.33%
PI3-kinase p110-alpha/p85-alpha	T80276	Non-hodgkin lymphoma [ICD-11: 2B33.5]	80.61%	94.33%
PI3-kinase p110-alpha/p85-alpha	T80276	Prostate cancer [ICD-11: 2C82.0]	80.61%	94.33%
PI3-kinase p110-alpha/p85-alpha	T80276	Solid tumour/cancer [ICD-11: 2A00-2F9Z]	80.61%	94.33%
Protein-tyrosine phosphatase 2C	T13057	Solid tumour/cancer [ICD-11: 2A00-2F9Z]	80.25%	94.42%
Cathepsin L	T41141	Abdominal aortic aneurysm [ICD-11:	79.68%	96.61%

Target Name	ChEMBL-ID	Indication	Probability	Model accuracy
		BD50.4]		
Cathepsin L	T41141	Asthma [ICD-11: CA23]	79.68%	96.61%
Cathepsin L	T41141	Bone cancer [ICD-11: 2B5Z]	79.68%	96.61%
Cathepsin L	T41141	Cancer related pain [ICD-11: MG30]	79.68%	96.61%
Cathepsin L	T41141	Chronic obstructive pulmonary disease [ICD-11: CA22]	79.68%	96.61%
Cathepsin L	T41141	Cirrhosis [ICD-11: DB93]	79.68%	96.61%
Cathepsin L	T41141	Coagulation defect [ICD-11: 3B10.0]	79.68%	96.61%
Cathepsin L	T41141	Coronavirus infection [ICD-11: 1D92]	79.68%	96.61%
Cathepsin L	T41141	Gastrointestinal disease [ICD-11: DE2Z]	79.68%	96.61%
Cathepsin L	T41141	Glioma [ICD-11: 2A00.0]	79.68%	96.61%
Cathepsin L	T41141	Hair loss [ICD-11: ED70]	79.68%	96.61%
Cathepsin L	T41141	Henipavirus Encephalitis [ICD-11: 1D63]	79.68%	96.61%
Cathepsin L	T41141	Middle East Respiratory Syndrome (MERS) [ICD-11: 1D64]	79.68%	96.61%
Cathepsin L	T41141	Multiple sclerosis [ICD-11: 8A40]	79.68%	96.61%
Cathepsin L	T41141	Psoriasis vulgaris [ICD-11: EA90]	79.68%	96.61%
Cathepsin L	T41141	Rheumatoid arthritis [ICD-11: FA20]	79.68%	96.61%
Cathepsin L	T41141	Severe acute respiratory syndrome (SARS) [ICD-11: 1D65]	79.68%	96.61%
Cathepsin L	T41141	Solid tumour/cancer [ICD-11: 2A00-2F9Z]	79.68%	96.61%
G-protein coupled bile acid receptor 1	T86273	Metabolic disorder [ICD-11: 5C50-5D2Z]	79.03%	93.65%
G-protein coupled bile acid receptor 1	T86273	Type-2 diabetes [ICD-11: 5A11]	79.03%	93.65%
Sodium/hydrogen exchanger 1	T82028	Angina pectoris [ICD-11: BA40]	78.96%	90.24%
Sodium/hydrogen exchanger 1	T82028	Cardiac arrhythmias [ICD-11: BC9Z]	78.96%	90.24%
Sodium/hydrogen exchanger 1	T82028	Heart arrhythmia [ICD-11: BC65]	78.96%	90.24%
Sodium/hydrogen exchanger 1	T82028	Myocardial infarction [ICD-11: BA41-BA43]	78.96%	90.24%
Transient receptor potential cation channel subfamily A member 1	T84040	Diabetic neuropathy [ICD-11: 8C0Z]	76.99%	92.17%
Transient receptor potential cation channel subfamily A member 1	T84040	Pain [ICD-11: MG30-MG3Z]	76.99%	92.17%
Transient receptor potential cation channel subfamily A member 1	T84040	Throat irritation [ICD-11: CA0Y]	76.99%	92.17%
HERG	T20251	Angina pectoris [ICD-11: BA40]	75.56%	89.76%
HERG	T20251	Cardiac arrhythmias [ICD-11: BC9Z]	75.56%	89.76%
HERG	T20251	Cardiac failure [ICD-11: BD10-BD13]	75.56%	89.76%
HERG	T20251	Heart arrhythmia [ICD-11: BC65]	75.56%	89.76%
HERG	T20251	Malaria [ICD-11: 1F40-1F45]	75.56%	89.76%
HERG	T20251	Multiple sclerosis [ICD-11: 8A40]	75.56%	89.76%
HERG	T20251	Ovarian cancer [ICD-11: 2C73]	75.56%	89.76%
HERG	T20251	Pain [ICD-11: MG30-MG3Z]	75.56%	89.76%
HERG	T20251	Sleep-wake disorder [ICD-11: 7A00-7B2Z]	75.56%	89.76%
ADAM10	T31902	Breast cancer [ICD-11: 2C60-2C65]	74.84%	97.5%

Target Name	ChEMBL-ID	Indication	Probability	Model accuracy
ADAM10	T31902	Solid tumour/cancer [ICD-11: 2A00-2F9Z]	74.84%	97.5%
Nicotinamide phosphoribosyltransferase	T54582	leukaemia [ICD-11: 2A60-2B33]	74.61%	96.25%
Nicotinamide phosphoribosyltransferase	T54582	Solid tumour/cancer [ICD-11: 2A00-2F9Z]	74.61%	96.25%
Glutamate receptor ionotropic, AMPA 2	T42392	Diabetic neuropathy [ICD-11: 8C0Z]	74.03%	86.92%
Glutamate receptor ionotropic, AMPA 2	T42392	Parkinson disease [ICD-11: 8A00.0]	74.03%	86.92%
Cytochrome P450 3A4	T37848	Atopic dermatitis [ICD-11: EA80]	73.39%	91.19%
Cytochrome P450 3A4	T37848	Solid tumour/cancer [ICD-11: 2A00-2F9Z]	73.39%	91.19%
C5a anaphylatoxin chemotactic receptor	T15439	Atopic dermatitis [ICD-11: EA80]	73.29%	92.62%
C5a anaphylatoxin chemotactic receptor	T15439	Eosinophilic granulomatosis with polyangiitis [ICD-11: 4A44.A2]	73.29%	92.62%
C5a anaphylatoxin chemotactic receptor	T15439	Hereditary haemolytic anemia [ICD-11: 3A10.3]	73.29%	92.62%
C5a anaphylatoxin chemotactic receptor	T15439	Inflammation [ICD-11: 1A00-CA43.1]	73.29%	92.62%
C5a anaphylatoxin chemotactic receptor	T15439	Rheumatoid arthritis [ICD-11: FA20]	73.29%	92.62%
C5a anaphylatoxin chemotactic receptor	T15439	Vasculitis [ICD-11: 4A44]	73.29%	92.62%
Histone deacetylase 8	T28887	Solid tumour/cancer [ICD-11: 2A00-2F9Z]	73.2%	93.99%
Aurora kinase B/Inner centromere protein	T46781	Acute myeloid leukaemia [ICD-11: 2A60]	73.11%	97.5%
Aurora kinase B/Inner centromere protein	T46781	Diabetic complication [ICD-11: 5A2Y]	73.11%	97.5%
Aurora kinase B/Inner centromere protein	T46781	Haematological malignancy [ICD-11: 2B33.Y]	73.11%	97.5%
Aurora kinase B/Inner centromere protein	T46781	Prostate cancer [ICD-11: 2C82.0]	73.11%	97.5%
Aurora kinase B/Inner centromere protein	T46781	Solid tumour/cancer [ICD-11: 2A00-2F9Z]	73.11%	97.5%
Adenosine A2b receptor	T86679	Acute and chronic heart failure [ICD-11: BD1Z]	72.68%	98.59%
Adenosine A2b receptor	T86679	Alzheimer disease [ICD-11: 8A20]	72.68%	98.59%
Adenosine A2b receptor	T86679	Apnea [ICD-11: MD11.0]	72.68%	98.59%
Adenosine A2b receptor	T86679	Asthma [ICD-11: CA23]	72.68%	98.59%
Adenosine A2b receptor	T86679	Herpes simplex virus infection [ICD-11: 1F00]	72.68%	98.59%
Adenosine A2b receptor	T86679	Hypertension [ICD-11: BA00-BA04]	72.68%	98.59%
Adenosine A2b receptor	T86679	Multiple sclerosis [ICD-11: 8A40]	72.68%	98.59%
Adenosine A2b receptor	T86679	Non-insulin dependent diabetes [ICD-11: 5A11]	72.68%	98.59%
Adenosine A2b receptor	T86679	Non-small-cell lung cancer [ICD-11: 2C25.Y]	72.68%	98.59%
Adenosine A2b receptor	T86679	Parkinson disease [ICD-11: 8A00.0]	72.68%	98.59%
Adenosine A2b receptor	T86679	Paroxysmal supraventricular	72.68%	98.59%

Target Name	ChEMBL-ID	Indication	Probability	Model accuracy
		tachycardia [ICD-11: BC81.Z]		
Adenosine A2b receptor	T86679	Solid tumour/cancer [ICD-11: 2A00-2F9Z]	72.68%	98.59%
Hexokinase type IV	T87166	Diabetic complication [ICD-11: 5A2Y]	71.51%	91.96%
Hexokinase type IV	T87166	Obesity [ICD-11: 5B81]	71.51%	91.96%
Hexokinase type IV	T87166	Type-2 diabetes [ICD-11: 5A11]	71.51%	91.96%
Cyclin-dependent kinase 4/cyclin D1	T85799	Breast cancer [ICD-11: 2C60-2C65]	71.36%	98.99%
Cyclin-dependent kinase 4/cyclin D1	T85799	Haematological malignancy [ICD-11: 2B33.Y]	71.36%	98.99%
Cyclin-dependent kinase 4/cyclin D1	T85799	Mantle cell lymphoma [ICD-11: 2A85.5]	71.36%	98.99%
Cyclin-dependent kinase 4/cyclin D1	T85799	Myelofibrosis [ICD-11: 2A20.2]	71.36%	98.99%
Cyclin-dependent kinase 4/cyclin D1	T85799	Psoriasis vulgaris [ICD-11: EA90]	71.36%	98.99%
Cyclin-dependent kinase 4/cyclin D1	T85799	Relapsed or refractory chronic lymphocytic leukaemia [ICD-11: 2A82.0]	71.36%	98.99%
Cyclin-dependent kinase 4/cyclin D1	T85799	Retinoblastoma [ICD-11: 2D02.2]	71.36%	98.99%
Cyclin-dependent kinase 4/cyclin D1	T85799	Schizophrenia [ICD-11: 6A20]	71.36%	98.99%
Cyclin-dependent kinase 4/cyclin D1	T85799	Small-cell lung cancer [ICD-11: 2C25.Y]	71.36%	98.99%
Cyclin-dependent kinase 4/cyclin D1	T85799	Solid tumour/cancer [ICD-11: 2A00-2F9Z]	71.36%	98.99%
Cyclin-dependent kinase 4/cyclin D1	T85799	Solid tumour/cancer [ICD-11: 2A00-2F9Z]	71.36%	98.99%
Formyl peptide receptor 1	T87831	Inflammation [ICD-11: 1A00-CA43.1]	71.22%	93.56%
Formyl peptide receptor 1	T87831	Peptic ulcer [ICD-11: DA61]	71.22%	93.56%
Sphingosine 1-phosphate receptor Edg-8	T50089	Multiple sclerosis [ICD-11: 8A40]	69.14%	100%
Cysteinyl leukotriene receptor 2	T74238	Asthma [ICD-11: CA23]	68.67%	98%
Glutaminase kidney isoform, mitochondrial	T86734	Breast cancer [ICD-11: 2C60-2C65]	68.14%	100%
Glutaminase kidney isoform, mitochondrial	T86734	leukaemia [ICD-11: 2A60-2B33]	68.14%	100%
Glutaminase kidney isoform, mitochondrial	T86734	Renal cell carcinoma [ICD-11: 2C90]	68.14%	100%
Glutaminase kidney isoform, mitochondrial	T86734	Solid tumour/cancer [ICD-11: 2A00-2F9Z]	68.14%	100%
DNA topoisomerase II alpha	T17048	Solid tumour/cancer [ICD-11: 2A00-2F9Z]	66.92%	89%
Platelet-derived growth factor receptor	T53524	Acute myelogenous leukaemia [ICD-11: 2A41]	66.88%	71.67%
Platelet-derived growth factor receptor	T53524	Acute myeloid leukaemia [ICD-11: 2A60]	66.88%	71.67%
Platelet-derived growth factor receptor	T53524	Alzheimer disease [ICD-11: 8A20]	66.88%	71.67%
Platelet-derived growth factor receptor	T53524	Brain injury [ICD-11: NA07.Z]	66.88%	71.67%
Platelet-derived growth factor receptor	T53524	Breast cancer [ICD-11: 2C60-2C65]	66.88%	71.67%
Platelet-derived growth factor	T53524	Colon cancer [ICD-11: 2B90.Z]	66.88%	71.67%

Target Name	ChEMBL-ID	Indication	Probability	Model accuracy
receptor				
Platelet-derived growth factor receptor	T53524	Colorectal cancer [ICD-11: 2B91.Z]	66.88%	71.67%
Platelet-derived growth factor receptor	T53524	Diabetic complication [ICD-11: 5A2Y]	66.88%	71.67%
Platelet-derived growth factor receptor	T53524	Gastric adenocarcinoma [ICD-11: 2B72]	66.88%	71.67%
Platelet-derived growth factor receptor	T53524	Gastrointestinal cancer [ICD-11: 2C11]	66.88%	71.67%
Platelet-derived growth factor receptor	T53524	Gastrointestinal stromal tumour [ICD-11: 2B5B]	66.88%	71.67%
Platelet-derived growth factor receptor	T53524	Glioblastoma multiforme [ICD-11: 2A00.0]	66.88%	71.67%
Platelet-derived growth factor receptor	T53524	Gram-positive bacterial infection [ICD-11: 1B74-1G40]	66.88%	71.67%
Platelet-derived growth factor receptor	T53524	Gram-positive bacterial infection [ICD-11: 1B74-1G40]	66.88%	71.67%
Platelet-derived growth factor receptor	T53524	Hepatocellular carcinoma [ICD-11: 2C12.02]	66.88%	71.67%
Platelet-derived growth factor receptor	T53524	Lung cancer [ICD-11: 2C25.0]	66.88%	71.67%
Platelet-derived growth factor receptor	T53524	Mast cell leukaemia [ICD-11: 2A21.00]	66.88%	71.67%
Platelet-derived growth factor receptor	T53524	Multiple myeloma [ICD-11: 2A83]	66.88%	71.67%
Platelet-derived growth factor receptor	T53524	Multiple myeloma [ICD-11: 2A83]	66.88%	71.67%
Platelet-derived growth factor receptor	T53524	Myelodysplastic syndrome [ICD-11: 2A37]	66.88%	71.67%
Platelet-derived growth factor receptor	T53524	Myelodysplastic syndrome [ICD-11: 2A37]	66.88%	71.67%
Platelet-derived growth factor receptor	T53524	Non-small-cell lung cancer [ICD-11: 2C25.Y]	66.88%	71.67%
Platelet-derived growth factor receptor	T53524	Non-small-cell lung cancer [ICD-11: 2C25.Y]	66.88%	71.67%
Platelet-derived growth factor receptor	T53524	Pancreatic cancer [ICD-11: 2C10]	66.88%	71.67%
Platelet-derived growth factor receptor	T53524	Prostate cancer [ICD-11: 2C82.0]	66.88%	71.67%
Platelet-derived growth factor receptor	T53524	Recurrent glioblastoma [ICD-11: 2A00.00]	66.88%	71.67%
Platelet-derived growth factor receptor	T53524	Renal cell carcinoma [ICD-11: 2C90]	66.88%	71.67%
Platelet-derived growth factor receptor	T53524	Soft tissue sarcoma [ICD-11: 2B57]	66.88%	71.67%
Platelet-derived growth factor receptor	T53524	Solid tumour/cancer [ICD-11: 2A00-2F9Z]	66.88%	71.67%
Platelet-derived growth factor receptor	T53524	Solid tumour/cancer [ICD-11: 2A00-2F9Z]	66.88%	71.67%
Platelet-derived growth factor receptor	T53524	Systemic mastocytosis [ICD-11: 2A21.0]	66.88%	71.67%
Platelet-derived growth factor receptor	T53524	Thrombocytopenia [ICD-11: 3B64]	66.88%	71.67%

Target Name	ChEMBL-ID	Indication	Probability	Model accuracy
Platelet-derived growth factor receptor	T53524	Thrombocytopenia [ICD-11: 3B64]	66.88%	71.67%
Voltage-gated N-type calcium channel alpha-1B subunit	T38338	Anxiety disorder [ICD-11: 6B00-6B0Z]	66.02%	97.14%
Voltage-gated N-type calcium channel alpha-1B subunit	T38338	Cardiac failure [ICD-11: BD10-BD13]	66.02%	97.14%
Voltage-gated N-type calcium channel alpha-1B subunit	T38338	High blood pressure [ICD-11: BA00]	66.02%	97.14%
Voltage-gated N-type calcium channel alpha-1B subunit	T38338	Migraine [ICD-11: 8A80]	66.02%	97.14%
Voltage-gated N-type calcium channel alpha-1B subunit	T38338	Neuropathic pain [ICD-11: 8E43.0]	66.02%	97.14%
Voltage-gated N-type calcium channel alpha-1B subunit	T38338	Pain [ICD-11: MG30-MG3Z]	66.02%	97.14%
Voltage-gated N-type calcium channel alpha-1B subunit	T38338	Reperfusion injury [ICD-11: ND56.Z]	66.02%	97.14%
Voltage-gated N-type calcium channel alpha-1B subunit	T38338	Traumatic brain injury [ICD-11: NA07.Z]	66.02%	97.14%
Voltage-gated N-type calcium channel alpha-1B subunit	T38338	Vascular purpura [ICD-11: 3B60]	66.02%	97.14%
Muscarinic acetylcholine receptor M4	T20709	Asthma [ICD-11: CA23]	65.05%	94.08%
Muscarinic acetylcholine receptor M4	T20709	Attention deficit hyperactivity disorder [ICD-11: 6A05.Z]	65.05%	94.08%
Muscarinic acetylcholine receptor M4	T20709	bronchial hyperreactivity [ICD-11: CB40]	65.05%	94.08%
Muscarinic acetylcholine receptor M4	T20709	Glaucoma/ocular hypertension [ICD-11: 9C61]	65.05%	94.08%
Muscarinic acetylcholine receptor M4	T20709	Mydriasis [ICD-11: LA11.62]	65.05%	94.08%
Muscarinic acetylcholine receptor M4	T20709	Nausea [ICD-11: MD90]	65.05%	94.08%
Amine oxidase, copper containing	T69619	Asthma [ICD-11: CA23]	64.83%	94%
Amine oxidase, copper containing	T69619	Atopic dermatitis [ICD-11: EA80]	64.83%	94%
Amine oxidase, copper containing	T69619	Central nervous system disease [ICD-11: 8A04-8D87]	64.83%	94%
Amine oxidase, copper containing	T69619	Cerebrovascular ischaemia [ICD-11: 8B1Z]	64.83%	94%
Amine oxidase, copper containing	T69619	Diabetic complication [ICD-11: 5A2Y]	64.83%	94%
Amine oxidase, copper containing	T69619	Diabetic macular edema [ICD-11: 9B71.02]	64.83%	94%
Amine oxidase, copper containing	T69619	Diabetic nephropathy [ICD-11: GB61.Z]	64.83%	94%
Amine oxidase, copper containing	T69619	Hypertension [ICD-11: BA00-BA04]	64.83%	94%
Amine oxidase, copper containing	T69619	Inflammation [ICD-11: 1A00-CA43.1]	64.83%	94%
Amine oxidase, copper containing	T69619	Psoriasis vulgaris [ICD-11: EA90]	64.83%	94%
Amine oxidase, copper containing	T69619	Pulmonary disease [ICD-11: 1B10-1F85]	64.83%	94%
Amine oxidase, copper containing	T69619	Vascular disease [ICD-11: BE2Z]	64.83%	94%
Glycine receptor subunit alpha-1	T50269	Muscle spasm [ICD-11: MB47.3]	63.93%	90.71%
Pregnane X receptor	T82702	Arteriosclerosis [ICD-11: BD40]	63.88%	94.73%

Target Name	ChEMBL-ID	Indication	Probability	Model accuracy
Maternal embryonic leucine zipper kinase	T96892	Acute lymphoblastic leukaemia [ICD-11: 2A85]	62.47%	81.58%
Maternal embryonic leucine zipper kinase	T96892	Acute lymphoblastic leukaemia [ICD-11: 2A85]	62.47%	81.58%
Maternal embryonic leucine zipper kinase	T96892	Acute myeloid leukaemia [ICD-11: 2A60]	62.47%	81.58%
Maternal embryonic leucine zipper kinase	T96892	Acute myeloid leukaemia [ICD-11: 2A60]	62.47%	81.58%
Maternal embryonic leucine zipper kinase	T96892	Breast cancer [ICD-11: 2C60-2C65]	62.47%	81.58%
Maternal embryonic leucine zipper kinase	T96892	Breast cancer [ICD-11: 2C60-2C65]	62.47%	81.58%
Maternal embryonic leucine zipper kinase	T96892	Chronic lymphocytic leukaemia [ICD-11: 2A82.0]	62.47%	81.58%
Maternal embryonic leucine zipper kinase	T96892	Chronic lymphocytic leukaemia [ICD-11: 2A82.0]	62.47%	81.58%
Maternal embryonic leucine zipper kinase	T96892	Myelodysplastic syndrome [ICD-11: 2A37]	62.47%	81.58%
Maternal embryonic leucine zipper kinase	T96892	Myelodysplastic syndrome [ICD-11: 2A37]	62.47%	81.58%
Maternal embryonic leucine zipper kinase	T96892	Myeloproliferative neoplasm [ICD-11: 2A20]	62.47%	81.58%
Maternal embryonic leucine zipper kinase	T96892	Myeloproliferative neoplasm [ICD-11: 2A20]	62.47%	81.58%
Protein tyrosine kinase 2 beta	T07087	Osteoporosis [ICD-11: FB83.0]	62.03%	91.03%
Toll-like receptor 4	T81443	Allergy [ICD-11: 4A80-4A85]	61.02%	92.5%
Toll-like receptor 4	T81443	Autoimmune diabetes [ICD-11: 5A10]	61.02%	92.5%
Toll-like receptor 4	T81443	Hepatitis virus infection [ICD-11: 1E50-1E51]	61.02%	92.5%
Toll-like receptor 4	T81443	Melanoma [ICD-11: 2C30]	61.02%	92.5%
Toll-like receptor 4	T81443	Non-hodgkin lymphoma [ICD-11: 2B33.5]	61.02%	92.5%
Toll-like receptor 4	T81443	Prostate cancer [ICD-11: 2C82.0]	61.02%	92.5%
Toll-like receptor 4	T81443	Sepsis [ICD-11: 1G40-1G41]	61.02%	92.5%
Toll-like receptor 4	T81443	Solid tumour/cancer [ICD-11: 2A00-2F9Z]	61.02%	92.5%
11-beta-hydroxysteroid dehydrogenase 2	T43721	Type-2 diabetes [ICD-11: 5A11]	60.5%	94.78%
Sodium channel protein type IV alpha subunit	T02546	Musculoskeletal disorder [ICD-11: FA00-FC0Z]	59.7%	92.32%
Sodium channel protein type IV alpha subunit	T02546	Neurological disorder [ICD-11: 6B60]	59.7%	92.32%
Sodium channel protein type IV alpha subunit	T02546	Pain [ICD-11: MG30-MG3Z]	59.7%	92.32%
Methionine aminopeptidase 2	T75596	Autoimmune diabetes [ICD-11: 5A10]	59.44%	97.28%
Methionine aminopeptidase 2	T75596	Obesity [ICD-11: 5B81]	59.44%	97.28%
Methionine aminopeptidase 2	T75596	Solid tumour/cancer [ICD-11: 2A00-2F9Z]	59.44%	97.28%
Leukotriene A4 hydrolase	T03691	Acute myeloid leukaemia [ICD-11: 2A60]	58.75%	97.86%

Target Name	ChEMBL-ID	Indication	Probability	Model accuracy
Leukotriene A4 hydrolase	T03691	Chronic blistering skin disorder [ICD-11: ME63.3]	58.75%	97.86%
Leukotriene A4 hydrolase	T03691	Inflammation [ICD-11: 1A00-CA43.1]	58.75%	97.86%
Leukotriene A4 hydrolase	T03691	Myocardial infarction [ICD-11: BA41-BA43]	58.75%	97.86%
C-X-C chemokine receptor type 4	T96079	Acute lymphoblastic leukaemia [ICD-11: 2A85]	57.88%	93.1%
C-X-C chemokine receptor type 4	T96079	Acute myeloid leukaemia [ICD-11: 2A60]	57.88%	93.1%
C-X-C chemokine receptor type 4	T96079	Autoimmune diabetes [ICD-11: 5A10]	57.88%	93.1%
C-X-C chemokine receptor type 4	T96079	B-cell chronic lymphocytic leukaemia [ICD-11: 2A82.00]	57.88%	93.1%
C-X-C chemokine receptor type 4	T96079	Bone marrow transplantation [ICD-11: QB63.6]	57.88%	93.1%
C-X-C chemokine receptor type 4	T96079	Breast cancer [ICD-11: 2C60-2C65]	57.88%	93.1%
C-X-C chemokine receptor type 4	T96079	Constitutional neutropenia [ICD-11: 4B00.0]	57.88%	93.1%
C-X-C chemokine receptor type 4	T96079	Haematological malignancy [ICD-11: 2B33.Y]	57.88%	93.1%
C-X-C chemokine receptor type 4	T96079	Human immunodeficiency virus infection [ICD-11: 1C62]	57.88%	93.1%
C-X-C chemokine receptor type 4	T96079	Macular degeneration [ICD-11: 9B78.3]	57.88%	93.1%
C-X-C chemokine receptor type 4	T96079	Melanoma [ICD-11: 2C30]	57.88%	93.1%
C-X-C chemokine receptor type 4	T96079	Merkel cell carcinoma [ICD-11: 2C34]	57.88%	93.1%
C-X-C chemokine receptor type 4	T96079	Multiple myeloma [ICD-11: 2A83]	57.88%	93.1%
C-X-C chemokine receptor type 4	T96079	Myelodysplastic syndrome [ICD-11: 2A37]	57.88%	93.1%
C-X-C chemokine receptor type 4	T96079	Non-hodgkin lymphoma [ICD-11: 2B33.5]	57.88%	93.1%
C-X-C chemokine receptor type 4	T96079	Pancreatic cancer [ICD-11: 2C10]	57.88%	93.1%
C-X-C chemokine receptor type 4	T96079	Peripheral vascular disease [ICD-11: BD4Z]	57.88%	93.1%
C-X-C chemokine receptor type 4	T96079	Psoriasis vulgaris [ICD-11: EA90]	57.88%	93.1%
C-X-C chemokine receptor type 4	T96079	Recurring respiratory infection [ICD-11: CA07-CA45]	57.88%	93.1%
C-X-C chemokine receptor type 4	T96079	Renal cell carcinoma [ICD-11: 2C90]	57.88%	93.1%
C-X-C chemokine receptor type 4	T96079	Sarcoma [ICD-11: 2A60-2C35]	57.88%	93.1%
C-X-C chemokine receptor type 4	T96079	Solid tumour/cancer [ICD-11: 2A00-2F9Z]	57.88%	93.1%
Integrin alpha-5/beta-1	T29879	Allergic rhinitis [ICD-11: CA08.0]	57.58%	96.39%
Integrin alpha-5/beta-1	T29879	Allergy [ICD-11: 4A80-4A85]	57.58%	96.39%
Integrin alpha-5/beta-1	T29879	Asthma [ICD-11: CA23]	57.58%	96.39%
Integrin alpha-5/beta-1	T29879	Macular degeneration [ICD-11: 9B78.3]	57.58%	96.39%
Integrin alpha-5/beta-1	T29879	Macular degeneration [ICD-11: 9B78.3]	57.58%	96.39%
Integrin alpha-5/beta-1	T29879	Multiple sclerosis [ICD-11: 8A40]	57.58%	96.39%
Integrin alpha-5/beta-1	T29879	Non-small-cell lung cancer [ICD-11:	57.58%	96.39%

Target Name	ChEMBL-ID	Indication	Probability	Model accuracy
		2C25.Y]		
Nitric oxide synthase, inducible	T02703	Artery stenosis [ICD-11: BD52]	55.24%	94.8%
Nitric oxide synthase, inducible	T02703	Asthma [ICD-11: CA23]	55.24%	94.8%
Nitric oxide synthase, inducible	T02703	Cardiovascular disease [ICD-11: BA00-BE2Z]	55.24%	94.8%
Nitric oxide synthase, inducible	T02703	Coronary artery disease [ICD-11: BA80]	55.24%	94.8%
Nitric oxide synthase, inducible	T02703	Diabetic kidney disease [ICD-11: GB61.Z]	55.24%	94.8%
Nitric oxide synthase, inducible	T02703	Diabetic retinopathy [ICD-11: 9B71.0]	55.24%	94.8%
Nitric oxide synthase, inducible	T02703	Diarrhea [ICD-11: ME05.1]	55.24%	94.8%
Nitric oxide synthase, inducible	T02703	Endotoxic shock [ICD-11: 1G41]	55.24%	94.8%
Nitric oxide synthase, inducible	T02703	Inflammation [ICD-11: 1A00-CA43.1]	55.24%	94.8%
Nitric oxide synthase, inducible	T02703	Insulin-resistant disorder [ICD-11: 5A44]	55.24%	94.8%
Nitric oxide synthase, inducible	T02703	Osteoarthritis [ICD-11: FA00-FA05]	55.24%	94.8%
Nitric oxide synthase, inducible	T02703	Postoperative pain [ICD-11: MG30-MG3Z]	55.24%	94.8%
Nitric oxide synthase, inducible	T02703	Rheumatoid arthritis [ICD-11: FA20]	55.24%	94.8%
Nitric oxide synthase, inducible	T02703	Sepsis [ICD-11: 1G40-1G41]	55.24%	94.8%
Nitric oxide synthase, inducible	T02703	Solid tumour/cancer [ICD-11: 2A00-2F9Z]	55.24%	94.8%
Fibroblast growth factor receptor 1	T47101	Actinic keratosis [ICD-11: EK90.0]	54.84%	98.47%
Fibroblast growth factor receptor 1	T47101	Acute myeloid leukaemia [ICD-11: 2A60]	54.84%	98.47%
Fibroblast growth factor receptor 1	T47101	Alzheimer disease [ICD-11: 8A20]	54.84%	98.47%
Fibroblast growth factor receptor 1	T47101	Bladder cancer [ICD-11: 2C94]	54.84%	98.47%
Fibroblast growth factor receptor 1	T47101	Breast cancer [ICD-11: 2C60-2C65]	54.84%	98.47%
Fibroblast growth factor receptor 1	T47101	Colorectal cancer [ICD-11: 2B91.Z]	54.84%	98.47%
Fibroblast growth factor receptor 1	T47101	Coronary heart disease [ICD-11: BA80.Z]	54.84%	98.47%
Fibroblast growth factor receptor 1	T47101	Dermatological disease [ICD-11: DA24.Y]	54.84%	98.47%
Fibroblast growth factor receptor 1	T47101	Diabetic complication [ICD-11: 5A2Y]	54.84%	98.47%
Fibroblast growth factor receptor 1	T47101	Endometrial cancer [ICD-11: 2C76]	54.84%	98.47%
Fibroblast growth factor receptor 1	T47101	Gram-positive bacterial infection [ICD-11: 1B74-1G40]	54.84%	98.47%
Fibroblast growth factor receptor 1	T47101	Idiopathic pulmonary fibrosis [ICD-11: CB03.4]	54.84%	98.47%
Fibroblast growth factor receptor 1	T47101	Intrahepatic cholangiocarcinoma [ICD-11: 2C12.10]	54.84%	98.47%
Fibroblast growth factor receptor 1	T47101	Lymphoma [ICD-11: 2A80-2A86]	54.84%	98.47%
Fibroblast growth factor receptor 1	T47101	Multiple myeloma [ICD-11: 2A83]	54.84%	98.47%
Fibroblast growth factor receptor 1	T47101	Myelodysplastic syndrome [ICD-11: 2A37]	54.84%	98.47%
Fibroblast growth factor receptor 1	T47101	Myeloproliferative syndrome [ICD-11: 2A22]	54.84%	98.47%
Fibroblast growth factor receptor 1	T47101	Non-small-cell lung cancer [ICD-11:	54.84%	98.47%

Target Name	ChEMBL-ID	Indication	Probability	Model accuracy
		2C25.Y]		
Fibroblast growth factor receptor 1	T47101	Ovarian cancer [ICD-11: 2C73]	54.84%	98.47%
Fibroblast growth factor receptor 1	T47101	Peripheral arterial disease [ICD-11: BD4Z]	54.84%	98.47%
Fibroblast growth factor receptor 1	T47101	Retinoblastoma [ICD-11: 2D02.2]	54.84%	98.47%
Fibroblast growth factor receptor 1	T47101	Solid tumour/cancer [ICD-11: 2A00-2F9Z]	54.84%	98.47%
Fibroblast growth factor receptor 1	T47101	Thrombocytopenia [ICD-11: 3B64]	54.84%	98.47%
Fibroblast growth factor receptor 1	T47101	Type-2 diabetes [ICD-11: 5A11]	54.84%	98.47%
Signal transducer and activator of transcription 1-alpha/beta	T64205	Skin infection [ICD-11: 1F28-1G0Z]	54.4%	72.62%
PI3-kinase p110-delta subunit	T67849	Arthritis [ICD-11: FA20]	54.23%	96.47%
PI3-kinase p110-delta subunit	T67849	Breast cancer [ICD-11: 2C60-2C65]	54.23%	96.47%
PI3-kinase p110-delta subunit	T67849	Chronic lymphocytic leukaemia [ICD-11: 2A82.0]	54.23%	96.47%
PI3-kinase p110-delta subunit	T67849	Coronavirus Disease 2019 (COVID-19) [ICD-11: 1D6Y]	54.23%	96.47%
PI3-kinase p110-delta subunit	T67849	Cutaneous T-cell lymphoma [ICD-11: 2B01]	54.23%	96.47%
PI3-kinase p110-delta subunit	T67849	Diffuse large B-cell lymphoma [ICD-11: 2A81]	54.23%	96.47%
PI3-kinase p110-delta subunit	T67849	Follicular lymphoma [ICD-11: 2A80]	54.23%	96.47%
PI3-kinase p110-delta subunit	T67849	Follicular lymphoma [ICD-11: 2A80]	54.23%	96.47%
PI3-kinase p110-delta subunit	T67849	Hematologic tumour [ICD-11: 2B33.Y]	54.23%	96.47%
PI3-kinase p110-delta subunit	T67849	Lymphoma [ICD-11: 2A80-2A86]	54.23%	96.47%
PI3-kinase p110-delta subunit	T67849	Mantle cell lymphoma [ICD-11: 2A85.5]	54.23%	96.47%
PI3-kinase p110-delta subunit	T67849	Marginal zone lymphoma [ICD-11: 2A85.0]	54.23%	96.47%
PI3-kinase p110-delta subunit	T67849	Myeloid leukaemia [ICD-11: 2B33.1]	54.23%	96.47%
PI3-kinase p110-delta subunit	T67849	Non-hodgkin lymphoma [ICD-11: 2B33.5]	54.23%	96.47%
PI3-kinase p110-delta subunit	T67849	Solid tumour/cancer [ICD-11: 2A00-2F9Z]	54.23%	96.47%
DNA topoisomerase I	T09826	Acquired immune deficiency syndrome [ICD-11: 1C62.3]	53.76%	97%
DNA topoisomerase I	T09826	Acute lymphoblastic leukaemia [ICD-11: 2A85]	53.76%	97%
DNA topoisomerase I	T09826	Bacterial infection [ICD-11: 1A00-1C4Z]	53.76%	97%
DNA topoisomerase I	T09826	Bladder cancer [ICD-11: 2C94]	53.76%	97%
DNA topoisomerase I	T09826	Breast cancer [ICD-11: 2C60-2C65]	53.76%	97%
DNA topoisomerase I	T09826	Colorectal cancer [ICD-11: 2B91.Z]	53.76%	97%
DNA topoisomerase I	T09826	Esophageal cancer [ICD-11: 2B70]	53.76%	97%
DNA topoisomerase I	T09826	Gastric adenocarcinoma [ICD-11: 2B72]	53.76%	97%
DNA topoisomerase I	T09826	Glioblastoma multiforme [ICD-11: 2A00.0]	53.76%	97%
DNA topoisomerase I	T09826	Human immunodeficiency virus infection [ICD-11: 1C62]	53.76%	97%

Target Name	ChEMBL-ID	Indication	Probability	Model accuracy
DNA topoisomerase I	T09826	Lung cancer [ICD-11: 2C25.0]	53.76%	97%
DNA topoisomerase I	T09826	Lymphoma [ICD-11: 2A80-2A86]	53.76%	97%
DNA topoisomerase I	T09826	Metastatic colorectal cancer [ICD-11: 2B91]	53.76%	97%
DNA topoisomerase I	T09826	Myelodysplastic syndrome [ICD-11: 2A37]	53.76%	97%
DNA topoisomerase I	T09826	Ovarian cancer [ICD-11: 2C73]	53.76%	97%
DNA topoisomerase I	T09826	Renal cell carcinoma [ICD-11: 2C90]	53.76%	97%
DNA topoisomerase I	T09826	Small-cell lung cancer [ICD-11: 2C25.Y]	53.76%	97%
DNA topoisomerase I	T09826	Solid tumour/cancer [ICD-11: 2A00-2F9Z]	53.76%	97%
Bromodomain testis-specific protein	T90985	Testicular cancer [ICD-11: 2C80]	53.66%	89.76%
Ephrin type-B receptor 2	T73756	Pancreatic cancer [ICD-11: 2C10]	52.5%	78%
Ephrin type-B receptor 2	T73756	Solid tumour/cancer [ICD-11: 2A00-2F9Z]	52.5%	78%
Tissue factor pathway inhibitor	T78890	Factor IX deficiency [ICD-11: 3B11]	52.15%	97.33%
Tissue factor pathway inhibitor	T78890	Sepsis [ICD-11: 1G40-1G41]	52.15%	97.33%
Histone deacetylase 2	T51191	Dermatitis [ICD-11: EA80-EA89]	50.54%	94.75%
Histone deacetylase 2	T51191	Solid tumour/cancer [ICD-11: 2A00-2F9Z]	50.54%	94.75%
Signal transducer and activator of transcription 3	T29130	Brain cancer [ICD-11: 2A00]	50.14%	82.69%
Signal transducer and activator of transcription 3	T29130	Chronic lymphocytic leukaemia [ICD-11: 2A82.0]	50.14%	82.69%
Signal transducer and activator of transcription 3	T29130	Hepatocellular carcinoma [ICD-11: 2C12.02]	50.14%	82.69%
Signal transducer and activator of transcription 3	T29130	Immune System disease [ICD-11: 4A01-4B41]	50.14%	82.69%
Signal transducer and activator of transcription 3	T29130	Inflammation [ICD-11: 1A00-CA43.1]	50.14%	82.69%
Signal transducer and activator of transcription 3	T29130	Multiple myeloma [ICD-11: 2A83]	50.14%	82.69%
Signal transducer and activator of transcription 3	T29130	Psoriasis vulgaris [ICD-11: EA90]	50.14%	82.69%
Signal transducer and activator of transcription 3	T29130	Recurrent glioblastoma [ICD-11: 2A00.00]	50.14%	82.69%
Signal transducer and activator of transcription 3	T29130	Solid tumour/cancer [ICD-11: 2A00-2F9Z]	50.14%	82.69%
Signal transducer and activator of transcription 3	T29130	Ulcerative colitis [ICD-11: DD71]	50.14%	82.69%
