# Supplementary material for: 3,4-Dihydropyrimidin-2(1H)-One Hybrids as Phenotypic Anti-Breast Cancer Agents: Synthesis, Colony-Formation Profiling and Computational Target Deconvolution
Source: Int J Mol Sci. 2026 Oct 27;27(21):9618. doi: 10.3390/ijms27219618 (PMC13763226; doi:10.3390/ijms27219618)
Supplement: Supplementary file 1 [file ijms-27-09618-s001.zip › Super-PRED_exported_data.zip!/Targets_4e.pdf]

Exported data

Target Name	ChEMBL-ID	Indication	Probability	Model accuracy
Cathepsin D	T67102	Hypertension [ICD-11: BA00-BA04]	96.75%	98.95%
Cathepsin D	T67102	Multiple sclerosis [ICD-11: 8A40]	96.75%	98.95%
Dihydrofolate reductase	T17345	Acute bacterial skin infection [ICD-11: 1C41]	94.81%	89.92%
Dihydrofolate reductase	T17345	Bacterial infection [ICD-11: 1A00-1C4Z]	94.81%	89.92%
Dihydrofolate reductase	T17345	Bladder cancer [ICD-11: 2C94]	94.81%	89.92%
Dihydrofolate reductase	T17345	Inflammatory bowel disease [ICD-11: DD72]	94.81%	89.92%
Dihydrofolate reductase	T17345	Pneumonia [ICD-11: CA40]	94.81%	89.92%
Dihydrofolate reductase	T17345	Pulmonary and extrapulmonary tuberculosis [ICD-11: 1B10.Z]	94.81%	89.92%
Dihydrofolate reductase	T17345	Rheumatoid arthritis [ICD-11: FA20]	94.81%	89.92%
Dihydrofolate reductase	T17345	Solid tumour/cancer [ICD-11: 2A00-2F9Z]	94.81%	89.92%
Dihydrofolate reductase	T17345	Urinary tract infection [ICD-11: GC08]	94.81%	89.92%
G-protein coupled bile acid receptor 1	T86273	Metabolic disorder [ICD-11: 5C50-5D2Z]	88.52%	93.65%
G-protein coupled bile acid receptor 1	T86273	Type-2 diabetes [ICD-11: 5A11]	88.52%	93.65%
GABA-A receptor; alpha-1/beta-2/gamma-2	T08910	Alcohol dependence [ICD-11: 6C40.2]	83.64%	92.67%
GABA-A receptor; alpha-1/beta-2/gamma-2	T08910	Anaesthesia [ICD-11: 9A78.6]	83.64%	92.67%
GABA-A receptor; alpha-1/beta-2/gamma-2	T08910	Anxiety disorder [ICD-11: 6B00-6B0Z]	83.64%	92.67%
GABA-A receptor; alpha-1/beta-2/gamma-2	T08910	Anxiety disorder [ICD-11: 6B00-6B0Z]	83.64%	92.67%
GABA-A receptor; alpha-1/beta-2/gamma-2	T08910	Anxiety disorder [ICD-11: 6B00-6B0Z]	83.64%	92.67%
GABA-A receptor; alpha-1/beta-2/gamma-2	T08910	Chronic pain [ICD-11: MG30]	83.64%	92.67%
GABA-A receptor; alpha-1/beta-2/gamma-2	T08910	Cystitis [ICD-11: GC00]	83.64%	92.67%
GABA-A receptor; alpha-1/beta-2/gamma-2	T08910	Depression [ICD-11: 6A70-6A7Z]	83.64%	92.67%
GABA-A receptor; alpha-1/beta-2/gamma-2	T08910	Depression [ICD-11: 6A70-6A7Z]	83.64%	92.67%
GABA-A receptor; alpha-1/beta-2/gamma-2	T08910	Depression [ICD-11: 6A70-6A7Z]	83.64%	92.67%
GABA-A receptor; alpha-1/beta-2/gamma-2	T08910	Epilepsy [ICD-11: 8A60-8A68]	83.64%	92.67%
GABA-A receptor; alpha-1/beta-2/gamma-2	T08910	Epileptic seizures [ICD-11: 8A61-8A6Z]	83.64%	92.67%
GABA-A receptor; alpha-1/beta-2/gamma-2	T08910	Epileptic seizures [ICD-11: 8A61-8A6Z]	83.64%	92.67%
GABA-A receptor; alpha-1/beta-2/gamma-2	T08910	Epileptic seizures [ICD-11: 8A61-8A6Z]	83.64%	92.67%
GABA-A receptor; alpha-1/beta-2/gamma-2	T08910	Essential tremor or related tremors [ICD-11: 8A04.1]	83.64%	92.67%

Target Name	ChEMBL-ID	Indication	Probability	Model accuracy
GABA-A receptor; alpha-1/beta-2/ gamma-2	T08910	Essential tremor or related tremors [ICD-11: 8A04.1]	83.64%	92.67%
GABA-A receptor; alpha-1/beta-2/ gamma-2	T08910	Essential tremor or related tremors [ICD-11: 8A04.1]	83.64%	92.67%
GABA-A receptor; alpha-1/beta-2/ gamma-2	T08910	Fibromyalgia [ICD-11: MG30.01]	83.64%	92.67%
GABA-A receptor; alpha-1/beta-2/ gamma-2	T08910	Headache [ICD-11: 8A80-8A84]	83.64%	92.67%
GABA-A receptor; alpha-1/beta-2/ gamma-2	T08910	Inflammation [ICD-11: 1A00-CA43.1]	83.64%	92.67%
GABA-A receptor; alpha-1/beta-2/ gamma-2	T08910	Inflammation [ICD-11: 1A00-CA43.1]	83.64%	92.67%
GABA-A receptor; alpha-1/beta-2/ gamma-2	T08910	Inflammation [ICD-11: 1A00-CA43.1]	83.64%	92.67%
GABA-A receptor; alpha-1/beta-2/ gamma-2	T08910	Inflammatory bowel disease [ICD-11: DD72]	83.64%	92.67%
GABA-A receptor; alpha-1/beta-2/ gamma-2	T08910	Inflammatory bowel disease [ICD-11: DD72]	83.64%	92.67%
GABA-A receptor; alpha-1/beta-2/ gamma-2	T08910	Inflammatory bowel disease [ICD-11: DD72]	83.64%	92.67%
GABA-A receptor; alpha-1/beta-2/ gamma-2	T08910	Insomnia [ICD-11: 7A00-7A0Z]	83.64%	92.67%
GABA-A receptor; alpha-1/beta-2/ gamma-2	T08910	Insomnia [ICD-11: 7A00-7A0Z]	83.64%	92.67%
GABA-A receptor; alpha-1/beta-2/ gamma-2	T08910	Intractable insomnia [ICD-11: 7A00]	83.64%	92.67%
GABA-A receptor; alpha-1/beta-2/ gamma-2	T08910	Ischemic reperfusion injury [ICD-11: DB98.B]	83.64%	92.67%
GABA-A receptor; alpha-1/beta-2/ gamma-2	T08910	Malaria [ICD-11: 1F40-1F45]	83.64%	92.67%
GABA-A receptor; alpha-1/beta-2/ gamma-2	T08910	Muscle spasm [ICD-11: MB47.3]	83.64%	92.67%
GABA-A receptor; alpha-1/beta-2/ gamma-2	T08910	Muscle spasm [ICD-11: MB47.3]	83.64%	92.67%
GABA-A receptor; alpha-1/beta-2/ gamma-2	T08910	Muscle spasm [ICD-11: MB47.3]	83.64%	92.67%
GABA-A receptor; alpha-1/beta-2/ gamma-2	T08910	Postpartum depression [ICD-11: 6E20.0]	83.64%	92.67%
GABA-A receptor; alpha-1/beta-2/ gamma-2	T08910	Postpartum depression [ICD-11: 6E20.0]	83.64%	92.67%
GABA-A receptor; alpha-1/beta-2/ gamma-2	T08910	Postpartum depression [ICD-11: 6E20.0]	83.64%	92.67%
GABA-A receptor; alpha-1/beta-2/ gamma-2	T08910	Premenstrual syndrome [ICD-11: GA34.40]	83.64%	92.67%
GABA-A receptor; alpha-1/beta-2/ gamma-2	T08910	Premenstrual syndrome [ICD-11: GA34.40]	83.64%	92.67%
GABA-A receptor; alpha-1/beta-2/ gamma-2	T08910	Premenstrual syndrome [ICD-11: GA34.40]	83.64%	92.67%
GABA-A receptor; alpha-1/beta-2/ gamma-2	T08910	Respiratory distress syndrome [ICD-11: CB00]	83.64%	92.67%
GABA-A receptor; alpha-1/beta-2/ gamma-2	T08910	Status epilepticus seizure [ICD-11: 8A66.1Y]	83.64%	92.67%
GABA-A receptor; alpha-1/beta-2/	T08910	Status epilepticus seizure [ICD-11:	83.64%	92.67%

Target Name	ChEMBL-ID	Indication	Probability	Model accuracy
gamma-2		8A66.1Y]		
GABA-A receptor; alpha-1/beta-2/ gamma-2	T08910	Status epilepticus seizure [ICD-11: 8A66.1Y]	83.64%	92.67%
Casein kinase II alpha/beta	T51565	Cholangiocarcinoma [ICD-11: 2C12.10]	83.63%	99.23%
Casein kinase II alpha/beta	T51565	Solid tumour/cancer [ICD-11: 2A00-2F9Z]	83.63%	99.23%
Beta-glucuronidase	T96413	Mucopolysaccharidosis [ICD-11: 5C56.3]	83.54%	77.68%
Beta-glucuronidase	T96413	Periodontal disease [ICD-11: DA0C]	83.54%	77.68%
Sodium/hydrogen exchanger 1	T82028	Angina pectoris [ICD-11: BA40]	82.59%	90.24%
Sodium/hydrogen exchanger 1	T82028	Cardiac arrhythmias [ICD-11: BC9Z]	82.59%	90.24%
Sodium/hydrogen exchanger 1	T82028	Heart arrhythmia [ICD-11: BC65]	82.59%	90.24%
Sodium/hydrogen exchanger 1	T82028	Myocardial infarction [ICD-11: BA41-BA43]	82.59%	90.24%
Adenosine A2b receptor	T86679	Acute and chronic heart failure [ICD-11: BD1Z]	82.1%	98.59%
Adenosine A2b receptor	T86679	Alzheimer disease [ICD-11: 8A20]	82.1%	98.59%
Adenosine A2b receptor	T86679	Apnea [ICD-11: MD11.0]	82.1%	98.59%
Adenosine A2b receptor	T86679	Asthma [ICD-11: CA23]	82.1%	98.59%
Adenosine A2b receptor	T86679	Herpes simplex virus infection [ICD-11: 1F00]	82.1%	98.59%
Adenosine A2b receptor	T86679	Hypertension [ICD-11: BA00-BA04]	82.1%	98.59%
Adenosine A2b receptor	T86679	Multiple sclerosis [ICD-11: 8A40]	82.1%	98.59%
Adenosine A2b receptor	T86679	Non-insulin dependent diabetes [ICD-11: 5A11]	82.1%	98.59%
Adenosine A2b receptor	T86679	Non-small-cell lung cancer [ICD-11: 2C25.Y]	82.1%	98.59%
Adenosine A2b receptor	T86679	Parkinson disease [ICD-11: 8A00.0]	82.1%	98.59%
Adenosine A2b receptor	T86679	Paroxysmal supraventricular tachycardia [ICD-11: BC81.Z]	82.1%	98.59%
Adenosine A2b receptor	T86679	Solid tumour/cancer [ICD-11: 2A00-2F9Z]	82.1%	98.59%
Aurora kinase B/Inner centromere protein	T46781	Acute myeloid leukaemia [ICD-11: 2A60]	81.69%	97.5%
Aurora kinase B/Inner centromere protein	T46781	Diabetic complication [ICD-11: 5A2Y]	81.69%	97.5%
Aurora kinase B/Inner centromere protein	T46781	Haematological malignancy [ICD-11: 2B33.Y]	81.69%	97.5%
Aurora kinase B/Inner centromere protein	T46781	Prostate cancer [ICD-11: 2C82.0]	81.69%	97.5%
Aurora kinase B/Inner centromere protein	T46781	Solid tumour/cancer [ICD-11: 2A00-2F9Z]	81.69%	97.5%
Protein-tyrosine phosphatase 2C	T13057	Solid tumour/cancer [ICD-11: 2A00-2F9Z]	80.46%	94.42%
Glycine receptor subunit alpha-1	T50269	Muscle spasm [ICD-11: MB47.3]	79.65%	90.71%
Tyrosine-protein kinase receptor UFO	T82383	Acute myeloid leukaemia [ICD-11: 2A60]	79.5%	90.95%
Tyrosine-protein kinase receptor UFO	T82383	Breast cancer [ICD-11: 2C60-2C65]	79.5%	90.95%
Tyrosine-protein kinase receptor UFO	T82383	Metastatic malignant neoplasm [ICD-11: 2D50-2E09]	79.5%	90.95%

Target Name	ChEMBL-ID	Indication	Probability	Model accuracy
Tyrosine-protein kinase receptor UFO	T82383	Multiple myeloma [ICD-11: 2A83]	79.5%	90.95%
Tyrosine-protein kinase receptor UFO	T82383	Myelodysplastic syndrome [ICD-11: 2A37]	79.5%	90.95%
Tyrosine-protein kinase receptor UFO	T82383	Non-small-cell lung cancer [ICD-11: 2C25.Y]	79.5%	90.95%
Tyrosine-protein kinase receptor UFO	T82383	Solid tumour/cancer [ICD-11: 2A00-2F9Z]	79.5%	90.95%
Amine oxidase, copper containing	T69619	Asthma [ICD-11: CA23]	78.74%	94%
Amine oxidase, copper containing	T69619	Atopic dermatitis [ICD-11: EA80]	78.74%	94%
Amine oxidase, copper containing	T69619	Central nervous system disease [ICD-11: 8A04-8D87]	78.74%	94%
Amine oxidase, copper containing	T69619	Cerebrovascular ischaemia [ICD-11: 8B1Z]	78.74%	94%
Amine oxidase, copper containing	T69619	Diabetic complication [ICD-11: 5A2Y]	78.74%	94%
Amine oxidase, copper containing	T69619	Diabetic macular edema [ICD-11: 9B71.02]	78.74%	94%
Amine oxidase, copper containing	T69619	Diabetic nephropathy [ICD-11: GB61.Z]	78.74%	94%
Amine oxidase, copper containing	T69619	Hypertension [ICD-11: BA00-BA04]	78.74%	94%
Amine oxidase, copper containing	T69619	Inflammation [ICD-11: 1A00-CA43.1]	78.74%	94%
Amine oxidase, copper containing	T69619	Psoriasis vulgaris [ICD-11: EA90]	78.74%	94%
Amine oxidase, copper containing	T69619	Pulmonary disease [ICD-11: 1B10-1F85]	78.74%	94%
Amine oxidase, copper containing	T69619	Vascular disease [ICD-11: BE2Z]	78.74%	94%
Formyl peptide receptor 1	T87831	Inflammation [ICD-11: 1A00-CA43.1]	78.12%	93.56%
Formyl peptide receptor 1	T87831	Peptic ulcer [ICD-11: DA61]	78.12%	93.56%
DNA topoisomerase II alpha	T17048	Solid tumour/cancer [ICD-11: 2A00-2F9Z]	74.67%	89%
Nicotinamide phosphoribosyltransferase	T54582	leukaemia [ICD-11: 2A60-2B33]	74.53%	96.25%
Nicotinamide phosphoribosyltransferase	T54582	Solid tumour/cancer [ICD-11: 2A00-2F9Z]	74.53%	96.25%
Cathepsin L	T41141	Abdominal aortic aneurysm [ICD-11: BD50.4]	74.29%	96.61%
Cathepsin L	T41141	Asthma [ICD-11: CA23]	74.29%	96.61%
Cathepsin L	T41141	Bone cancer [ICD-11: 2B5Z]	74.29%	96.61%
Cathepsin L	T41141	Cancer related pain [ICD-11: MG30]	74.29%	96.61%
Cathepsin L	T41141	Chronic obstructive pulmonary disease [ICD-11: CA22]	74.29%	96.61%
Cathepsin L	T41141	Cirrhosis [ICD-11: DB93]	74.29%	96.61%
Cathepsin L	T41141	Coagulation defect [ICD-11: 3B10.0]	74.29%	96.61%
Cathepsin L	T41141	Coronavirus infection [ICD-11: 1D92]	74.29%	96.61%
Cathepsin L	T41141	Gastrointestinal disease [ICD-11: DE2Z]	74.29%	96.61%
Cathepsin L	T41141	Glioma [ICD-11: 2A00.0]	74.29%	96.61%
Cathepsin L	T41141	Hair loss [ICD-11: ED70]	74.29%	96.61%
Cathepsin L	T41141	Henipavirus Encephalitis [ICD-11: 1D63]	74.29%	96.61%
Cathepsin L	T41141	Middle East Respiratory Syndrome	74.29%	96.61%

Target Name	ChEMBL-ID	Indication	Probability	Model accuracy
		(MERS) [ICD-11: 1D64]		
Cathepsin L	T41141	Multiple sclerosis [ICD-11: 8A40]	74.29%	96.61%
Cathepsin L	T41141	Psoriasis vulgaris [ICD-11: EA90]	74.29%	96.61%
Cathepsin L	T41141	Rheumatoid arthritis [ICD-11: FA20]	74.29%	96.61%
Cathepsin L	T41141	Severe acute respiratory syndrome (SARS) [ICD-11: 1D65]	74.29%	96.61%
Cathepsin L	T41141	Solid tumour/cancer [ICD-11: 2A00-2F9Z]	74.29%	96.61%
HERG	T20251	Angina pectoris [ICD-11: BA40]	73.84%	89.76%
HERG	T20251	Cardiac arrhythmias [ICD-11: BC9Z]	73.84%	89.76%
HERG	T20251	Cardiac failure [ICD-11: BD10-BD13]	73.84%	89.76%
HERG	T20251	Heart arrhythmia [ICD-11: BC65]	73.84%	89.76%
HERG	T20251	Malaria [ICD-11: 1F40-1F45]	73.84%	89.76%
HERG	T20251	Multiple sclerosis [ICD-11: 8A40]	73.84%	89.76%
HERG	T20251	Ovarian cancer [ICD-11: 2C73]	73.84%	89.76%
HERG	T20251	Pain [ICD-11: MG30-MG3Z]	73.84%	89.76%
HERG	T20251	Sleep-wake disorder [ICD-11: 7A00-7B2Z]	73.84%	89.76%
Cysteinyl leukotriene receptor 2	T74238	Asthma [ICD-11: CA23]	72.61%	98%
PI3-kinase p110-alpha/p85-alpha	T80276	Breast cancer [ICD-11: 2C60-2C65]	72.26%	94.33%
PI3-kinase p110-alpha/p85-alpha	T80276	Follicular lymphoma [ICD-11: 2A80]	72.26%	94.33%
PI3-kinase p110-alpha/p85-alpha	T80276	Non-hodgkin lymphoma [ICD-11: 2B33.5]	72.26%	94.33%
PI3-kinase p110-alpha/p85-alpha	T80276	Prostate cancer [ICD-11: 2C82.0]	72.26%	94.33%
PI3-kinase p110-alpha/p85-alpha	T80276	Solid tumour/cancer [ICD-11: 2A00-2F9Z]	72.26%	94.33%
Histone deacetylase 4	T63816	Advanced stage follicular lymphoma [ICD-11: 2A80]	71.42%	92.97%
Histone deacetylase 4	T63816	Huntington disease [ICD-11: 8A01.10]	71.42%	92.97%
Histone deacetylase 4	T63816	Solid tumour/cancer [ICD-11: 2A00-2F9Z]	71.42%	92.97%
PI3-kinase p110-delta subunit	T67849	Arthritis [ICD-11: FA20]	71.22%	96.47%
PI3-kinase p110-delta subunit	T67849	Breast cancer [ICD-11: 2C60-2C65]	71.22%	96.47%
PI3-kinase p110-delta subunit	T67849	Chronic lymphocytic leukaemia [ICD-11: 2A82.0]	71.22%	96.47%
PI3-kinase p110-delta subunit	T67849	Coronavirus Disease 2019 (COVID-19) [ICD-11: 1D6Y]	71.22%	96.47%
PI3-kinase p110-delta subunit	T67849	Cutaneous T-cell lymphoma [ICD-11: 2B01]	71.22%	96.47%
PI3-kinase p110-delta subunit	T67849	Diffuse large B-cell lymphoma [ICD-11: 2A81]	71.22%	96.47%
PI3-kinase p110-delta subunit	T67849	Follicular lymphoma [ICD-11: 2A80]	71.22%	96.47%
PI3-kinase p110-delta subunit	T67849	Follicular lymphoma [ICD-11: 2A80]	71.22%	96.47%
PI3-kinase p110-delta subunit	T67849	Hematologic tumour [ICD-11: 2B33.Y]	71.22%	96.47%
PI3-kinase p110-delta subunit	T67849	Lymphoma [ICD-11: 2A80-2A86]	71.22%	96.47%
PI3-kinase p110-delta subunit	T67849	Mantle cell lymphoma [ICD-11: 2A85.5]	71.22%	96.47%
PI3-kinase p110-delta subunit	T67849	Marginal zone lymphoma [ICD-11:	71.22%	96.47%

Target Name	ChEMBL-ID	Indication	Probability	Model accuracy
		2A85.0]		
PI3-kinase p110-delta subunit	T67849	Myeloid leukaemia [ICD-11: 2B33.1]	71.22%	96.47%
PI3-kinase p110-delta subunit	T67849	Non-hodgkin lymphoma [ICD-11: 2B33.5]	71.22%	96.47%
PI3-kinase p110-delta subunit	T67849	Solid tumour/cancer [ICD-11: 2A00-2F9Z]	71.22%	96.47%
Acetylcholinesterase	T30082	Alcohol dependence [ICD-11: 6C40.2]	71.06%	94.45%
Acetylcholinesterase	T30082	Alzheimer disease [ICD-11: 8A20]	71.06%	94.45%
Acetylcholinesterase	T30082	Chronic glaucoma [ICD-11: 9C61.0Z]	71.06%	94.45%
Acetylcholinesterase	T30082	Cognitive impairment [ICD-11: 6D71]	71.06%	94.45%
Acetylcholinesterase	T30082	Dementia [ICD-11: 6D80-6D86]	71.06%	94.45%
Acetylcholinesterase	T30082	Functional dyspepsia [ICD-11: DD90.3]	71.06%	94.45%
Acetylcholinesterase	T30082	Helminth infection [ICD-11: 1F90.0]	71.06%	94.45%
Acetylcholinesterase	T30082	Myasthenia gravis [ICD-11: 8C6Y]	71.06%	94.45%
Acetylcholinesterase	T30082	Neurodegenerative disorder [ICD-11: 8A20-8A23]	71.06%	94.45%
Acetylcholinesterase	T30082	Open-angle glaucoma [ICD-11: 9C61]	71.06%	94.45%
Acetylcholinesterase	T30082	Pain [ICD-11: MG30-MG3Z]	71.06%	94.45%
Acetylcholinesterase	T30082	Parkinson disease [ICD-11: 8A00.0]	71.06%	94.45%
Acetylcholinesterase	T30082	Pediculus capitis infestation [ICD-11: 1G00.0]	71.06%	94.45%
Acetylcholinesterase	T30082	Poisoning due to pesticides and chemicals [ICD-11: NE6Z]	71.06%	94.45%
Acetylcholinesterase	T30082	Solid tumour/cancer [ICD-11: 2A00-2F9Z]	71.06%	94.45%
Acetylcholinesterase	T30082	Urinary dysfunction [ICD-11: GC2Z]	71.06%	94.45%
Acetylcholinesterase	T30082	Xerostomia [ICD-11: DA02.1]	71.06%	94.45%
Sodium channel protein type IV alpha subunit	T02546	Musculoskeletal disorder [ICD-11: FA00-FC0Z]	70.49%	92.32%
Sodium channel protein type IV alpha subunit	T02546	Neurological disorder [ICD-11: 6B60]	70.49%	92.32%
Sodium channel protein type IV alpha subunit	T02546	Pain [ICD-11: MG30-MG3Z]	70.49%	92.32%
Transient receptor potential cation channel subfamily A member 1	T84040	Diabetic neuropathy [ICD-11: 8C0Z]	70.32%	92.17%
Transient receptor potential cation channel subfamily A member 1	T84040	Pain [ICD-11: MG30-MG3Z]	70.32%	92.17%
Transient receptor potential cation channel subfamily A member 1	T84040	Throat irritation [ICD-11: CA0Y]	70.32%	92.17%
Histone deacetylase 2	T51191	Dermatitis [ICD-11: EA80-EA89]	69.5%	94.75%
Histone deacetylase 2	T51191	Solid tumour/cancer [ICD-11: 2A00-2F9Z]	69.5%	94.75%
Platelet-derived growth factor receptor alpha	T53524	Acute myelogenous leukaemia [ICD-11: 2A41]	68.2%	91.07%
Platelet-derived growth factor receptor alpha	T53524	Acute myeloid leukaemia [ICD-11: 2A60]	68.2%	91.07%
Platelet-derived growth factor receptor alpha	T53524	Breast cancer [ICD-11: 2C60-2C65]	68.2%	91.07%

Target Name	ChEMBL-ID	Indication	Probability	Model accuracy
Platelet-derived growth factor receptor alpha	T53524	Colon cancer [ICD-11: 2B90.Z]	68.2%	91.07%
Platelet-derived growth factor receptor alpha	T53524	Colorectal cancer [ICD-11: 2B91.Z]	68.2%	91.07%
Platelet-derived growth factor receptor alpha	T53524	Gastric adenocarcinoma [ICD-11: 2B72]	68.2%	91.07%
Platelet-derived growth factor receptor alpha	T53524	Gastrointestinal cancer [ICD-11: 2C11]	68.2%	91.07%
Platelet-derived growth factor receptor alpha	T53524	Gastrointestinal stromal tumour [ICD-11: 2B5B]	68.2%	91.07%
Platelet-derived growth factor receptor alpha	T53524	Glioblastoma multiforme [ICD-11: 2A00.0]	68.2%	91.07%
Platelet-derived growth factor receptor alpha	T53524	Gram-positive bacterial infection [ICD-11: 1B74-1G40]	68.2%	91.07%
Platelet-derived growth factor receptor alpha	T53524	Lung cancer [ICD-11: 2C25.0]	68.2%	91.07%
Platelet-derived growth factor receptor alpha	T53524	Mast cell leukaemia [ICD-11: 2A21.00]	68.2%	91.07%
Platelet-derived growth factor receptor alpha	T53524	Multiple myeloma [ICD-11: 2A83]	68.2%	91.07%
Platelet-derived growth factor receptor alpha	T53524	Myelodysplastic syndrome [ICD-11: 2A37]	68.2%	91.07%
Platelet-derived growth factor receptor alpha	T53524	Non-small-cell lung cancer [ICD-11: 2C25.Y]	68.2%	91.07%
Platelet-derived growth factor receptor alpha	T53524	Pancreatic cancer [ICD-11: 2C10]	68.2%	91.07%
Platelet-derived growth factor receptor alpha	T53524	Prostate cancer [ICD-11: 2C82.0]	68.2%	91.07%
Platelet-derived growth factor receptor alpha	T53524	Recurrent glioblastoma [ICD-11: 2A00.00]	68.2%	91.07%
Platelet-derived growth factor receptor alpha	T53524	Soft tissue sarcoma [ICD-11: 2B57]	68.2%	91.07%
Platelet-derived growth factor receptor alpha	T53524	Solid tumour/cancer [ICD-11: 2A00-2F9Z]	68.2%	91.07%
Platelet-derived growth factor receptor alpha	T53524	Systemic mastocytosis [ICD-11: 2A21.0]	68.2%	91.07%
Platelet-derived growth factor receptor alpha	T53524	Thrombocytopenia [ICD-11: 3B64]	68.2%	91.07%
Delta opioid receptor	T58992	Alcohol dependence [ICD-11: 6C40.2]	68.13%	99.35%
Delta opioid receptor	T58992	Anxiety disorder [ICD-11: 6B00-6B0Z]	68.13%	99.35%
Delta opioid receptor	T58992	Back pain [ICD-11: ME84.Z]	68.13%	99.35%
Delta opioid receptor	T58992	Bladder disease [ICD-11: DC11-DC1Z]	68.13%	99.35%
Delta opioid receptor	T58992	Cerebral vasospasm [ICD-11: BA85.Z]	68.13%	99.35%
Delta opioid receptor	T58992	Cough [ICD-11: MD12]	68.13%	99.35%
Delta opioid receptor	T58992	Diarrhea [ICD-11: ME05.1]	68.13%	99.35%
Delta opioid receptor	T58992	Diarrhea-predominant irritable bowel syndrome [ICD-11: DD91.01]	68.13%	99.35%
Delta opioid receptor	T58992	Ischemic heart disease [ICD-11:	68.13%	99.35%

Target Name	ChEMBL-ID	Indication	Probability	Model accuracy
		BA40-BA6Z]		
Delta opioid receptor	T58992	Major depressive disorder [ICD-11: 6A70.3]	68.13%	99.35%
Delta opioid receptor	T58992	Migraine [ICD-11: 8A80]	68.13%	99.35%
Delta opioid receptor	T58992	Obesity [ICD-11: 5B81]	68.13%	99.35%
Delta opioid receptor	T58992	Opioid dependence [ICD-11: 6C43.2Z]	68.13%	99.35%
Delta opioid receptor	T58992	Overactive bladder [ICD-11: GC50.0]	68.13%	99.35%
Delta opioid receptor	T58992	Pain [ICD-11: MG30-MG3Z]	68.13%	99.35%
Delta opioid receptor	T58992	Premature ejaculation [ICD-11: HA03.0Z]	68.13%	99.35%
Delta opioid receptor	T58992	Rheumatoid arthritis [ICD-11: FA20]	68.13%	99.35%
Delta opioid receptor	T58992	Substance use disorder [ICD-11: 6C4Z]	68.13%	99.35%
Delta opioid receptor	T58992	Urinary incontinence [ICD-11: MF50.2]	68.13%	99.35%
Glutamate receptor ionotropic, AMPA 2	T42392	Diabetic neuropathy [ICD-11: 8C0Z]	68.1%	86.92%
Glutamate receptor ionotropic, AMPA 2	T42392	Parkinson disease [ICD-11: 8A00.0]	68.1%	86.92%
Sphingosine 1-phosphate receptor Edg-8	T50089	Multiple sclerosis [ICD-11: 8A40]	67.94%	100%
C-X-C chemokine receptor type 4	T96079	Acute lymphoblastic leukaemia [ICD-11: 2A85]	67.91%	93.1%
C-X-C chemokine receptor type 4	T96079	Acute myeloid leukaemia [ICD-11: 2A60]	67.91%	93.1%
C-X-C chemokine receptor type 4	T96079	Autoimmune diabetes [ICD-11: 5A10]	67.91%	93.1%
C-X-C chemokine receptor type 4	T96079	B-cell chronic lymphocytic leukaemia [ICD-11: 2A82.00]	67.91%	93.1%
C-X-C chemokine receptor type 4	T96079	Bone marrow transplantation [ICD-11: QB63.6]	67.91%	93.1%
C-X-C chemokine receptor type 4	T96079	Breast cancer [ICD-11: 2C60-2C65]	67.91%	93.1%
C-X-C chemokine receptor type 4	T96079	Constitutional neutropenia [ICD-11: 4B00.0]	67.91%	93.1%
C-X-C chemokine receptor type 4	T96079	Haematological malignancy [ICD-11: 2B33.Y]	67.91%	93.1%
C-X-C chemokine receptor type 4	T96079	Human immunodeficiency virus infection [ICD-11: 1C62]	67.91%	93.1%
C-X-C chemokine receptor type 4	T96079	Macular degeneration [ICD-11: 9B78.3]	67.91%	93.1%
C-X-C chemokine receptor type 4	T96079	Melanoma [ICD-11: 2C30]	67.91%	93.1%
C-X-C chemokine receptor type 4	T96079	Merkel cell carcinoma [ICD-11: 2C34]	67.91%	93.1%
C-X-C chemokine receptor type 4	T96079	Multiple myeloma [ICD-11: 2A83]	67.91%	93.1%
C-X-C chemokine receptor type 4	T96079	Myelodysplastic syndrome [ICD-11: 2A37]	67.91%	93.1%
C-X-C chemokine receptor type 4	T96079	Non-hodgkin lymphoma [ICD-11: 2B33.5]	67.91%	93.1%
C-X-C chemokine receptor type 4	T96079	Pancreatic cancer [ICD-11: 2C10]	67.91%	93.1%
C-X-C chemokine receptor type 4	T96079	Peripheral vascular disease [ICD-11:	67.91%	93.1%

Target Name	ChEMBL-ID	Indication	Probability	Model accuracy
		BD4Z]		
C-X-C chemokine receptor type 4	T96079	Psoriasis vulgaris [ICD-11: EA90]	67.91%	93.1%
C-X-C chemokine receptor type 4	T96079	Recurring respiratory infection [ICD-11: CA07-CA45]	67.91%	93.1%
C-X-C chemokine receptor type 4	T96079	Renal cell carcinoma [ICD-11: 2C90]	67.91%	93.1%
C-X-C chemokine receptor type 4	T96079	Sarcoma [ICD-11: 2A60-2C35]	67.91%	93.1%
C-X-C chemokine receptor type 4	T96079	Solid tumour/cancer [ICD-11: 2A00-2F9Z]	67.91%	93.1%
Protein tyrosine kinase 2 beta	T07087	Osteoporosis [ICD-11: FB83.0]	67.78%	91.03%
DNA-(apurinic or apyrimidinic site) lyase	T13348	Glioma [ICD-11: 2A00.0]	67.37%	91.11%
DNA-(apurinic or apyrimidinic site) lyase	T13348	Melanoma [ICD-11: 2C30]	67.37%	91.11%
DNA-(apurinic or apyrimidinic site) lyase	T13348	Ocular cancer [ICD-11: 2D00-2D07]	67.37%	91.11%
DNA-(apurinic or apyrimidinic site) lyase	T13348	Solid tumour/cancer [ICD-11: 2A00-2F9Z]	67.37%	91.11%
Muscarinic acetylcholine receptor M4	T20709	Asthma [ICD-11: CA23]	67.29%	94.08%
Muscarinic acetylcholine receptor M4	T20709	Attention deficit hyperactivity disorder [ICD-11: 6A05.Z]	67.29%	94.08%
Muscarinic acetylcholine receptor M4	T20709	bronchial hyperreactivity [ICD-11: CB40]	67.29%	94.08%
Muscarinic acetylcholine receptor M4	T20709	Glaucoma/ocular hypertension [ICD-11: 9C61]	67.29%	94.08%
Muscarinic acetylcholine receptor M4	T20709	Mydriasis [ICD-11: LA11.62]	67.29%	94.08%
Muscarinic acetylcholine receptor M4	T20709	Nausea [ICD-11: MD90]	67.29%	94.08%
TGF-beta receptor type II	T49989	Solid tumour/cancer [ICD-11: 2A00-2F9Z]	66.95%	88.18%
TGF-beta receptor type II	T49989	Wound healing [ICD-11: EL8Y]	66.95%	88.18%
C5a anaphylatoxin chemotactic receptor	T15439	Atopic dermatitis [ICD-11: EA80]	66.21%	92.62%
C5a anaphylatoxin chemotactic receptor	T15439	Eosinophilic granulomatosis with polyangiitis [ICD-11: 4A44.A2]	66.21%	92.62%
C5a anaphylatoxin chemotactic receptor	T15439	Hereditary haemolytic anemia [ICD-11: 3A10.3]	66.21%	92.62%
C5a anaphylatoxin chemotactic receptor	T15439	Inflammation [ICD-11: 1A00-CA43.1]	66.21%	92.62%
C5a anaphylatoxin chemotactic receptor	T15439	Rheumatoid arthritis [ICD-11: FA20]	66.21%	92.62%
C5a anaphylatoxin chemotactic receptor	T15439	Vasculitis [ICD-11: 4A44]	66.21%	92.62%
Histone deacetylase 8	T28887	Solid tumour/cancer [ICD-11: 2A00-2F9Z]	66.15%	93.99%
Pregnane X receptor	T82702	Arteriosclerosis [ICD-11: BD40]	65.9%	94.73%
ADAM10	T31902	Breast cancer [ICD-11: 2C60-2C65]	65.31%	97.5%
ADAM10	T31902	Solid tumour/cancer [ICD-11: 2A00-2F9Z]	65.31%	97.5%
DNA topoisomerase I	T09826	Acquired immune deficiency syndrome [ICD-11: 1C62.3]	64.25%	97%

Target Name	ChEMBL-ID	Indication	Probability	Model accuracy
DNA topoisomerase I	T09826	Acute lymphoblastic leukaemia [ICD-11: 2A85]	64.25%	97%
DNA topoisomerase I	T09826	Bacterial infection [ICD-11: 1A00-1C4Z]	64.25%	97%
DNA topoisomerase I	T09826	Bladder cancer [ICD-11: 2C94]	64.25%	97%
DNA topoisomerase I	T09826	Breast cancer [ICD-11: 2C60-2C65]	64.25%	97%
DNA topoisomerase I	T09826	Colorectal cancer [ICD-11: 2B91.Z]	64.25%	97%
DNA topoisomerase I	T09826	Esophageal cancer [ICD-11: 2B70]	64.25%	97%
DNA topoisomerase I	T09826	Gastric adenocarcinoma [ICD-11: 2B72]	64.25%	97%
DNA topoisomerase I	T09826	Glioblastoma multiforme [ICD-11: 2A00.0]	64.25%	97%
DNA topoisomerase I	T09826	Human immunodeficiency virus infection [ICD-11: 1C62]	64.25%	97%
DNA topoisomerase I	T09826	Lung cancer [ICD-11: 2C25.0]	64.25%	97%
DNA topoisomerase I	T09826	Lymphoma [ICD-11: 2A80-2A86]	64.25%	97%
DNA topoisomerase I	T09826	Metastatic colorectal cancer [ICD-11: 2B91]	64.25%	97%
DNA topoisomerase I	T09826	Myelodysplastic syndrome [ICD-11: 2A37]	64.25%	97%
DNA topoisomerase I	T09826	Ovarian cancer [ICD-11: 2C73]	64.25%	97%
DNA topoisomerase I	T09826	Renal cell carcinoma [ICD-11: 2C90]	64.25%	97%
DNA topoisomerase I	T09826	Small-cell lung cancer [ICD-11: 2C25.Y]	64.25%	97%
DNA topoisomerase I	T09826	Solid tumour/cancer [ICD-11: 2A00-2F9Z]	64.25%	97%
Toll-like receptor 4	T81443	Allergy [ICD-11: 4A80-4A85]	62.84%	92.5%
Toll-like receptor 4	T81443	Autoimmune diabetes [ICD-11: 5A10]	62.84%	92.5%
Toll-like receptor 4	T81443	Hepatitis virus infection [ICD-11: 1E50-1E51]	62.84%	92.5%
Toll-like receptor 4	T81443	Melanoma [ICD-11: 2C30]	62.84%	92.5%
Toll-like receptor 4	T81443	Non-hodgkin lymphoma [ICD-11: 2B33.5]	62.84%	92.5%
Toll-like receptor 4	T81443	Prostate cancer [ICD-11: 2C82.0]	62.84%	92.5%
Toll-like receptor 4	T81443	Sepsis [ICD-11: 1G40-1G41]	62.84%	92.5%
Toll-like receptor 4	T81443	Solid tumour/cancer [ICD-11: 2A00-2F9Z]	62.84%	92.5%
Anandamide amidohydrolase	T11754	Anaesthesia [ICD-11: 9A78.6]	62.6%	97.53%
Anandamide amidohydrolase	T11754	Anxiety disorder [ICD-11: 6B00-6B0Z]	62.6%	97.53%
Anandamide amidohydrolase	T11754	Inflammation [ICD-11: 1A00-CA43.1]	62.6%	97.53%
Anandamide amidohydrolase	T11754	Liver disease [ICD-11: DB90-BD99]	62.6%	97.53%
Anandamide amidohydrolase	T11754	Major depressive disorder [ICD-11: 6A70.3]	62.6%	97.53%
Anandamide amidohydrolase	T11754	Neuropathic pain [ICD-11: 8E43.0]	62.6%	97.53%
Anandamide amidohydrolase	T11754	Pain [ICD-11: MG30-MG3Z]	62.6%	97.53%
Anandamide amidohydrolase	T11754	Social anxiety disorder [ICD-11: 6B04]	62.6%	97.53%
Tyrosine-protein kinase receptor RET	T60631	Acute lymphoblastic leukaemia	62.25%	91.79%

Target Name	ChEMBL-ID	Indication	Probability	Model accuracy
		[ICD-11: 2A85]		
Tyrosine-protein kinase receptor RET	T60631	Inflammatory bowel disease [ICD-11: DD72]	62.25%	91.79%
Tyrosine-protein kinase receptor RET	T60631	Metastatic colorectal cancer [ICD-11: 2B91]	62.25%	91.79%
Tyrosine-protein kinase receptor RET	T60631	Multiple myeloma [ICD-11: 2A83]	62.25%	91.79%
Tyrosine-protein kinase receptor RET	T60631	Myelodysplastic syndrome [ICD-11: 2A37]	62.25%	91.79%
Tyrosine-protein kinase receptor RET	T60631	Neurodegenerative disorder [ICD-11: 8A20-8A23]	62.25%	91.79%
Tyrosine-protein kinase receptor RET	T60631	Non-small-cell lung cancer [ICD-11: 2C25.Y]	62.25%	91.79%
Tyrosine-protein kinase receptor RET	T60631	Solid tumour/cancer [ICD-11: 2A00-2F9Z]	62.25%	91.79%
Tyrosine-protein kinase receptor RET	T60631	Thrombocytopenia [ICD-11: 3B64]	62.25%	91.79%
Tyrosine-protein kinase receptor RET	T60631	Thyroid cancer [ICD-11: 2D10]	62.25%	91.79%
11-beta-hydroxysteroid dehydrogenase 2	T43721	Type-2 diabetes [ICD-11: 5A11]	60.48%	94.78%
Signal transducer and activator of transcription 3	T29130	Brain cancer [ICD-11: 2A00]	60.34%	82.69%
Signal transducer and activator of transcription 3	T29130	Chronic lymphocytic leukaemia [ICD-11: 2A82.0]	60.34%	82.69%
Signal transducer and activator of transcription 3	T29130	Hepatocellular carcinoma [ICD-11: 2C12.02]	60.34%	82.69%
Signal transducer and activator of transcription 3	T29130	Immune System disease [ICD-11: 4A01-4B41]	60.34%	82.69%
Signal transducer and activator of transcription 3	T29130	Inflammation [ICD-11: 1A00-CA43.1]	60.34%	82.69%
Signal transducer and activator of transcription 3	T29130	Multiple myeloma [ICD-11: 2A83]	60.34%	82.69%
Signal transducer and activator of transcription 3	T29130	Psoriasis vulgaris [ICD-11: EA90]	60.34%	82.69%
Signal transducer and activator of transcription 3	T29130	Recurrent glioblastoma [ICD-11: 2A00.00]	60.34%	82.69%
Signal transducer and activator of transcription 3	T29130	Solid tumour/cancer [ICD-11: 2A00-2F9Z]	60.34%	82.69%
Signal transducer and activator of transcription 3	T29130	Ulcerative colitis [ICD-11: DD71]	60.34%	82.69%
Methionine aminopeptidase 2	T75596	Autoimmune diabetes [ICD-11: 5A10]	59.73%	97.28%
Methionine aminopeptidase 2	T75596	Obesity [ICD-11: 5B81]	59.73%	97.28%
Methionine aminopeptidase 2	T75596	Solid tumour/cancer [ICD-11: 2A00-2F9Z]	59.73%	97.28%
Ephrin type-B receptor 2	T73756	Pancreatic cancer [ICD-11: 2C10]	59.53%	78%
Ephrin type-B receptor 2	T73756	Solid tumour/cancer [ICD-11: 2A00-2F9Z]	59.53%	78%
Alkaline phosphatase, tissue-nonspecific isozyme	T09538	Colon cancer [ICD-11: 2B90.Z]	59.32%	85.4%
Alkaline phosphatase, tissue-nonspecific isozyme	T09538	Genetic disease [ICD-11: 8E02]	59.32%	85.4%
Alkaline phosphatase, tissue-nonspecific isozyme	T09538	Parasitic infection [ICD-11: 1D0Y-1G2Z]	59.32%	85.4%

Target Name	ChEMBL-ID	Indication	Probability	Model accuracy
Cyclin-dependent kinase 5/CDK5 activator 1	T20973	Obesity [ICD-11: 5B81]	58.67%	93.03%
Integrin alpha-5/beta-1	T29879	Allergic rhinitis [ICD-11: CA08.0]	58.57%	96.39%
Integrin alpha-5/beta-1	T29879	Allergy [ICD-11: 4A80-4A85]	58.57%	96.39%
Integrin alpha-5/beta-1	T29879	Asthma [ICD-11: CA23]	58.57%	96.39%
Integrin alpha-5/beta-1	T29879	Macular degeneration [ICD-11: 9B78.3]	58.57%	96.39%
Integrin alpha-5/beta-1	T29879	Macular degeneration [ICD-11: 9B78.3]	58.57%	96.39%
Integrin alpha-5/beta-1	T29879	Multiple sclerosis [ICD-11: 8A40]	58.57%	96.39%
Integrin alpha-5/beta-1	T29879	Non-small-cell lung cancer [ICD-11: 2C25.Y]	58.57%	96.39%
Glutaminase kidney isoform, mitochondrial	T86734	Breast cancer [ICD-11: 2C60-2C65]	56.79%	100%
Glutaminase kidney isoform, mitochondrial	T86734	leukaemia [ICD-11: 2A60-2B33]	56.79%	100%
Glutaminase kidney isoform, mitochondrial	T86734	Renal cell carcinoma [ICD-11: 2C90]	56.79%	100%
Glutaminase kidney isoform, mitochondrial	T86734	Solid tumour/cancer [ICD-11: 2A00-2F9Z]	56.79%	100%
Histone deacetylase 10	T94324	Solid tumour/cancer [ICD-11: 2A00-2F9Z]	56.4%	90.08%
G-protein coupled receptor 55	T87670	Attention deficit hyperactivity disorder [ICD-11: 6A05.Z]	54.35%	78.15%
Serotonin 2c (5-HT2c) receptor	T83813	Alcohol dependence [ICD-11: 6C40.2]	53.36%	89.62%
Serotonin 2c (5-HT2c) receptor	T83813	Alzheimer disease [ICD-11: 8A20]	53.36%	89.62%
Serotonin 2c (5-HT2c) receptor	T83813	Anxiety disorder [ICD-11: 6B00-6B0Z]	53.36%	89.62%
Serotonin 2c (5-HT2c) receptor	T83813	Attention deficit hyperactivity disorder [ICD-11: 6A05.Z]	53.36%	89.62%
Serotonin 2c (5-HT2c) receptor	T83813	Depression [ICD-11: 6A70-6A7Z]	53.36%	89.62%
Serotonin 2c (5-HT2c) receptor	T83813	Diabetic complication [ICD-11: 5A2Y]	53.36%	89.62%
Serotonin 2c (5-HT2c) receptor	T83813	Drug abuse [ICD-11: 6C4G.1Z]	53.36%	89.62%
Serotonin 2c (5-HT2c) receptor	T83813	Dyskinesia [ICD-11: MB47.4]	53.36%	89.62%
Serotonin 2c (5-HT2c) receptor	T83813	Generalized anxiety disorder [ICD-11: 6B00]	53.36%	89.62%
Serotonin 2c (5-HT2c) receptor	T83813	Hyperprolactinaemia [ICD-11: 5A60.1]	53.36%	89.62%
Serotonin 2c (5-HT2c) receptor	T83813	Major depressive disorder [ICD-11: 6A70.3]	53.36%	89.62%
Serotonin 2c (5-HT2c) receptor	T83813	Metabolic disorder [ICD-11: 5C50-5D2Z]	53.36%	89.62%
Serotonin 2c (5-HT2c) receptor	T83813	Migraine [ICD-11: 8A80]	53.36%	89.62%
Serotonin 2c (5-HT2c) receptor	T83813	Mood disorder [ICD-11: 6A60-6E23]	53.36%	89.62%
Serotonin 2c (5-HT2c) receptor	T83813	Neurological disorder [ICD-11: 6B60]	53.36%	89.62%
Serotonin 2c (5-HT2c) receptor	T83813	Non-hodgkin lymphoma [ICD-11: 2B33.5]	53.36%	89.62%
Serotonin 2c (5-HT2c) receptor	T83813	Obesity [ICD-11: 5B81]	53.36%	89.62%
Serotonin 2c (5-HT2c) receptor	T83813	Pain [ICD-11: MG30-MG3Z]	53.36%	89.62%

Target Name	ChEMBL-ID	Indication	Probability	Model accuracy
Serotonin 2c (5-HT2c) receptor	T83813	Parkinson disease [ICD-11: 8A00.0]	53.36%	89.62%
Serotonin 2c (5-HT2c) receptor	T83813	Primary insomnia [ICD-11: 7A00]	53.36%	89.62%
Serotonin 2c (5-HT2c) receptor	T83813	Psychotic disorder [ICD-11: 6A20-6A25]	53.36%	89.62%
Serotonin 2c (5-HT2c) receptor	T83813	Schizophrenia [ICD-11: 6A20]	53.36%	89.62%
Serotonin 2c (5-HT2c) receptor	T83813	Sleep-wake disorder [ICD-11: 7A00-7B2Z]	53.36%	89.62%
Glutathione S-transferase Pi	T21669	Myelodysplastic syndrome [ICD-11: 2A37]	53.21%	93.81%
Glutathione S-transferase Pi	T21669	Solid tumour/cancer [ICD-11: 2A00-2F9Z]	53.21%	93.81%
Cyclin-dependent kinase 4/cyclin D1	T85799	Breast cancer [ICD-11: 2C60-2C65]	52.99%	98.99%
Cyclin-dependent kinase 4/cyclin D1	T85799	Haematological malignancy [ICD-11: 2B33.Y]	52.99%	98.99%
Cyclin-dependent kinase 4/cyclin D1	T85799	Mantle cell lymphoma [ICD-11: 2A85.5]	52.99%	98.99%
Cyclin-dependent kinase 4/cyclin D1	T85799	Myelofibrosis [ICD-11: 2A20.2]	52.99%	98.99%
Cyclin-dependent kinase 4/cyclin D1	T85799	Psoriasis vulgaris [ICD-11: EA90]	52.99%	98.99%
Cyclin-dependent kinase 4/cyclin D1	T85799	Relapsed or refractory chronic lymphocytic leukaemia [ICD-11: 2A82.0]	52.99%	98.99%
Cyclin-dependent kinase 4/cyclin D1	T85799	Retinoblastoma [ICD-11: 2D02.2]	52.99%	98.99%
Cyclin-dependent kinase 4/cyclin D1	T85799	Schizophrenia [ICD-11: 6A20]	52.99%	98.99%
Cyclin-dependent kinase 4/cyclin D1	T85799	Small-cell lung cancer [ICD-11: 2C25.Y]	52.99%	98.99%
Cyclin-dependent kinase 4/cyclin D1	T85799	Solid tumour/cancer [ICD-11: 2A00-2F9Z]	52.99%	98.99%
Cyclin-dependent kinase 4/cyclin D1	T85799	Solid tumour/cancer [ICD-11: 2A00-2F9Z]	52.99%	98.99%
Tyrosine-protein kinase FYN	T17980	Chronic myelogenous leukaemia [ICD-11: 2A20.0]	52.57%	81.29%
Tyrosine-protein kinase FYN	T17980	Multiple myeloma [ICD-11: 2A83]	52.57%	81.29%
Tyrosine-protein kinase FYN	T17980	Solid tumour/cancer [ICD-11: 2A00-2F9Z]	52.57%	81.29%
Serotonin 7 (5-HT7) receptor	T79062	Alzheimer disease [ICD-11: 8A20]	52.5%	90.71%
Serotonin 7 (5-HT7) receptor	T79062	Attention deficit hyperactivity disorder [ICD-11: 6A05.Z]	52.5%	90.71%
Serotonin 7 (5-HT7) receptor	T79062	Bipolar disorder [ICD-11: 6A60]	52.5%	90.71%
Serotonin 7 (5-HT7) receptor	T79062	Major depressive disorder [ICD-11: 6A70.3]	52.5%	90.71%
Serotonin 7 (5-HT7) receptor	T79062	Parkinson disease [ICD-11: 8A00.0]	52.5%	90.71%
Serotonin 7 (5-HT7) receptor	T79062	Psychiatric disorder [ICD-11: 6E8Z]	52.5%	90.71%
Serotonin 7 (5-HT7) receptor	T79062	Schizophrenia [ICD-11: 6A20]	52.5%	90.71%
Serotonin 7 (5-HT7) receptor	T79062	Sleep-wake disorder [ICD-11: 7A00-7B2Z]	52.5%	90.71%
Cytochrome P450 3A4	T37848	Atopic dermatitis [ICD-11: EA80]	52.12%	91.19%
Cytochrome P450 3A4	T37848	Solid tumour/cancer [ICD-11: 2A00-2F9Z]	52.12%	91.19%
Leukotriene A4 hydrolase	T03691	Acute myeloid leukaemia [ICD-11: 2A60]	51.88%	97.86%
Leukotriene A4 hydrolase	T03691	Chronic blistering skin disorder	51.88%	97.86%

Target Name	ChEMBL-ID	Indication	Probability	Model accuracy
		[ICD-11: ME63.3]		
Leukotriene A4 hydrolase	T03691	Inflammation [ICD-11: 1A00-CA43.1]	51.88%	97.86%
Leukotriene A4 hydrolase	T03691	Myocardial infarction [ICD-11: BA41-BA43]	51.88%	97.86%
Cyclin-dependent kinase 1/cyclin B1	T49898	Acute lymphoblastic leukaemia [ICD-11: 2A85]	51.72%	91.24%
Cyclin-dependent kinase 1/cyclin B1	T49898	Allergic inflammation [ICD-11: 4A80-4A85]	51.72%	91.24%
Cyclin-dependent kinase 1/cyclin B1	T49898	Breast cancer [ICD-11: 2C60-2C65]	51.72%	91.24%
Cyclin-dependent kinase 1/cyclin B1	T49898	Graft rejection [ICD-11: NE84]	51.72%	91.24%
Cyclin-dependent kinase 1/cyclin B1	T49898	Haematological malignancy [ICD-11: 2B33.Y]	51.72%	91.24%
Cyclin-dependent kinase 1/cyclin B1	T49898	Mantle cell lymphoma [ICD-11: 2A85.5]	51.72%	91.24%
Cyclin-dependent kinase 1/cyclin B1	T49898	Nasopharyngeal carcinoma [ICD-11: 2B6B]	51.72%	91.24%
Cyclin-dependent kinase 1/cyclin B1	T49898	Non-small-cell lung cancer [ICD-11: 2C25.Y]	51.72%	91.24%
Cyclin-dependent kinase 1/cyclin B1	T49898	Obesity [ICD-11: 5B81]	51.72%	91.24%
Cyclin-dependent kinase 1/cyclin B1	T49898	Pain [ICD-11: MG30-MG3Z]	51.72%	91.24%
Cyclin-dependent kinase 1/cyclin B1	T49898	Solid tumour/cancer [ICD-11: 2A00-2F9Z]	51.72%	91.24%
Ghrelin receptor	T59604	Alcohol dependence [ICD-11: 6C40.2]	50.77%	92%
Ghrelin receptor	T59604	Anorexia nervosa cachexia [ICD-11: 6B80]	50.77%	92%
Ghrelin receptor	T59604	Cachexia [ICD-11: MG20]	50.77%	92%
Ghrelin receptor	T59604	Carbohydrate metabolism disorder [ICD-11: 5C51.Z]	50.77%	92%
Ghrelin receptor	T59604	Fibromyalgia [ICD-11: MG30.01]	50.77%	92%
Ghrelin receptor	T59604	Frailty [ICD-11: MG2A]	50.77%	92%
Ghrelin receptor	T59604	Functional bowel disorder [ICD-11: DD91]	50.77%	92%
Ghrelin receptor	T59604	Gastric motility disorder [ICD-11: DA21]	50.77%	92%
Ghrelin receptor	T59604	Gastrointestinal disease [ICD-11: DE2Z]	50.77%	92%
Ghrelin receptor	T59604	Gastroparesis [ICD-11: DA41.00]	50.77%	92%
Ghrelin receptor	T59604	Growth hormone deficiency [ICD-11: 5A61.3]	50.77%	92%
Ghrelin receptor	T59604	Ileus [ICD-11: DA93.0]	50.77%	92%
Ghrelin receptor	T59604	Metabolic disorder [ICD-11: 5C50-5D2Z]	50.77%	92%
Ghrelin receptor	T59604	Obesity [ICD-11: 5B81]	50.77%	92%
Ghrelin receptor	T59604	Postoperative ileus [ICD-11: DA91-DB30]	50.77%	92%
Ghrelin receptor	T59604	Solid tumour/cancer [ICD-11: 2A00-2F9Z]	50.77%	92%
Ghrelin receptor	T59604	Somatotropin deficiency [ICD-11: 5A61.0]	50.77%	92%
Ghrelin receptor	T59604	Type-2 diabetes [ICD-11: 5A11]	50.77%	92%

Target Name	ChEMBL-ID	Indication	Probability	Model accuracy
Multidrug resistance-associated protein 1	T11288	Gout [ICD-11: FA25]	50.49%	96.37%
Platelet-derived growth factor receptor	T53524	Acute myelogenous leukaemia [ICD-11: 2A41]	50.22%	71.67%
Platelet-derived growth factor receptor	T53524	Acute myeloid leukaemia [ICD-11: 2A60]	50.22%	71.67%
Platelet-derived growth factor receptor	T53524	Alzheimer disease [ICD-11: 8A20]	50.22%	71.67%
Platelet-derived growth factor receptor	T53524	Brain injury [ICD-11: NA07.Z]	50.22%	71.67%
Platelet-derived growth factor receptor	T53524	Breast cancer [ICD-11: 2C60-2C65]	50.22%	71.67%
Platelet-derived growth factor receptor	T53524	Colon cancer [ICD-11: 2B90.Z]	50.22%	71.67%
Platelet-derived growth factor receptor	T53524	Colorectal cancer [ICD-11: 2B91.Z]	50.22%	71.67%
Platelet-derived growth factor receptor	T53524	Diabetic complication [ICD-11: 5A2Y]	50.22%	71.67%
Platelet-derived growth factor receptor	T53524	Gastric adenocarcinoma [ICD-11: 2B72]	50.22%	71.67%
Platelet-derived growth factor receptor	T53524	Gastrointestinal cancer [ICD-11: 2C11]	50.22%	71.67%
Platelet-derived growth factor receptor	T53524	Gastrointestinal stromal tumour [ICD-11: 2B5B]	50.22%	71.67%
Platelet-derived growth factor receptor	T53524	Glioblastoma multiforme [ICD-11: 2A00.0]	50.22%	71.67%
Platelet-derived growth factor receptor	T53524	Gram-positive bacterial infection [ICD-11: 1B74-1G40]	50.22%	71.67%
Platelet-derived growth factor receptor	T53524	Gram-positive bacterial infection [ICD-11: 1B74-1G40]	50.22%	71.67%
Platelet-derived growth factor receptor	T53524	Hepatocellular carcinoma [ICD-11: 2C12.02]	50.22%	71.67%
Platelet-derived growth factor receptor	T53524	Lung cancer [ICD-11: 2C25.0]	50.22%	71.67%
Platelet-derived growth factor receptor	T53524	Mast cell leukaemia [ICD-11: 2A21.00]	50.22%	71.67%
Platelet-derived growth factor receptor	T53524	Multiple myeloma [ICD-11: 2A83]	50.22%	71.67%
Platelet-derived growth factor receptor	T53524	Multiple myeloma [ICD-11: 2A83]	50.22%	71.67%
Platelet-derived growth factor receptor	T53524	Myelodysplastic syndrome [ICD-11: 2A37]	50.22%	71.67%
Platelet-derived growth factor receptor	T53524	Myelodysplastic syndrome [ICD-11: 2A37]	50.22%	71.67%
Platelet-derived growth factor receptor	T53524	Non-small-cell lung cancer [ICD-11: 2C25.Y]	50.22%	71.67%
Platelet-derived growth factor receptor	T53524	Non-small-cell lung cancer [ICD-11: 2C25.Y]	50.22%	71.67%
Platelet-derived growth factor receptor	T53524	Pancreatic cancer [ICD-11: 2C10]	50.22%	71.67%
Platelet-derived growth factor receptor	T53524	Prostate cancer [ICD-11: 2C82.0]	50.22%	71.67%
Platelet-derived growth factor	T53524	Recurrent glioblastoma [ICD-11:	50.22%	71.67%

Target Name	ChEMBL-ID	Indication	Probability	Model accuracy
receptor		2A00.00]		
Platelet-derived growth factor receptor	T53524	Renal cell carcinoma [ICD-11: 2C90]	50.22%	71.67%
Platelet-derived growth factor receptor	T53524	Soft tissue sarcoma [ICD-11: 2B57]	50.22%	71.67%
Platelet-derived growth factor receptor	T53524	Solid tumour/cancer [ICD-11: 2A00-2F9Z]	50.22%	71.67%
Platelet-derived growth factor receptor	T53524	Solid tumour/cancer [ICD-11: 2A00-2F9Z]	50.22%	71.67%
Platelet-derived growth factor receptor	T53524	Systemic mastocytosis [ICD-11: 2A21.0]	50.22%	71.67%
Platelet-derived growth factor receptor	T53524	Thrombocytopenia [ICD-11: 3B64]	50.22%	71.67%
Platelet-derived growth factor receptor	T53524	Thrombocytopenia [ICD-11: 3B64]	50.22%	71.67%
Acyl-CoA desaturase	T10897	Bacterial infection [ICD-11: 1A00-1C4Z]	50.07%	97.5%
Acyl-CoA desaturase	T10897	Diabetic complication [ICD-11: 5A2Y]	50.07%	97.5%
Acyl-CoA desaturase	T10897	Lipid metabolism disorder [ICD-11: 5C52.Z]	50.07%	97.5%
Acyl-CoA desaturase	T10897	Type-2 diabetes [ICD-11: 5A11]	50.07%	97.5%
